# Larvicidal and adulticidal activity of essential oils from plants of the Lamiaceae family against the West Nile virus vector, *Culex pipiens* (Diptera: Culicidae)

**DOI:** 10.1016/j.sjbs.2022.103350

**Published:** 2022-06-16

**Authors:** Hanan Abo El-Kasem Bosly

**Affiliations:** Entomology Biology Department, Faculty of Science, Jazan University, PO Box 2097, Jizan 45142, Saudi Arabia

**Keywords:** Lavender, Peppermint, Rosemary, Essential oils, *Culex pipiens*, Insecticidal

## Abstract

*Culex pipiens* mosquitoes are the most widely distributed primary vector of the West Nile virus worldwide. Many attempts for investigation of botanical pesticides to avoid the development of pesticide resistance to conventional synthetic pesticides that are recognized as a threat to the diversity of ecosystems. The study aimed to determine the components of three essential oils of Lamiaceae family, lavender (*Lavandula angustifolia*), peppermint (*Mentha piperita* L.), and rosemary (*Rosmarinus officinalis* L.) by gas chromatography-mass spectrometry (GC–MS) analysis. Furthermore, aimed to validate the insecticidal activities of these oils as larvicidal agents against the third instar larvae of *Culex pipiens* using five different concentrations (62.5, 125, 250, 500, and 1000 ppm) for each oil in five replicates and as an adulticidal agent against approximately three-day-old female adults of *Cx. Pipiens* using 0.5, 1, 2, 4, and 5% concentrations in three replicates. The results generally showed a dose-related response. At 1000 ppm, rosemary oil showed the highest larvicidal (100%) (LC_50_, 214.97 ppm), followed by peppermint oil (92.00% mortality and LC_50_ (269.35 ppm). Lavender oil showed the lowest efficacy with 87.20% mortality and LC_50_ (301.11 ppm). At 5% oil concentration, the highest knockdown rate at 1 h was recorded for lavender oil (95.55%), followed by peppermint oil (88.89%) and lastly rosemary oil (84.44%). After 24 h, rosemary oil showed the lowest adult mortality rate (88.89%; LC_50_, 1.44%), while lavender and peppermint oils both showed a 100% mortality rate, with (LC_50_, 0.81% and 0.91%, respectively). The chemical constituents of the oils consisted of monoterpenes and sesquiterpenes that determined their insecticidal activities against the target insect stage. The study proposed that rosemary essential oil may be useful for the control of *Cx. pipiens* larvae as part of an integrated water treatment strategy, and lavender and peppermint oils may be used in an integrated plan for adult’s control.

## 1 Introduction

The Lamiaceae family called the mint family is characterized by economically important species of aromatic plants including 250 genera and 7825 species (Prakash et al., 2016). Their essential oils are obtained from different aerial parts (seeds, leaves, flowers, shoots, and fruits) or roots. These oils contain characteristic functional groups such as aldehydes, esters, phenols, ketones, alcohols, organic acids, hydrocarbons and terpenoids Essential oils may be preferred in the industrial economic sector due to the development of fewer toxic side products and economic viability (Nazar et al., 2022, Ramos da Silva et al., 2021).

The present study investigated three essential oils from three genera of the family Lamiaceae. The first was lavender oil from the flowering parts of *Lavandula angustifolia* L. This species showed anti-inflammatory, antibacterial, antiviral, and antifungal properties and anticancer activity as well as to provide mood disturbance relief (Cavanagh and Wilkinson, 2002, Denner, 2009, Rai et al., 2020). The second was peppermint oil from the aerial parts of *Mentha* × *piperita* L. which is a hybrid mint (*M. aquatica* × *M. spicata*). Peppermint oil is used in the health industry because of its wide spectrum of therapeutic properties. These include analgesic, antispasmodic, and antiemetic action, abdominal pain relief, antioxidant activity, and cytotoxicity against bacteria and fungi as well as insecticidal effects against many pests (da Silva Ramos et al., 2017, Kalemba and Synowiec, 2019, Mahendran and Rahman, 2020). The third was rosemary oil from the flowering tops of the plant *Rosmarinus officinalis* L. which has been shown to have anti-inflammatory properties with potential applications in inflammatory-related diseases. Furthermore, it exerts anti-depressive, antimicrobial, antioxidant, antiallergic, and smooth muscle relaxant effects, as well as shows antifungal, antimutagenic, and insecticidal activities (Borges et al., 2019, De Oliveira et al., 2019, Isman et al., 2008, Krzyżowski et al., 2020).

*Culex pipiens* is commonly known as house mosquito and is one of the most widely distributed mosquitoes worldwide and several studies reported the involvement of *Cx. pipiens* in transmission of the West Nile virus (Al-Mekhlafi et al., 2018, El-Akhal et al., 2015). A previous study suggested that control efforts focused on *Cx. pipiens* alone may greatly reduce both human exposure, infection, and epidemic distribution (Hamer et al., 2008). The research interest for botanical pesticides containing natural compounds as active ingredients, from which essential oils are considered a part. Where the use of these compounds attempts to avoid the development of pesticide resistance to conventional synthetic pesticides that are also now recognized as a threat to the diversity of ecosystems (Ahmed et al., 2021).

The present study aimed to evaluate the adulticidal and larvicidal potential of lavender, peppermint, and rosemary oils against the common house mosquito, *Cx. pipiens.* Furthermore, we aimed to determine the oils compounds by gas chromatography-mass spectrometry (GC–MS) analysis.

## 2 Material and methods

### 2.1 Mosquito colony rearing and experimental conditions

Mosquitoes (*Cx. pipiens*) were reared for several generations at the Center for Environmental Research and Studies at Jazan University. Rearing was performed under controlled conditions (27 ± 2 °C, relative humidity at 70% ± 10%, and 12:12 h light:dark regime). Adult mosquitoes were reared in wooden cages and supplied daily by 10% sucrose solution soaked in sponge pieces for 3–4 days post-emergence. Then, females were supplied pigeon blood meal. Plastic oviposition cups containing tap water (without chlorine) were placed in the cages. The resulting egg mass were transferred into plastic pans containing 3 L of tap water for 24 h. The hatching larvae were provided with a diet of fish food daily. The third instar *Cx. pipiens* larvae were used for the larvicidal examination, and the adults used for the adulticidal examination were glucose-fed female mosquitoes reared under the aforementioned controlled conditions.

### 2.2 Essential oils

Lavender essential oil, steam-distilled from flowering tops of *L. angustifolia*, (Lamiales: Lamiaceae; Case No. 8000-28-0), Peppermint essential oil, steam-distilled from the aerial parts of *M. piperita* L. (Lamiales: Lamiaceae; Case No. 8006-90-4), and Rosemary essential oil, steam-distilled from the tops of *R. officinalis* L. (Lamiales: Lamiaceae; Case No. 8000-25-7) were purchased from NOW Foods company (Natural Organic and Wholesome Foods), a distributor in Jeddah, Kingdom of Saudi Arabia.

### 2.3 Identification of the chemical composition using gas chromatography-mass spectrometer

The investigation of the chemical composition of the essential oils was performed by GC–MS using the Trace GC-TSQ mass spectrometer (Thermo Fisher Scientific, Waltham, MA, USA) with a direct capillary column, TG–5MS (30 m × 0.25 mm × 0.25 m thickness of the film). The operating conditions of the GC were column oven temperature initially maintained at 50 °C, then elevated at a rate of 5 °C/min up to 250 °C, maintained for 2 min, then elevated at a rate of 30 °C/min to 300 °C. The MS transfer line and the injector were adjusted at 270 °C and 260 °C, respectively, and helium was the carrier gas at the rate of 1 mL/min. The solvent retention time was 4 min, and 1 μL of the diluted samples was automatically injected using an AS1300 autosampler and GC split mode. In full scanning mode, electrospray ionization (EI) mass spectra were in the range of 50–650 m/s at an ionization voltage of 70 V. The temperature of the ion source was fixed at 200 °C. The mass spectra of the components were then compared with those in the mass spectral libraries from the NIST 14 and WILEY 09 databases, the selected constituents were identified from the Total Ion Chromatogram (TIC).

### 2.4 Larvicidal assay

The larvicidal activity was determined for the lavender, peppermint, and rosemary oils against *Cx. pipiens* third instar larvae according to guidelines of the World Health Organization (WHO, 2005). The solution of each oil was set as stock by mixing 1 mL oil with distilled water containing 0.2 mL Tween-20. Subsequently, five different concentrations were prepared, (62.5, 125, 250, 500, and 1000 ppm) based on v/v percent of 1% of stock solution. Twenty-five *Cx. pipiens* larvae were offered to each oil in 250 mL glass beakers containing 150 mL water at the aforementioned controlled conditions. Five replicates per concentration per oil and control were conducted. The larval mortalities were detected after 24 h for estimation of larval lethal concentration (LC_50_) by probit analysis.

### 2.5 Adulticidal assay

The adulticidal activity for the oils was analyzed by the adapted CDC bottle protocol (Ilahi et al., 2021, WHO, 2016). Five different concentrations from each oil dissolved in ethanol were prepared (0.5, 1, 2, 4, and 5%). Each prepared concentration for every oil was used to coat the CDC bottles (250 mL Wheaton bottles with screw lids) similarly to the control bottle, which was coated with only ethanol. The solvent was evaporated from the bottles for 1 h at 27 ± 2 °C. Three replicates per concentration per oil and the control were conducted. Adult glucose-fed female mosquitoes (n = 15) aged 3–4 d were selected using an aspirator and gently introduced to each bottle, and the bottles were closed with their lids. If a mosquito was knocked down or unable to move or stand within 60 min of exposure, it was considered as dead. The number was recorded for each bottle after 5, 10, 20, 30 and 60 min for determination of the median knockdown time (KT_50_) value and KT_90_ and KT_95_ values of each concentration through probit analysis. Live mosquitoes were then removed from bottles after 1 h and placed in separate paper cups containing 10% sucrose solution. Subsequently, the adult mortality rate was measured after 24 h within the replicates for determination of the sLC_50,_ LC_90_ and LC_95_ by probit analysis.

### 2.6 Data analysis

The percentage of mortalities were calculated according to Abbott’s formula (Abbott, 1925). The larval control results did not need correction, as the mortality was less than 5%, according to the WHO guidelines (WHO, 2005) (no larval control mortality was recorded throughout the study). The adult data were also not corrected, as the control adults had a mortality of less than 20% (WHO, 2016). Data from all the replicates were statistically analyzed to determine the larval and adult LC_50_, LC_50_, and LC_95_ values and the adult KT_50_, KT_90_, and KT_95_ values as well as chi-square values within confidence limits at 95% (lower confidence limit [LCL] and upper confidence limit [UCL]) by probit analysis using regression between log oil concentration and probit values. Mortality data were subjected to a one-way analysis of variance (ANOVA) (with a least significant difference (LSD) test). Data analysis was performed using SPSS software (IBM SPSS Statistics v22 – 64 bit), and p less than 0.05 was considered significant.

## 3 Results

### 3.1 Chemical analysis

The GC–MS analysis represented that the main compounds of the three essential oils were the monoterpenoids and sesquiterpenes (Table 4; Fig. 4). In lavender (*L. angustifolia*) essential oil, the main represented area% were monoterpenoids, linalool (23.75%), linalyl anthranilate (21.92%), lavandulyl acetate (11.85%), 4-terpineol (8.90%), and linalyl acetate (6.66%), followed by geranyl acetate (2.99%), L-α-terpineol (0.70%), and bornyl acetate (0.10%). The sesquiterpenoids were β-caryophyllene (16.35%), (E)-β-famesene (14.09%), germacrene D (1.29%), caryophyllene oxide (0.77%), γ-muurolene (0.35%), and transe-α-bergamotene (0.26%). In peppermint (*M. piperita* L.) oil, the main represented monoterpenoids were menthyl acetate (32.76%), L-menthol (26.41%), and L-menthone (13.57%), followed by pulegone (1.94%), L-alpha-terpineol (1.63%), and piperitone (1.32%). The main sesquiterpenoid was β-caryophyllene (18.48%), followed by humulene (1.41%), germacrene D (0.88%), β-bourbonene (0.58%), elemene isomer (0.46%), and alloaromadendrene (0.34%). In Rosemary (*R. officinalis* L.) essential oil, the chemical compounds were represented by camphor (56.55%) as the major monoterpenoid, followed by isoborneol (7.16%), α-terpineol (5.40%), and bornyl acetate (3.69%), and the main sesquiterpenoid was β-caryophyllene (23.0%), followed by humulene (3.20%), cadina-1(10),4-diene (0.39%), α-copaene (0.34%), and γ-muurolene (0.26%).

### 3.2 Larvicidal activity

**Fig. 1:**
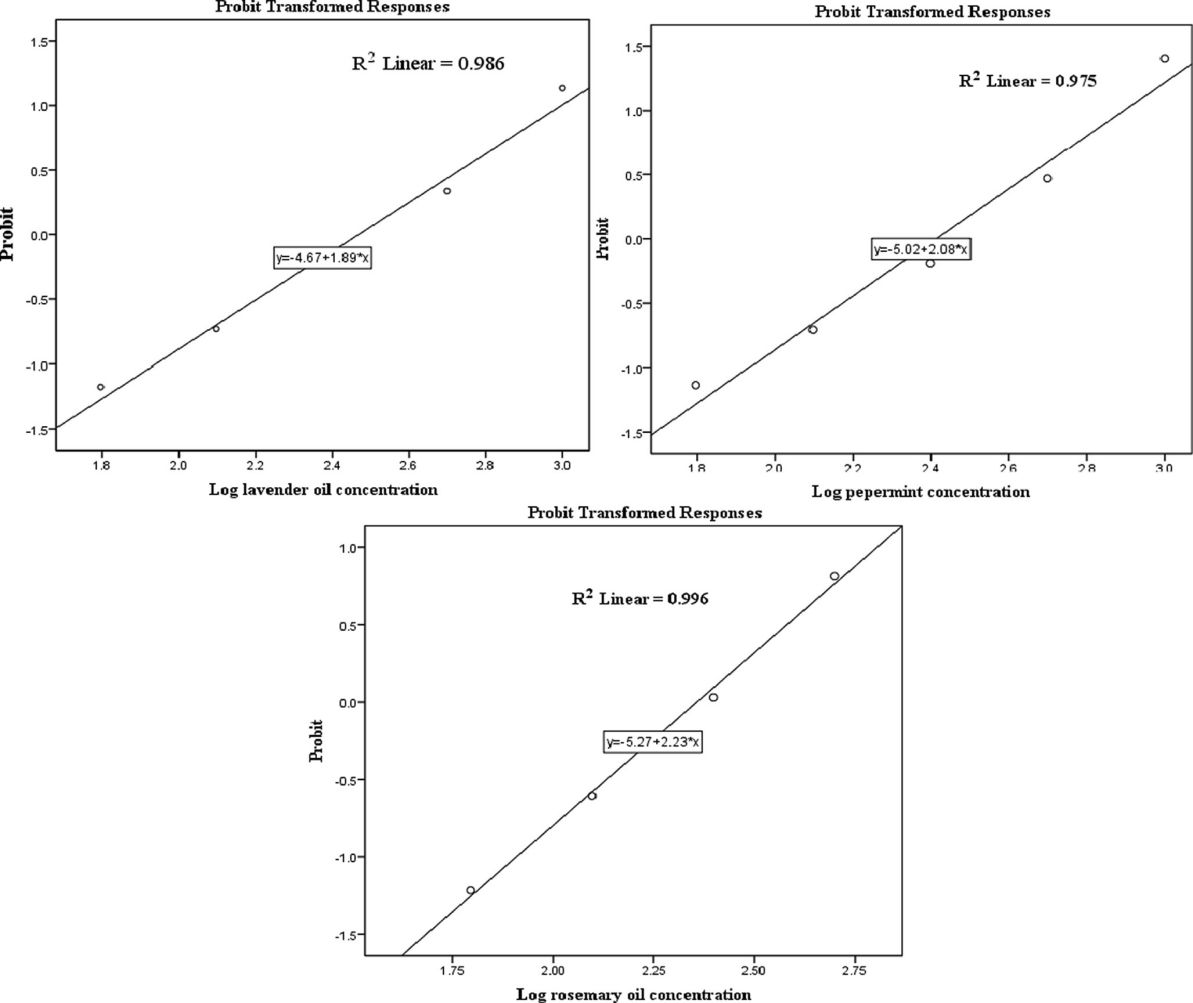
Probit regression responses of lavender, peppermint and rosemary essential oils against *Culex pipiens* larval mortality.

The data on the larvicidal activity of the tested essential oils against the third instar larvae of *Cx. pipiens* are represented in Table 1. The probit regression responses of the essential oils tested revealed that the larvicidal activities after 24 h ranged from 87.20 to 100% mortality at 1000 ppm. Rosemary essential oil showed the highest efficacy by inducing 100% mortality (LC_50_, 214.97, LC_90_, 671.77, and LC_95_, 927.90 ppm), followed by peppermint oil by 92.00% mortality (LC_50_, 269.35, LC_90_, 1137.74, and LC_95_, 1711.70 ppm). Lavender oil was the least effective, inducing 87.20% mortality (LC_50_, 301.11, LC_90_, 1437.63, and LC_95_, 2239.31 ppm) (Fig. 1).

**Table 1:** The larvicidal effects of essential oils against the third instar larvae of *Culex pipiens* at 24 h post-treatment.

Oil	Conc. ppm	Mortality% (Mean ± SE)	LC_50_ (LCL - UCL.)	LC_90_ (LCL - UCL.)	LC_95_ (LCL - UCL.)	Chi (Sig)
**Lavender** *Lavandula angustifolia*	0.0	0.00 ± 0.0^a^	301.11 (259.12-352.37)	1437.63 (1087.06-2096.14)	2239.31 (1600.19-3544.70)	2.175 (0.537^a^)
	62.5	12.00 ± 1.26^b^				
	125	23.20 ± 1.50^c^				
	250	40.80 ± 1.96^d^				
	500	63.20 ± 4.27^e^				
	1000	87.20 ± 3.44^f^				
**Peppermint** *Mentha piperita* L.	0.0	0.00 ± 0.0^a^	269.35 (233.37-311.12)	1137.74 (890.87-1571.50)	1711.70 (1276.39-2536.21)	4.148 (0.246^a^)
	62.5	12.80 ± 0.80^b^				
	125	24.00 ± 1.79^c^				
	250	42.40 ± 1.60^d^				
	500	68.00 ± 2.83^e^				
	1000	92.00 ± 1.79^f^				
**Rosemary** *Rosmarinus officinalis* L	0.0	0.00 ± 0.0^a^	214.97 (190.54-242.28)	671.77 (560.35-845.93)	927.90 (748.20-1226.12)	7.742 (0.052^a^)
	62.5	11.20 ± 1.50^b^				
	125	27.20 ± 1.50^c^				
	250	51.20 ± 2.94^d^				
	500	79.20 ± 0.80^e^				
	1000	100.00 ± 0.00^f^				

Significance at 0.05 level between different superscripts. (a) In Chi-Square Tests, no heterogeneity factor was used in the calculation of confidence limits because the significance level was greater than 0.050.

### 3.3 Adulticidal activity

**Fig. 2:**
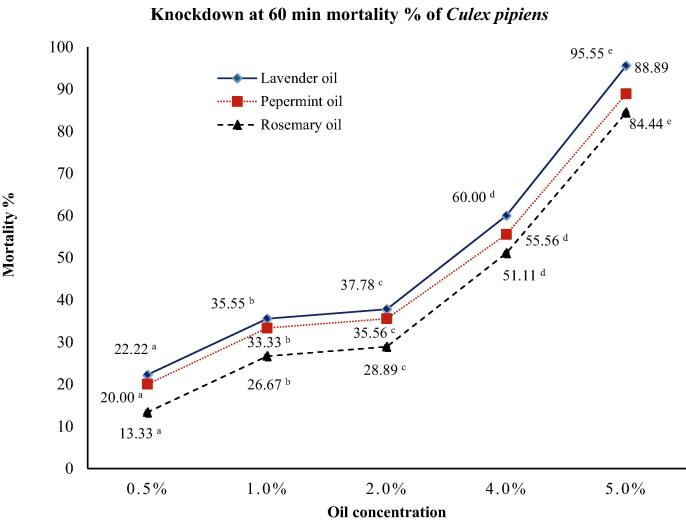
Knockdown rate (mortality %) of lavender, peppermint and rosemary essential oils against *Culex pipiens* female adults. Significant differences at 0.05 level between different superscript letters to means of the same oil.

The effect of the test concentrations of the three essentials oils on *Cx. pipiens* females were evaluated after 60 min of exposure in terms of LC_50,_ which is shown in Fig. 2, KT_50_, KT_90_, and KT_95_, which are listed in Table 2. According to the analysis, the highest knockdown rate was recorded for lavender oil (95.55%) followed by that for peppermint oil (88.89%) and lastly rosemary oil (84.44%) at the highest tested concentration of the oils (5%).

**Table 2:** Probit analysis of knockdown time and mortality rates of *Culex pipiens* females after oil exposure for 60 min.

Oil	Conc. (%)	KT_50_	KT_90_	KT_95_	R^2^	Equation	Chi (Sig)
**Lavender** *Lavandula angustifolia*	0.5	508.20	17769.11	48670.18	0.982	0.84*x-2.26	0.90 (0.962a)
	1.0	125.63	1559.97	3186.07	0.990	1.19*x-2.49	0.329(0.954a)
	2.0	122.96	2743.34	6615.54	0.996	0.96*x-2	0.111 (0.990a)
	4.0	47.70	441.56	829.79	0.960	1.29*x-2.18	2.759 (0.430a)
	5.0	17.43	57.51	80.68	0.958	2.57*x-3.13	6.900 (0.075a)
**Peppermint** *Mentha piperita* L.	0.5	871.38	48153.74	150169.81	0.973	0.75*x-2.18	0.330 (0.954a)
	1.0	152.41	2260.18	4854.49	0.980	1.12*x-2.43	0.657 (0.883a)
	2.0	152.49	4339.66	11212.58	0.995	0.89*x-1.94	0.108 (0.991a)
	4.0	70.59	633.83	1263.49	0.967	1.19*x-2.08	1.965 (0.580a)
	5.0	22.81	95.54	143.40	0.926	2.08*x-2.77	11.017 (0.012a)
**Rosemary** *Rosmarinus officinalis* L	0.5	1803.86	79,399	232145.78	0.957	0.77*x-2.53	0.495 (0.920a)
	1.0	256.06	6176.80	15228.61	0.971	0.9*x-2.19	0.617 (0.893a)
	2.0	186.45	1677.43	3126.90	0.990	1.28*x-2.96	0.360 (0.948a)
	4.0	77.40	772.27	1482.45	0.899	1.19*x-2.3	5.688 (0.128a)
	5.0	26.64	102.56	150.29	0.951	2.13*x-3.01	7.743 (0.52a)

Data represent the mean knockdown time. (a) In Chi-Square Tests, no heterogeneity factor was used in the calculation of confidence limits because the significance level was greater than 0.050.

When considering knockdown times, data revealed that at a concentration of 5%, the knockdown times were the lowest for lavender oil compared with those recorded for the other two oils. Lavender oil had a KT_50_ of 17.43 min, KT_90_ of 57.51 min, and KT_95_ of 80.68 min. Peppermint oil showed a KT_50_ of 22.81 min, KT_90_ of 95.54 min, and KT_95_ of 143.40 min. Furthermore, rosemary oil revealed a KT_50_ of 26.64 min, KT_90_ of 102.56 min, and KT_95_ of 150.29 min.

**Fig. 3:**
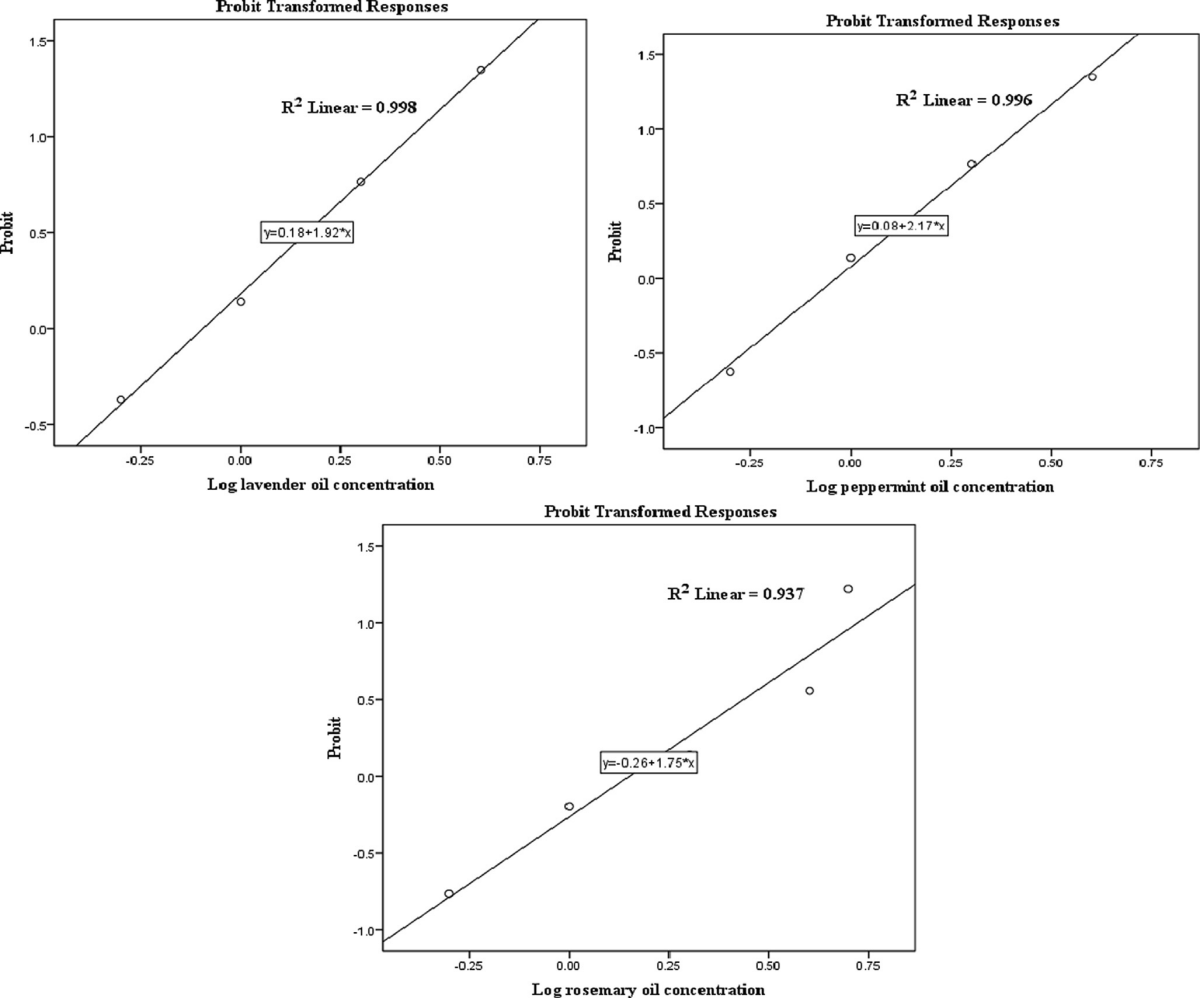
Probit regression responses of lavender, peppermint and rosemary essential oils against *Culex pipiens* adult mortality.

Adult toxicity of the three tested oils after 24 h was also assessed (Table 3), and probit regression results are shown in Fig. 3. Data showed contrary results to those observed for the larvicidal activity of the tested oils. Here, rosemary essential oil showed the least adulticidal efficiency (88.89% mortality rate) and an LC_50_ of 1.44% after 24 h at the highest tested concentration (5%). Lavender and peppermint oils at 5% concentration both showed a mortality rate of 100%, with an LC_50_ of 0.81% and 0.91%, respectively, after 24 h. It was noted that the lowest median adulticidal concentration was that of lavender oil which was in line with knockdown assay results.

**Table 3:** The adulticidal effects of essential oils against female adults of *Culex pipiens* at 24 h post-treatment.

Oil	Conc. %	Mortality% (Mean ± SE)	LC_50_ (LCL - UCL.)	LC_90_ (LCL - UCL.)	LC_95_ (LCL - UCL.)	Chi (Sig)
**Lavender** *Lavandula angustifolia*	0.0	2.47 ± 0.63^a^	0.81 (0.67-0.94)	3.09 (2.57-3.93)	4.52 (3.60-6.15)	6.642 (0.084^a^)
	0.5	35.56 ± 2.22^b^				
	1.0	55.56 ± 2.22^c^				
	2.0	77.78 ± 2.22^d^				
	4.0	91.11 ± 2.22^e^				
	5.0	100.00 ± 0.00^f^				
**Peppermint** *Mentha piperita* L.	0.0	2.47 ± 0.63^a^	0.91 (0.78-1.04)	3.02 (2.55-3.74)	4.24 (3.46-5.55)	5.891 (0.117^a^)
	0.5	26.67 ± 3.85^b^				
	1.0	55.56 ± 2.22^c^				
	2.0	77.78 ± 2.22^d^				
	4.0	91.11 ± 2.22^e^				
	5.0	100.00 ± 0.00^f^				
**Rosemary** *Rosmarinus officinalis* L	0.0	2.47 ± 0.63^a^	1.44 (1.20-1.69)	8.24 (6.14-12.51)	13.52 (9.38-22.95)	6.449 (0.092^a^)
	0.5	22.22 ± 2.22^b^				
	1.0	42.22 ± 2.22^c^				
	2.0	55.56 ± 2.22^d^				
	4.0	71.11 ± 2.22^e^				
	5.0	88.89 ± 5.88^f^				

(a) In Chi-Square Tests, no heterogeneity factor was used in the calculation of confidence limits because the significance level was greater than 0.050.

**Table 4:** Chemical constituents of lavender, peppermint, and rosemary essential oils.

Oil	Molecular formula	Chemical name, (synonym)	Area (%)	RT	Nature of compound
Lavender (*Lavandula angustifolia*)	C_10_H_18_O	**Linalool**, 3,7-Dimethylocta-1,6-dien-3-ol	23.75	4.26 4.50	Monoterpenoid
	C_10_H_18_O	**4-terpineol**,3-Cyclohexen-1-ol, 4-methyl-1-(1-methylethyl)-, (R) -	8.90	5.19 5.52	Monoterpenoid
	C_10_H_18_O	**L-alpha-Terpineol**,3-Cyclohexene-1-methanol, à,à,4-trimethyl-,(S) -	0.70	5.89	Monoterpene alcohol
	C_12_H_20_O_2_	**Linalyl acetate**, 1,6-Octadien-3-ol, 3,7-dimethyl-, acetate	6.66	6.88 7.12	Acyclic monoterpenoid
	C_17_H2_23_NO_2_	**Linalyl anthranilate**, 1,5-Dimethyl-1-vinyl-4-hexen-1-yl o-aminobenzoate,	21.92	7.33	Aromatic monoterpenoid
	C_12_H_20_O_2_	**Bornyl acetate**, Bicyclo[2.2.1]heptan-2-ol, 1,7,7-trimethyl-, acetate, endo-	0.10	7.66	Bicyclic monoterpenoid
	C_12_H_20_O_2_	**Lavandulyl acetate**, 4-Hexen-1-ol,5-methyl-2-(1-methylethenyl) -, Acetate	11.85	7.89	Monoterpenoid Acetic acid lavandulylester
	C_12_H_20_O_2_	**Geranyl acetate**, 2,6-Octadien-1-ol, 3,7-dimethyl-,acetate, (Z) -	2.99	9.48 9.97	Monoterpenoid
	C_15_H_24_	β**-Caryophyllene**, Bicyclo[7.2.0]undec-4-ene, 4,11,11-trimethyl-8-methylene-,[1R-(1R*,4Z,9S*)]-	16.35	10.63	Sesquiterpenoid
	C_15_H_24_	**transe-Alpha-Bergamotene,** Bicyclo[3.1.1]hept-2-ene,2,6-dimethyl-6-(4-methyl-3-pentenyl) -	0.26	10.95	Sesquiterpenoid
	C_15_H_24_	**(E)-beta-Famesene**, 1,6,10-Dodecatriene, 7,11-dimethyl-3-methylene-, (E)-	14.09	11.63	Sesquiterpenoid
	C_15_H_24_	**Germacrene D**, (S,1Z,6Z)-8-Isopropyl-1-methyl-5-methylenecyclodeca-1,6-diene	1.29	12.01	Sesquiterpenoid
	C_15_H_24_	γ**-Muurolene**, Naphthalene, 1,2,3,4,4a,5,6,8a-octahydro-7-methyl-4-methylene-1-(1-methylethyl)-, (1α,4aβ,8aα)-	0.35	12.76	Sesquiterpenoid
	C_15_H_24_O	**Caryophyllene oxide**, 5-Oxatricyclo[8.2.0.0(4,6)-]dodecane, 4,12,12-trimethyl-9-methylene-, [1R-(1R*,4R*,6R*,10S*)]-	0.77	14.33	Sesquiterpenoid
Peppermint (*Mentha piperita* L.)	C_10_H_18_O	**L-Menthone,** 2-Isopropyl-5-methylcyclohexanone	13.57	4.68 4.88 5.19	Monoterpenoid
	C_10_H_20_O	**L-Menthol**,Cyclohexanol, 5-methyl-2-(1-methylethyl)-, [1R-(1à,2á,5à) ]-	26.41	6.01	Monoterpenoid
	C_10_H_18_O	**L-alpha-Terpineol**,3-Cyclohexene-1-methanol, à,à,4-trimethyl-, (S) -	1.63	6.09	Monoterpenoid
	C_10_H_16_O	**Pulegone**, Cyclohexanone,5-methyl-2-(1-methylethylidene) -	1.94	6.80	Monoterpenoid
	C_10_H_16_O	**Piperitone**, 6-isopropyl-3-methylcyclohex-2-enone	1.32	7.13	Monoterpenoid
	C_12_H_22_O_2_	**Menthyl acetate**, Cyclohexanol, 5-methyl-2-(1-methylethyl)-, acetate, (1à,2á,5à)-	32.76	7.98	Monoterpenoid
	C_15_H_24_	**Elemene isomer**, Cyclohexane,1-ethenyl-1-methyl-2-(1-methylethenyl)-4-(1-methylethylidene) -	0.46	8.67	Sesquiterpenoid
	C_15_H_24_	**Beta-Bourbonene**,Cyclobuta(1,2:3,4) dicyclopentene, 1,2,3,3a,3bbeta,4,5,6,6abeta,6balpha-decahydro-1alpha-isopropyl-3aalpha-methyl-6-methylene	0.58	9.73	Sesquiterpenoid
	C_15_H_24_	β**-Caryophyllene**, Bicyclo[7.2.0]undec-4-ene, 4,11,11-trimethyl-8-methylene-,[1R-(1R*,4Z,9S*)]-	18.48	10.62	Sesquiterpenoid
	C_15_H_24_	**Humulene**,1,4,8-Cycloundecatriene, 2,6,6,9-tetramethyl-, (E,E,E) -	1.41	11.32	Sesquiterpenoid
	C_15_H_24_	**Germacrene D**,1-Methyl-5-methylene-8-(1-methylethyl) -1,6-cyclodecadiene	0.88	11.97	Sesquiterpenoid
	C_15_H_24_	**Alloaromadendrene**	0.34	12.33	Sesquiterpenoid
Rosemary (*Rosmarinus officinalis* L)	C_10_H_16_O	**Camphor,** Bicyclo[2.2.1]heptan-2-one, 1,7,7-trimethyl-, (1R)-	56.55	4.74 5.02	Bicyclic monoterpenoid
	C_10_H_18_O	**Isoborneol**, Bicyclo[2.2.1]heptan-2-ol, 1,7,7-trimethyl-, exo-	7.16	5.27	Bicyclic monoterpenoid
	C_10_H_18_O	**alpha-Terpineol**,3-Cyclohexene-1-methanol, à,à,4-trimethyl-, (S) -	5.40	5.79	Monoterpene alcohol
	C_12_H_20_O_2_	**Bornyl acetate**, Bicyclo[2.2.1]heptan-2-ol, 1,7,7-trimethyl-, acetate, (1S-endo)-	3.69	7.57	Bicyclic monoterpenoid
	C_15_H_24_	**alpha-Copaene**,Tricyclo[4.4.0.0(2,7)]dec-3-ene, 1,3-dimethyl-8-(1-methylethyl) -, st	0.34	9.50	Sesquiterpenes
	C_15_H_24_	**β-Caryophyllene**, Bicyclo[7.2.0]undec-4-ene, 4,11,11-trimethyl-8-methylene-, [1R-(1R*,4E,9S*)]-	23.00	10.60	Sesquiterpenes
	C_15_H_24_	**Humulene, alpha-Caryophyllene**, 1,4,8-Cycloundecatriene, 2,6,6,9-tetramethyl-, (E,E,E)-	3.20	11.32	Sesquiterpenes
	C_15_H_24_	**γ-Muurolene**, Naphthalene, 1,2,3,4,4a,5,6,8a-octahydro-7-methyl-4-methylene-1-(1-methylethyl)-, (1α,4aβ,8aα)-	0.26	11.88	Sesquiterpenes
	C_15_H_24_	**Cadina-1(10),4-diene, delta-Cadinene,** Naphthalene, 1,2,3,5,6,8a-hexahydro-4,7-dimethyl-1-(1-methylethyl)-, (1S-cis)-	0.39	12.98	Sesquiterpenes

**Fig. 4:**
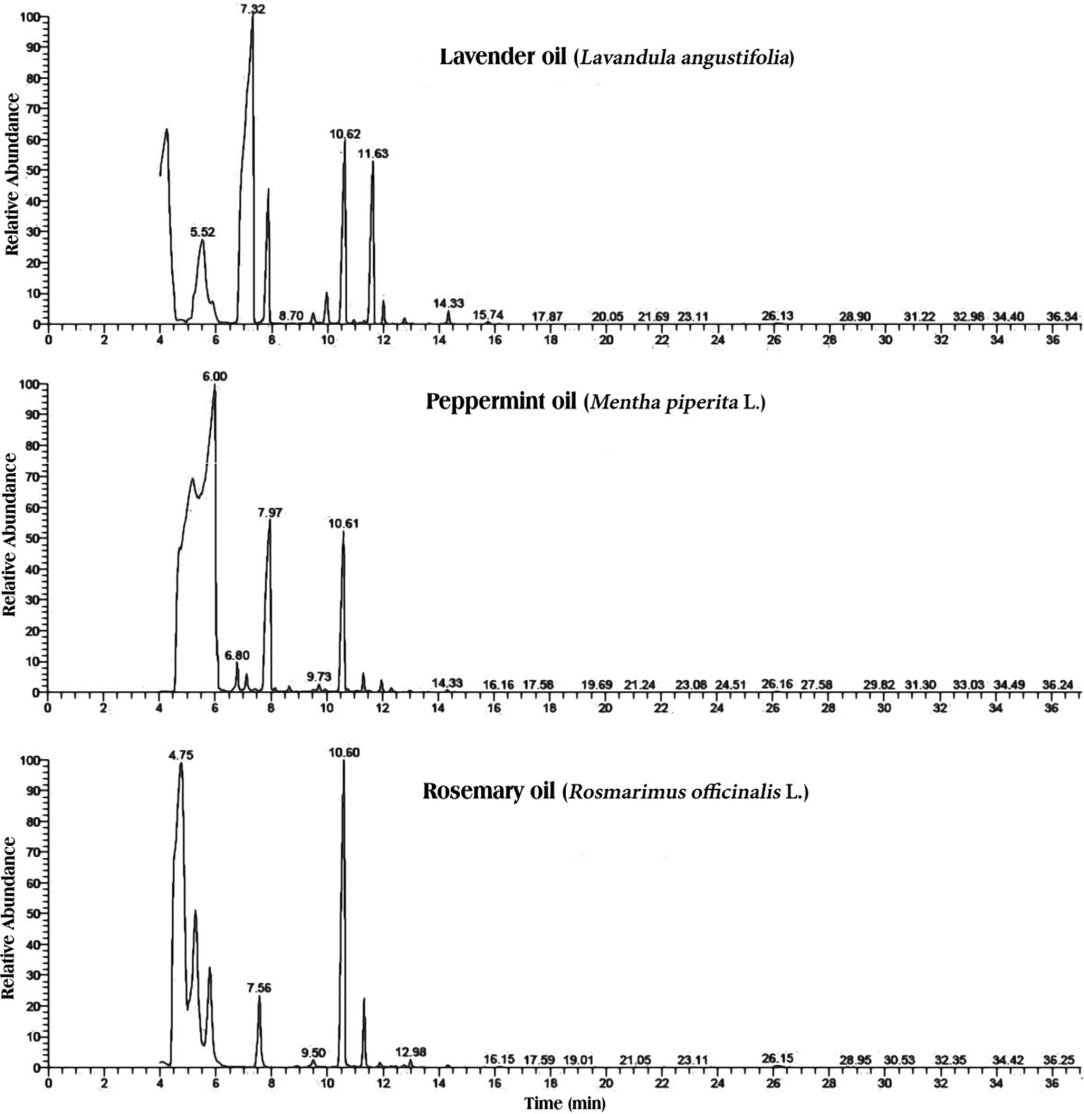
The TIC chromatograms of lavender, peppermint and rosemary essential oils chemical constituents detected by GC–MS.

## 4 Discussion

The present study tested the larvicidal and adulticidal efficacy of lavender, peppermint, and rosemary essential oils against *Cx. pipiens* mosquitos. These effects were expected owing to the active constituents of these oils. The major components present in lavender essential oil are as determined by GC–MS in this study corresponded with the findings of previous studies (El-Akhal et al., 2021, Karamaouna et al., 2013), as did the components present in the peppermint essential oil (Ebadollahi et al., 2017, Mackled et al., 2019, Samarasekera et al., 2008) and the rosemary essential oil (Jafari-sales and Pashazadeh, 2020, Zeghib et al., 2020). Generally, the tested three essential oils recorded chemical constituents and their insecticidal activities in accordance with the methods of several previous studies reviewed by Ebadollahi et al (2020).

The study was coverage the larvicidal activity of the tested oils against the third instar larvae of *Cx. pipiens.* The study evaluated the concentration-related mortalities for each of the oils, and graded the mortalities at the highest used concentration (1000 ppm), where rosemary oil acquired the most potent larvicidal activity (100% mortality) followed by peppermint oil (92%) and lastly lavender oil (87%).

In line with the present results about larvicidal LC_50_ value, Radwan et al. (2008), recorded an LC_50_ of 216.10 ppm for rosemary essential oil and lower larvicidal efficacy of peppermint oil (>500 ppm) against *Cx. pipiens*. The study presented that the saturated monoterpenoid alcohol menthol, which is the major component in peppermint oil, exhibited lower larvicidal activity against *Cx. pipiens*. Zeghib et al. (2020) evaluated larvicidal activity against the fourth instar larvae of *Cx. pipiens* of rosemary essential oil extracted from the aerial parts of *R. officinalis.* They recorded 100% mortality at 99.34 ppm of the oil concentration and evaluated camphor as one of the major components. A further component that had a role in toxicity was β-caryophyllene, which was the second most abundant component detected in rosemary oil (23%) and the third most abundant in lavender (16.35%) and peppermint oils (18.48%) and displayed effective larvicidal activity as an individual compound against *Anopheles subpictus, Aedes albopictus*, and *Cx. tritaeniorhynchus* (Govindarajan et al., 2016). A sesquiterpene compound, α-humulene, was present in a higher percentage in rosemary oil (3.20%) than in peppermint oil (1.41%) and not detected in lavender oil. This compound exhibited larvicidal activity at low dosages against *A. subpictus, Ae. albopictus,* and *Cx. tritaeniorhynchus* third instar larvae (Govindarajan and Benelli, 2016). Linalool, the main constituent in lavender oil, had larvicidal activity against third instar larvae of *Ae. aegypti*, and morphological alterations detected in larvae exposed to sublethal doses of linalool included abdomen elongation and curving and gut content partial extrusion involving the peritrophic matrix (Fujiwara et al., 2017). The essential oil extracted from *M. piperita* leaves exhibited larvicidal and repellent efficacy against the early fourth instar larvae of *Ae. aegypti* (Kumar et al., 2011). Another study showed the toxicity effects of peppermint and lavender essential oils against *M. domestica* larvae in conjunction with larval morphological abnormalities (Bosly, 2013). Moreover, studies reported that minor compounds found such as bornyl acetate (present in rosemary oil) exhibited strong larvicidal activity against *Cx. pipiens* larvae (Zeghib et al., 2020). Other compounds present in high concentrations, similarly to our study, such as linalool and terpineol (in lavender oil) and menthone (in peppermint oil) individually exhibited low larvicidal activities with recorded LD_50_ of 193,194, and 156 mg/L, respectively, against *Cx. pipiens* larvae (Radwan et al., 2008, Traboulsi et al., 2002).

Contrary to the larvicidal results obtained in this study, lavender, peppermint, and rosemary essential oils at 1000 ppm concentration resulted in larval mortality of 68, 100 and 80%, respectively, after 24 h against *Cx. quinquefasciatus*, revealing the potent larvicidal effect of peppermint oil (Manimaran et al., 2012). El-Akhal et al (2021) showed that lavender oil at 800 ppm resulted in 100% larval mortality with an LC_50_ of 140 ppm against *Cx. pipiens* larvae. Zhu and Tian (2013) tested the larvicidal activities of camphor, eucalyptol, terpine-4-ol, germacrene D, caryophyllene oxide, and caryophyllene against *A. anthropophagus* and found that the most potent were caryophyllene oxide and germacrene D, followed by terpine-4-ol and camphor, and the least potent was caryophyllene. In addition, Dias and Moraes (2014) reported that the majority of essential oils showed potent larvicidal activity derived from species of particular families, such as Laminaceae that are rich in sesquiterpenes and monoterpene hydrocarbons, and their individual pure compounds showed against mosquito larvae high activity.

Herein, the tested plant oils are not toxic to vertebrates and are identified to be eco-friendly as well as used as medicinal plants. However, their varied related toxicity potential with insect stage. Rosemary oil displayed the strongest larvicidal activity, while lavender and peppermint oils exhibited the strongest adulticidal activity. The adulticidal and knockdown activities of the tested oils recorded 100% mortalities for lavender and peppermint oils with KT_50_ of 17.43 and 22.81 min, respectively, while rosemary oil showed only 88.89% mortality with a KT_50_ of 26.64 min.

Structural variations of peppermint constituents, menthol, menthyl acetate, menthone, α-terpineol, pulegone, and β-caryophyllene, which were previously shown to be adulticidal and have knockdown efficacy against 3-day-old females of *Cx. Quinquefasciatus*, could contribute towards retaining or enhancing the oil mosquitocidal activity (Samarasekera et al., 2008). Pulegone was the most effective adulticidal among different tested terpenes with LC_50_ values lower than 0.1 mg/L against *Ae. aegypti* adults (Flores Guillermo et al., 2020). Linalool had an LC_50_ value of 14.87 μg/mL against adult *Cx. pipiens* mosquitoes (Tabari et al., 2017). Additionally, the repellent properties of peppermint essential oil were established against adults *Ae. aegypti* (Kumar et al., 2011, Manh and Tuyet, 2020). The effective repellent activity was also evaluated for lavender and rosemary essential oils against adult mosquitoes of *Cx. pipiens pallens* (Choi et al., 2002).

The toxicity of rosemary essential oil towards adults of *Cx. pipiens* was previously evaluated and proposed be attributed to its major monoterpenes constituents and recorded strong fumigant toxicity against adults, meanwhile, showed weak toxicity against larvae (Zahran et al., 2017).

The study confirmed the oil constituent’s role in toxicity, where interaction between the oil constituents is very important for the development of its insecticidal formulation and adjustment of the content active substances for formulation to ensure the biological efficacy as previously confirmed (Pavela, 2015, Sharma et al., 2022).

## 5 Conclusion

The present study reviled that rosemary oil acquired the most potent larvicidal activity (100% mortality) followed by peppermint oil (92%) and lastly lavender oil (87%). The adulticidal and knockdown activities of the tested oils recorded 100% mortalities for lavender and peppermint oils with KT_50_ of 17.43 and 22.81 min, respectively, while rosemary oil showed only 88.89% mortality with a KT_50_ of 26.64 min. The present study suggests that the synergistic effect of compounds present in each essential oil may elevate its biological activity against the target insect stage and proposed rosemary essential oil may be useful for control of *Cx. pipiens* larvae as a water treatment product and lavender and peppermint for application in control of adult mosquitoes.

## Funding

None.

## Ethics approval and consent to participate

Not applicable.

## Declaration of Competing Interest

The authors declare that they have no known competing financial interests or personal relationships that could have appeared to influence the work reported in this paper.
