# Supplementary material for: Larvicidal and adulticidal activity of essential oils from plants of the Lamiaceae family against the West Nile virus vector, Culex pipiens (Diptera: Culicidae)
Source: Saudi J Biol Sci. 2022 Jun 16;29(8):103350. doi: 10.1016/j.sjbs.2022.103350 (PMC9232543; doi:10.1016/j.sjbs.2022.103350)

# My GC-MS Report

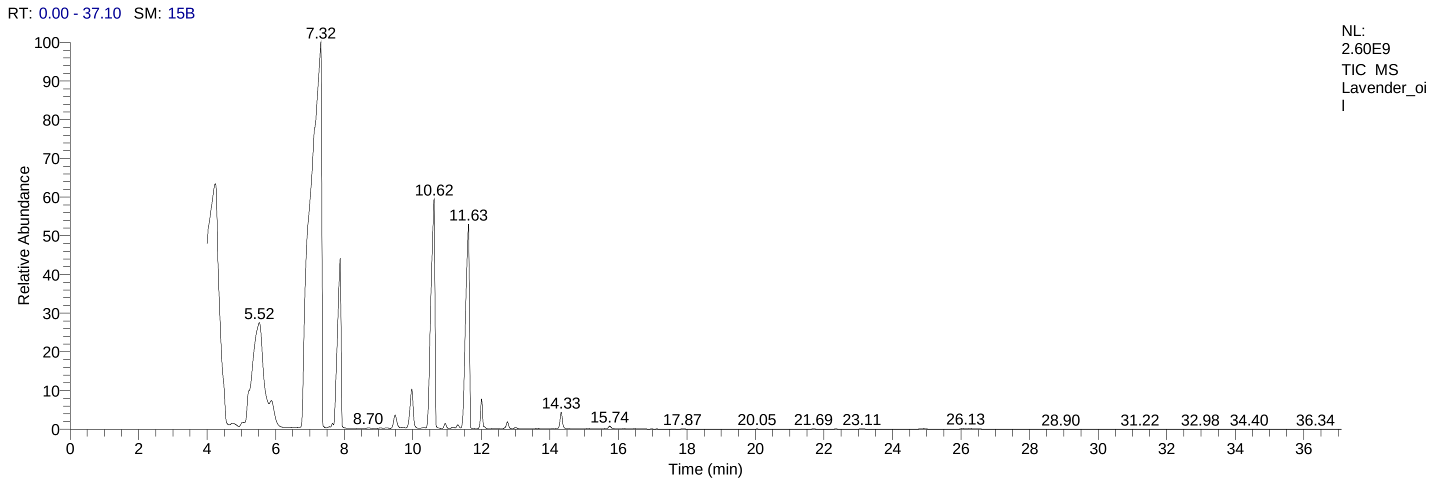


| RT | Area % | Peak Area | Peak Height |
|---:|---:|---:|---:|
| 4.26 | 13.71 | 8336492900.63 | 697429860.56 |
| 4.50 | 0.25 | 154909057.07 | 57642903.37 |
| 5.19 | 0.46 | 280772178.21 | 78836482.86 |
| 5.52 | 8.24 | 5007377838.79 | 376205403.32 |
| 5.89 | 0.70 | 425340162.10 | 66014869.38 |
| 6.88 | 6.10 | 3710435713.27 | 397416709.28 |
| 7.12 | 0.56 | 341823470.06 | 96569564.69 |
| 7.33 | 21.92 | 13329674483.47 | 2117722417.52 |
| 7.66 | 0.10 | 58279099.53 | 26430609.79 |
| 7.89 | 11.85 | 7203733810.27 | 1121851949.68 |
| 9.48 | 0.75 | 457341384.15 | 82160639.64 |
| 9.97 | 2.24 | 1359985919.45 | 250072852.48 |
| 10.63 | 16.35 | 9937617862.18 | 1489085704.51 |
| 10.95 | 0.26 | 159212634.14 | 37796866.06 |
| 11.63 | 14.09 | 8567015622.13 | 1333205117.22 |
| 12.01 | 1.29 | 786098696.69 | 232459266.03 |
| 12.76 | 0.35 | 210505368.86 | 48877491.37 |
| 14.33 | 0.77 | 470823529.81 | 118358748.34 |

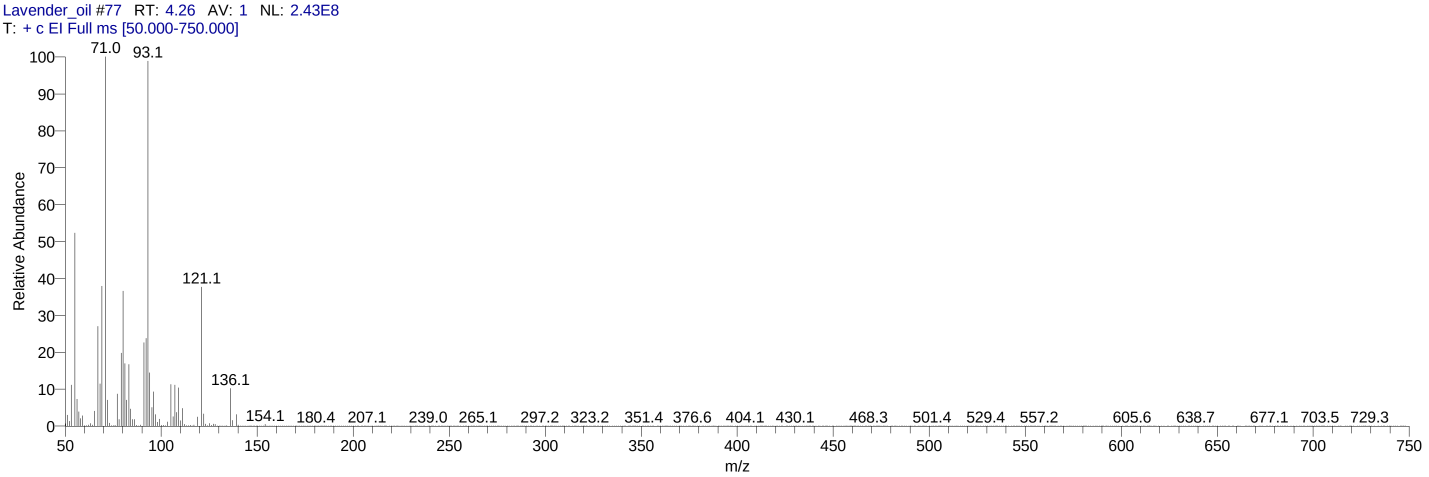


| RT | Compound Name | Area % | MF | Molecular Formula | Molecular Weight | Cas # | Library |
|---:|---:|---:|---:|---:|---:|---:|---:|
| 4.26 | 1,6-OCTADIEN-3-OL, 3,7-DIMETHYL- | 13.71 | 942 | C10H18O | 154 | 78-70-6 | WileyRegistry8e |

# My GC-MS Report

| RT | Compound Name | Area % | MF | Molecular Formula | Molecular Weight | Cas # | Library |
|---|---|---|---|---|---|---|---|
| 4.26 | 1,6-OCTADIEN-3-OL, 3,7-DIMETHYL- | 13.71 | 940 | C10H18O | 154 | 78-70-6 | WileyRegistry8e |
| 4.26 | 1,6-OCTADIEN-3-OL, 3,7-DIMETHYL- | 13.71 | 933 | C10H18O | 154 | 78-70-6 | WileyRegistry8e |
| 4.26 | Linalool | 13.71 | 925 | C10H18O | 154 | 78-70-6 | mainlib |
| 4.26 | 1,6-OCTADIEN-3-OL, 3,7-DIMETHYL- | 13.71 | 921 | C10H18O | 154 | 78-70-6 | WileyRegistry8e |

| Compound Structure | Hit Spectrum |
|---|---|

1,6-OCTADIEN-3-OL, 3,7-DIMETHYL-
Formula C10H18O, MW 154, CAS# 78-70-6, Entry# 38740
3,7-DIMETHYLOCTA-1,6-DIEN-3-OL

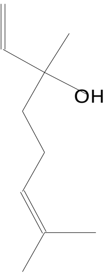


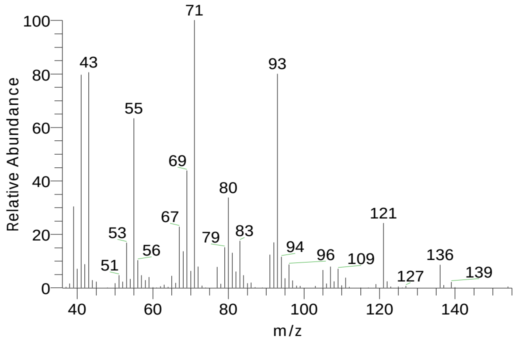


1,6-OCTADIEN-3-OL, 3,7-DIMETHYL-
Formula C10H18O, MW 154, CAS# 78-70-6, Entry# 38753
3,7-DIMETHYLOCTA-1,6-DIEN-3-OL

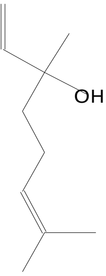


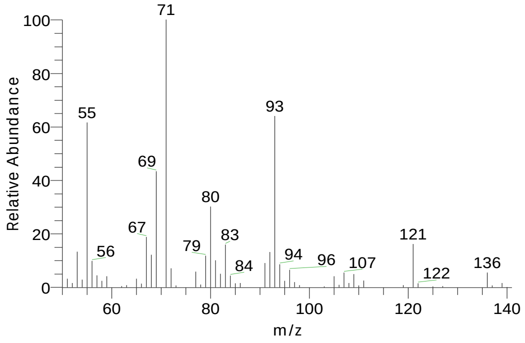


1,6-OCTADIEN-3-OL, 3,7-DIMETHYL-
Formula C10H18O, MW 154, CAS# 78-70-6, Entry# 38714
3,7-DIMETHYLOCTA-1,6-DIEN-3-OL

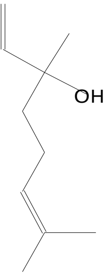


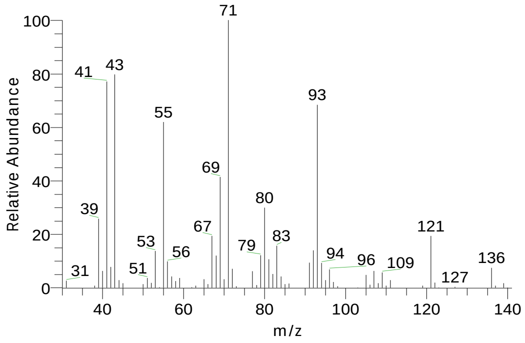


Linalool
Formula C10H18O, MW 154, CAS# 78-70-6, Entry# 39161
1,6-Octadien-3-ol, 3,7-dimethyl-

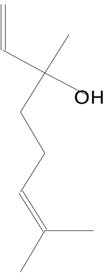


SI 924, RSI 925, mainlib, Entry# 39161, CAS# 78-70-6, Linalool

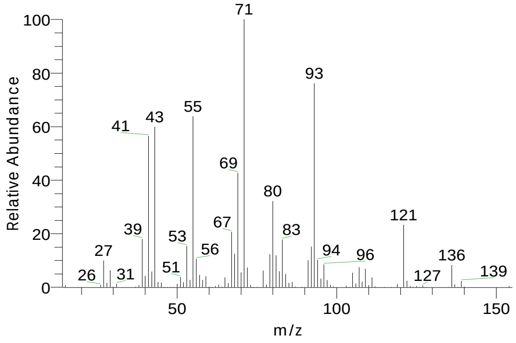

# *My GC-MS Report*

| Compound Structure | Hit Spectrum |
|---|---|

1,6-OCTADIEN-3-OL, 3,7-DIMETHYL-
Formula C10H18O, MW 154, CAS# 78-70-6, Entry# 358563
3,7-DIMETHYLOCTA-1,6-DIEN-3-OL

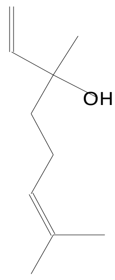


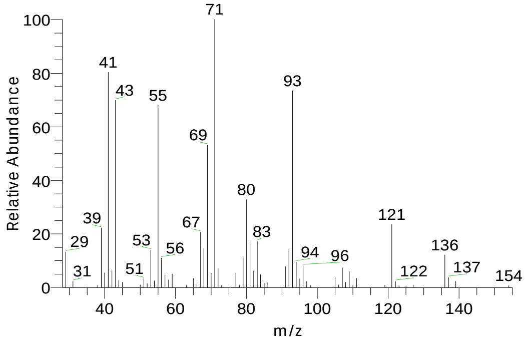


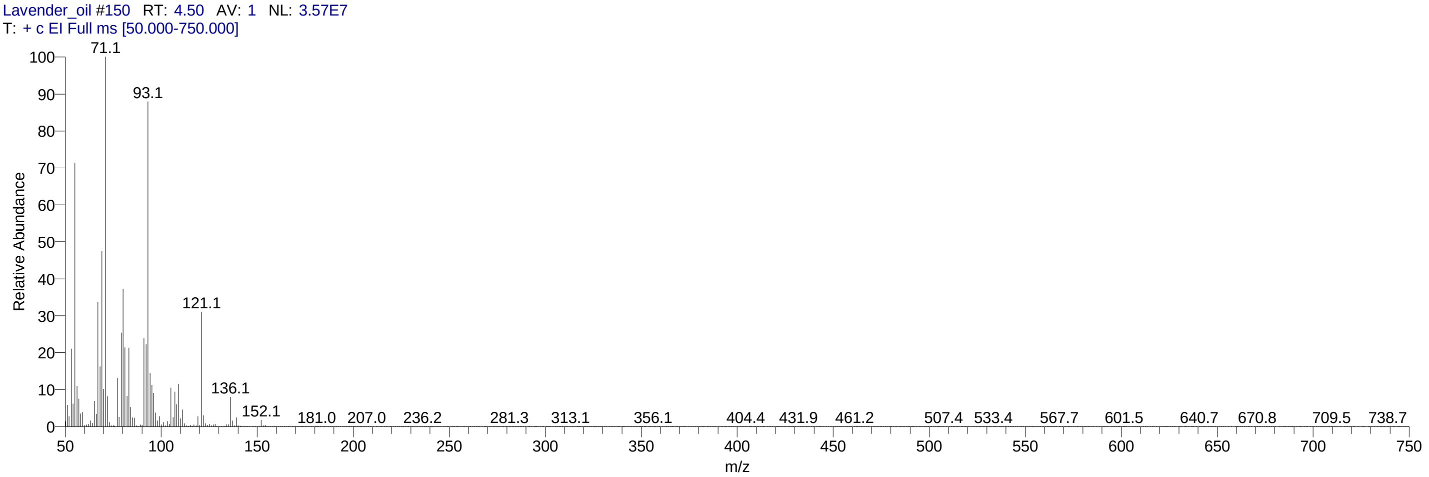


| RT | Compound Name | Area % | MF | Molecular Formula | Molecular Weight | Cas # | Library |
|---|---|---|---|---|---|---|---|
| 4.50 | 1,6-OCTADIEN-3-OL, 3,7-DIMETHYL- | 0.25 | 929 | C10H18O | 154 | 78-70-6 | WileyRegistry8e |
| 4.50 | 1,6-OCTADIEN-3-OL, 3,7-DIMETHYL- | 0.25 | 913 | C10H18O | 154 | 78-70-6 | WileyRegistry8e |
| 4.50 | 1,6-OCTADIEN-3-OL, 3,7-DIMETHYL- | 0.25 | 908 | C10H18O | 154 | 78-70-6 | WileyRegistry8e |
| 4.50 | 1,6-OCTADIEN-3-OL, 3,7-DIMETHYL- | 0.25 | 905 | C10H18O | 154 | 78-70-6 | WileyRegistry8e |
| 4.50 | 1,6-OCTADIEN-3-OL, 3,7-DIMETHYL- | 0.25 | 912 | C10H18O | 154 | 78-70-6 | WileyRegistry8e |

| Compound Structure | Hit Spectrum |
|---|---|

1,6-OCTADIEN-3-OL, 3,7-DIMETHYL-
Formula C10H18O, MW 154, CAS# 78-70-6, Entry# 38242
3,7-DIMETHYLOCTA-1,6-DIEN-3-OL

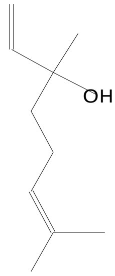


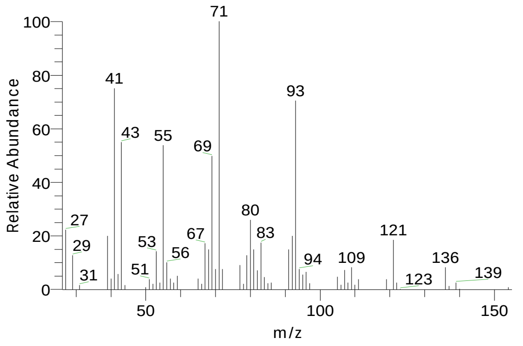

# *My GC-MS Report*

Compound Structure | Hit Spectrum

1,6-OCTADIEN-3-OL, 3,7-DIMETHYL-
Formula C10H18O, MW 154, CAS# 78-70-6, Entry# 38740
3,7-DIMETHYLOCTA-1,6-DIEN-3-OL

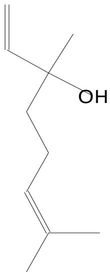


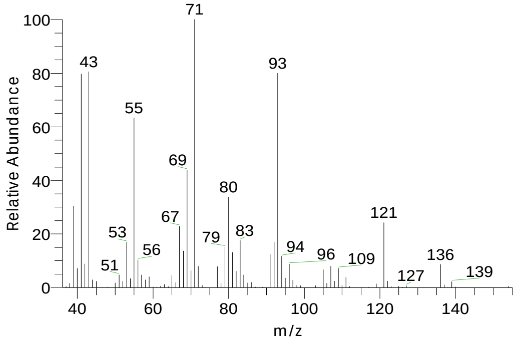


1,6-OCTADIEN-3-OL, 3,7-DIMETHYL-
Formula C10H18O, MW 154, CAS# 78-70-6, Entry# 358563
3,7-DIMETHYLOCTA-1,6-DIEN-3-OL

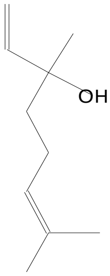


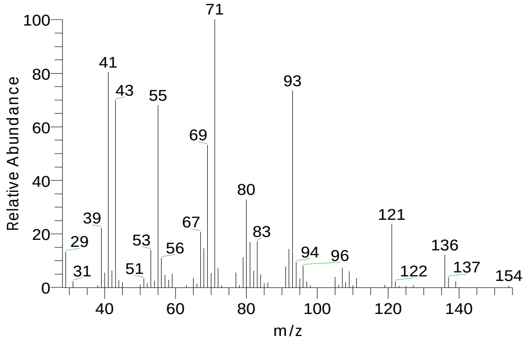


1,6-OCTADIEN-3-OL, 3,7-DIMETHYL-
Formula C10H18O, MW 154, CAS# 78-70-6, Entry# 38239
3,7-DIMETHYLOCTA-1,6-DIEN-3-OL

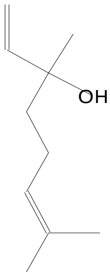


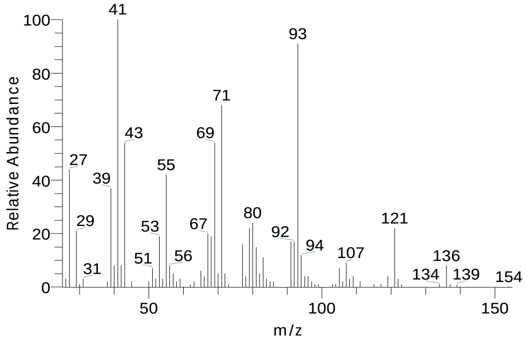


1,6-OCTADIEN-3-OL, 3,7-DIMETHYL-
Formula C10H18O, MW 154, CAS# 78-70-6, Entry# 38753
3,7-DIMETHYLOCTA-1,6-DIEN-3-OL

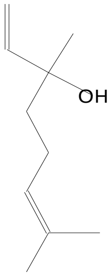


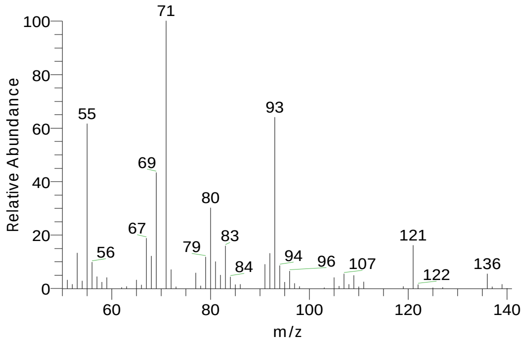

# *My GC-MS Report*

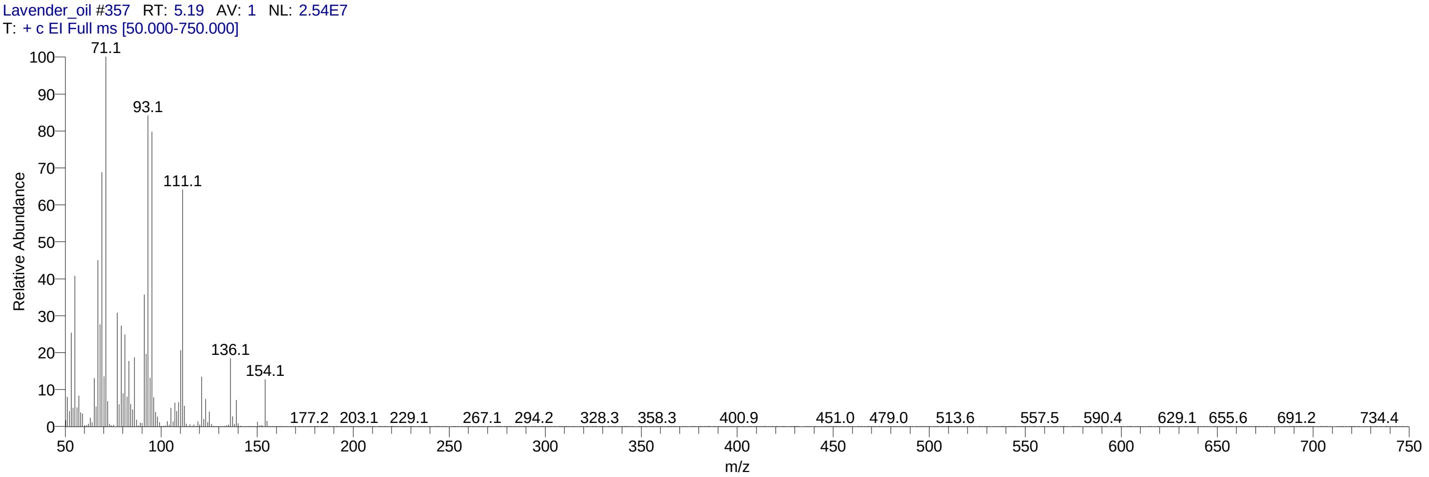


| RT | Compound Name | Area % | MF | Molecular Formula | Molecular Weight | Cas # | Library |
|---|---|---|---|---|---|---|---|
| 5.19 | 3-CYCLOHEXEN-1-OL, 4-METHYL-1-(1-METHYLETHYL)- | 0.46 | 874 | C10H18O | 154 | 562-74-3 | WileyRegistry8e |
| 5.19 | 3-CYCLOHEXEN-1-OL, 4-METHYL-1-(1-METHYLETHYL)- | 0.46 | 871 | C10H18O | 154 | 562-74-3 | WileyRegistry8e |
| 5.19 | 3-CYCLOHEXEN-1-OL, 4-METHYL-1-(1-METHYLETHYL)- | 0.46 | 869 | C10H18O | 154 | 562-74-3 | WileyRegistry8e |
| 5.19 | Terpinen-4-ol | 0.46 | 858 | C10H18O | 154 | 562-74-3 | replib |
| 5.19 | 1-ISOPROPYL-4-METHYL-3-CYCLOHEXEN-1-OL | 0.46 | 858 | C10H18O | 154 | NA | WileyRegistry8e |

Compound Structure | Hit Spectrum

3-CYCLOHEXEN-1-OL, 4-METHYL-1-(1-METHYLETHYL)-
Formula C10H18O, MW 154, CAS# 562-74-3, Entry# 38345
1-ISOPROPYL-4-METHYL-3-CYCLOHEXEN-1-OL #

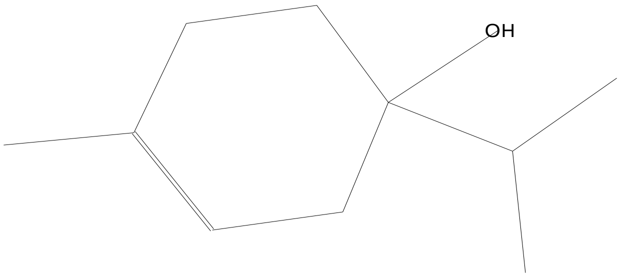


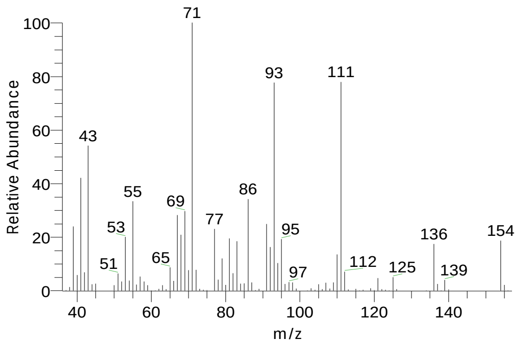


3-CYCLOHEXEN-1-OL, 4-METHYL-1-(1-METHYLETHYL)-
Formula C10H18O, MW 154, CAS# 562-74-3, Entry# 38346
1-ISOPROPYL-4-METHYL-3-CYCLOHEXEN-1-OL #

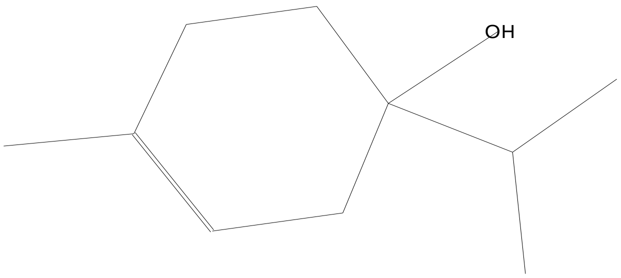


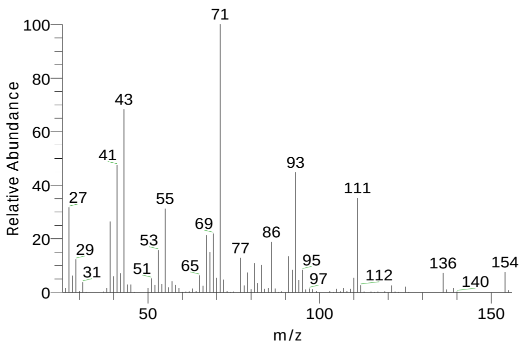

# My GC-MS Report

| Compound Structure | Hit Spectrum |
|---|---|

3-CYCLOHEXEN-1-OL, 4-METHYL-1-(1-METHYLETHYL)-
Formula C10H18O, MW 154, CAS# 562-74-3, Entry# 38347
1-ISOPROPYL-4-METHYL-3-CYCLOHEXEN-1-OL #

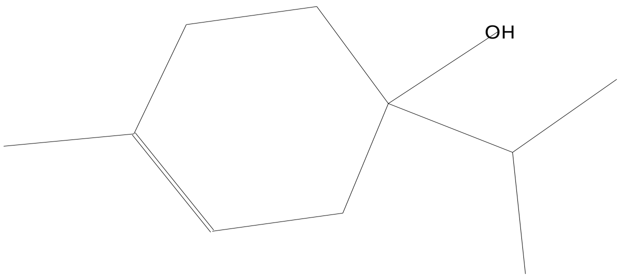


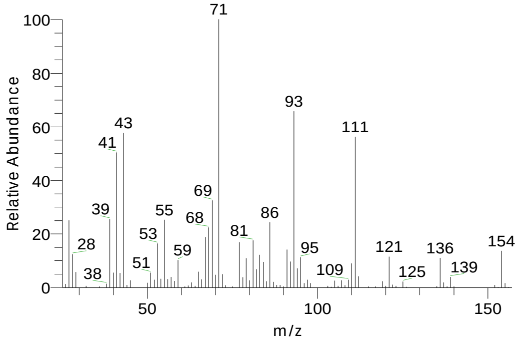


SI 853, RSI 858, replib, Entry# 9168, CAS# 562-74-3, Terpinen-4-ol

Terpinen-4-ol
Formula C10H18O, MW 154, CAS# 562-74-3, Entry# 9168
3-Cyclohexen-1-ol, 4-methyl-1-(1-methylethyl)-

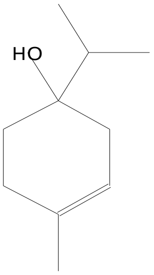


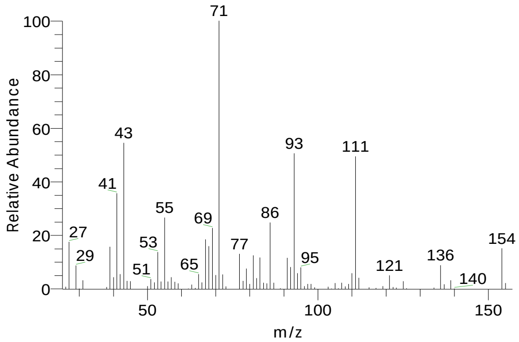


1-ISOPROPYL-4-METHYL-3-CYCLOHEXEN-1-OL
Formula C10H18O, MW 154, CAS# NA, Entry# 383374
4-TERPINEOL

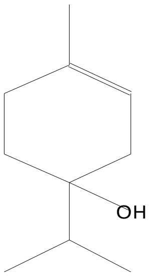


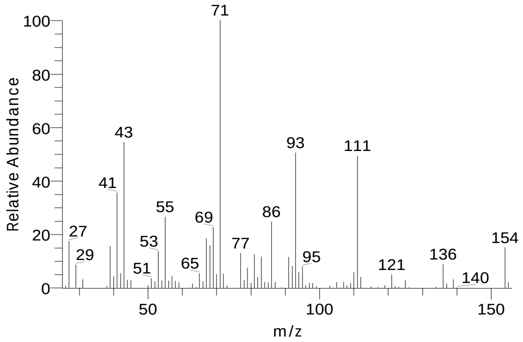


Lavender_oil #455 RT: 5.52 AV: 1 NL: 1.13E8
T: + c EI Full ms [50.000-750.000]

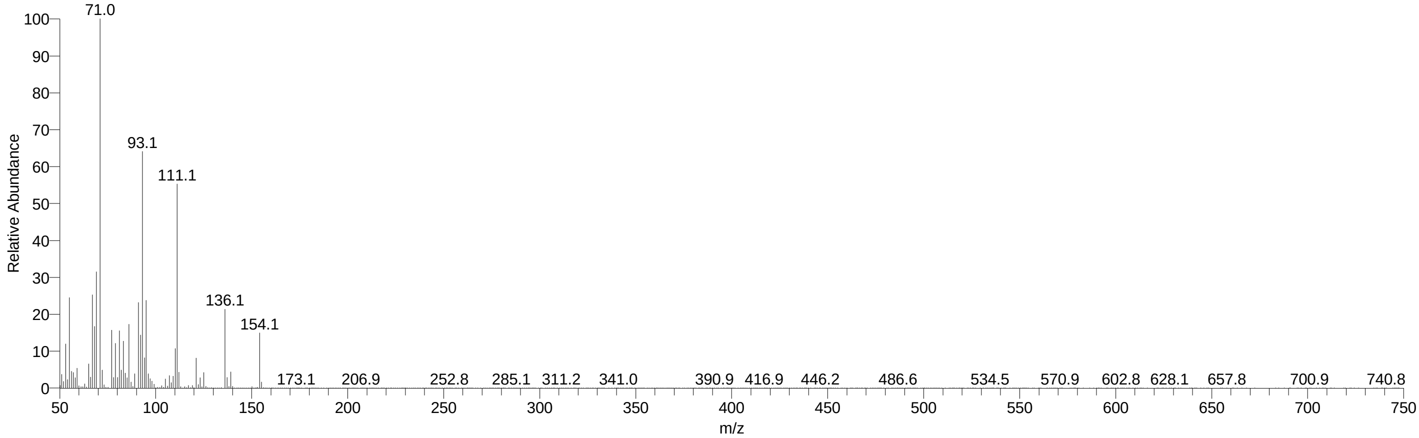


| RT | Compound Name | Area % | MF | Molecular Formula | Molecular Weight | Cas # | Library |
|---|---|---|---|---|---|---|---|
| 5.52 | 3-CYCLOHEXEN-1-OL, 4-METHYL-1-(1-METHYLETHYL)- | 8.24 | 907 | C10H18O | 154 | 562-74-3 | WileyRegistry8e |

# My GC-MS Report

| RT | Compound Name | Area % | MF | Molecular Formula | Molecular Weight | Cas # | Library |
|---|---|---|---|---|---|---|---|
| 5.52 | 3-CYCLOHEXEN-1-OL, 4-METHYL-1-(1-METHYLETHYL)- | 8.24 | 892 | C10H18O | 154 | 562-74-3 | WileyRegistry8e |
| 5.52 | 3-CYCLOHEXEN-1-OL, 4-METHYL-1-(1-METHYLETHYL)- | 8.24 | 888 | C10H18O | 154 | 562-74-3 | WileyRegistry8e |
| 5.52 | 3-CYCLOHEXEN-1-OL, 4-METHYL-1-(1-METHYLETHYL)- | 8.24 | 882 | C10H18O | 154 | 562-74-3 | WileyRegistry8e |
| 5.52 | 3-CYCLOHEXEN-1-OL, 4-METHYL-1-(1-METHYLETHYL)- | 8.24 | 881 | C10H18O | 154 | 562-74-3 | WileyRegistry8e |

| Compound Structure | Hit Spectrum |
|---|---|

3-CYCLOHEXEN-1-OL, 4-METHYL-1-(1-METHYLETHYL)-
Formula C10H18O, MW 154, CAS# 562-74-3, Entry# 38350
1-ISOPROPYL-4-METHYL-3-CYCLOHEXEN-1-OL #

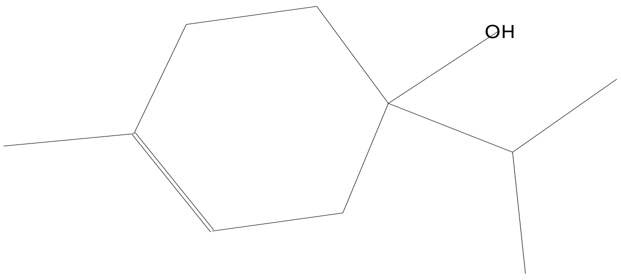


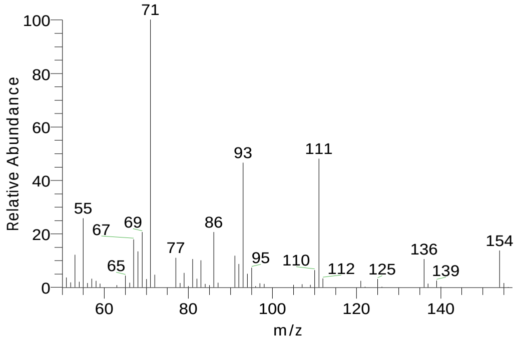


3-CYCLOHEXEN-1-OL, 4-METHYL-1-(1-METHYLETHYL)-
Formula C10H18O, MW 154, CAS# 562-74-3, Entry# 38347
1-ISOPROPYL-4-METHYL-3-CYCLOHEXEN-1-OL #

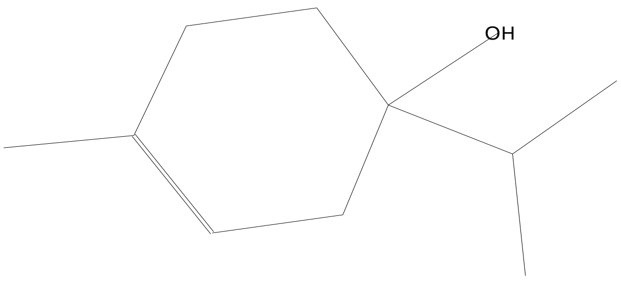


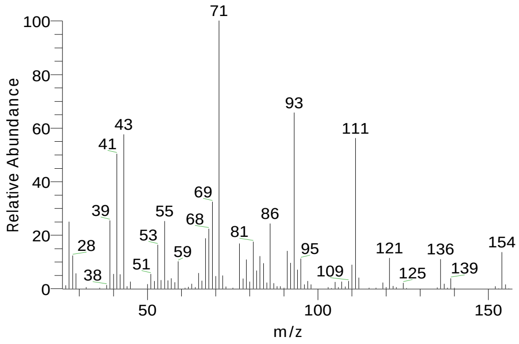


3-CYCLOHEXEN-1-OL, 4-METHYL-1-(1-METHYLETHYL)-
Formula C10H18O, MW 154, CAS# 562-74-3, Entry# 38346
1-ISOPROPYL-4-METHYL-3-CYCLOHEXEN-1-OL #

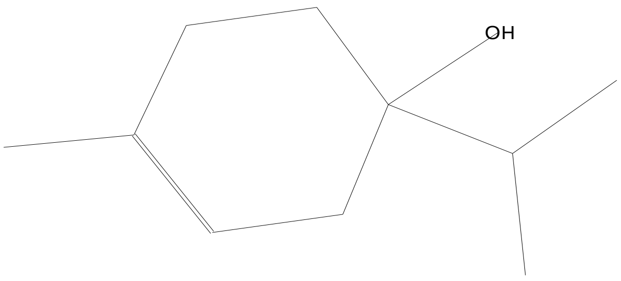


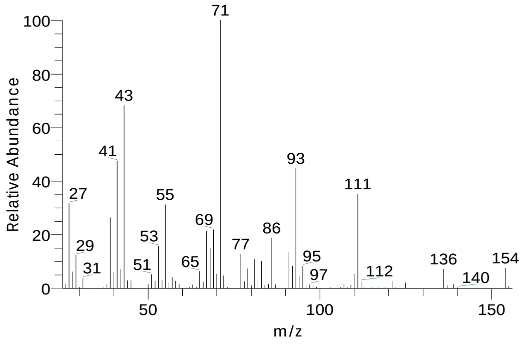

# *My GC-MS Report*

Compound Structure | Hit Spectrum

3-CYCLOHEXEN-1-OL, 4-METHYL-1-(1-METHYLETHYL)-
Formula C10H18O, MW 154, CAS# 562-74-3, Entry# 38348
1-ISOPROPYL-4-METHYL-3-CYCLOHEXEN-1-OL #

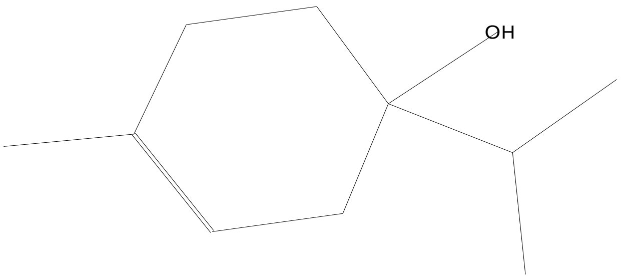


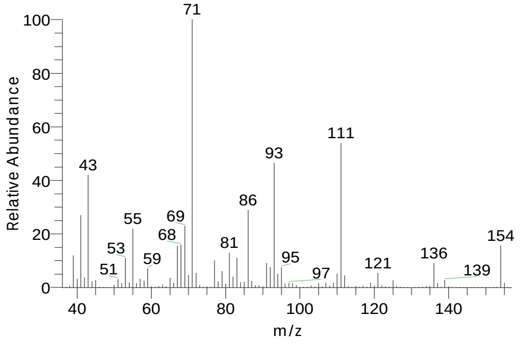


3-CYCLOHEXEN-1-OL, 4-METHYL-1-(1-METHYLETHYL)-
Formula C10H18O, MW 154, CAS# 562-74-3, Entry# 38345
1-ISOPROPYL-4-METHYL-3-CYCLOHEXEN-1-OL #

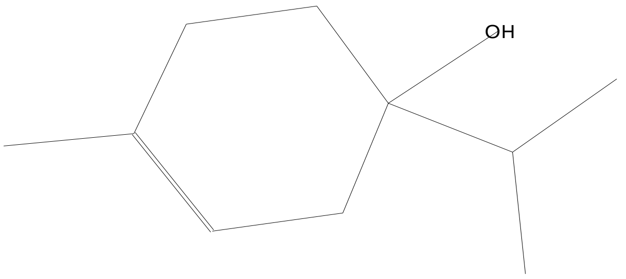


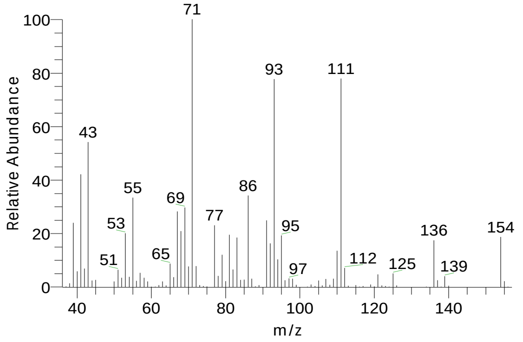


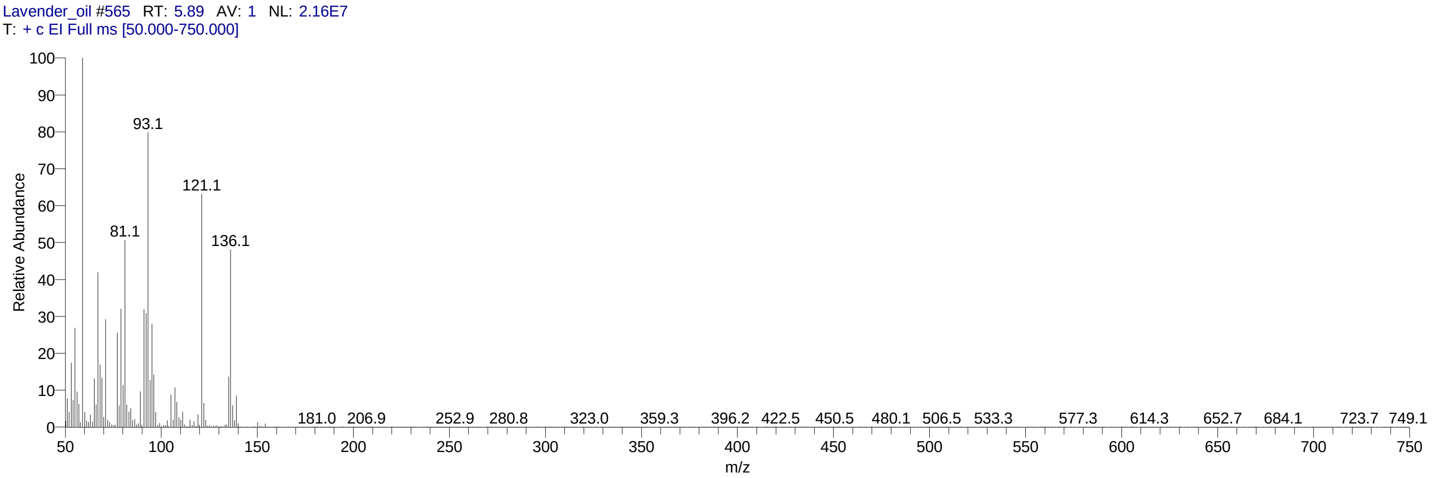


| RT | Compound Name | Area % | MF | Molecular Formula | Molecular Weight | Cas # | Library |
|---|---|---|---|---|---|---|---|
| 5.89 | L-à-Terpineol | 0.70 | 875 | C10H18O | 154 | 10482-56-1 | replib |
| 5.89 | à-Terpineol | 0.70 | 912 | C10H18O | 154 | 98-55-5 | mainlib |
| 5.89 | 3-CYCLOHEXENE-1-METHANOL, à,à,4-TRIMETHYL- | 0.70 | 882 | C10H18O | 154 | 98-55-5 | WileyRegistry8e |
| 5.89 | 3-CYCLOHEXENE-1-METHANOL, à,à,4-TRIMETHYL-, (S)- | 0.70 | 893 | C10H18O | 154 | 10482-56-1 | WileyRegistry8e |
| 5.89 | (-)-(1S,2R,4R)-BETA-FENCHOL | 0.70 | 882 | C10H18O | 154 | 470-08-6 | WileyRegistry8e |

# My GC-MS Report

Compound Structure | Hit Spectrum

L-à-Terpineol
Formula C10H18O, MW 154, CAS# 10482-56-1, Entry# 7441
3-Cyclohexene-1-methanol, à,à,4-trimethyl-, (S)-

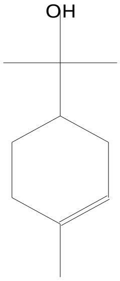


SI 866, RSI 875, replib, Entry# 7441, CAS# 10482-56-1, L-à-Terpineol

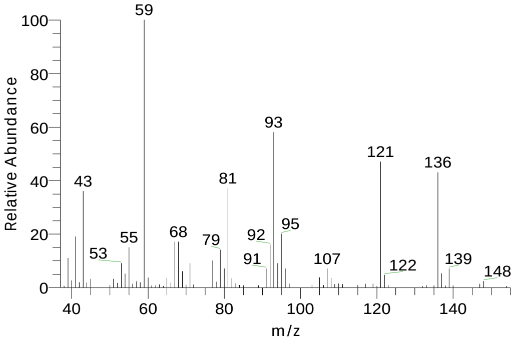


à-Terpineol
Formula C10H18O, MW 154, CAS# 98-55-5, Entry# 30324
3-Cyclohexene-1-methanol, à,à4-trimethyl-

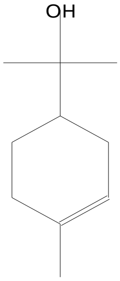


SI 863, RSI 912, mainlib, Entry# 30324, CAS# 98-55-5, à-Terpineol

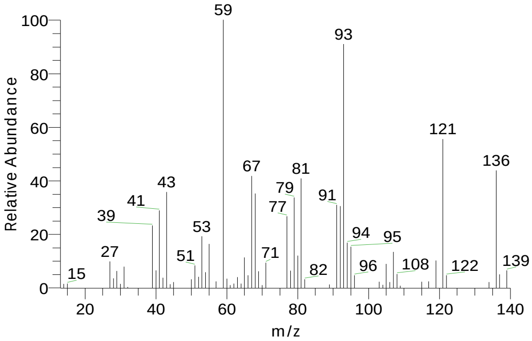


3-CYCLOHEXENE-1-METHANOL, à,à,4-TRIMETHYL-
Formula C10H18O, MW 154, CAS# 98-55-5, Entry# 38365
3-CYCLOHEXENE-1-METHANOL, à,à,4-TRIMETHYL-, (S)-

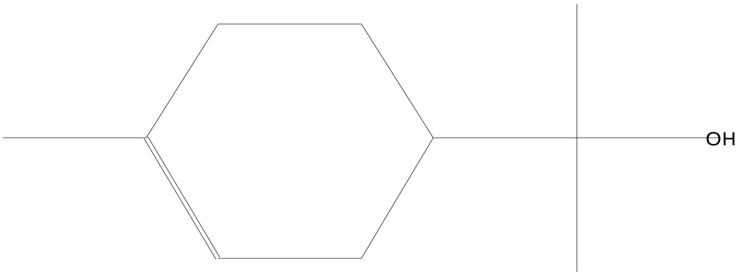


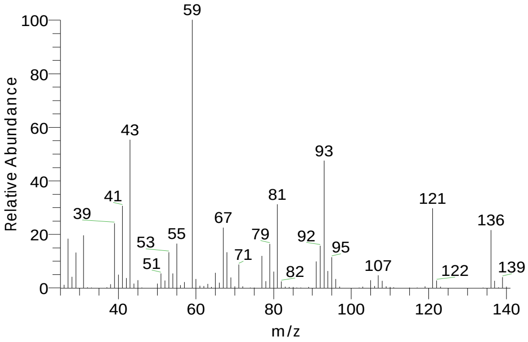


3-CYCLOHEXENE-1-METHANOL, à,à,4-TRIMETHYL-, (S)-
Formula C10H18O, MW 154, CAS# 10482-56-1, Entry# 38377
2-(4-METHYL-3-CYCLOHEXEN-1-YL)-2-PROPANOL #

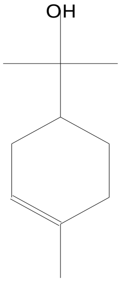


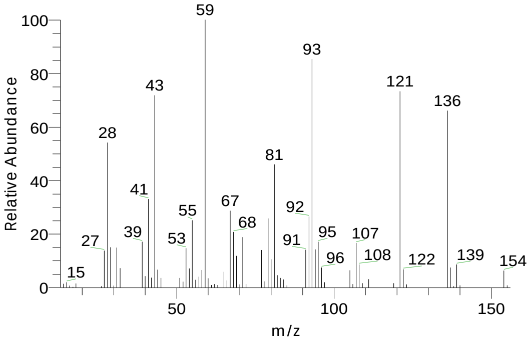

# *My GC-MS Report*

Compound Structure | Hit Spectrum

(-)-(1S,2R,4R)-BETA-FENCHOL
Formula C10H18O, MW 154, CAS# 470-08-6, Entry# 38737
(-)-BETA-FENCHOL

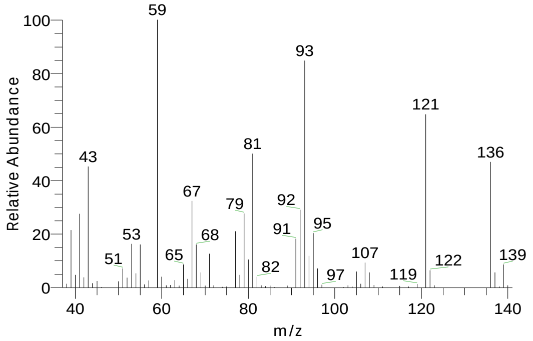


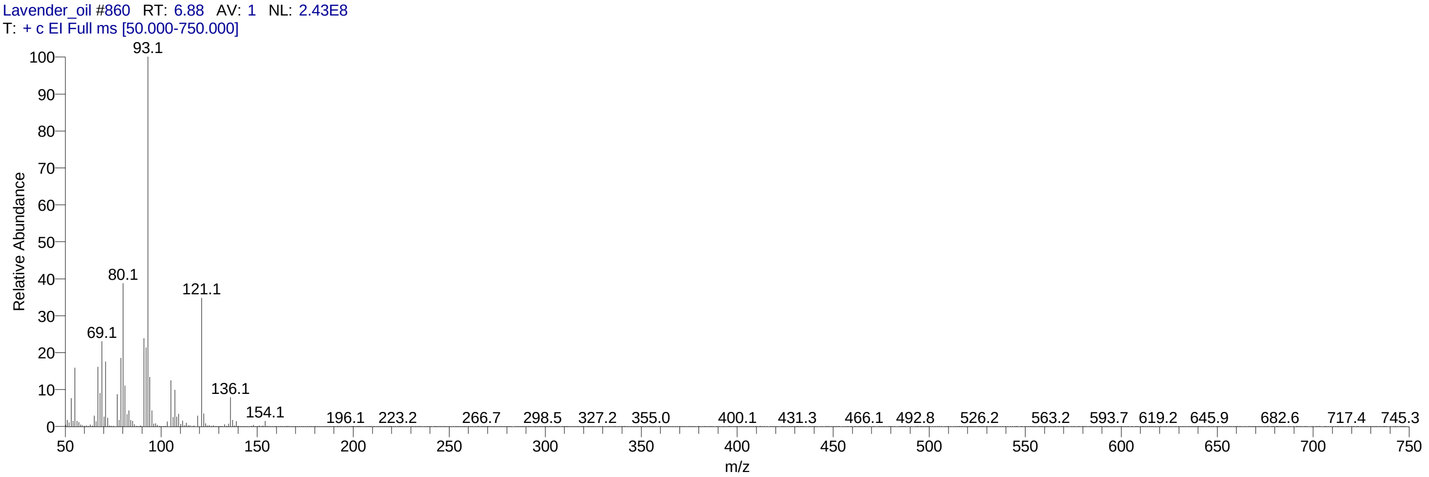


| RT | Compound Name | Area % | MF | Molecular Formula | Molecular Weight | Cas # | Library |
|---|---|---|---|---|---|---|---|
| 6.88 | 1,6-OCTADIEN-3-OL, 3,7-DIMETHYL-, ACETATE | 6.10 | 950 | C12H20O2 | 196 | 115-95-7 | WileyRegistry8e |
| 6.88 | 1,5-DIMETHYL-1-VINYL-4-HEXENYL 2-AMINOBENZOATE # | 6.10 | 949 | C17H23NO2 | 273 | 7149-26-0 | WileyRegistry8e |
| 6.88 | Linalyl acetate | 6.10 | 943 | C12H20O2 | 196 | 115-95-7 | mainlib |
| 6.88 | 1,6-OCTADIEN-3-OL, 3,7-DIMETHYL-, ACETATE | 6.10 | 947 | C12H20O2 | 196 | 115-95-7 | WileyRegistry8e |
| 6.88 | 1,6-OCTADIEN-3-OL, 3,7-DIMETHYL-, ACETATE | 6.10 | 940 | C12H20O2 | 196 | 115-95-7 | WileyRegistry8e |

Compound Structure | Hit Spectrum

1,6-OCTADIEN-3-OL, 3,7-DIMETHYL-, ACETATE
Formula C12H20O2, MW 196, CAS# 115-95-7, Entry# 80154
(1)-1,5-DIMETHYL-1-VINYLHEX-4-ENYL ACETATE

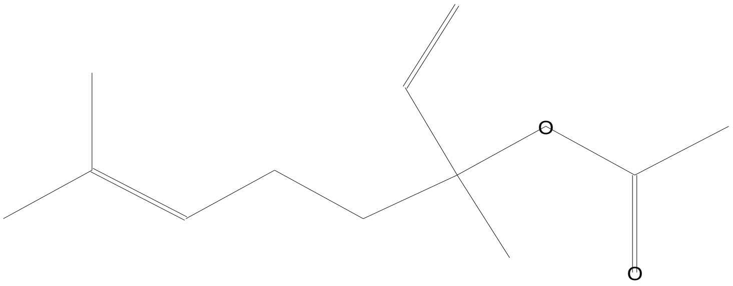


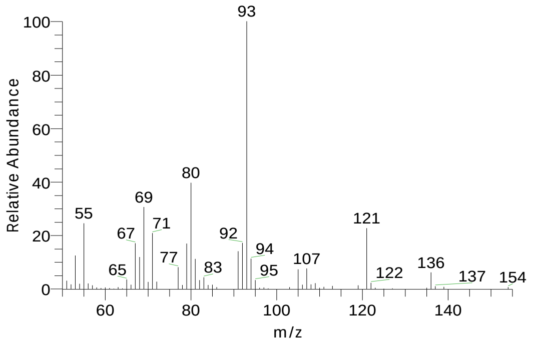

# *My GC-MS Report*

Compound Structure | Hit Spectrum

1,5-DIMETHYL-1-VINYL-4-HEXENYL 2-AMINOBENZOATE #
Formula C17H23NO2, MW 273, CAS# 7149-26-0, Entry# 163952
1, 6-OCTADIEN-3-OL, 3,7-DIMETHYL-, 2-AMINOBENZOATE

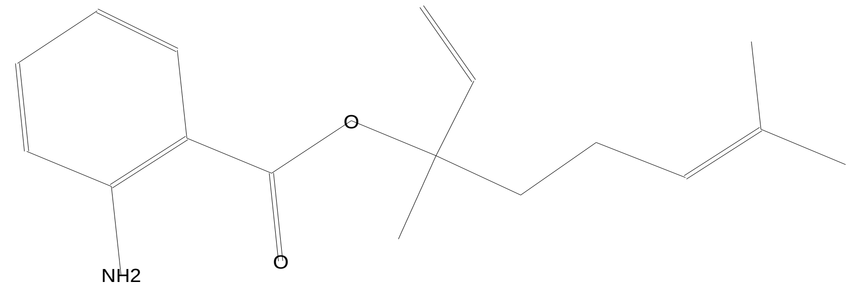


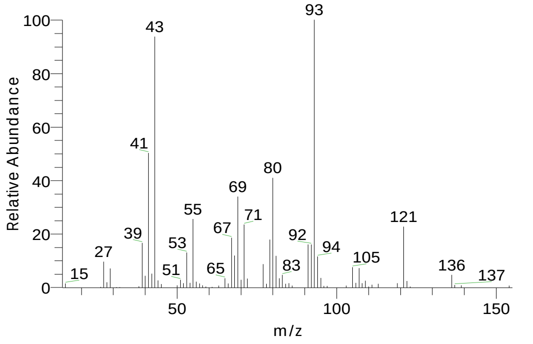


SI 941, RSI 943, mainlib, Entry# 65823, CAS# 115-95-7, Linalyl acetate

Linalyl acetate
Formula C12H20O2, MW 196, CAS# 115-95-7, Entry# 65823
1,6-Octadien-3-ol, 3,7-dimethyl-, acetate

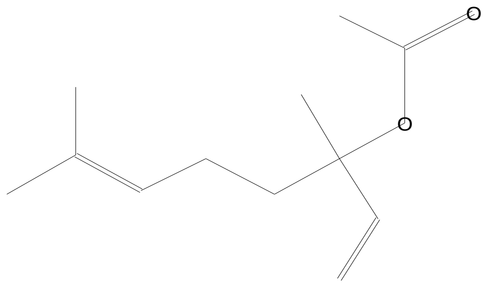


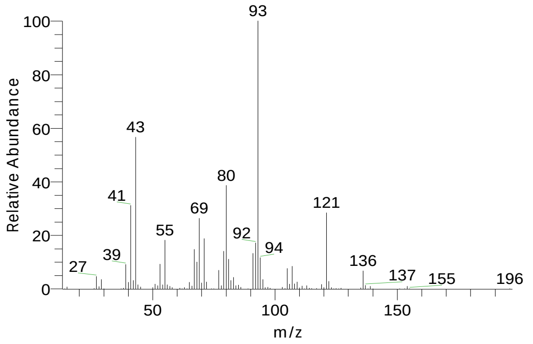


1,6-OCTADIEN-3-OL, 3,7-DIMETHYL-, ACETATE
Formula C12H20O2, MW 196, CAS# 115-95-7, Entry# 80128
(1)-1,5-DIMETHYL-1-VINYLHEX-4-ENYL ACETATE

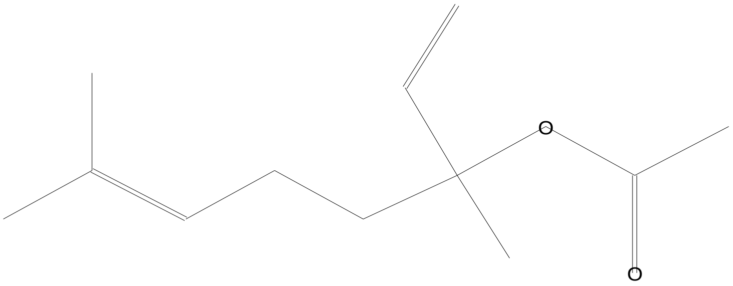


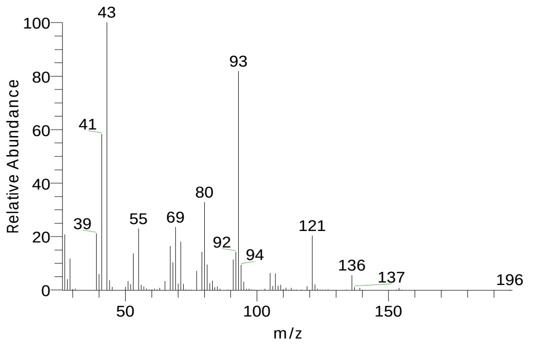


1,6-OCTADIEN-3-OL, 3,7-DIMETHYL-, ACETATE
Formula C12H20O2, MW 196, CAS# 115-95-7, Entry# 80148
(1)-1,5-DIMETHYL-1-VINYLHEX-4-ENYL ACETATE

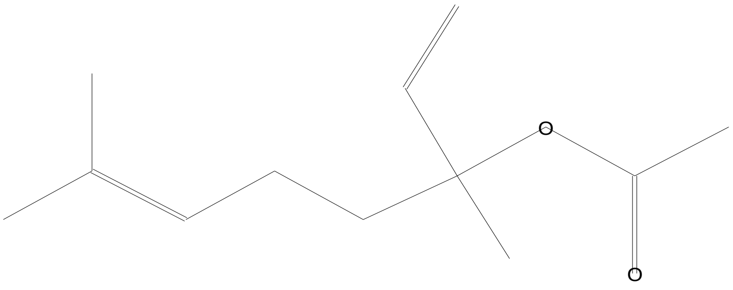


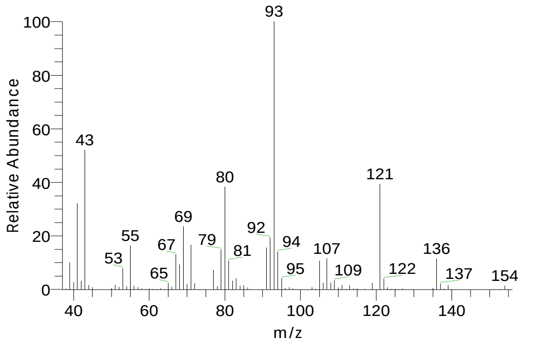

# *My GC-MS Report*

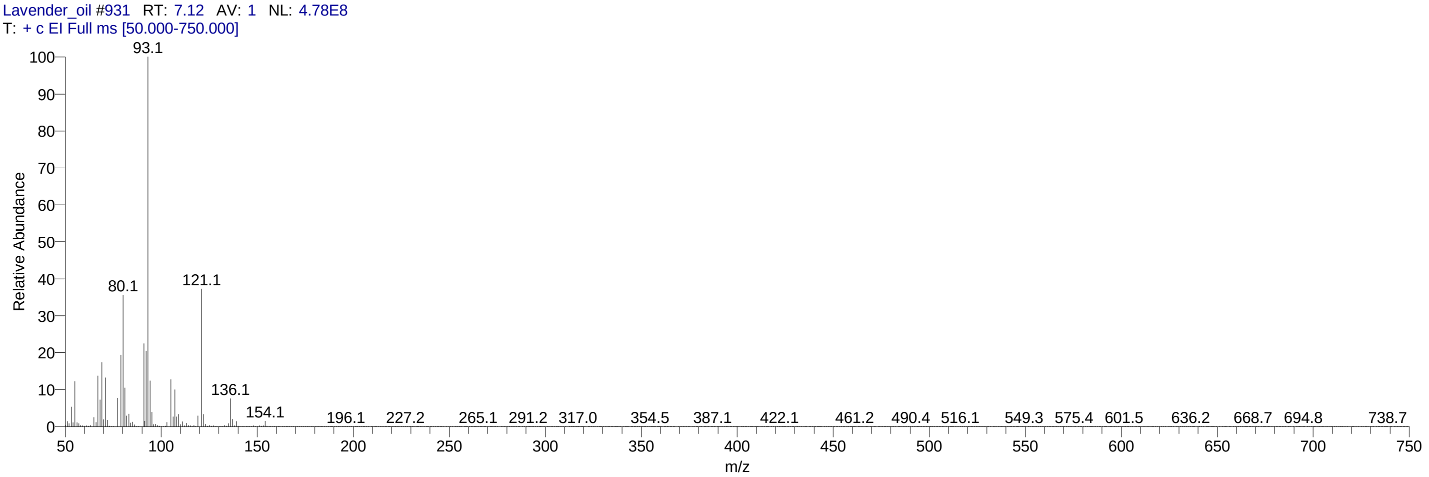


| RT | Compound Name | Area % | MF | Molecular Formula | Molecular Weight | Cas # | Library |
|---|---|---|---|---|---|---|---|
| 7.12 | 1,6-OCTADIEN-3-OL, 3,7-DIMETHYL-, ACETATE | 0.56 | 939 | C12H20O2 | 196 | 115-95-7 | WileyRegistry8e |
| 7.12 | 1,6-OCTADIEN-3-OL, 3,7-DIMETHYL-, ACETATE | 0.56 | 940 | C12H20O2 | 196 | 115-95-7 | WileyRegistry8e |
| 7.12 | Linalyl acetate | 0.56 | 936 | C12H20O2 | 196 | 115-95-7 | mainlib |
| 7.12 | 1,5-DIMETHYL-1-VINYL-4-HEXENYL 2-AMINOBENZOATE # | 0.56 | 936 | C17H23NO2 | 273 | 7149-26-0 | WileyRegistry8e |
| 7.12 | 1,6-OCTADIEN-3-OL, 3,7-DIMETHYL-, ACETATE | 0.56 | 934 | C12H20O2 | 196 | 115-95-7 | WileyRegistry8e |

Compound Structure | Hit Spectrum

1,6-OCTADIEN-3-OL, 3,7-DIMETHYL-, ACETATE
Formula C12H20O2, MW 196, CAS# 115-95-7, Entry# 80148
(1)-1,5-DIMETHYL-1-VINYLHEX-4-ENYL ACETATE

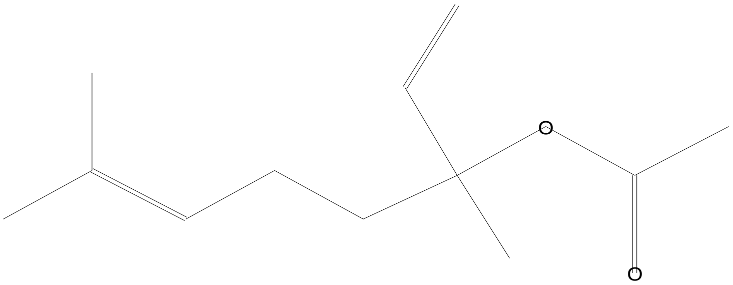


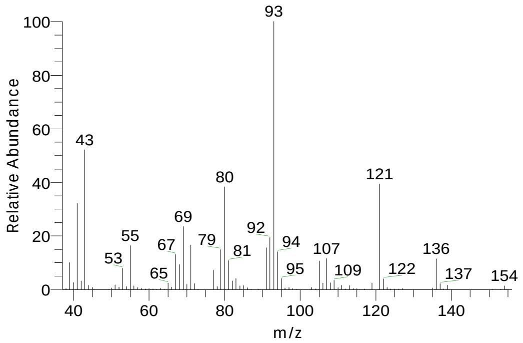


1,6-OCTADIEN-3-OL, 3,7-DIMETHYL-, ACETATE
Formula C12H20O2, MW 196, CAS# 115-95-7, Entry# 80128
(1)-1,5-DIMETHYL-1-VINYLHEX-4-ENYL ACETATE

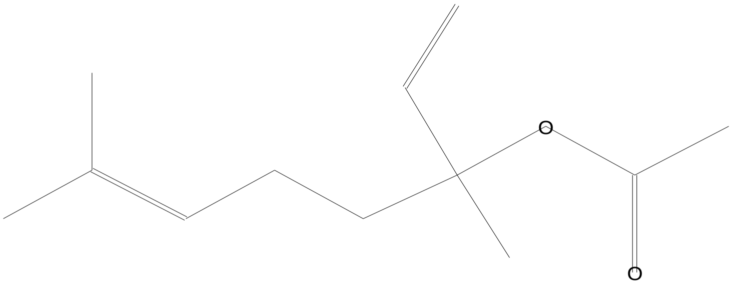


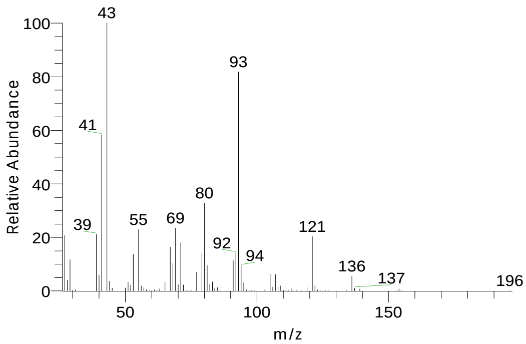

# *My GC-MS Report*

| Compound Structure | Hit Spectrum |
|---|---|

Linalyl acetate
Formula C12H20O2, MW 196, CAS# 115-95-7, Entry# 65823
1,6-Octadien-3-ol, 3,7-dimethyl-, acetate

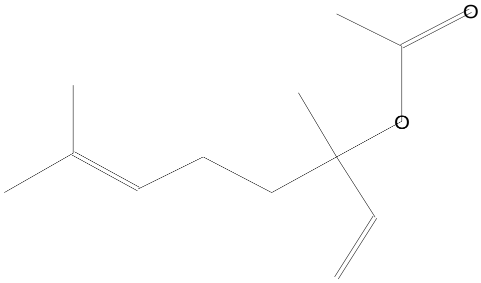


SI 935, RSI 936, mainlib, Entry# 65823, CAS# 115-95-7, Linalyl acetate

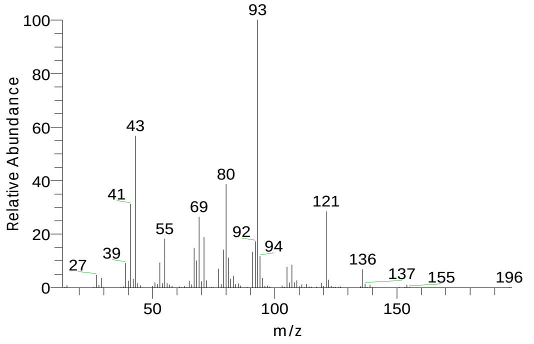


1,5-DIMETHYL-1-VINYL-4-HEXENYL 2-AMINOBENZOATE #
Formula C17H23NO2, MW 273, CAS# 7149-26-0, Entry# 163952
1, 6-OCTADIEN-3-OL, 3,7-DIMETHYL-, 2-AMINOBENZOATE

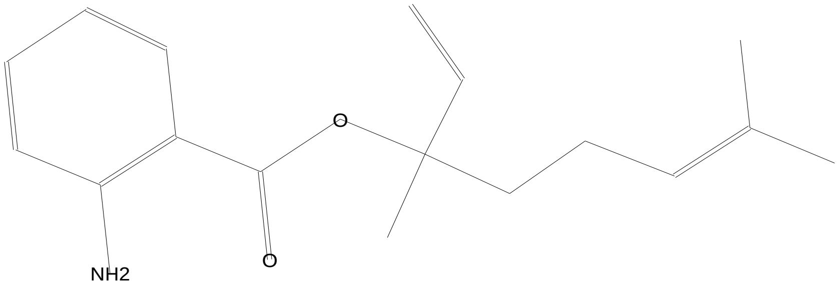


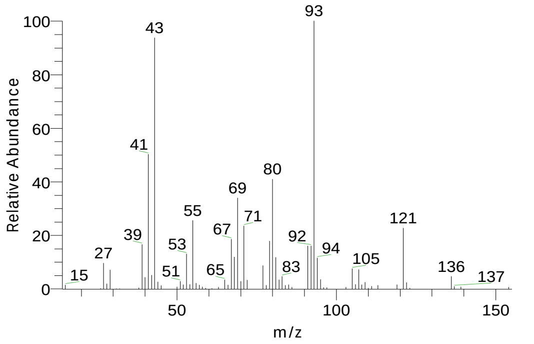


1,6-OCTADIEN-3-OL, 3,7-DIMETHYL-, ACETATE
Formula C12H20O2, MW 196, CAS# 115-95-7, Entry# 80154
(1)-1,5-DIMETHYL-1-VINYLHEX-4-ENYL ACETATE

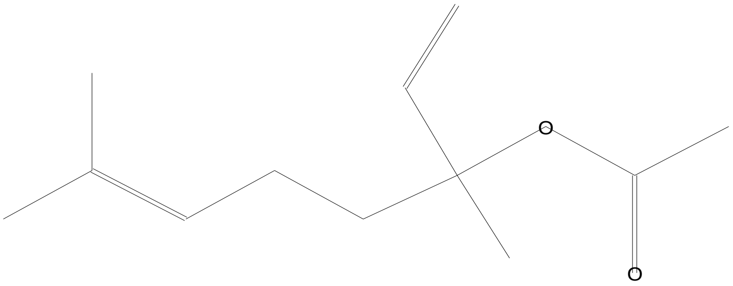


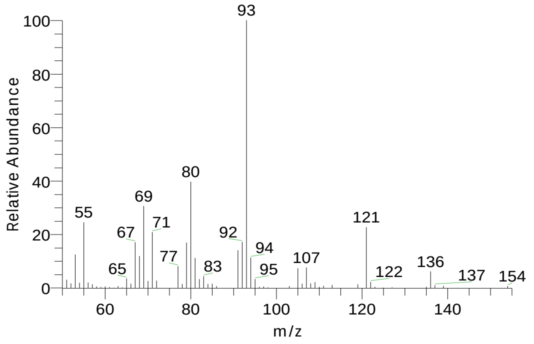


Lavender_oil #994 RT: 7.33 AV: 1 NL: 6.03E8
T: + c EI Full ms [50.000-750.000]

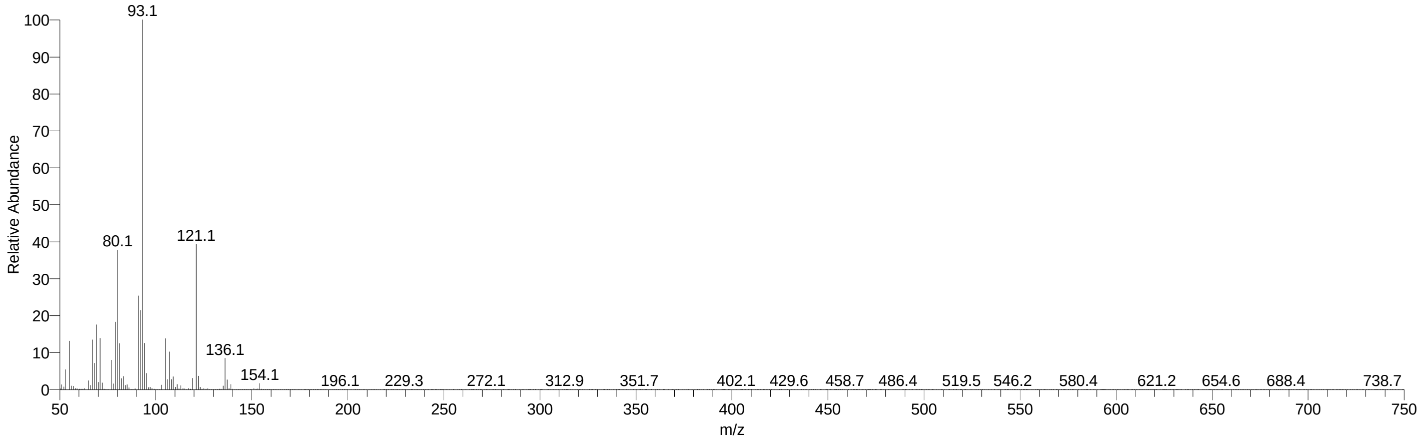


| RT | Compound Name | Area % | MF | Molecular Formula | Molecular Weight | Cas # | Library |
|---|---|---|---|---|---|---|---|
| 7.33 | 1,5-DIMETHYL-1-VINYL-4-HEXENYL 2-AMINOBENZOATE # | 21.92 | 934 | C17H23NO2 | 273 | 7149-26-0 | WileyRegistry8e |
| 7.33 | 1,6-OCTADIEN-3-OL, 3,7-DIMETHYL-, ACETATE | 21.92 | 933 | C12H20O2 | 196 | 115-95-7 | WileyRegistry8e |

# *My GC-MS Report*

| RT | Compound Name | Area % | MF | Molecular Formula | Molecular Weight | Cas # | Library |
|---|---|---|---|---|---|---|---|
| 7.33 | 1,6-OCTADIEN-3-OL, 3,7-DIMETHYL-, ACETATE | 21.92 | 928 | C12H20O2 | 196 | 115-95-7 | WileyRegistry8e |
| 7.33 | 1,6-OCTADIEN-3-OL, 3,7-DIMETHYL-, ACETATE | 21.92 | 926 | C12H20O2 | 196 | 115-95-7 | WileyRegistry8e |
| 7.33 | Linalyl acetate | 21.92 | 924 | C12H20O2 | 196 | 115-95-7 | mainlib |

| Compound Structure | Hit Spectrum |
|---|---|

1,5-DIMETHYL-1-VINYL-4-HEXENYL 2-AMINOBENZOATE #
Formula C17H23NO2, MW 273, CAS# 7149-26-0, Entry# 163952
1, 6-OCTADIEN-3-OL, 3,7-DIMETHYL-, 2-AMINOBENZOATE

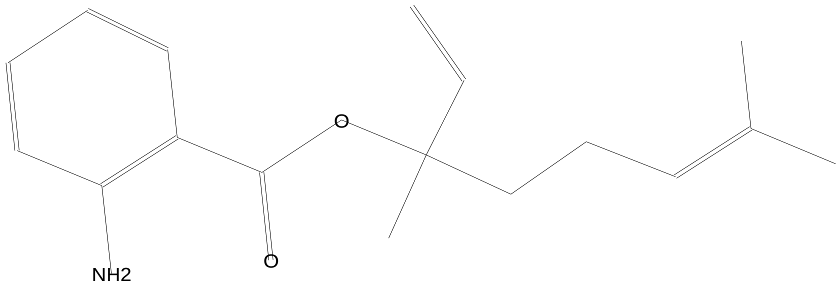


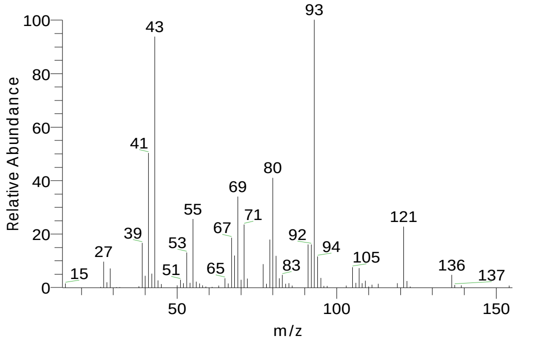


1,6-OCTADIEN-3-OL, 3,7-DIMETHYL-, ACETATE
Formula C12H20O2, MW 196, CAS# 115-95-7, Entry# 80128
(1)-1,5-DIMETHYL-1-VINYLHEX-4-ENYL ACETATE

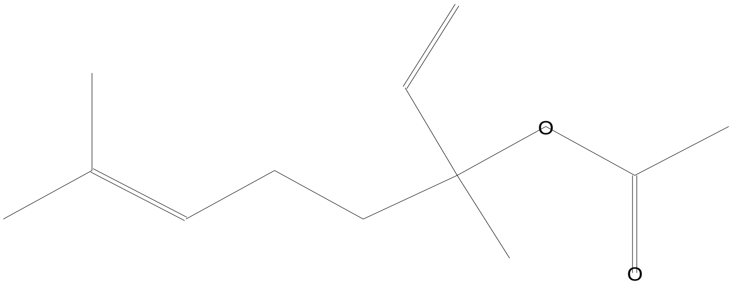


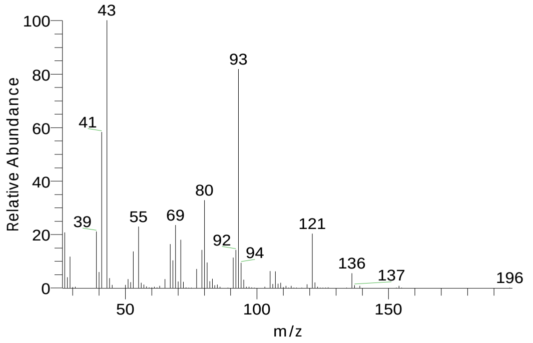


1,6-OCTADIEN-3-OL, 3,7-DIMETHYL-, ACETATE
Formula C12H20O2, MW 196, CAS# 115-95-7, Entry# 80154
(1)-1,5-DIMETHYL-1-VINYLHEX-4-ENYL ACETATE

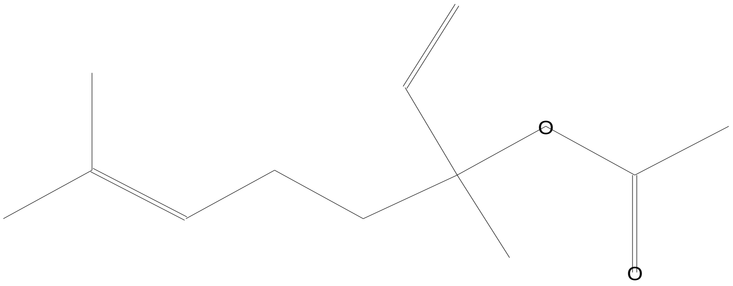


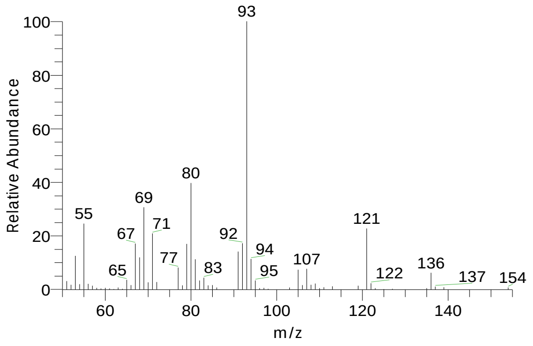


1,6-OCTADIEN-3-OL, 3,7-DIMETHYL-, ACETATE
Formula C12H20O2, MW 196, CAS# 115-95-7, Entry# 80148
(1)-1,5-DIMETHYL-1-VINYLHEX-4-ENYL ACETATE

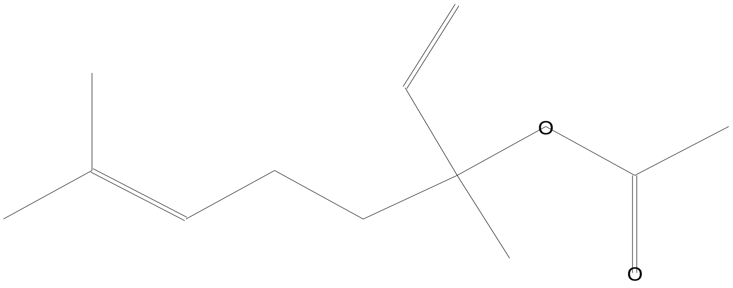


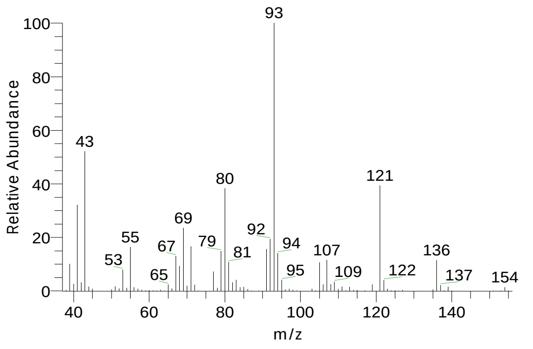

# My GC-MS Report

Compound Structure | Hit Spectrum

Linalyl acetate
Formula C12H20O2, MW 196, CAS# 115-95-7, Entry# 65823
1,6-Octadien-3-ol, 3,7-dimethyl-, acetate

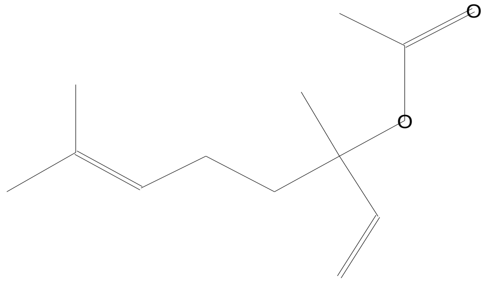


SI 924, RSI 924, mainlib, Entry# 65823, CAS# 115-95-7, Linalyl acetate

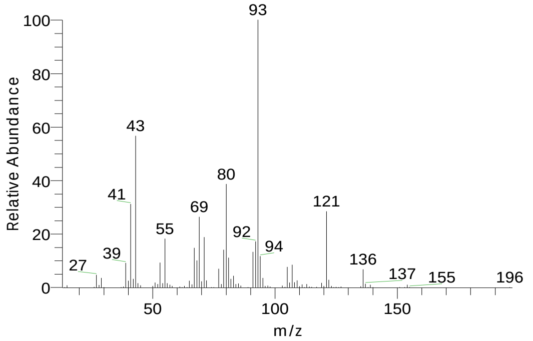


Lavender_oil #1091 RT: 7.66 AV: 1 NL: 6.76E6
T: + c EI Full ms [50.000-750.000]

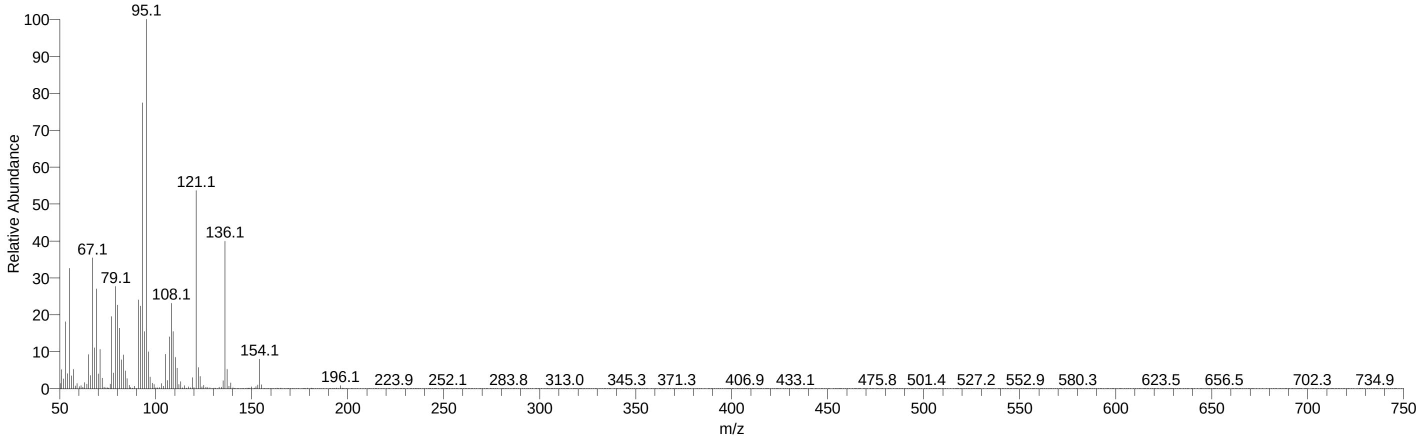


| RT | Compound Name | Area % | MF | Molecular Formula | Molecular Weight | Cas # | Library |
|---|---|---|---|---|---|---|---|
| 7.66 | Bornyl acetate | 0.10 | 919 | C12H20O2 | 196 | 76-49-3 | mainlib |
| 7.66 | BICYCLO[2.2.1]HEPTAN-2-OL, 1,7,7-TRIMETHYL-, ACETATE, (1S-ENDO)- | 0.10 | 919 | C12H20O2 | 196 | 5655-61-8 | WileyRegistry8e |
| 7.66 | BICYCLO[2.2.1]HEPTAN-2-OL, 1,7,7-TRIMETHYL-, ACETATE, ENDO- | 0.10 | 912 | C12H20O2 | 196 | 76-49-3 | WileyRegistry8e |
| 7.66 | BICYCLO[2.2.1]HEPTAN-2-OL, 1,7,7-TRIMETHYL-, ACETATE, EXO- | 0.10 | 914 | C12H20O2 | 196 | 125-12-2 | WileyRegistry8e |
| 7.66 | BICYCLO[2.2.1]HEPTAN-2-OL, 1,7,7-TRIMETHYL-, ACETATE, ENDO- | 0.10 | 914 | C12H20O2 | 196 | 76-49-3 | WileyRegistry8e |

Compound Structure | Hit Spectrum

Bornyl acetate
Formula C12H20O2, MW 196, CAS# 76-49-3, Entry# 67808
Bicyclo[2.2.1]heptan-2-ol, 1,7,7-trimethyl-, acetate, endo-

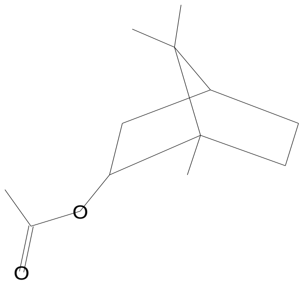


SI 917, RSI 919, mainlib, Entry# 67808, CAS# 76-49-3, Bornyl acetate

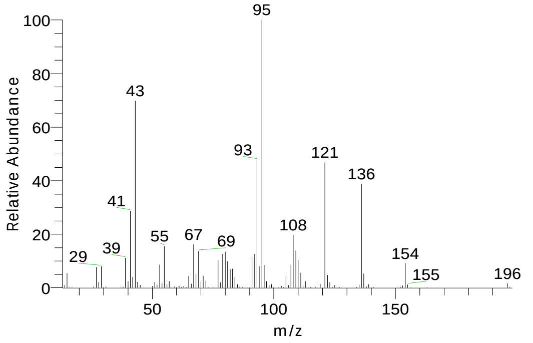

# *My GC-MS Report*

Compound Structure | Hit Spectrum

BICYCLO[2.2.1]HEPTAN-2-OL, 1,7,7-TRIMETHYL-, ACETATE, (1S-ENDO)-
Formula C12H20O2, MW 196, CAS# 5655-61-8, Entry# 80072
1,7,7-TRIMETHYLBICYCLO[2.2.1]HEPT-2-YL ACETATE #

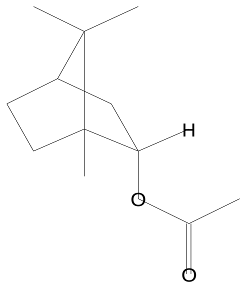


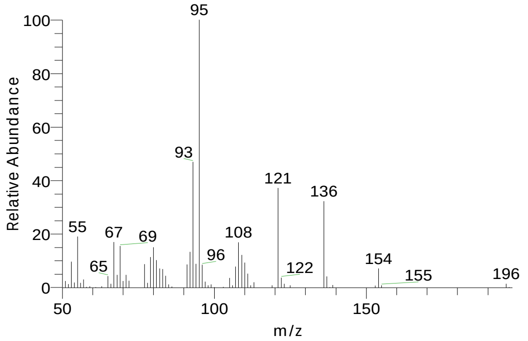


BICYCLO[2.2.1]HEPTAN-2-OL, 1,7,7-TRIMETHYL-, ACETATE, ENDO-
Formula C12H20O2, MW 196, CAS# 76-49-3, Entry# 80076
1,7,7-TRIMETHYLBICYCLO[2.2.1]HEPT-2-YL ACETATE #

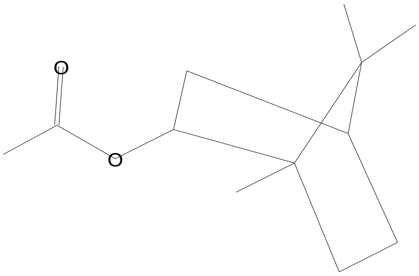


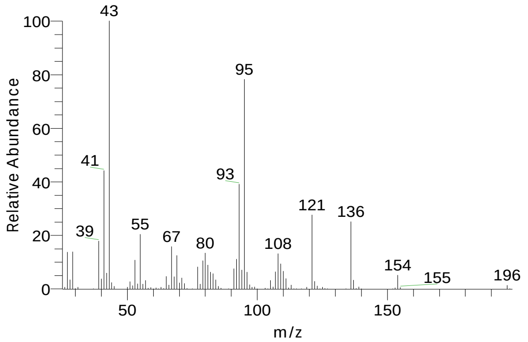


BICYCLO[2.2.1]HEPTAN-2-OL, 1,7,7-TRIMETHYL-, ACETATE, EXO-
Formula C12H20O2, MW 196, CAS# 125-12-2, Entry# 80091
1,7,7-TRIMETHYLBICYCLO[2.2.1]HEPT-2-YL ACETATE #

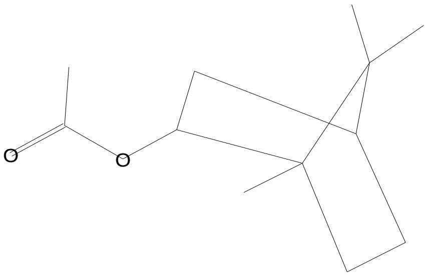


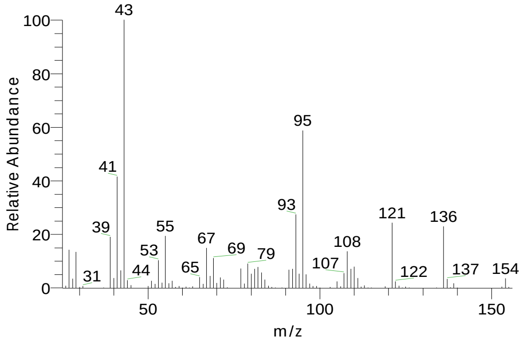


BICYCLO[2.2.1]HEPTAN-2-OL, 1,7,7-TRIMETHYL-, ACETATE, ENDO-
Formula C12H20O2, MW 196, CAS# 76-49-3, Entry# 80082
1,7,7-TRIMETHYLBICYCLO[2.2.1]HEPT-2-YL ACETATE #

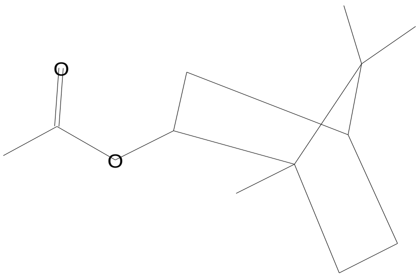


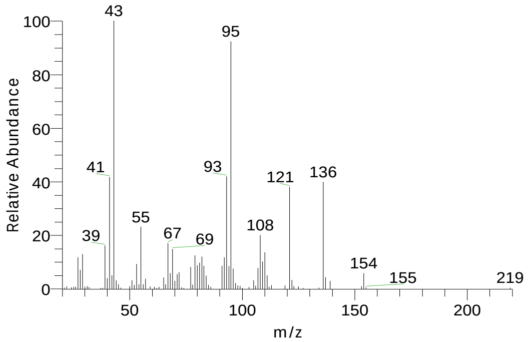

# My GC-MS Report

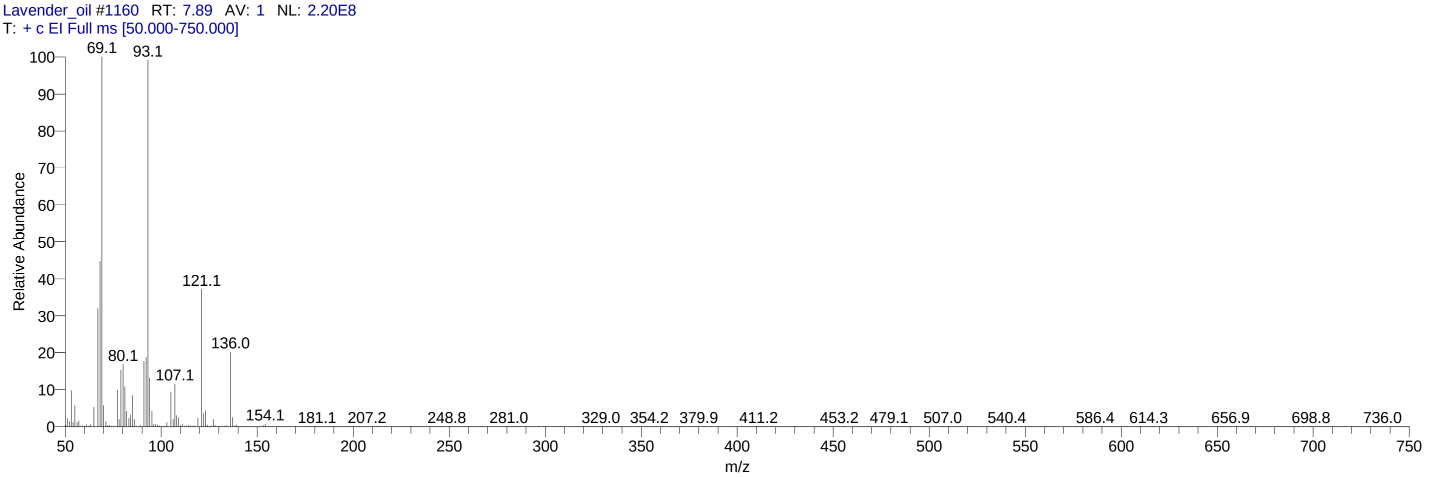


| RT | Compound Name | Area % | MF | Molecular Formula | Molecular Weight | Cas # | Library |
|---|---|---|---|---|---|---|---|
| 7.89 | 4-Hexen-1-ol, 5-methyl-2-(1-methylethenyl)-, acetate | 11.85 | 940 | C12H20O2 | 196 | 25905-14-0 | mainlib |
| 7.89 | 4-HEXEN-1-OL, 2-ISOPROPENYL-5-METHYL-, ACETATE | 11.85 | 946 | C12H20O2 | 196 | 25905-14-0 | WileyRegistry8e |
| 7.89 | 4-Hexen-1-ol, 5-methyl-2-(1-methylethenyl)-, acetate | 11.85 | 905 | C12H20O2 | 196 | 25905-14-0 | replib |
| 7.89 | 2,6-OCTADIEN-1-OL, 3,7-DIMETHYL-, ACETATE | 11.85 | 892 | C12H20O2 | 196 | 105-87-3 | WileyRegistry8e |
| 7.89 | 2,6-OCTADIEN-1-OL, 3,7-DIMETHYL-, ACETATE | 11.85 | 887 | C12H20O2 | 196 | 105-87-3 | WileyRegistry8e |

Compound Structure | Hit Spectrum

4-Hexen-1-ol, 5-methyl-2-(1-methylethenyl)-, acetate
Formula C12H20O2, MW 196, CAS# 25905-14-0, Entry# 35656
2-Isopropenyl-5-methyl-4-hexenyl acetate #

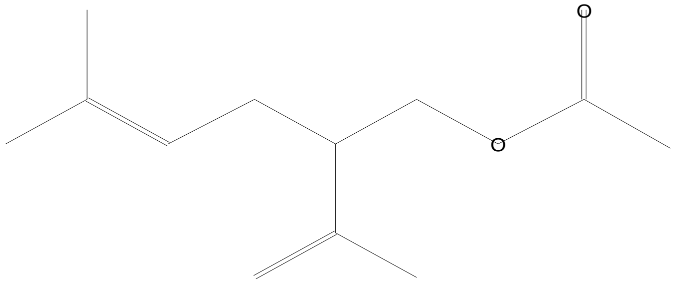

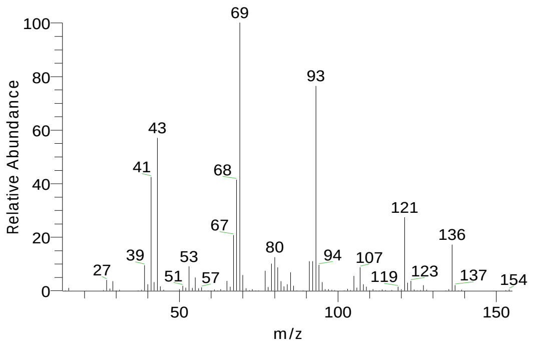


4-HEXEN-1-OL, 2-ISOPROPENYL-5-METHYL-, ACETATE
Formula C12H20O2, MW 196, CAS# 25905-14-0, Entry# 80143
2-ISOPROPENYL-5-METHYL-4-HEXENYL ACETATE #

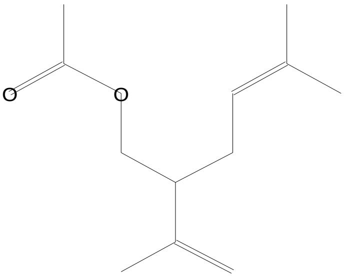

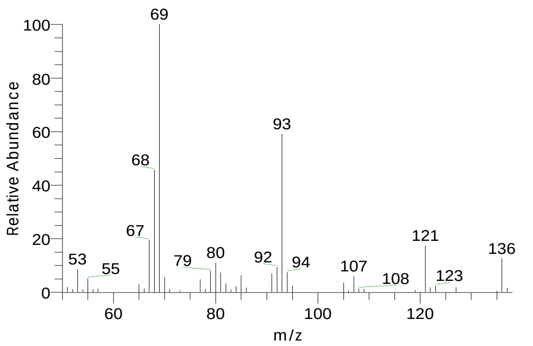

# *My GC-MS Report*

Compound Structure | Hit Spectrum

4-Hexen-1-ol, 5-methyl-2-(1-methylethenyl)-, acetate
Formula C12H20O2, MW 196, CAS# 25905-14-0, Entry# 8604
2-Isopropenyl-5-methyl-4-hexenyl acetate #

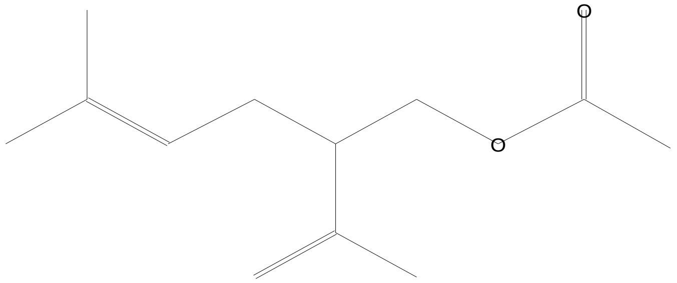


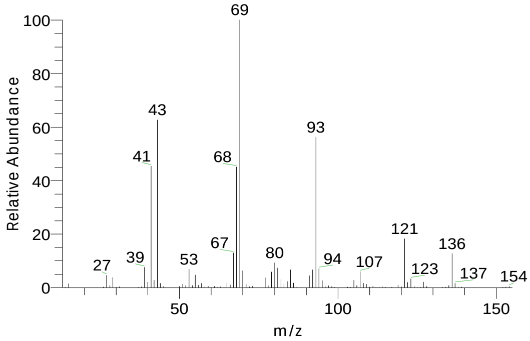


2,6-OCTADIEN-1-OL, 3,7-DIMETHYL-, ACETATE
Formula C12H20O2, MW 196, CAS# 105-87-3, Entry# 79958
2,6-OCTADIEN-1-OL, 3,7-DIMETHYL-, ACETATE, (E)-

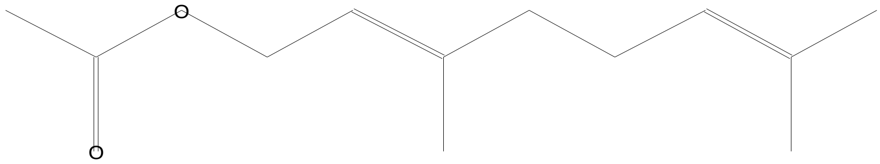


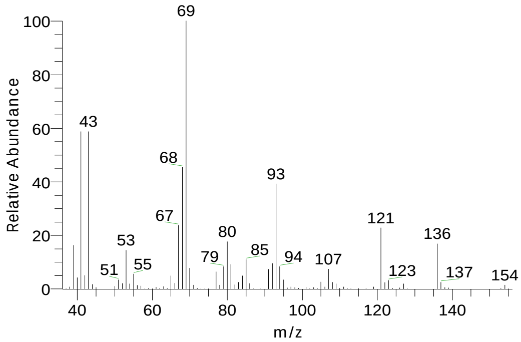


2,6-OCTADIEN-1-OL, 3,7-DIMETHYL-, ACETATE
Formula C12H20O2, MW 196, CAS# 105-87-3, Entry# 79954
2,6-OCTADIEN-1-OL, 3,7-DIMETHYL-, ACETATE, (E)-

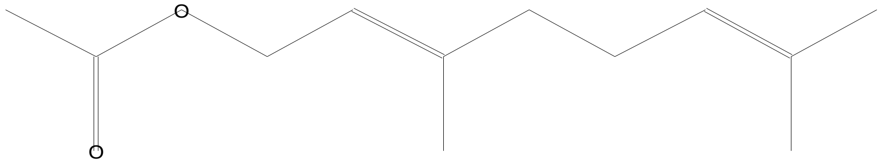


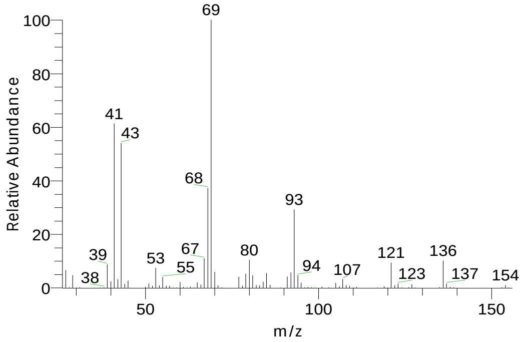


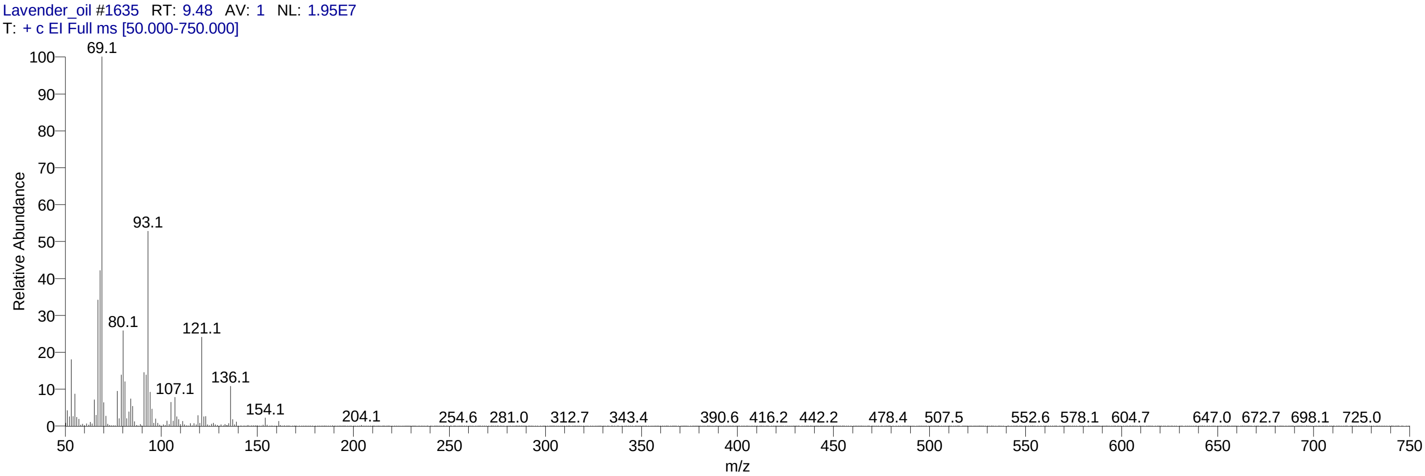


| RT | Compound Name | Area % | MF | Molecular Formula | Molecular Weight | Cas # | Library |
|---|---|---|---|---|---|---|---|
| 9.48 | 2,6-OCTADIEN-1-OL, 3,7-DIMETHYL-, ACETATE, (Z)- | 0.75 | 934 | C12H20O2 | 196 | 141-12-8 | WileyRegistry8e |
| 9.48 | 2,6-Octadien-1-ol, 3,7-dimethyl-, acetate, (Z)- | 0.75 | 925 | C12H20O2 | 196 | 141-12-8 | mainlib |

# My GC-MS Report

| RT | Compound Name | Area % | MF | Molecular Formula | Molecular Weight | Cas # | Library |
|---|---|---|---|---|---|---|---|
| 9.48 | 2,6-OCTADIEN-1-OL, 3,7-DIMETHYL-, ACETATE, (Z)- | 0.75 | 928 | C12H20O2 | 196 | 141-12-8 | WileyRegistry8e |
| 9.48 | 2,6-Octadien-1-ol, 3,7-dimethyl-, acetate, (Z)- | 0.75 | 927 | C12H20O2 | 196 | 141-12-8 | replib |
| 9.48 | 2,6-OCTADIEN-1-OL, 3,7-DIMETHYL-, ACETATE | 0.75 | 913 | C12H20O2 | 196 | 105-87-3 | WileyRegistry8e |

| Compound Structure | Hit Spectrum |
|---|---|

2,6-OCTADIEN-1-OL, 3,7-DIMETHYL-, ACETATE, (Z)-
Formula C12H20O2, MW 196, CAS# 141-12-8, Entry# 80149
(2Z)-3,7-DIMETHYL-2,6-OCTADIENYL ACETATE #

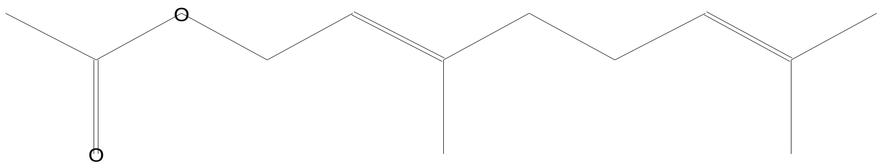


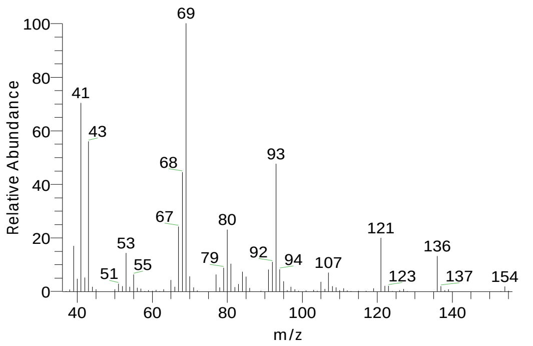


2,6-Octadien-1-ol, 3,7-dimethyl-, acetate, (Z)-
Formula C12H20O2, MW 196, CAS# 141-12-8, Entry# 35647
Nerol acetate

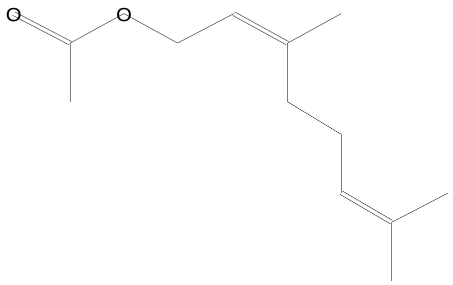


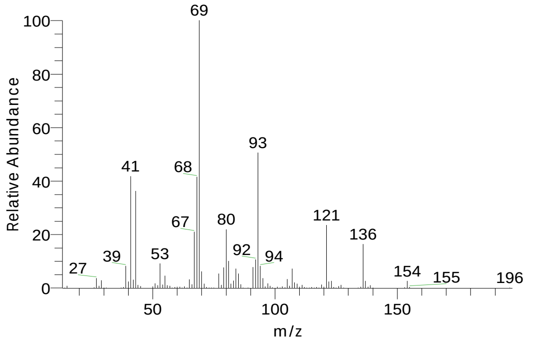


2,6-OCTADIEN-1-OL, 3,7-DIMETHYL-, ACETATE, (Z)-
Formula C12H20O2, MW 196, CAS# 141-12-8, Entry# 79948
(2Z)-3,7-DIMETHYL-2,6-OCTADIENYL ACETATE #

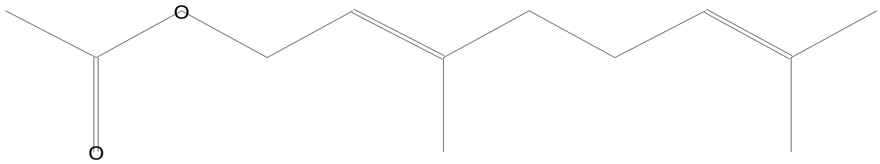


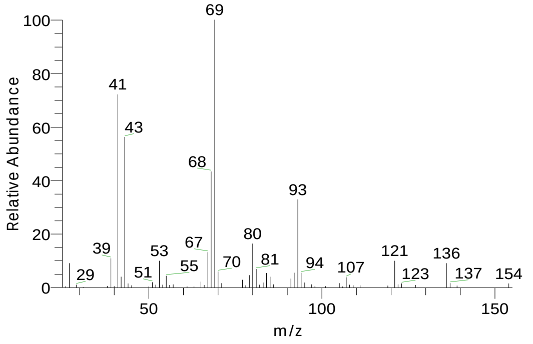


2,6-Octadien-1-ol, 3,7-dimethyl-, acetate, (Z)-
Formula C12H20O2, MW 196, CAS# 141-12-8, Entry# 8433
Nerol acetate

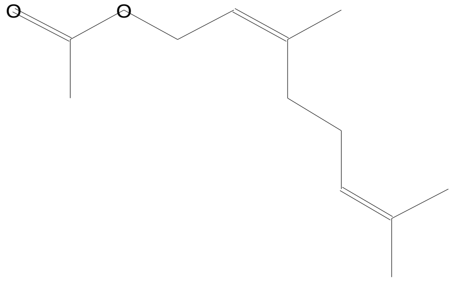


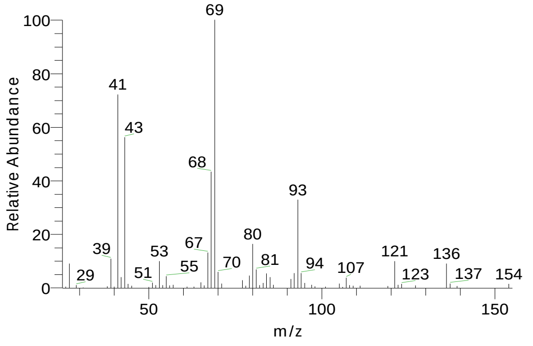

# *My GC-MS Report*

Compound Structure | Hit Spectrum

2,6-OCTADIEN-1-OL, 3,7-DIMETHYL-, ACETATE
Formula C12H20O2, MW 196, CAS# 105-87-3, Entry# 79958
2,6-OCTADIEN-1-OL, 3,7-DIMETHYL-, ACETATE, (E)-

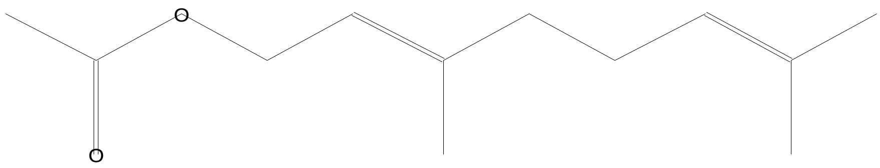

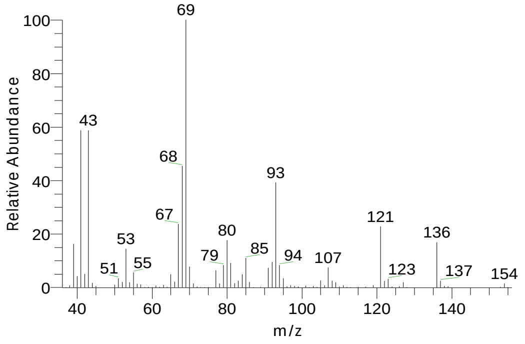


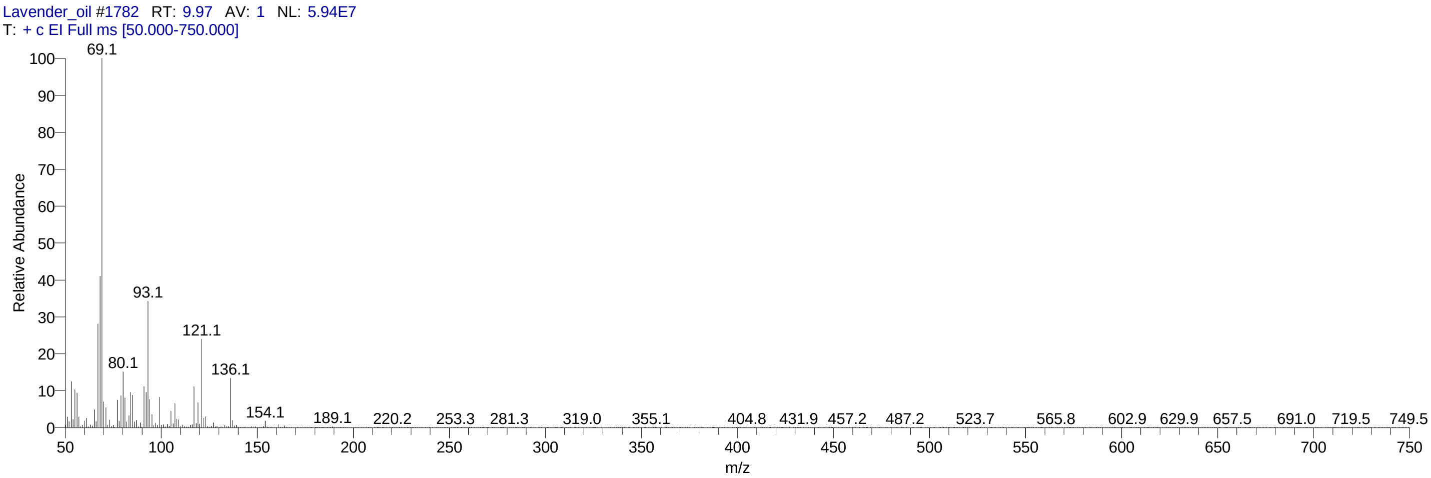


| RT | Compound Name | Area % | MF | Molecular Formula | Molecular Weight | Cas # | Library |
|---|---|---|---|---|---|---|---|
| 9.97 | 2,6-OCTADIEN-1-OL, 3,7-DIMETHYL-, ACETATE, (Z)- | 2.24 | 879 | C12H20O2 | 196 | 141-12-8 | WileyRegistry8e |
| 9.97 | 2,6-Octadien-1-ol, 3,7-dimethyl-, acetate | 2.24 | 907 | C12H20O2 | 196 | 16409-44-2 | replib |
| 9.97 | Geranyl acetate | 2.24 | 870 | C12H20O2 | 196 | 105-87-3 | mainlib |
| 9.97 | 2,6-OCTADIEN-1-OL, 3,7-DIMETHYL-, ACETATE | 2.24 | 868 | C12H20O2 | 196 | 105-87-3 | WileyRegistry8e |
| 9.97 | 2,6-OCTADIEN-1-OL, 3,7-DIMETHYL-, ACETATE | 2.24 | 871 | C12H20O2 | 196 | 105-87-3 | WileyRegistry8e |

Compound Structure | Hit Spectrum

2,6-OCTADIEN-1-OL, 3,7-DIMETHYL-, ACETATE, (Z)-
Formula C12H20O2, MW 196, CAS# 141-12-8, Entry# 80149
(2Z)-3,7-DIMETHYL-2,6-OCTADIENYL ACETATE #

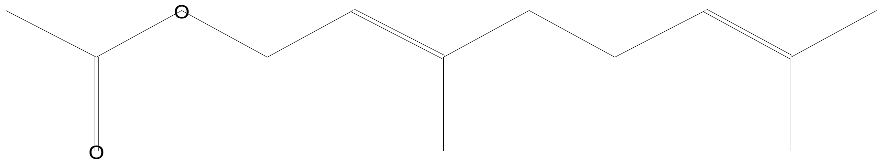

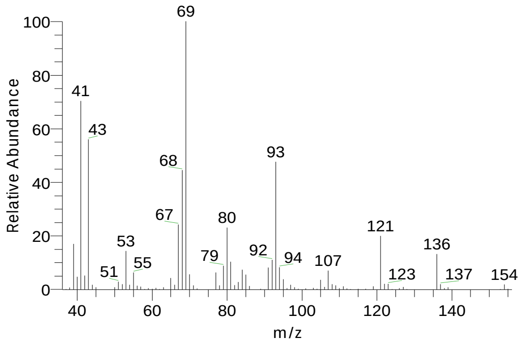

# My GC-MS Report

Compound Structure | Hit Spectrum

2,6-Octadien-1-ol, 3,7-dimethyl-, acetate
Formula C12H20O2, MW 196, CAS# 16409-44-2, Entry# 8437
2,6-Dimethyl-2,6-octadien-8-yl acetate

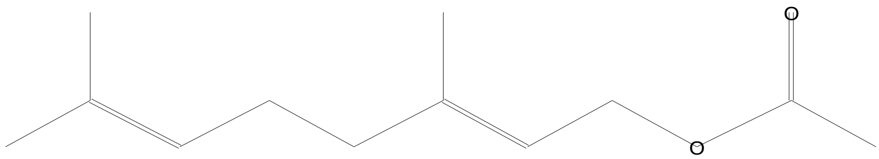


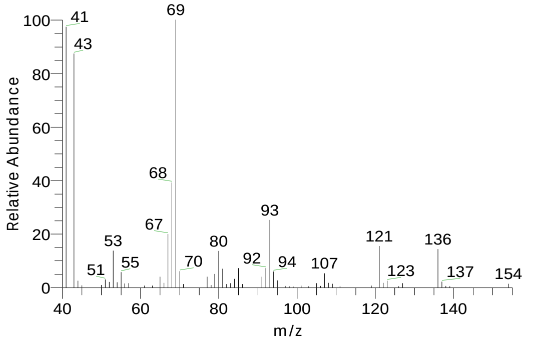


Geranyl acetate
Formula C12H20O2, MW 196, CAS# 105-87-3, Entry# 34129
2,6-Octadien-1-ol, 3,7-dimethyl-, acetate, (E)-

SI 859, RSI 870, mainlib, Entry# 34129, CAS# 105-87-3, Geranyl acetate

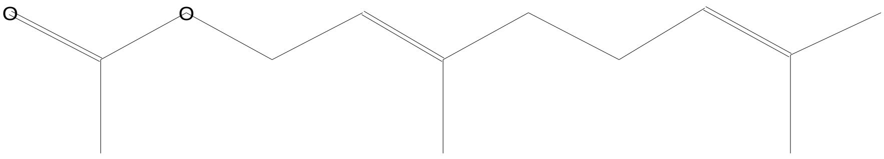


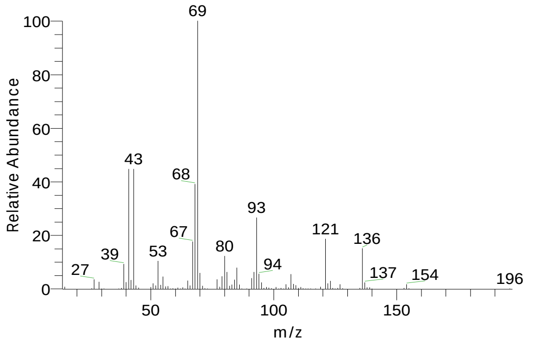


2,6-OCTADIEN-1-OL, 3,7-DIMETHYL-, ACETATE
Formula C12H20O2, MW 196, CAS# 105-87-3, Entry# 79958
2,6-OCTADIEN-1-OL, 3,7-DIMETHYL-, ACETATE, (E)-

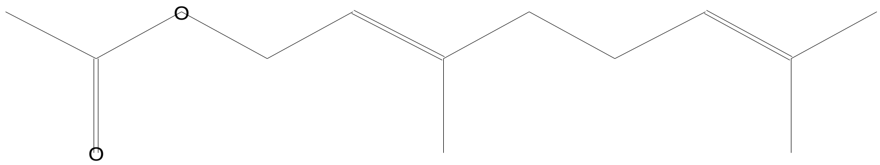


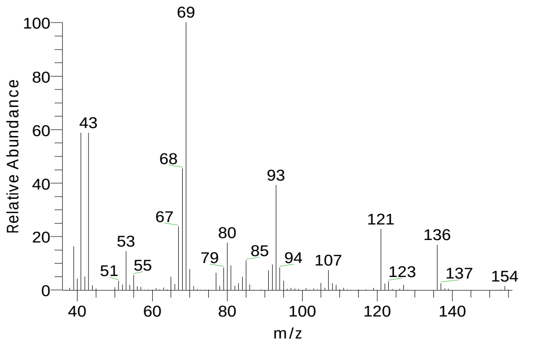


2,6-OCTADIEN-1-OL, 3,7-DIMETHYL-, ACETATE
Formula C12H20O2, MW 196, CAS# 105-87-3, Entry# 79957
2,6-OCTADIEN-1-OL, 3,7-DIMETHYL-, ACETATE, (E)-

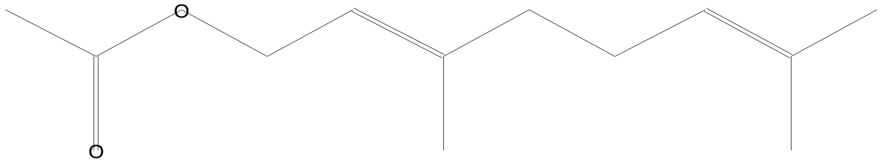


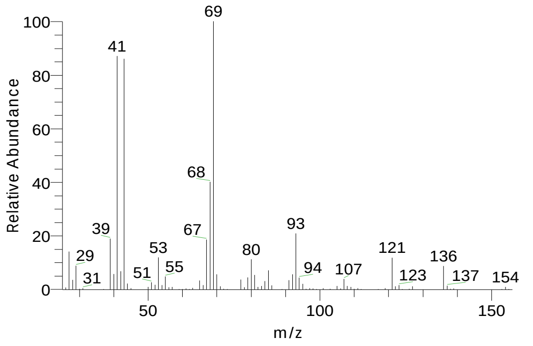

# *My GC-MS Report*

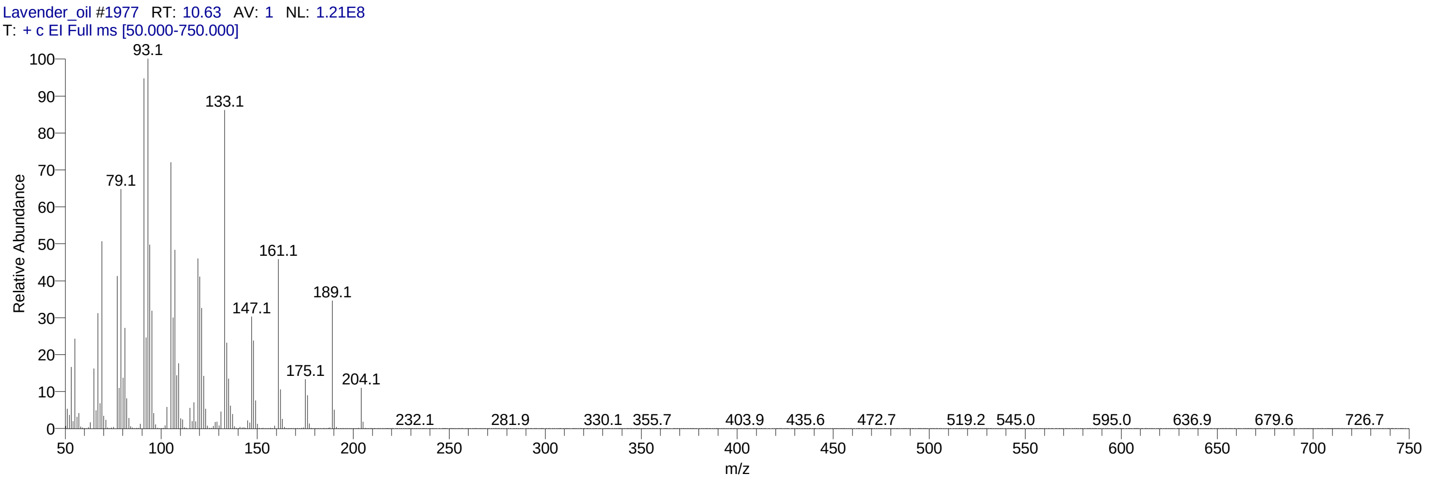


| RT | Compound Name | Area % | MF | Molecular Formula | Molecular Weight | Cas # | Library |
|---|---|---|---|---|---|---|---|
| 10.63 | Caryophyllene | 16.35 | 933 | C15H24 | 204 | 87-44-5 | mainlib |
| 10.63 | BICYCLO[7.2.0]UNDEC-4-ENE, 4,11,11-TRIMETHYL-8-METHYLENE-, [1R-(1R*,4E,9S*)]- | 16.35 | 930 | C15H24 | 204 | 87-44-5 | WileyRegistry8e |
| 10.63 | Isocaryophillene | 16.35 | 919 | C15H24 | 204 | NA | mainlib |
| 10.63 | BICYCLO[7.2.0]UNDEC-4-ENE, 4,11,11-TRIMETHYL-8-METHYLENE-, [1R-(1R*,4E,9S*)]- | 16.35 | 919 | C15H24 | 204 | 87-44-5 | WileyRegistry8e |
| 10.63 | Bicyclo[7.2.0]undec-4-ene, 4,11,11-trimethyl-8-methylene-,[1R-(1R*,4Z,9S*)]- | 16.35 | 915 | C15H24 | 204 | 118-65-0 | replib |

Compound Structure

Hit Spectrum

Caryophyllene
Formula C15H24, MW 204, CAS# 87-44-5, Entry# 66572
Bicyclo[7.2.0]undec-4-ene, 4,11,11-trimethyl-8-methylene-, [1R-(1R*,4E,9S*)]-

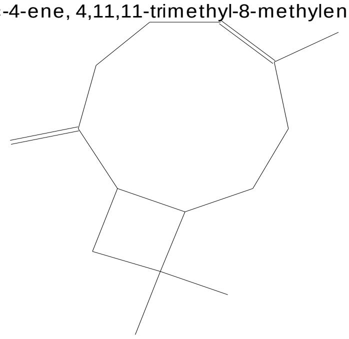

SI 933, RSI 933, mainlib, Entry# 66572, CAS# 87-44-5, Caryophyllene

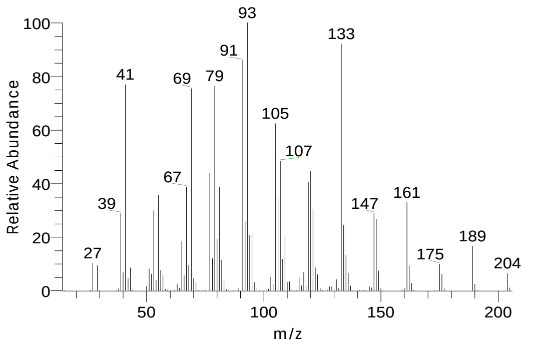


BICYCLO[7.2.0]UNDEC-4-ENE, 4,11,11-TRIMETHYL-8-METHYLENE-, [1R-(1R*,4E,9S*)]-
Formula C15H24, MW 204, CAS# 87-44-5, Entry# 89162
2,6,10,10-TETRAMETHYLBICYCLO[7.2.0]UNDECA-1,6-DIENE #

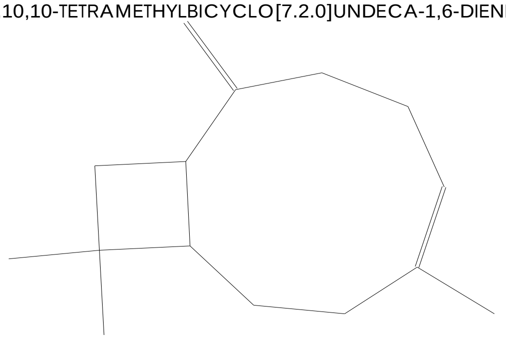

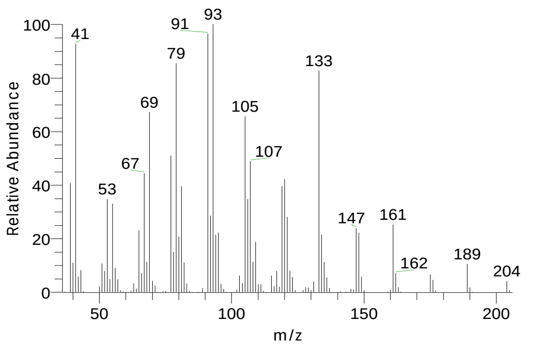

# *My GC-MS Report*

Compound Structure | Hit Spectrum

Isocaryophillene
Formula C15H24, MW 204, CAS# NA, Entry# 65988
4,11,11-Trimethyl-8-methylenebicyclo[7.2.0]undec-4-ene #

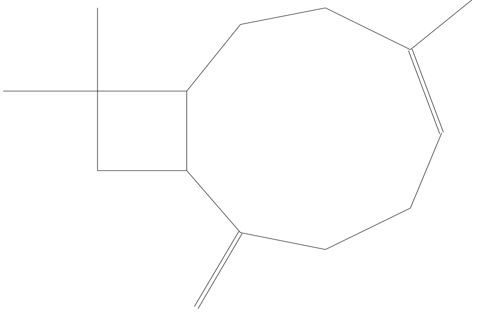

SI 915, RSI 919, mainlib, Entry# 65988, CAS# NA, Isocaryophillene

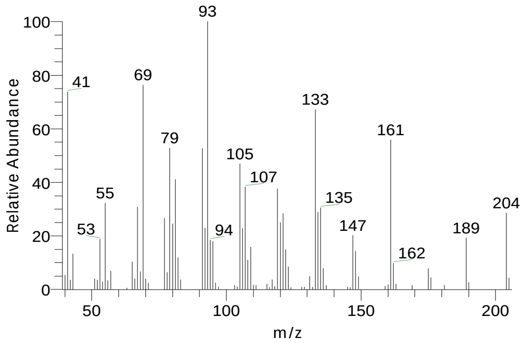


BICYCLO[7.2.0]UNDEC-4-ENE, 4,11,11-TRIMETHYL-8-METHYLENE-, [1R-(1R*,4E,9S*)]-
Formula C15H24, MW 204, CAS# 87-44-5, Entry# 89170
2,6,10,10-TETRAMETHYLBICYCLO[7.2.0]UNDECA-1,6-DIENE #

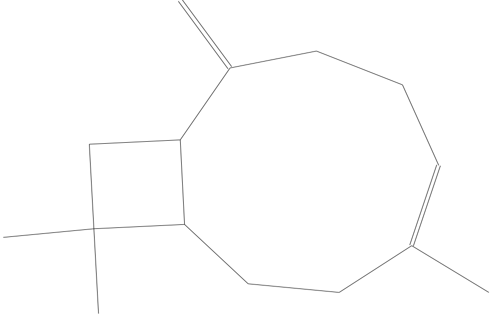

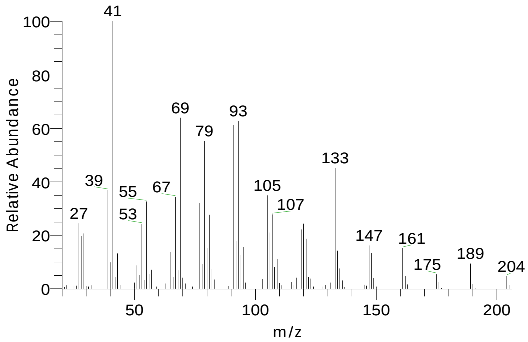


Bicyclo[7.2.0]undec-4-ene, 4,11,11-trimethyl-8-methylene-,[1R-(1R*,4Z,9S*)]-
Formula C15H24, MW 204, CAS# 118-65-0, Entry# 1329
Isocaryophyllene

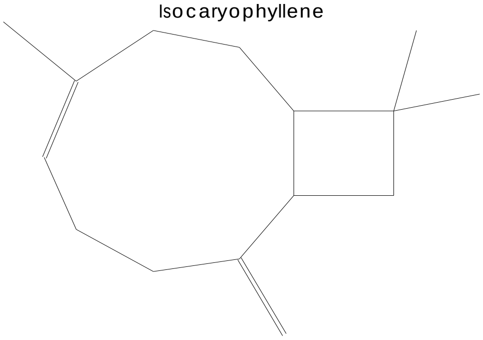

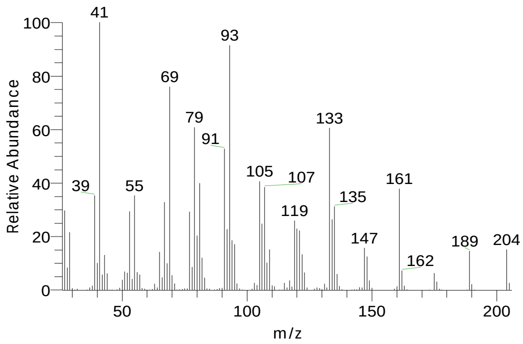


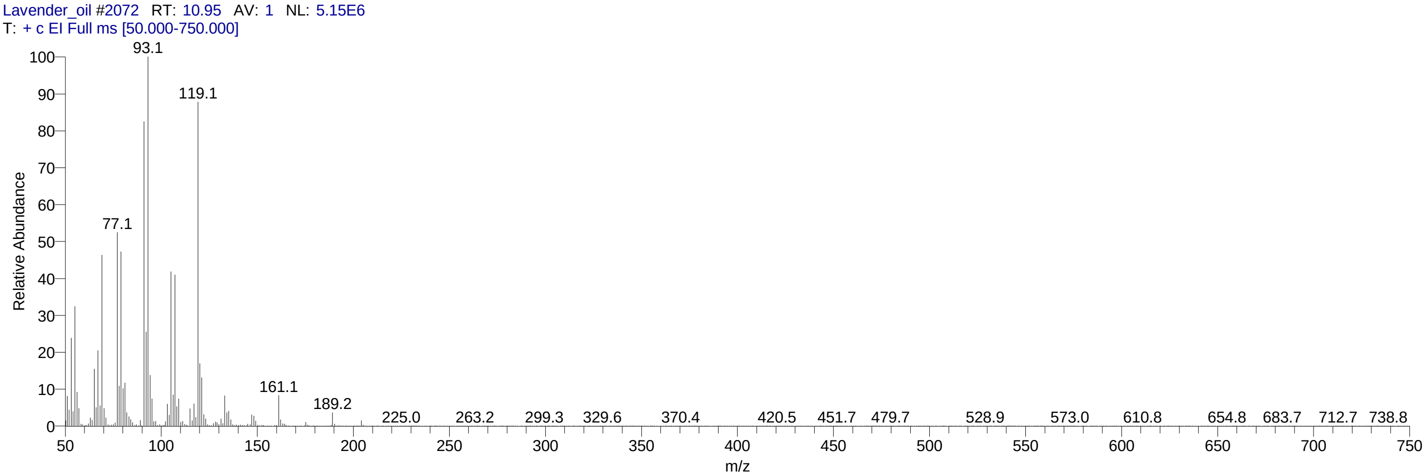


| RT | Compound Name | Area % | MF | Molecular Formula | Molecular Weight | Cas # | Library |
|---|---|---|---|---|---|---|---|
| 10.95 | Bicyclo[3.1.1]hept-2-ene, 2,6-dimethyl-6-(4-methyl-3-pentenyl)- | 0.26 | 958 | C15H24 | 204 | 17699-05-7 | mainlib |
| 10.95 | trans-à-Bergamotene | 0.26 | 954 | C15H24 | 204 | 13474-59-4 | replib |

# *My GC-MS Report*

| RT | Compound Name | Area % | MF | Molecular Formula | Molecular Weight | Cas # | Library |
|---|---|---|---|---|---|---|---|
| 10.95 | 1,3,6,10-Dodecatetraene, 3,7,11-trimethyl-, (Z,E)- | 0.26 | 920 | C15H24 | 204 | 26560-14-5 | mainlib |
| 10.95 | 1,3,6,10-Dodecatetraene, 3,7,11-trimethyl-, (Z,E)- | 0.26 | 937 | C15H24 | 204 | 26560-14-5 | replib |
| 10.95 | cis-à-Bergamotene | 0.26 | 908 | C15H24 | 204 | 18252-46-5 | mainlib |

Compound Structure | Hit Spectrum

Bicyclo[3.1.1]hept-2-ene, 2,6-dimethyl-6-(4-methyl-3-pentenyl)-
Formula C15H24, MW 204, CAS# 17699-05-7, Entry# 65801
2-Norpinene, 2,6-dimethyl-6-(4-methyl-3-pentenyl)-

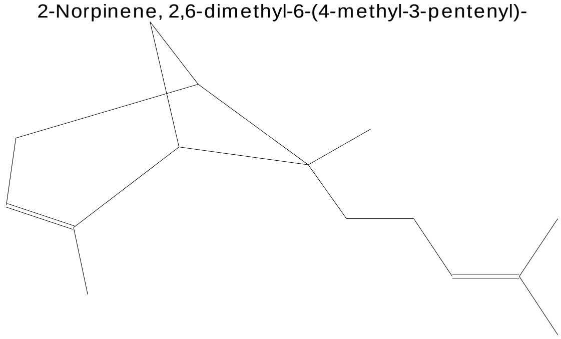

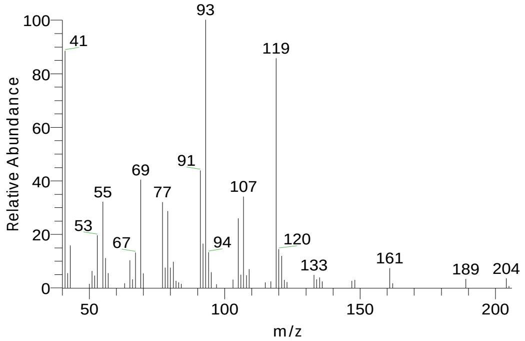


trans-à-Bergamotene
Formula C15H24, MW 204, CAS# 13474-59-4, Entry# 14007
2,6-Dimethyl-6-(4-methyl-3-pentenyl)bicyclo[3.1.1]hept-2-ene #

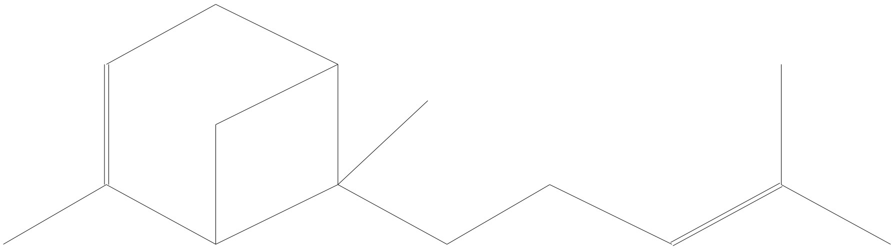

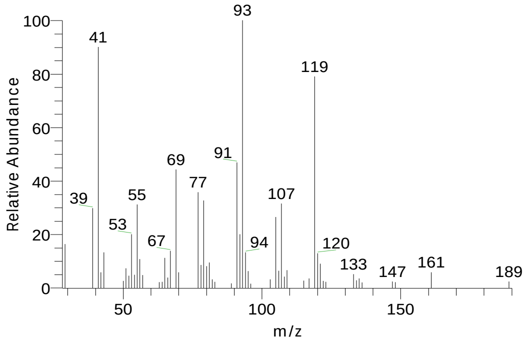


1,3,6,10-Dodecatetraene, 3,7,11-trimethyl-, (Z,E)-
Formula C15H24, MW 204, CAS# 26560-14-5, Entry# 3802
(Z,E)-à-Farnesene

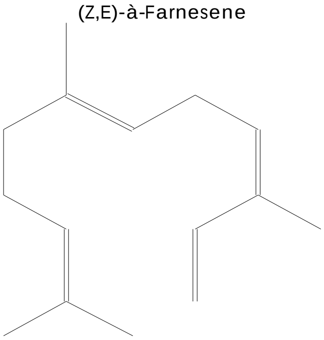

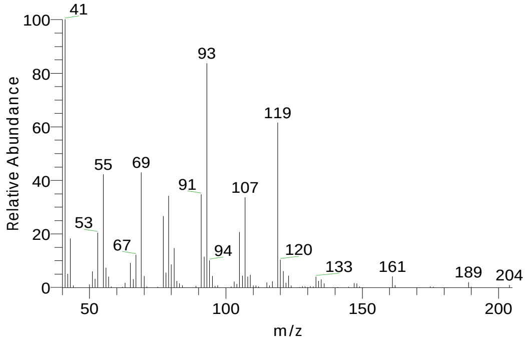


1,3,6,10-Dodecatetraene, 3,7,11-trimethyl-, (Z,E)-
Formula C15H24, MW 204, CAS# 26560-14-5, Entry# 1332
(Z,E)-à-Farnesene

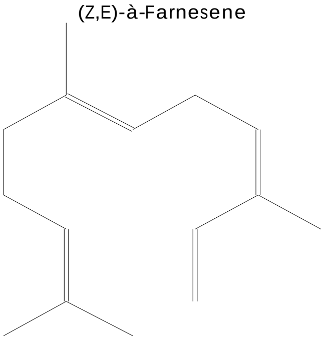

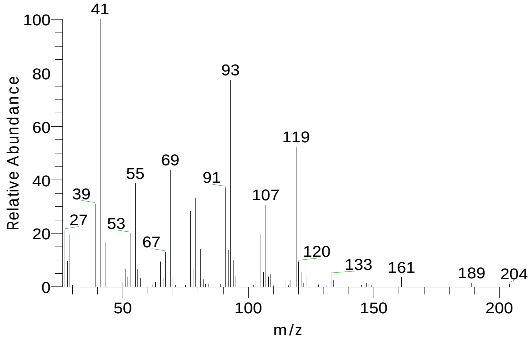

# My GC-MS Report

Compound Structure | Hit Spectrum

cis-à-Bergamotene
Formula C15H24, MW 204, CAS# 18252-46-5, Entry# 99916
(1R,5R,6R)-2,6-Dimethyl-6-(4-methylpent-3-en-1-yl)bicyclo[3.1.1]hept-2-ene

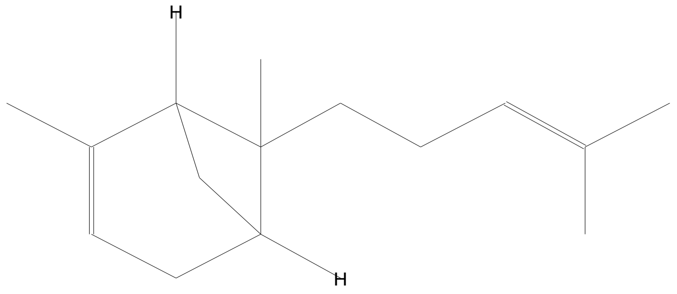


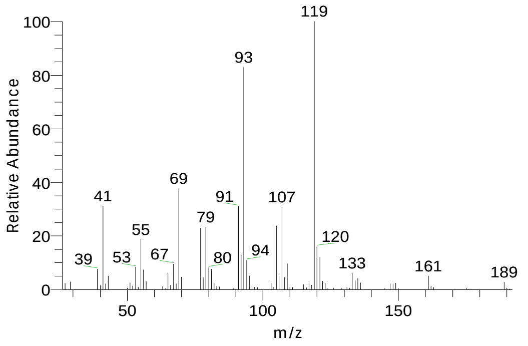


Lavender_oil #2277 RT: 11.63 AV: 1 NL: 2.05E8
T: + c EI Full ms [50.000-750.000]

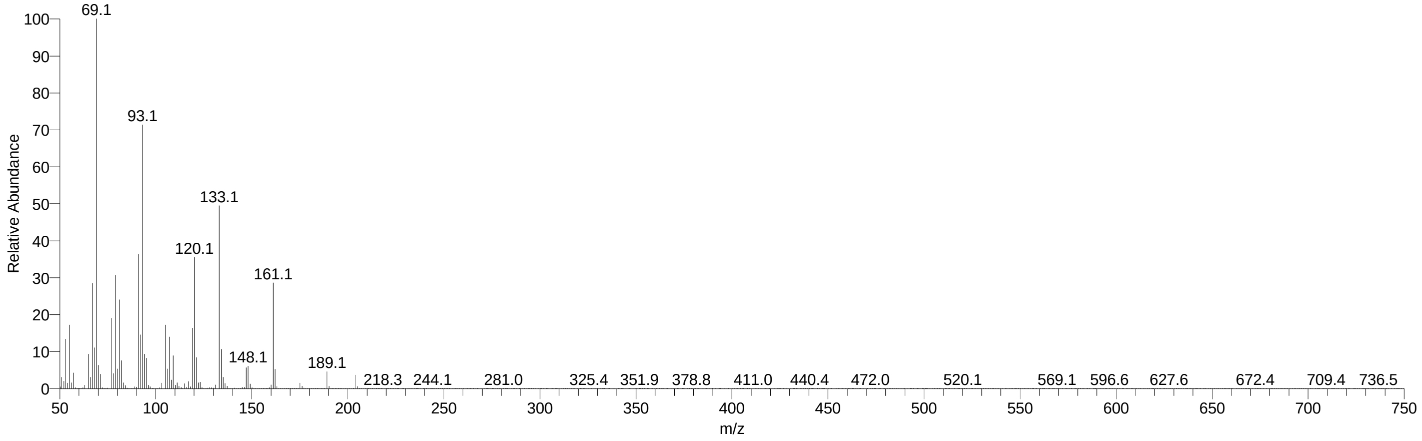


| RT | Compound Name | Area % | MF | Molecular Formula | Molecular Weight | Cas # | Library |
|---|---|---|---|---|---|---|---|
| 11.63 | cis-á-Farnesene | 14.09 | 938 | C15H24 | 204 | 28973-97-9 | mainlib |
| 11.63 | (E)-á-Famesene | 14.09 | 931 | C15H24 | 204 | 18794-84-8 | mainlib |
| 11.63 | (E)-á-Famesene | 14.09 | 943 | C15H24 | 204 | 18794-84-8 | replib |
| 11.63 | 1,6,10-DODECATRIENE, 7,11-DIMETHYL-3-METHYLENE-, (E)- | 14.09 | 941 | C15H24 | 204 | 502-60-3 | WileyRegistry8e |
| 11.63 | Bicyclo[7.2.0]undec-4-ene, 4,11,11-trimethyl-8-methylene-,[1R-(1R*,4Z,9S*)]- | 14.09 | 891 | C15H24 | 204 | 118-65-0 | replib |

Compound Structure | Hit Spectrum

cis-á-Farnesene
Formula C15H24, MW 204, CAS# 28973-97-9, Entry# 3453
1,6,10-Dodecatriene, 7,11-dimethyl-3-methylene-, (Z)-

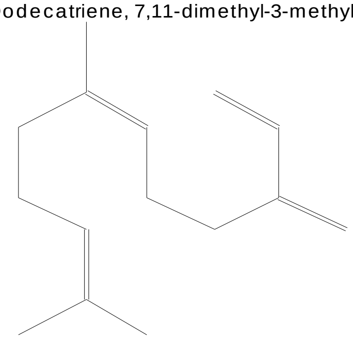

SI 935, RSI 938, mainlib, Entry# 3453, CAS# 28973-97-9, cis-á-Farnesene

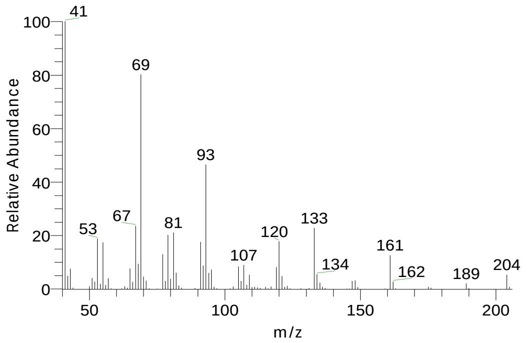

# *My GC-MS Report*

Compound Structure | Hit Spectrum

(E)-á-Famesene
Formula C15H24, MW 204, CAS# 18794-84-8, Entry# 3451
1,6,10-Dodecatriene, 7,11-dimethyl-3-methylene-, (E)-

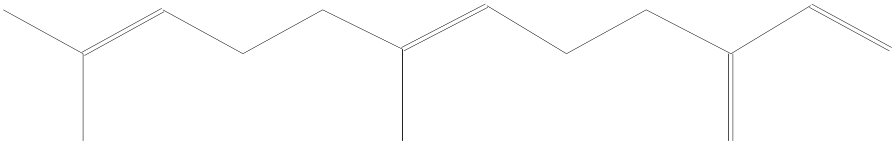

SI 928, RSI 931, mainlib, Entry# 3451, CAS# 18794-84-8, (E)-á-Famesene

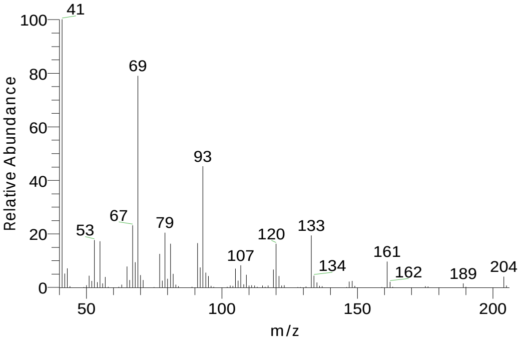


(E)-á-Famesene
Formula C15H24, MW 204, CAS# 18794-84-8, Entry# 8532
1,6,10-Dodecatriene, 7,11-dimethyl-3-methylene-, (E)-

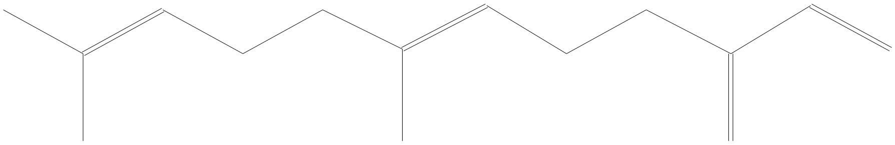

SI 920, RSI 943, replib, Entry# 8532, CAS# 18794-84-8, (E)-á-Famesene

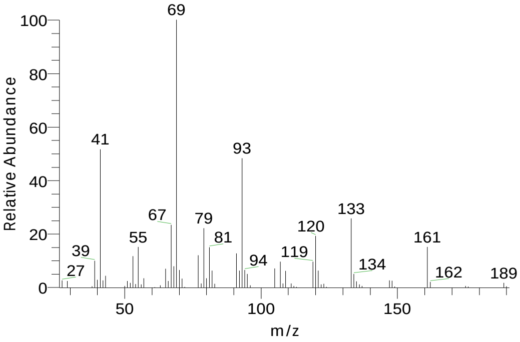


1,6,10-DODECATRIENE, 7,11-DIMETHYL-3-METHYLENE-, (E)-
Formula C15H24, MW 204, CAS# 502-60-3, Entry# 89037
(E)-á-FARNESENE

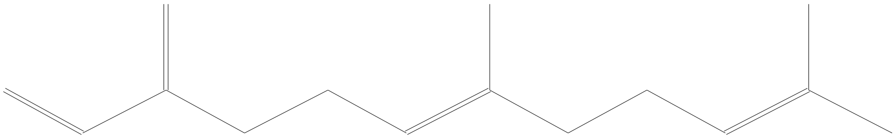

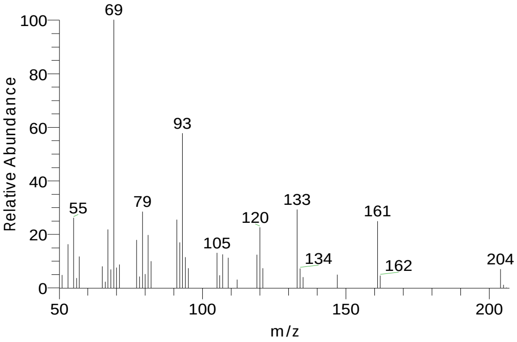


Bicyclo[7.2.0]undec-4-ene, 4,11,11-trimethyl-8-methylene-,[1R-(1R*,4Z,9S*)]-
Formula C15H24, MW 204, CAS# 118-65-0, Entry# 1245
Isocaryophyllene

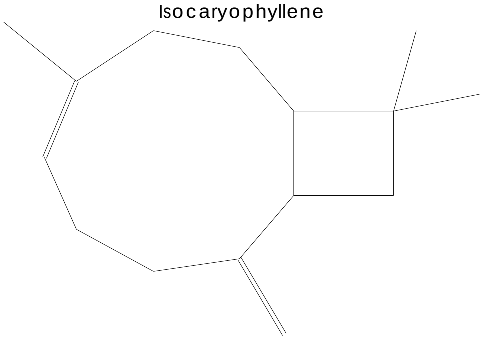

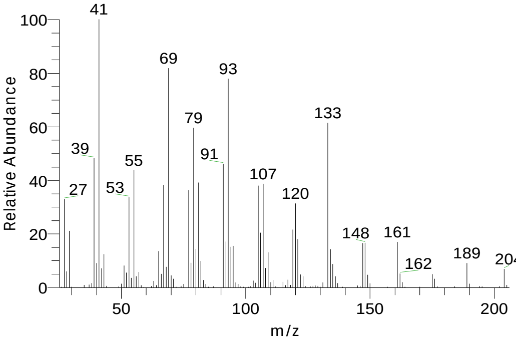

# My GC-MS Report

Lavender_oil #2389 RT: 12.01 AV: 1 NL: 3.18E7
T: + c EI Full ms [50.000-750.000]

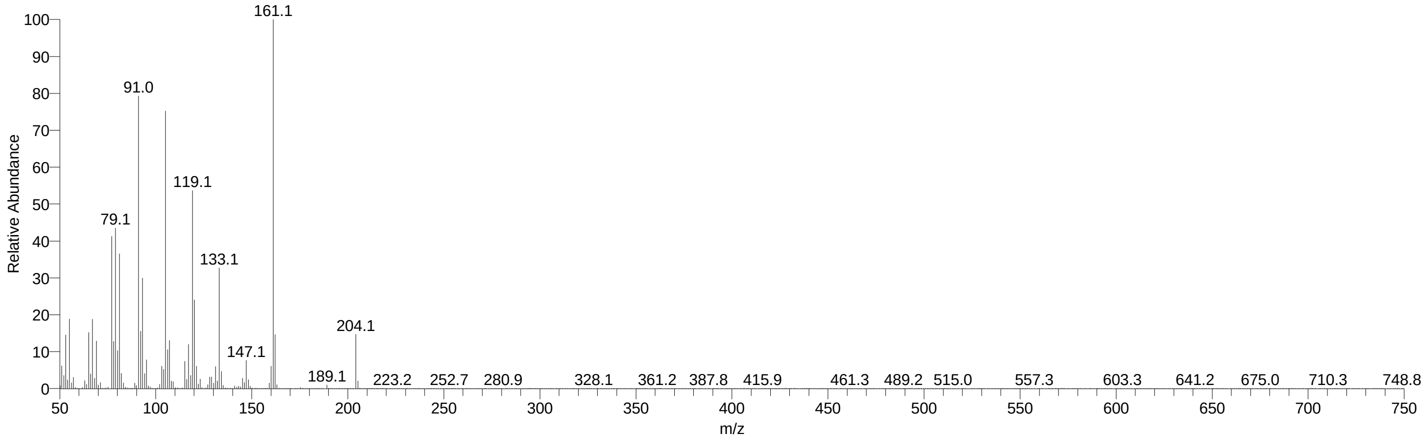


| RT | Compound Name | Area % | MF | Molecular Formula | Molecular Weight | Cas # | Library |
|---|---|---|---|---|---|---|---|
| 12.01 | 1,6-CYCLODECADIENE, 1-METHYL-5-METHYLENE-8-(1-METHYLETHYL)-, [S-(E,E)]- | 1.29 | 961 | C15H24 | 204 | 23986-74-5 | WileyRegistry8e |
| 12.01 | 1,6-CYCLODECADIENE, 1-METHYL-5-METHYLENE-8-(1-METHYLETHYL)-, [S-(E,E)]- | 1.29 | 948 | C15H24 | 204 | 23986-74-5 | WileyRegistry8e |
| 12.01 | Germacrene D | 1.29 | 949 | C15H24 | 204 | 23986-74-5 | replib |
| 12.01 | Germacrene D | 1.29 | 951 | C15H24 | 204 | 23986-74-5 | mainlib |
| 12.01 | 1,6-CYCLODECADIENE, 1-METHYL-5-METHYLENE-8-(1-METHYLETHYL)-, [S-(E,E)]- | 1.29 | 951 | C15H24 | 204 | 23986-74-5 | WileyRegistry8e |

Compound Structure | Hit Spectrum

1,6-CYCLODECADIENE, 1-METHYL-5-METHYLENE-8-(1-METHYLETHYL)-, [S-(E,E)]-
Formula C15H24, MW 204, CAS# 23986-74-5, Entry# 89578
GERMACRA-1(10),4(15),5-TRIENE, (-)-

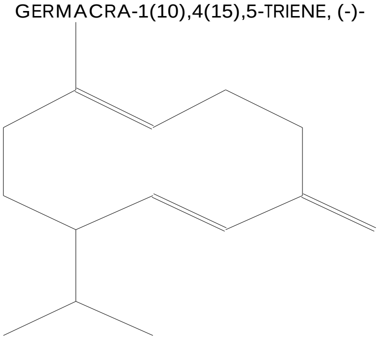

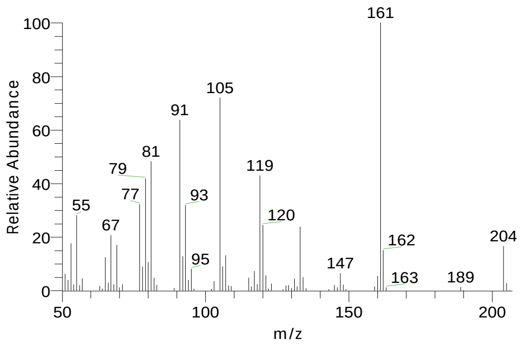


1,6-CYCLODECADIENE, 1-METHYL-5-METHYLENE-8-(1-METHYLETHYL)-, [S-(E,E)]-
Formula C15H24, MW 204, CAS# 23986-74-5, Entry# 89560
GERMACRA-1(10),4(15),5-TRIENE, (-)-

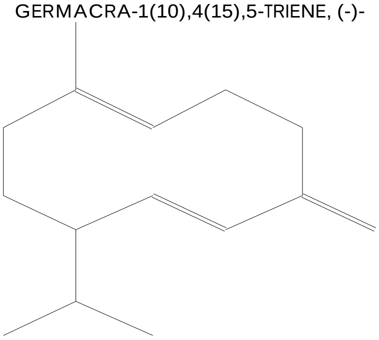

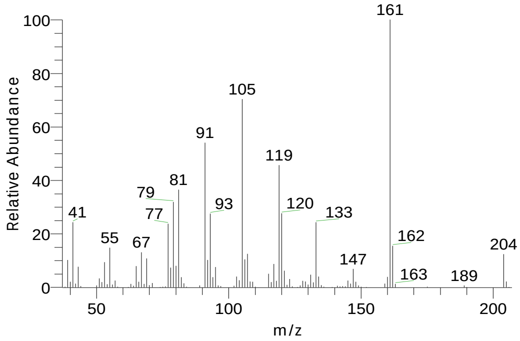

# *My GC-MS Report*

Compound Structure | Hit Spectrum

Germacrene D
Formula C15H24, MW 204, CAS# 23986-74-5, Entry# 24886
(S,1Z,6Z)-8-Isopropyl-1-methyl-5-methylenecyclodeca-1,6-diene

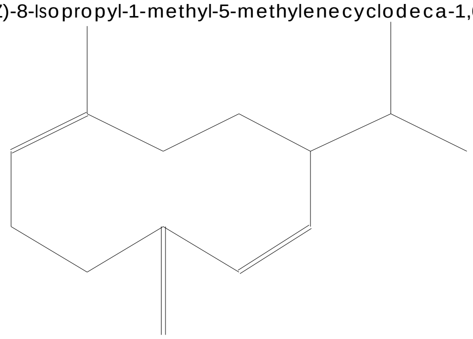

SI 934, RSI 949, replib, Entry# 24886, CAS# 23986-74-5, Germacrene D

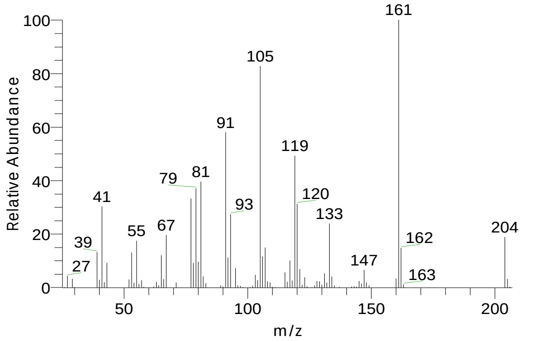


Germacrene D
Formula C15H24, MW 204, CAS# 23986-74-5, Entry# 150487
(S,1Z,6Z)-8-Isopropyl-1-methyl-5-methylenecyclodeca-1,6-diene

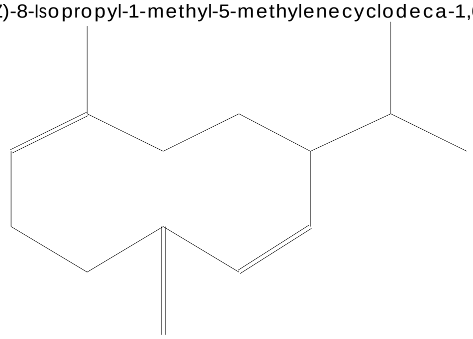

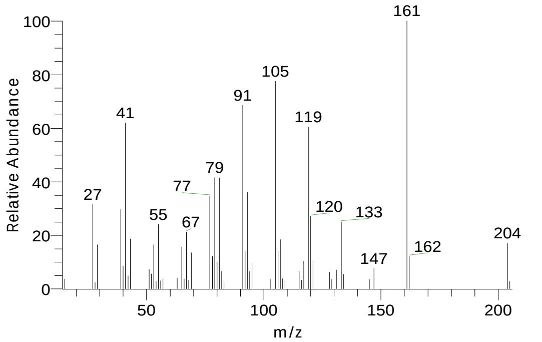


1,6-CYCLODECADIENE, 1-METHYL-5-METHYLENE-8-(1-METHYLETHYL)-, [S-(E,E)]-
Formula C15H24, MW 204, CAS# 23986-74-5, Entry# 362375
GERMACRA-1(10),4(15),5-TRIENE, (-)-

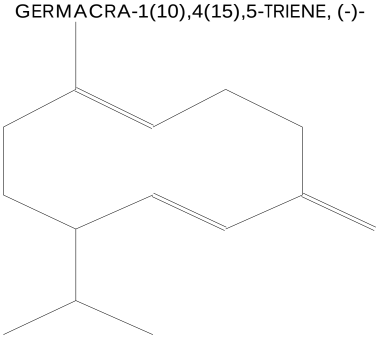

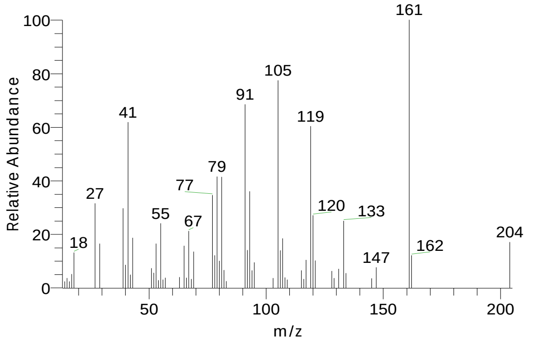


Lavender_oil #2614 RT: 12.76 AV: 1 NL: 5.43E6
T: + c EI Full ms [50.000-750.000]

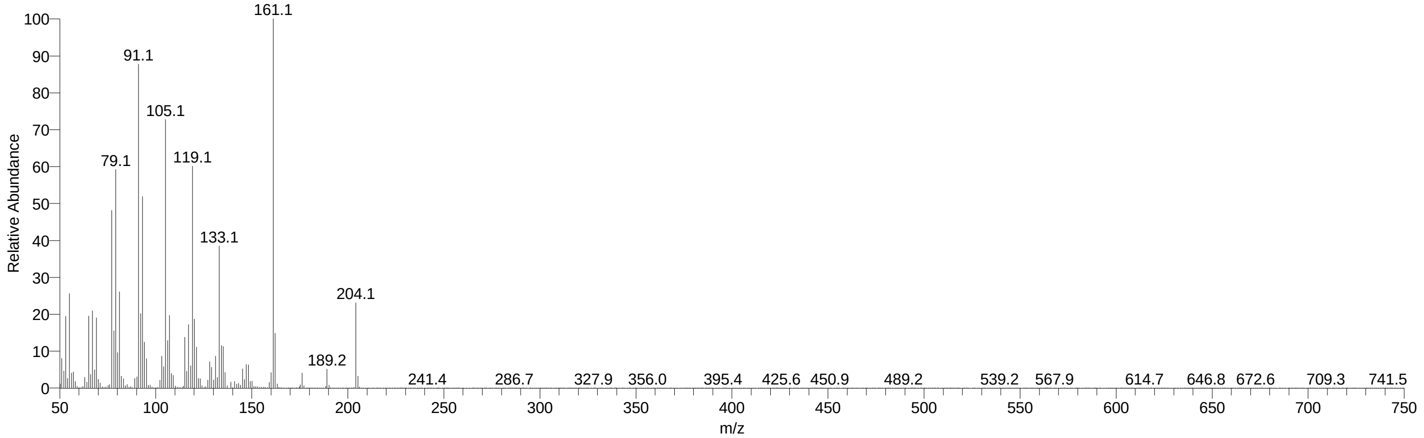


| RT | Compound Name | Area % | MF | Molecular Formula | Molecular Weight | Cas # | Library |
|---|---|---|---|---|---|---|---|
| 12.76 | Naphthalene, 1,2,3,4,4a,5,6,8a-octahydro-7-methyl-4-methylene-1-(1-methylethyl)-, (1à,4aá,8aà)- | 0.35 | 924 | C15H24 | 204 | 39029-41-9 | replib |

# My GC-MS Report

| RT | Compound Name | Area % | MF | Molecular Formula | Molecular Weight | Cas # | Library |
|---|---|---|---|---|---|---|---|
| 12.76 | ç-Muurolene | 0.35 | 927 | C15H24 | 204 | 30021-74-0 | replib |
| 12.76 | NAPHTHALENE, 1,2,3,4,4A,5,6,8A-OCTAHYDRO-7-METHYL-4-METHYLENE-1-(1-METHYLETHYL)-, (1à,4Aá,8Aà)- | 0.35 | 917 | C15H24 | 204 | 39029-41-9 | WileyRegistry8e |
| 12.76 | Naphthalene, 1,2,3,4,4a,5,6,8a-octahydro-7-methyl-4-methylene-1-(1-methylethyl)-, (1à,4aá,8aà)- | 0.35 | 915 | C15H24 | 204 | 39029-41-9 | replib |
| 12.76 | ç-Muurolene | 0.35 | 912 | C15H24 | 204 | 30021-74-0 | replib |

Compound Structure | Hit Spectrum

Formula C15H24, MW 204, CAS# 39029-41-9, Entry# 24895
ç-Cadinene

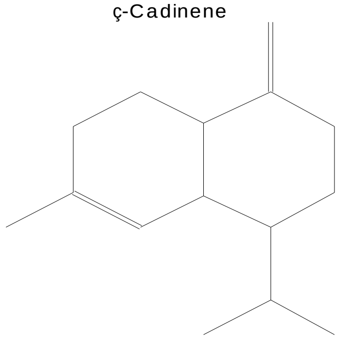

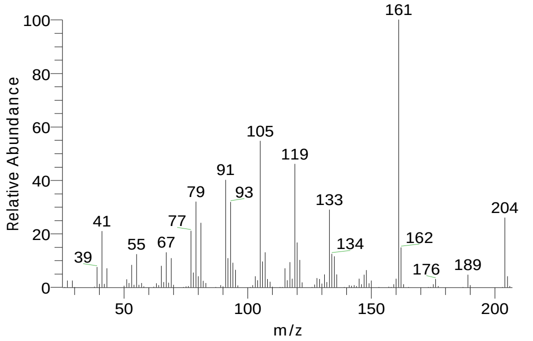


ç-Muurolene
Formula C15H24, MW 204, CAS# 30021-74-0, Entry# 24843

SI 916, RSI 927, replib, Entry# 24843, CAS# 30021-74-0, ç-Muurolene

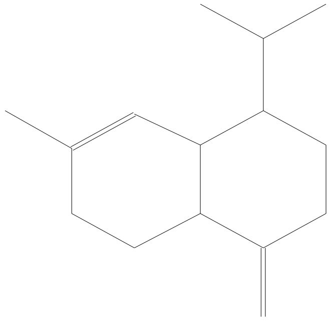

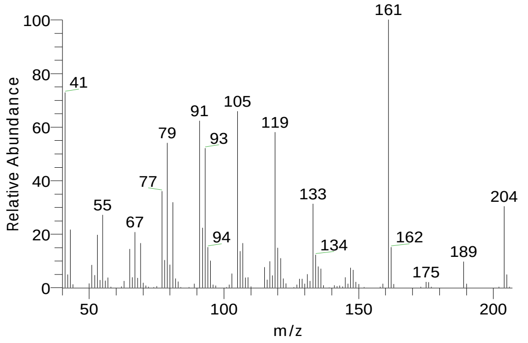


Formula C15H24, MW 204, CAS# 39029-41-9, Entry# 89268
1-ISOPROPYL-7-METHYL-4-METHYLENE-1,2,3,4,4A,5,6,8A-OCTAHYDRONAPHTHALENE #

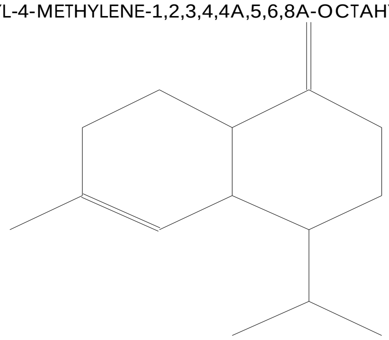

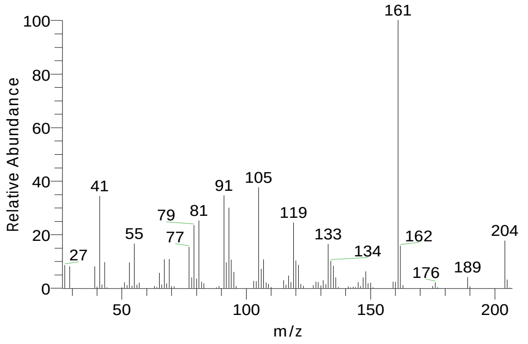

# *My GC-MS Report*

Compound Structure | Hit Spectrum

Formula C15H24, MW 204, CAS# 39029-41-9, Entry# 24890
ç-Cadinene

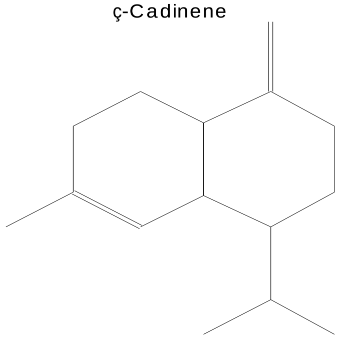

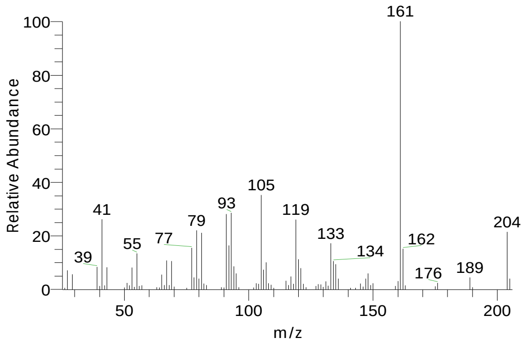


ç-Muurolene
Formula C15H24, MW 204, CAS# 30021-74-0, Entry# 24906

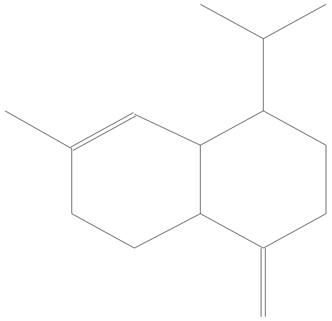

SI 906, RSI 912, replib, Entry# 24906, CAS# 30021-74-0, ç-Muurolene

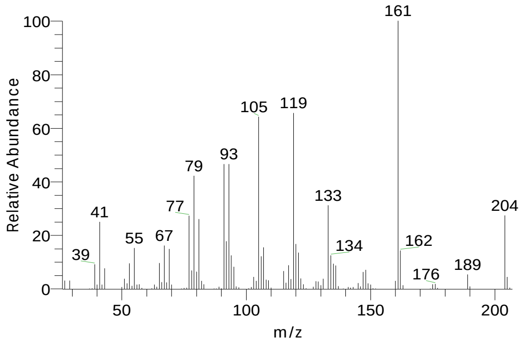


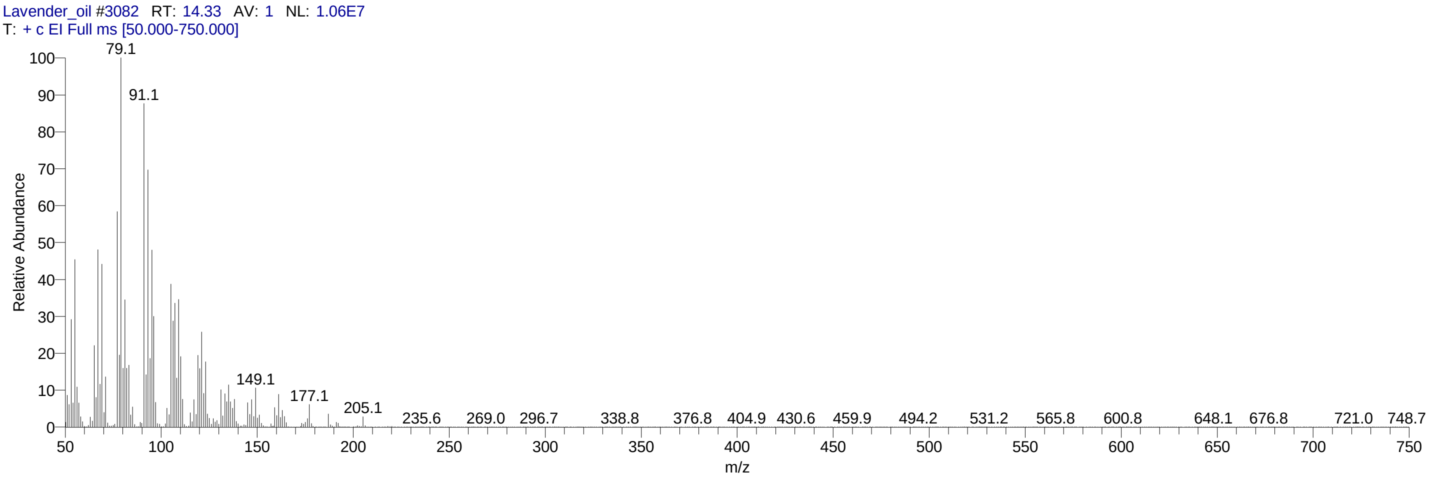


| RT | Compound Name | Area % | MF | Molecular Formula | Molecular Weight | Cas # | Library |
|---|---|---|---|---|---|---|---|
| 14.33 | (-)-5-OXATRICYCLO[8.2.0.0(4,6)] DODECANE,,12-TRIMETHYL-9-M ETHYLENE-, [1R-(1R*,4R*,6R*,10S*)]- | 0.77 | 940 | C15H24O | 220 | 1139-3 0-6 | WileyRegi stry8e |
| 14.33 | (-)-5-OXATRICYCLO[8.2.0.0(4,6)] DODECANE,,12-TRIMETHYL-9-M ETHYLENE-, [1R-(1R*,4R*,6R*,10S*)]- | 0.77 | 929 | C15H24O | 220 | 1139-3 0-6 | WileyRegi stry8e |
| 14.33 | (-)-5-OXATRICYCLO[8.2.0.0(4,6)] DODECANE,,12-TRIMETHYL-9-M ETHYLENE-, [1R-(1R*,4R*,6R*,10S*)]- | 0.77 | 926 | C15H24O | 220 | 1139-3 0-6 | WileyRegi stry8e |
| 14.33 | Caryophyllene oxide | 0.77 | 913 | C15H24O | 220 | 1139-3 0-6 | mainlib |
| 14.33 | 4,12,12-TRIMETHYL-9-METHYLE NE-5-OXATRICYCLO[8.2.0.0~4,6 ~]DODECANE | 0.77 | 913 | C15H24O | 220 | NA | WileyRegi stry8e |

# *My GC-MS Report*

Compound Structure | Hit Spectrum

Formula C15H24O, MW 220, CAS# 1139-30-6, Entry# 107245
5-OXATRICYCLO[8.2.0.04,6]DODECANE, 4,12,12-TRIMETHYL-9-METHYLENE-, (1R,4R,6R,10S)-

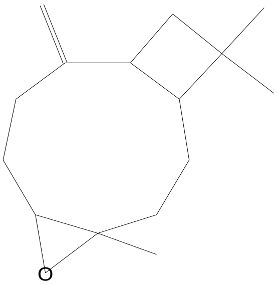


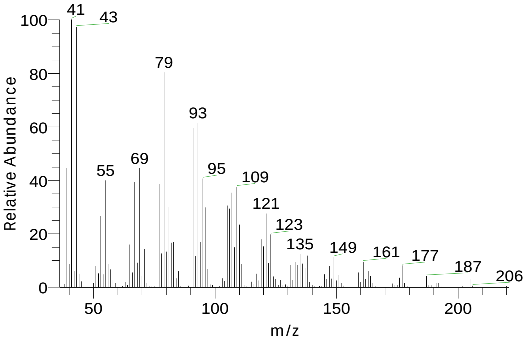


Formula C15H24O, MW 220, CAS# 1139-30-6, Entry# 107244
5-OXATRICYCLO[8.2.0.04,6]DODECANE, 4,12,12-TRIMETHYL-9-METHYLENE-, (1R,4R,6R,10S)-

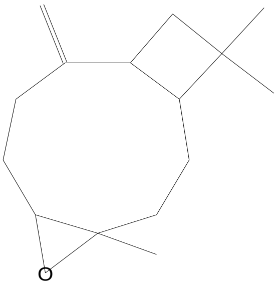


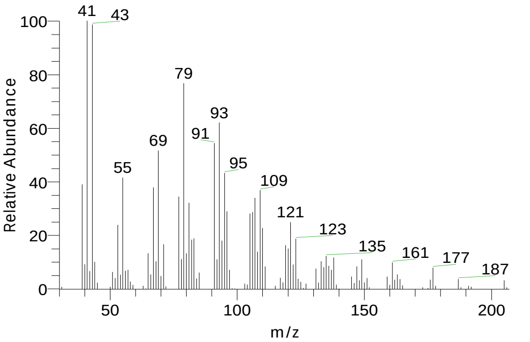


Formula C15H24O, MW 220, CAS# 1139-30-6, Entry# 107246
5-OXATRICYCLO[8.2.0.04,6]DODECANE, 4,12,12-TRIMETHYL-9-METHYLENE-, (1R,4R,6R,10S)-

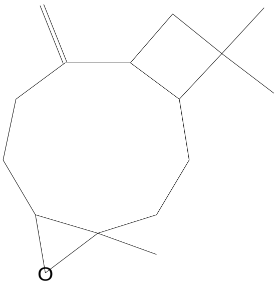


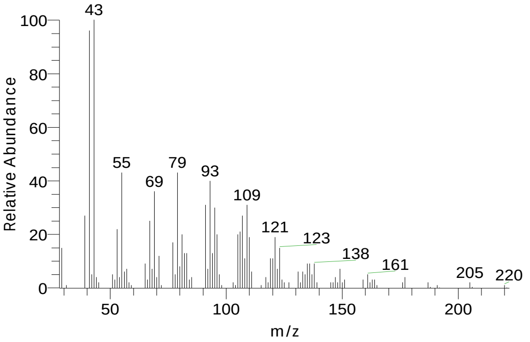


Caryophyllene oxide
Formula C15H24O, MW 220, CAS# 1139-30-6, Entry# 6247

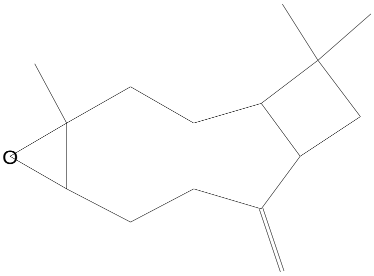


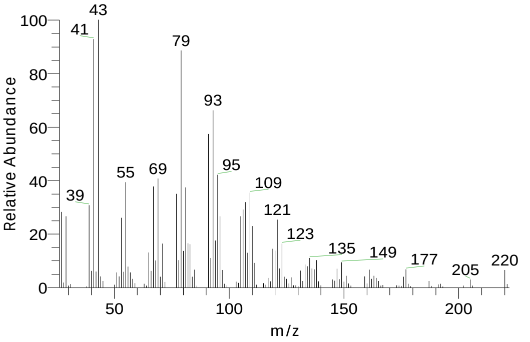

# My GC-MS Report

Compound Structure

4,12,12-TRIMETHYL-9-METHYLENE-5-OXATRICYCLO[8.2.0.0~4,6~]DODECANE
Formula C15H24O, MW 220, CAS# NA, Entry# 388109
CARYOPHYLLENOXID

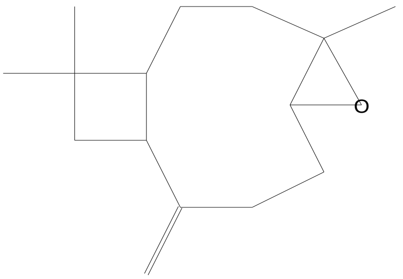


Hit Spectrum

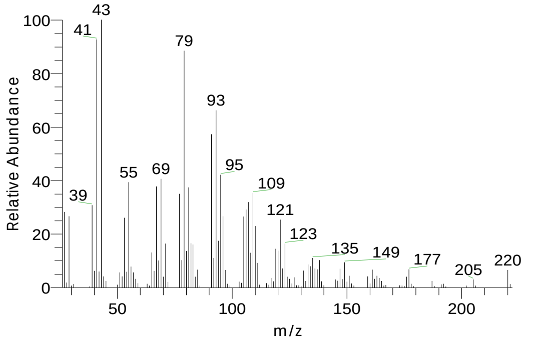

Supplement: Supplementary data 1 [file mmc1.pdf]
