# Supplementary material for: Larvicidal and adulticidal activity of essential oils from plants of the Lamiaceae family against the West Nile virus vector, Culex pipiens (Diptera: Culicidae)
Source: Saudi J Biol Sci. 2022 Jun 16;29(8):103350. doi: 10.1016/j.sjbs.2022.103350 (PMC9232543; doi:10.1016/j.sjbs.2022.103350)

My GC-MS Report

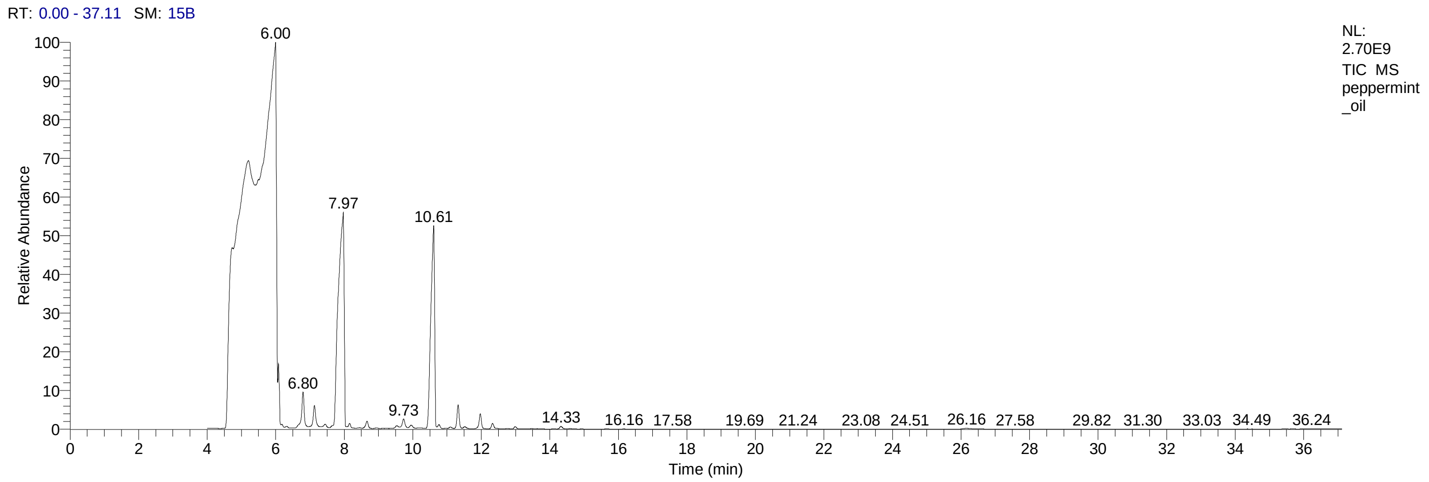

RT	Area %	Peak Area	Peak Height
4.68	8.42	4063067884.30	501024703.52
4.88	0.24	114931501.35	29943849.73
5.19	4.91	2368487049.80	192050196.78
6.01	26.41	12739183474.35	1988600551.22
6.09	1.63	785413160.64	372819888.67
6.80	1.94	937648855.46	262990672.27
7.13	1.32	634605734.41	158927031.10
7.98	32.76	15802629814.66	1448062177.76
8.16	0.21	102338294.49	34126440.78
8.67	0.46	223501444.15	52989470.49
9.73	0.58	279985605.09	63142981.17
10.62	18.48	8915803583.91	1374122019.40
11.32	1.41	680176010.03	181532778.35
11.97	0.88	423518845.05	110511938.85
12.33	0.34	163893944.46	39601044.18

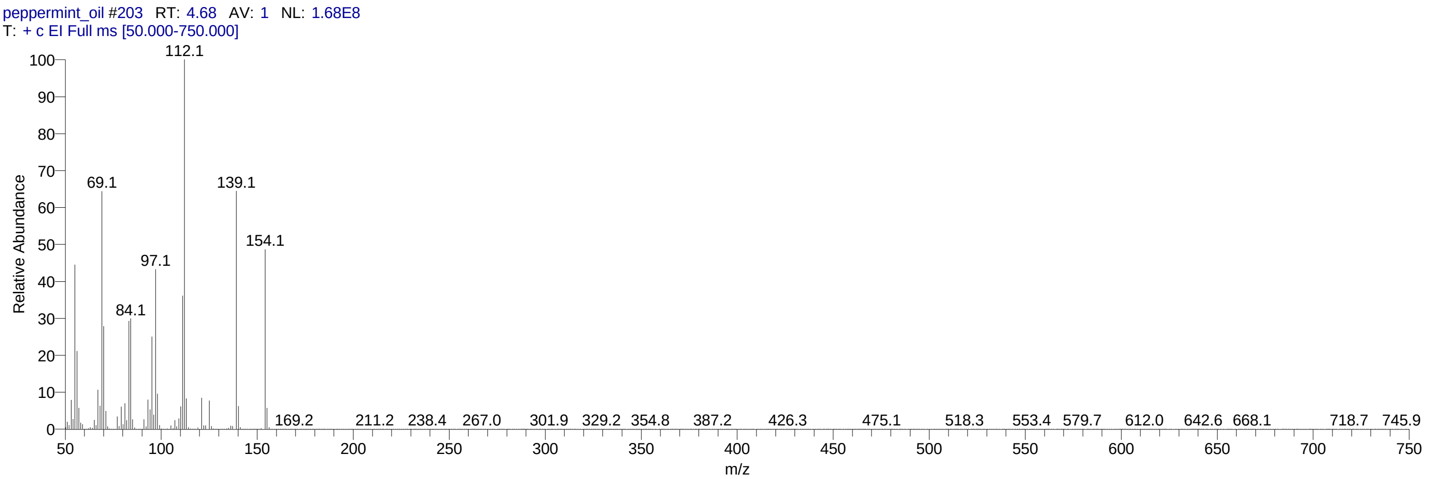

RT	Compound Name	Area %	MF	Molecular Formula	Molecular Weight	Cas #	Library
4.68	Cyclohexanone, 5-methyl-2-(1-methylethyl)-, cis-	8.42	945	C10H18O	154	491-07-6	replib
4.68	l-Menthone	8.42	955	C10H18O	154	14073-97-3	mainlib

My GC-MS Report

RT	Compound Name	Area %	MF	Molecular Formula	Molecular Weight	Cas #	Library
4.68	CYCLOHEXANONE, 5-METHYL-2-(1-METHYLETHYL)-, TRANS-	8.42	935	C10H18O	154	89-80-5	WileyRegistry8e
4.68	CYCLOHEXANONE, 5-METHYL-2-(1-METHYLETHYL)-, CIS-	8.42	936	C10H18O	154	491-07-6	WileyRegistry8e
4.68	Cyclohexanone, 5-methyl-2-(1-methylethyl)-, cis-	8.42	941	C10H18O	154	491-07-6	replib

Compound Structure	Hit Spectrum

Cyclohexanone, 5-methyl-2-(1-methylethyl)-, cis-
Formula C10H18O, MW 154, CAS# 491-07-6, Entry# 17708
p-Menthan-3-one, cis-

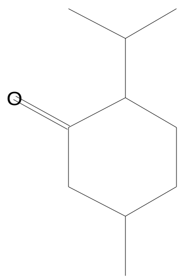

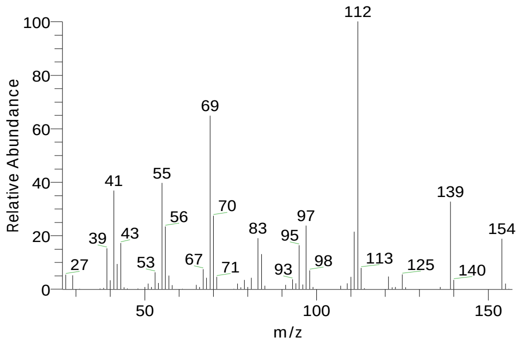

SI 935, RSI 955, mainlib, Entry# 89414, CAS# 14073-97-3, l-Menthone

l-Menthone
Formula C10H18O, MW 154, CAS# 14073-97-3, Entry# 89414
Cyclohexanone, 5-methyl-2-(1-methylethyl)-, (2S-trans)-

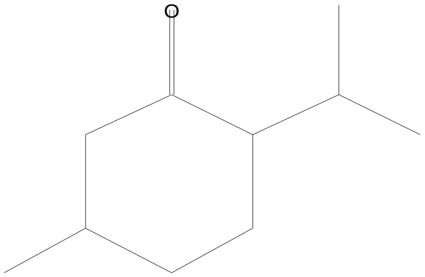

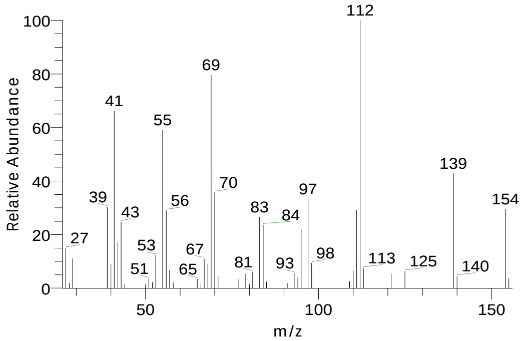

CYCLOHEXANONE, 5-METHYL-2-(1-METHYLETHYL)-, TRANS-
Formula C10H18O, MW 154, CAS# 89-80-5, Entry# 38733
2-ISOPROPYL-5-METHYLCYCLOHEXANONE #

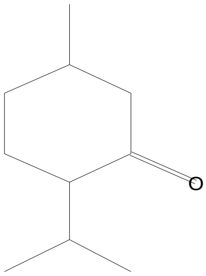

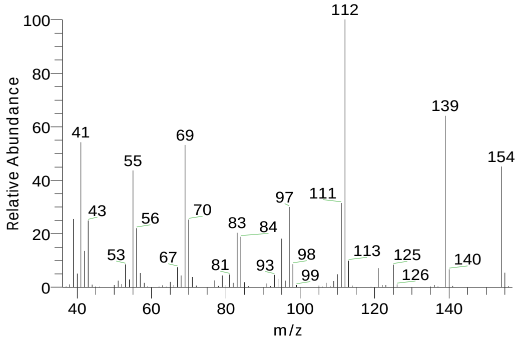

CYCLOHEXANONE, 5-METHYL-2-(1-METHYLETHYL)-, CIS-
Formula C10H18O, MW 154, CAS# 491-07-6, Entry# 38412
(1)-ISOMENTHONE

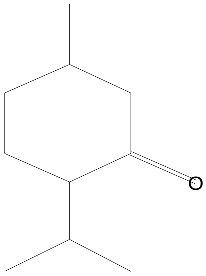

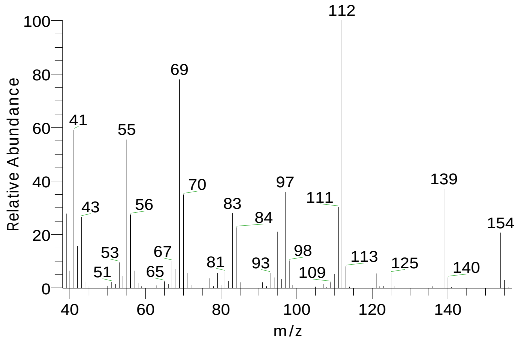

My GC-MS Report

Compound Structure	Hit Spectrum

Cyclohexanone, 5-methyl-2-(1-methylethyl)-, cis-
Formula C10H18O, MW 154, CAS# 491-07-6, Entry# 1370
p-Menthan-3-one, cis-

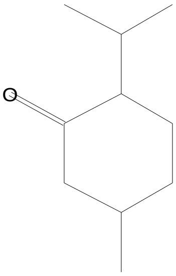

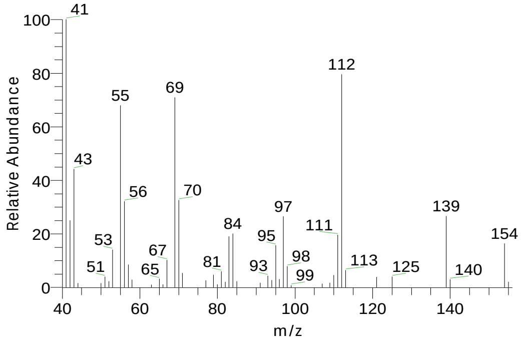

peppermint_oil #264 RT: 4.88 AV: 1 NL: 2.02E8
T: + c EI Full ms [50.000-750.000]

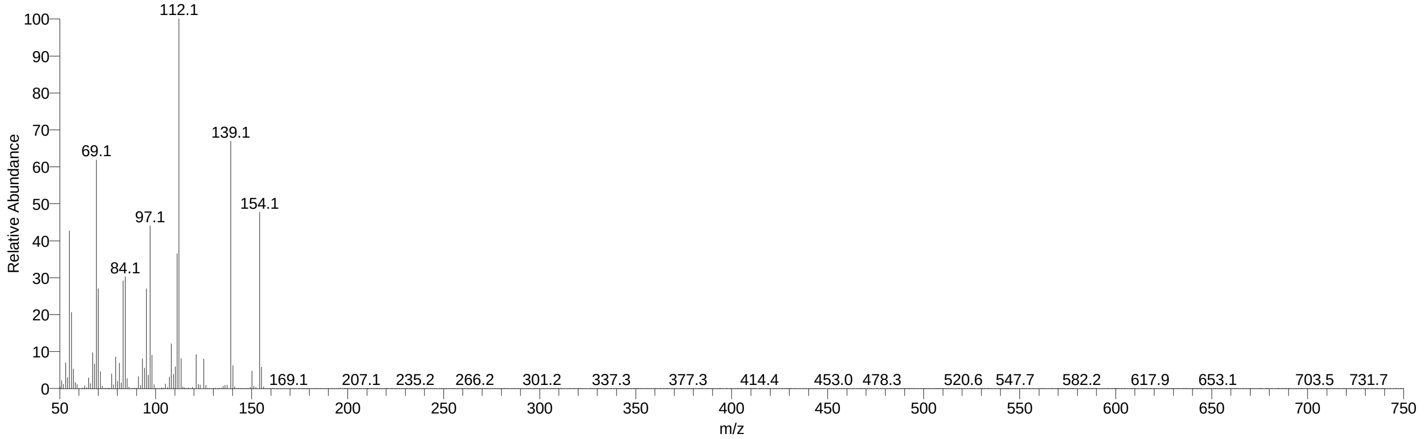

RT	Compound Name	Area %	MF	Molecular Formula	Molecular Weight	Cas #	Library
4.88	l-Menthone	0.24	956	C10H18O	154	14073-97-3	mainlib
4.88	Cyclohexanone, 5-methyl-2-(1-methylethyl)-, cis-	0.24	937	C10H18O	154	491-07-6	replib
4.88	CYCLOHEXANONE, 5-METHYL-2-(1-METHYLETHYL)-, CIS-	0.24	915	C10H18O	154	491-07-6	WileyRegistry8e
4.88	CYCLOHEXANONE, 5-METHYL-2-(1-METHYLETHYL)-, TRANS-	0.24	908	C10H18O	154	89-80-5	WileyRegistry8e
4.88	CYCLOHEXANONE, 5-METHYL-2-(1-METHYLETHYL)-, TRANS-	0.24	904	C10H18O	154	89-80-5	WileyRegistry8e

Compound Structure	Hit Spectrum

l-Menthone
Formula C10H18O, MW 154, CAS# 14073-97-3, Entry# 89414
Cyclohexanone, 5-methyl-2-(1-methylethyl)-, (2S-trans)-

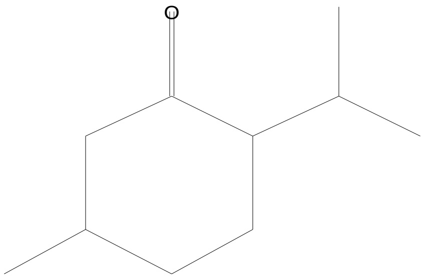

SI 917, RSI 956, mainlib, Entry# 89414, CAS# 14073-97-3, l-Menthone

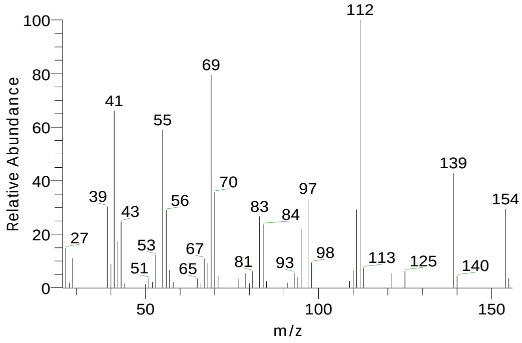

My GC-MS Report

Compound Structure | Hit Spectrum

Cyclohexanone, 5-methyl-2-(1-methylethyl)-, cis-
Formula C10H18O, MW 154, CAS# 491-07-6, Entry# 17708
p-Menthan-3-one, cis-

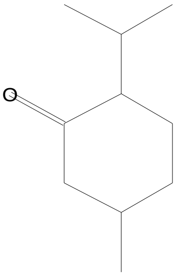

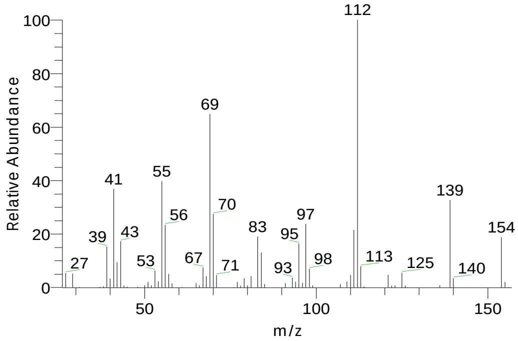

CYCLOHEXANONE, 5-METHYL-2-(1-METHYLETHYL)-, CIS-
Formula C10H18O, MW 154, CAS# 491-07-6, Entry# 38412
(1)-ISOMENTHONE

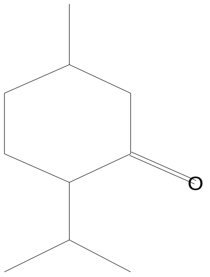

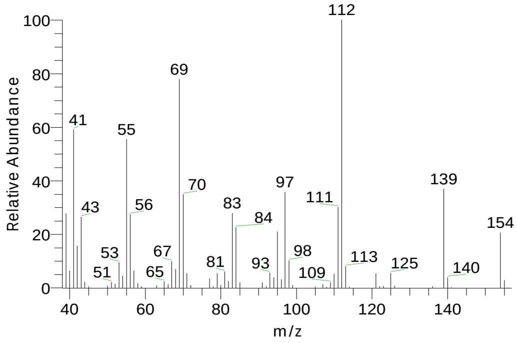

CYCLOHEXANONE, 5-METHYL-2-(1-METHYLETHYL)-, TRANS-
Formula C10H18O, MW 154, CAS# 89-80-5, Entry# 38733
2-ISOPROPYL-5-METHYLCYCLOHEXANONE #

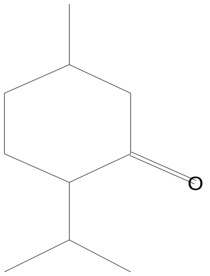

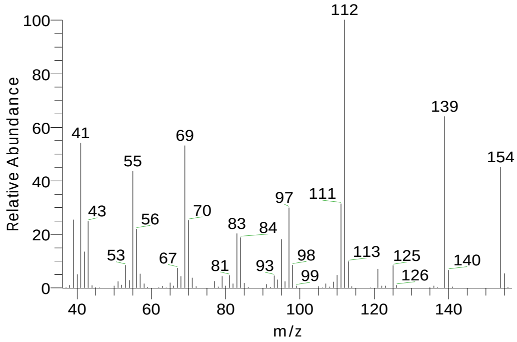

CYCLOHEXANONE, 5-METHYL-2-(1-METHYLETHYL)-, TRANS-
Formula C10H18O, MW 154, CAS# 89-80-5, Entry# 38426
2-ISOPROPYL-5-METHYLCYCLOHEXANONE #

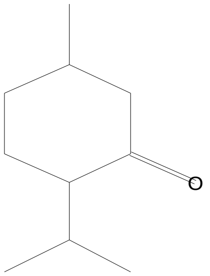

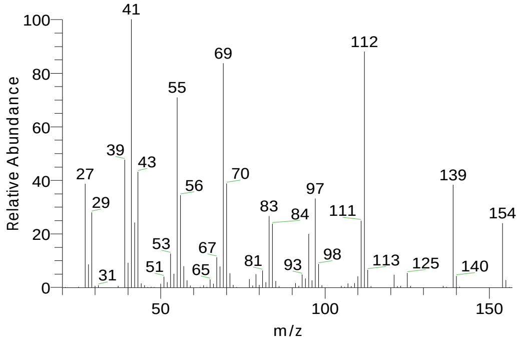

My GC-MS Report

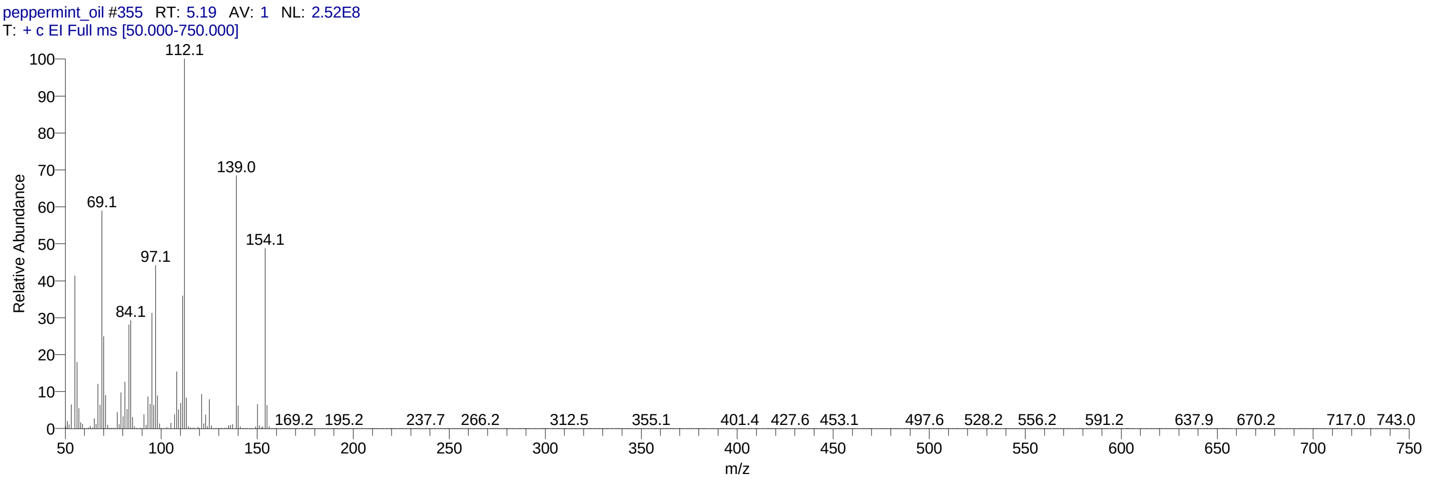

RT	Compound Name	Area %	MF	Molecular Formula	Molecular Weight	Cas #	Library
5.19	l-Menthone	4.91	944	C10H18O	154	14073-97-3	mainlib
5.19	Cyclohexanone, 5-methyl-2-(1-methylethyl)-	4.91	912	C10H18O	154	10458-14-7	mainlib
5.19	CYCLOHEXANONE, 5-METHYL-2-(1-METHYLETHYL)-	4.91	912	C10H18O	154	10458-14-7	WileyRegistry8e
5.19	Cyclohexanone, 5-methyl-2-(1-methylethyl)-, cis-	4.91	914	C10H18O	154	491-07-6	replib
5.19	Cyclohexanone, 5-methyl-2-(1-methylethyl)-, cis-	4.91	921	C10H18O	154	491-07-6	replib

Compound Structure

l-Menthone
Formula C10H18O, MW 154, CAS# 14073-97-3, Entry# 89414
Cyclohexanone, 5-methyl-2-(1-methylethyl)-, (2S-trans)-

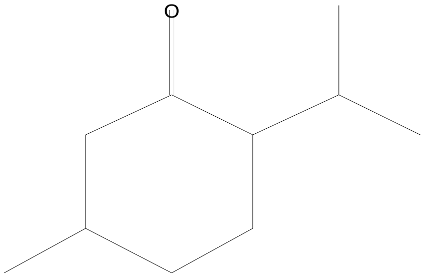

Cyclohexanone, 5-methyl-2-(1-methylethyl)-
Formula C10H18O, MW 154, CAS# 10458-14-7, Entry# 89410
p-Menthan-3-one

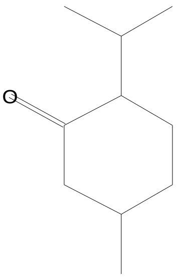

Hit Spectrum

SI 894, RSI 944, mainlib, Entry# 89414, CAS# 14073-97-3, l-Menthone

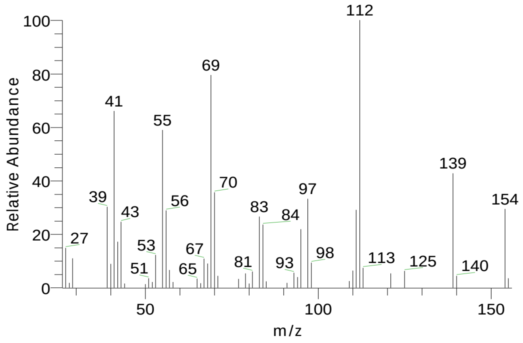

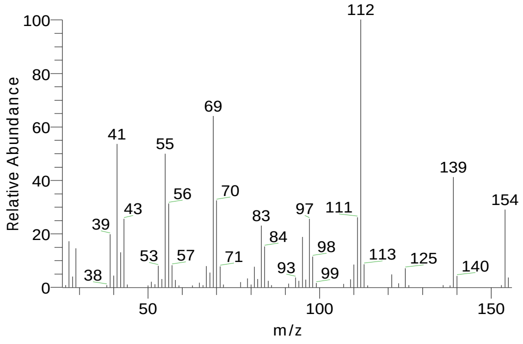

My GC-MS Report

Compound Structure | Hit Spectrum

CYCLOHEXANONE, 5-METHYL-2-(1-METHYLETHYL)-
Formula C10H18O, MW 154, CAS# 10458-14-7, Entry# 373487
2-ISOPROPYL-5-METHYLCYCLOHEXANONE

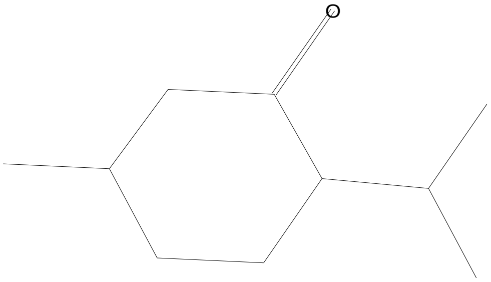

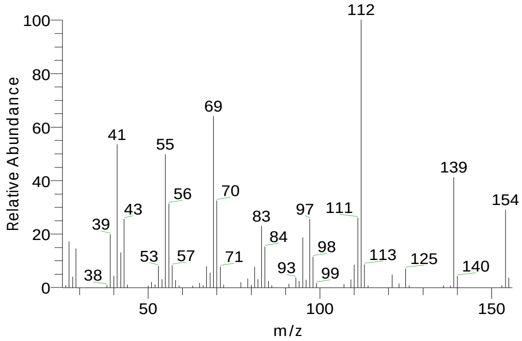

Cyclohexanone, 5-methyl-2-(1-methylethyl)-, cis-
Formula C10H18O, MW 154, CAS# 491-07-6, Entry# 17708
p-Menthan-3-one, cis-

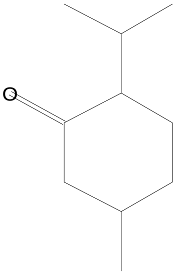

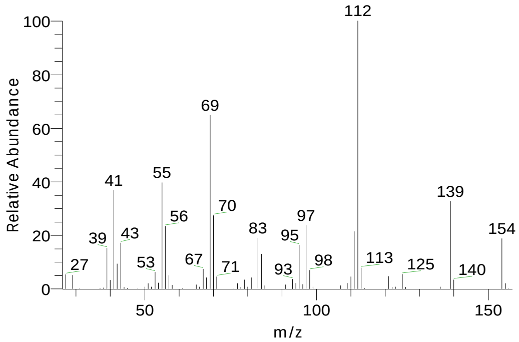

Cyclohexanone, 5-methyl-2-(1-methylethyl)-, cis-
Formula C10H18O, MW 154, CAS# 491-07-6, Entry# 1370
p-Menthan-3-one, cis-

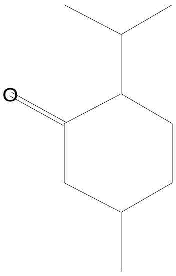

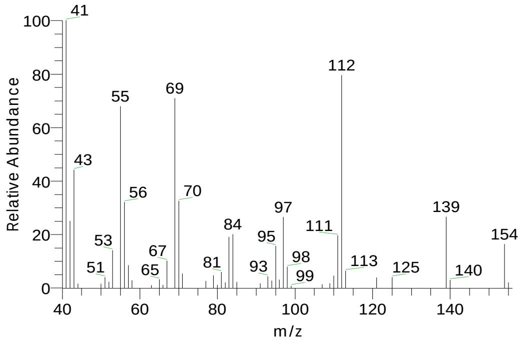

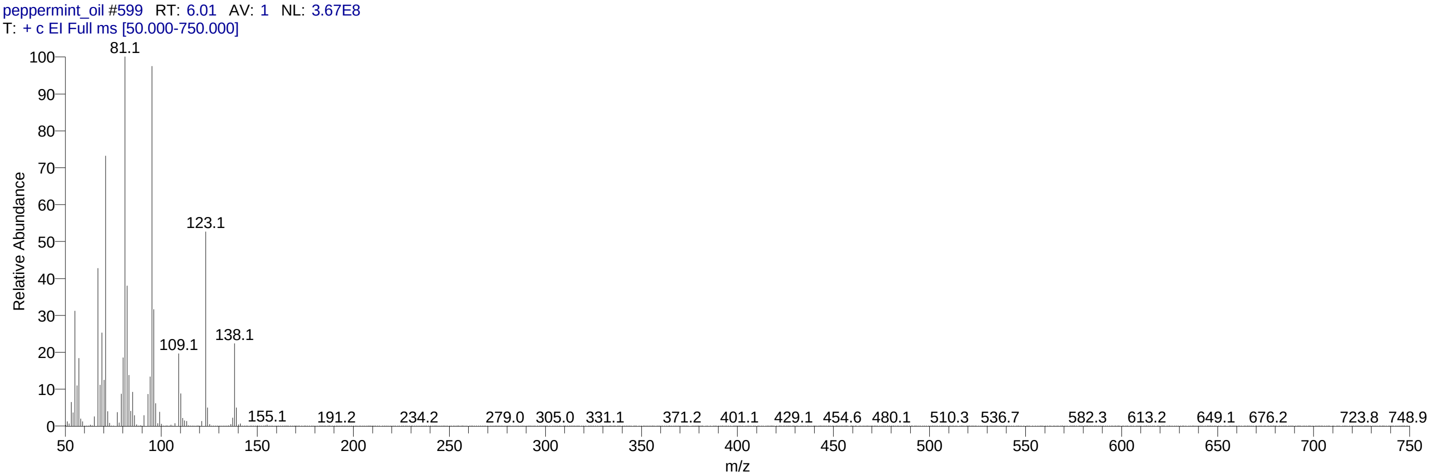

RT	Compound Name	Area %	MF	Molecular Formula	Molecular Weight	Cas #	Library
6.01	Levomenthol	26.41	937	C10H20O	156	2216-51-5	replib
6.01	CYCLOHEXANOL, 5-METHYL-2-(1-METHYLETHYL)-, (1à,2á,5à)-	26.41	926	C10H20O	156	89-78-1	WileyRegistry8e

My GC-MS Report

RT	Compound Name	Area %	MF	Molecular Formula	Molecular Weight	Cas #	Library
6.01	Levomenthol	26.41	928	C10H20O	156	2216-51-5	replib
6.01	Cyclohexanol, 5-methyl-2-(1-methylethyl)-, (1à,2á,5à)-(ñ)-	26.41	925	C10H20O	156	15356-70-4	mainlib
6.01	2-ISOPROPYL-5-METHYLCYCLOHEXANOL #	26.41	924	C10H20O	156	15356-70-4	WileyRegistry8e

Compound Structure | Hit Spectrum

Levomenthol
Formula C10H20O, MW 156, CAS# 2216-51-5, Entry# 9225
Cyclohexanol, 5-methyl-2-(1-methylethyl)-, [1R-(1à,2á,5à)]-

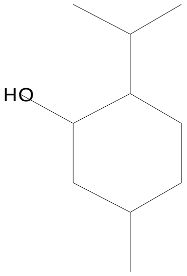

SI 935, RSI 937, replib, Entry# 9225, CAS# 2216-51-5, Levomenthol

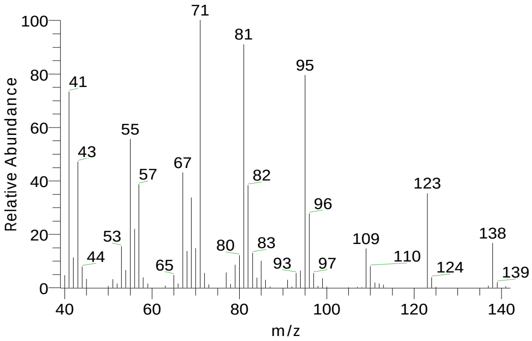

CYCLOHEXANOL, 5-METHYL-2-(1-METHYLETHYL)-, (1à,2á,5à)-
Formula C10H20O, MW 156, CAS# 89-78-1, Entry# 394892
(+-)-(1R*,3R*,4S*)-MENTHOL

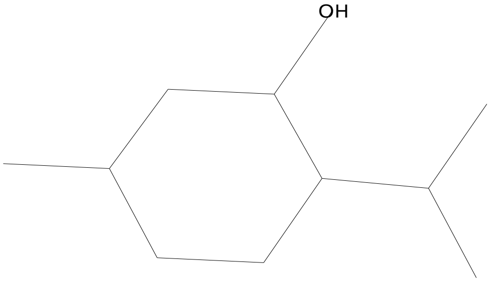

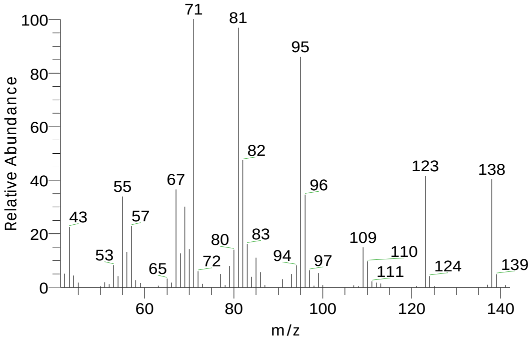

Levomenthol
Formula C10H20O, MW 156, CAS# 2216-51-5, Entry# 11525
Cyclohexanol, 5-methyl-2-(1-methylethyl)-, [1R-(1à,2á,5à)]-

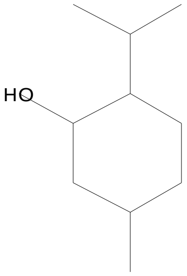

SI 921, RSI 928, replib, Entry# 11525, CAS# 2216-51-5, Levomenthol

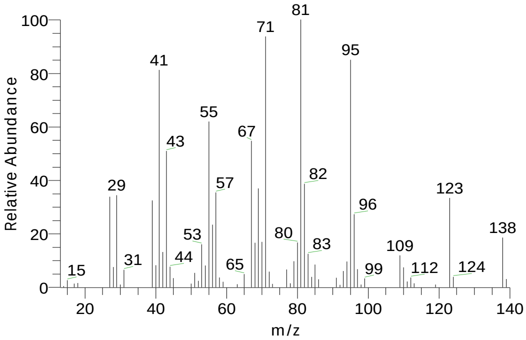

Cyclohexanol, 5-methyl-2-(1-methylethyl)-, (1à,2á,5à)-(ñ)-
Formula C10H20O, MW 156, CAS# 15356-70-4, Entry# 38982
Menthol, (ñ)-

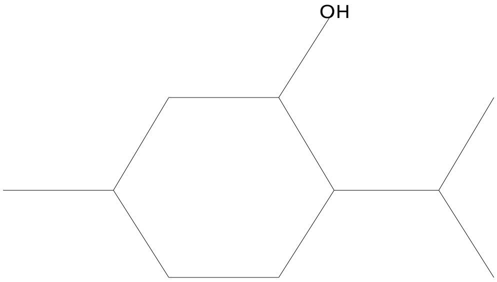

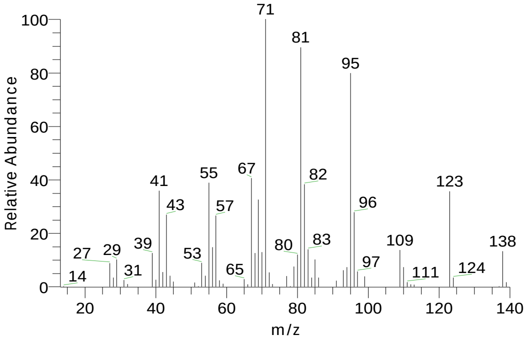

My GC-MS Report

Compound Structure | Hit Spectrum

2-ISOPROPYL-5-METHYLCYCLOHEXANOL #
Formula C10H20O, MW 156, CAS# 15356-70-4, Entry# 40741
(+-)-(1R*,3R*,4S*)-MENTHOL

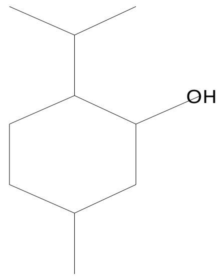

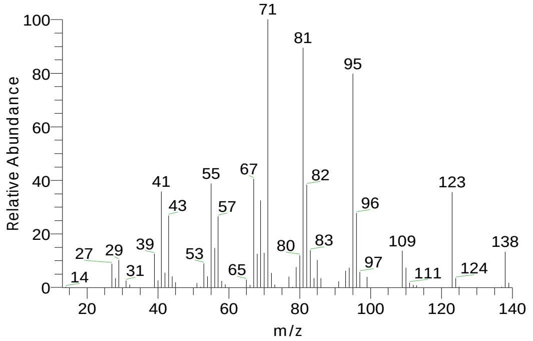

peppermint_oil #624 RT: 6.09 AV: 1 NL: 7.81E7
T: + c EI Full ms [50.000-750.000]

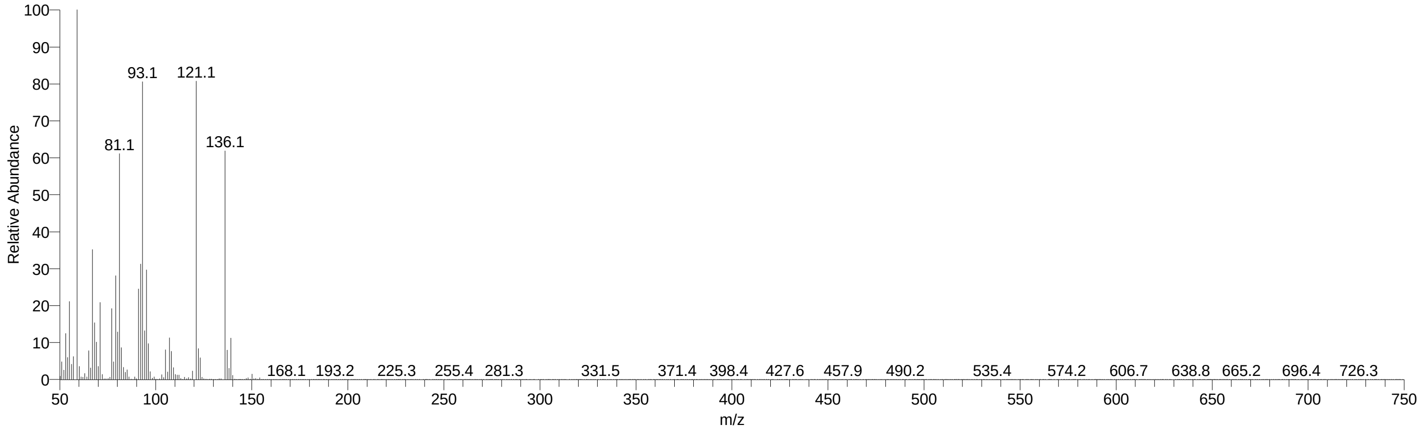

RT	Compound Name	Area %	MF	Molecular Formula	Molecular Weight	Cas #	Library
6.09	(-)-(1S,2R,4R)-BETA-FENCHOL	1.63	907	C10H18O	154	470-08-6	WileyRegistry8e
6.09	3-CYCLOHEXENE-1-METHANOL, à,à,4-TRIMETHYL-	1.63	903	C10H18O	154	98-55-5	WileyRegistry8e
6.09	3-CYCLOHEXENE-1-METHANOL, à,à,4-TRIMETHYL-, (S)-	1.63	903	C10H18O	154	10482-56-1	WileyRegistry8e
6.09	L-à-Terpineol	1.63	899	C10H18O	154	10482-56-1	replib
6.09	(+)-ALPHA-TERPINEOL (P-MENTH-1-EN-8-OL)	1.63	898	C10H18O	154	NA	WileyRegistry8e

Compound Structure | Hit Spectrum

(-)-(1S,2R,4R)-BETA-FENCHOL
Formula C10H18O, MW 154, CAS# 470-08-6, Entry# 38737
(-)-BETA-FENCHOL

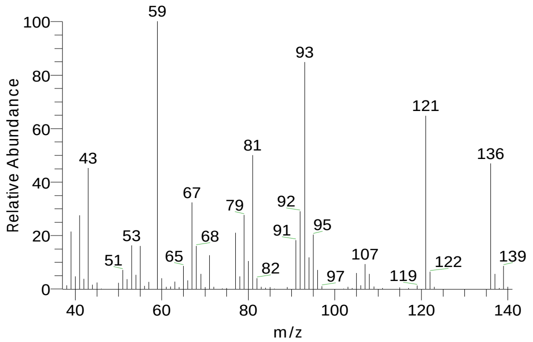

My GC-MS Report

Compound Structure | Hit Spectrum

3-CYCLOHEXENE-1-METHANOL, à,à,4-TRIMETHYL-
Formula C10H18O, MW 154, CAS# 98-55-5, Entry# 38742
3-CYCLOHEXENE-1-METHANOL, à,à,4-TRIMETHYL-, (S)-

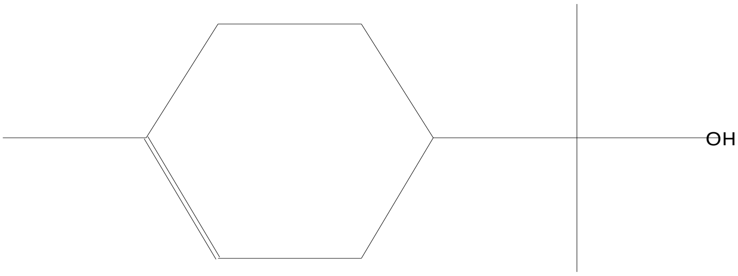

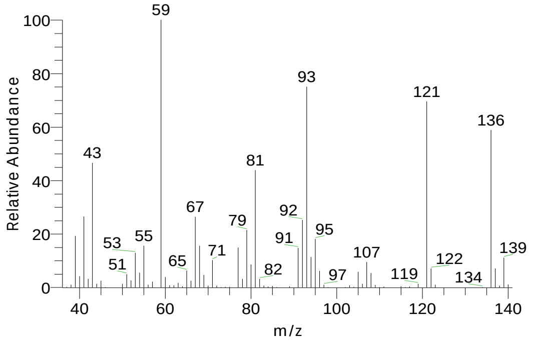

3-CYCLOHEXENE-1-METHANOL, à,à,4-TRIMETHYL-, (S)-
Formula C10H18O, MW 154, CAS# 10482-56-1, Entry# 38379
2-(4-METHYL-3-CYCLOHEXEN-1-YL)-2-PROPANOL #

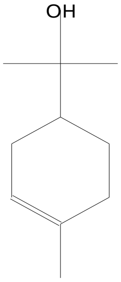

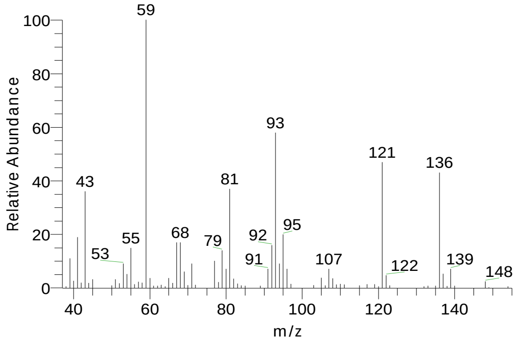

L-à-Terpineol
Formula C10H18O, MW 154, CAS# 10482-56-1, Entry# 7441
3-Cyclohexene-1-methanol, à,à,4-trimethyl-, (S)-

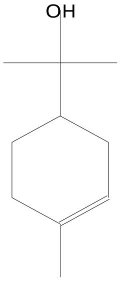

SI 893, RSI 899, replib, Entry# 7441, CAS# 10482-56-1, L-à-Terpineol

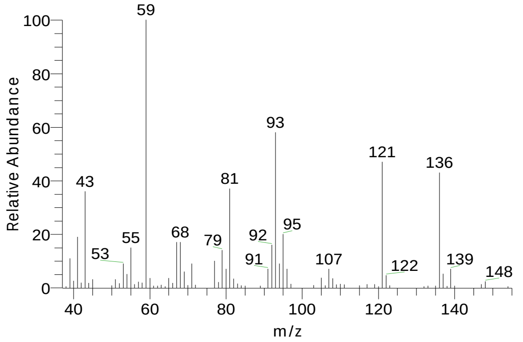

(+)-ALPHA-TERPINEOL (P-MENTH-1-EN-8-OL)
Formula C10H18O, MW 154, CAS# NA, Entry# 383373
2-(4-METHYL-3-CYCLOHEXEN-1-YL)-2-PROPANOL

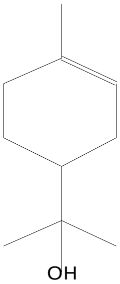

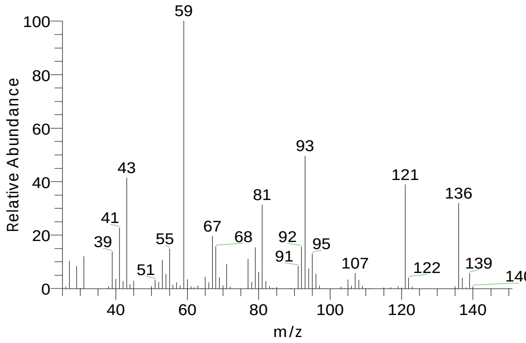

My GC-MS Report

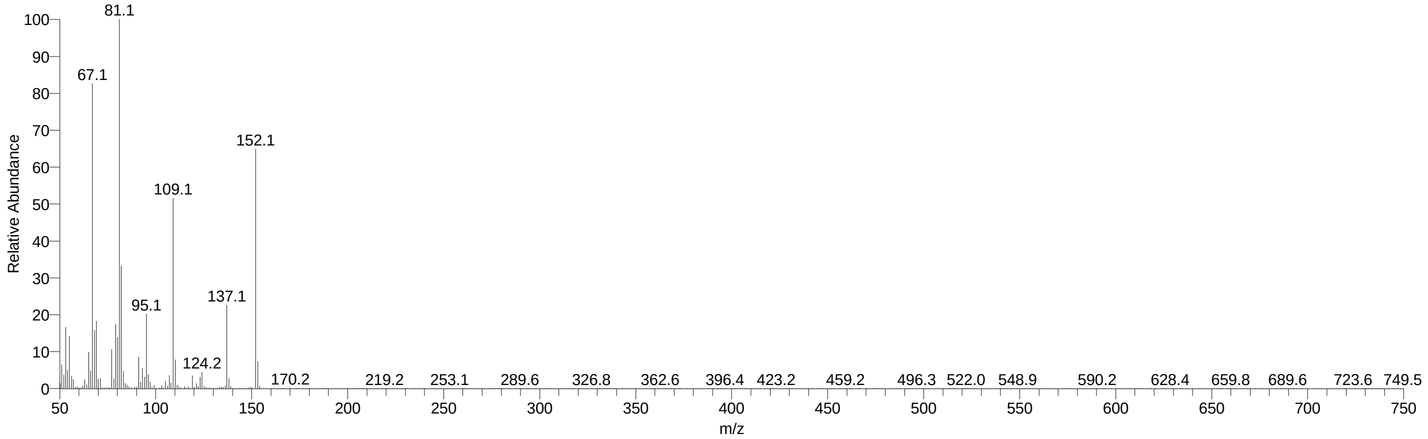

RT	Compound Name	Area %	MF	Molecular Formula	Molecular Weight	Cas #	Library
6.80	Cyclohexanone, 5-methyl-2-(1-methylethylidene)-	1.94	931	C10H16O	152	15932-80-6	replib
6.80	2-ISOPROPYLIDENE-5-METHYL-CYCLOHEXANONE	1.94	931	C10H16O	152	NA	WileyRegistry8e
6.80	CIS-ISOPULEGONE	1.94	922	C10H16O	152	NA	WileyRegistry8e
6.80	CYCLOHEXANONE, 5-METHYL-2-(1-METHYLETHYLIDENE)-, (R)-	1.94	919	C10H16O	152	89-82-7	WileyRegistry8e
6.80	CYCLOHEXANONE, 5-METHYL-2-(1-METHYLETHYLIDENE)-	1.94	915	C10H16O	152	15932-80-6	WileyRegistry8e

Compound Structure	Hit Spectrum

Cyclohexanone, 5-methyl-2-(1-methylethylidene)-
Formula C10H16O, MW 152, CAS# 15932-80-6, Entry# 11655
p-Menth-4(8)-en-3-one

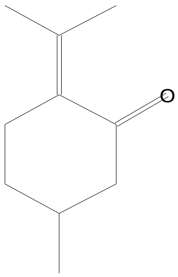

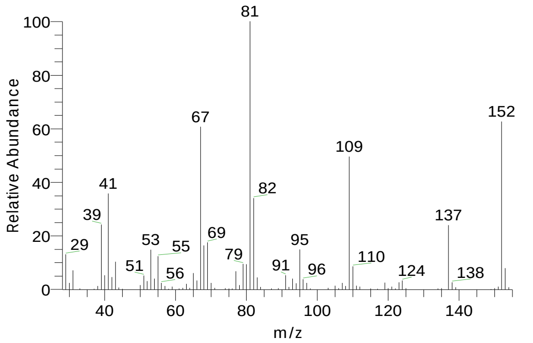

2-ISOPROPYLIDENE-5-METHYL-CYCLOHEXANONE
Formula C10H16O, MW 152, CAS# NA, Entry# 370741
5-METHYL-2-(1-METHYLETHYLIDENE)CYCLOHEXANONE

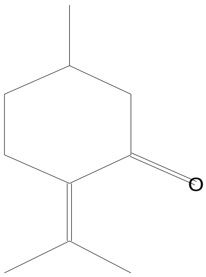

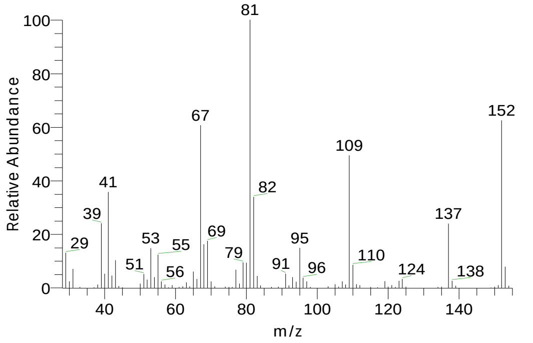

My GC-MS Report

Compound Structure	Hit Spectrum

CIS-ISOPULEGONE
Formula C10H16O, MW 152, CAS# NA, Entry# 36394

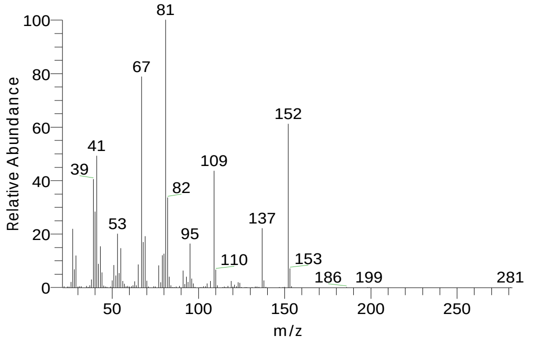

CYCLOHEXANONE, 5-METHYL-2-(1-METHYLETHYLIDENE)-, (R)-
Formula C10H16O, MW 152, CAS# 89-82-7, Entry# 36050
5-METHYL-2-(1-METHYLETHYLIDENE)CYCLOHEXANONE #

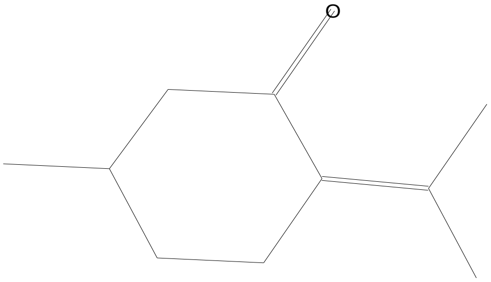

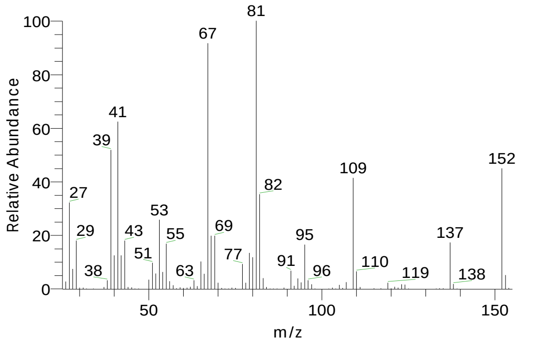

CYCLOHEXANONE, 5-METHYL-2-(1-METHYLETHYLIDENE)-
Formula C10H16O, MW 152, CAS# 15932-80-6, Entry# 36046
5-METHYL-2-(1-METHYLETHYLIDENE)CYCLOHEXANONE #

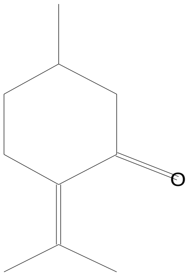

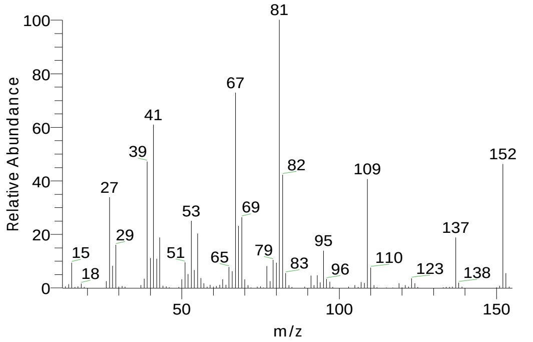

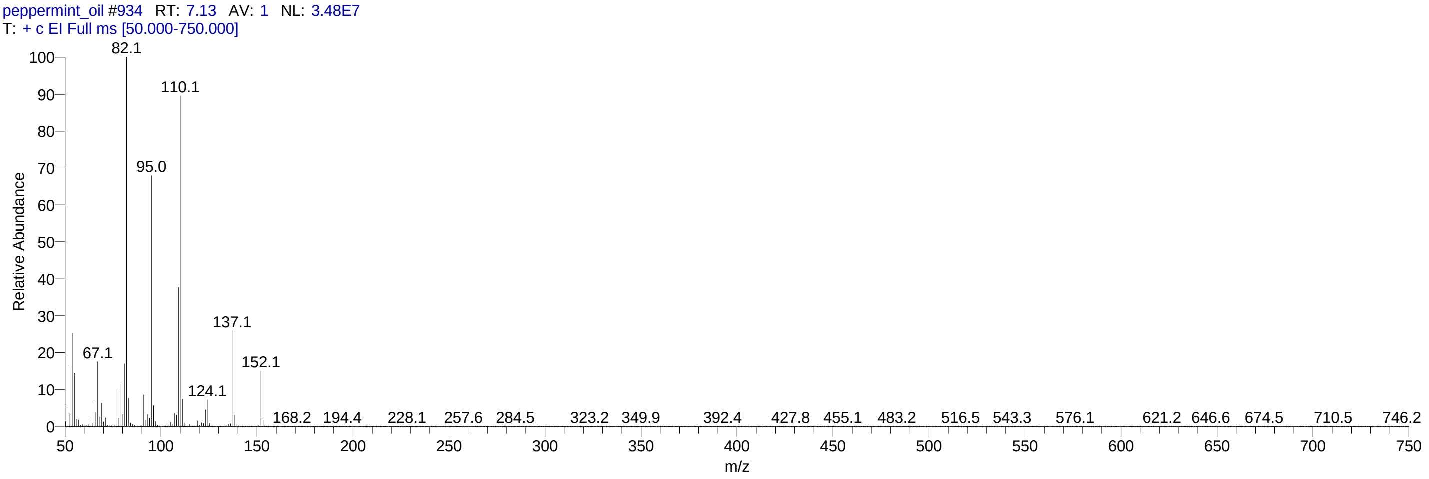

RT	Compound Name	Area %	MF	Molecular Formula	Molecular Weight	Cas #	Library
7.13	2-CYCLOHEXEN-1-ONE, 3-METHYL-6-(1-METHYLETHYL)-	1.32	914	C10H16O	152	89-81-6	WileyRegistry8e
7.13	2-Cyclohexen-1-one, 3-methyl-6-(1-methylethyl)-	1.32	926	C10H16O	152	89-81-6	replib

My GC-MS Report

RT	Compound Name	Area %	MF	Molecular Formula	Molecular Weight	Cas #	Library
7.13	2-Cyclohexen-1-one, 3-methyl-6-(1-methylethyl)-	1.32	912	C10H16O	152	89-81-6	replib
7.13	2-CYCLOHEXEN-1-ONE, 3-METHYL-6-(1-METHYLETHYL)-	1.32	903	C10H16O	152	89-81-6	WileyRegistry8e
7.13	2-Cyclohexen-1-one, 3-methyl-6-(1-methylethyl)-	1.32	896	C10H16O	152	89-81-6	mainlib

Compound Structure | Hit Spectrum

2-CYCLOHEXEN-1-ONE, 3-METHYL-6-(1-METHYLETHYL)-
Formula C10H16O, MW 152, CAS# 89-81-6, Entry# 35991
6-ISOPROPYL-3-METHYL-2-CYCLOHEXEN-1-ONE #

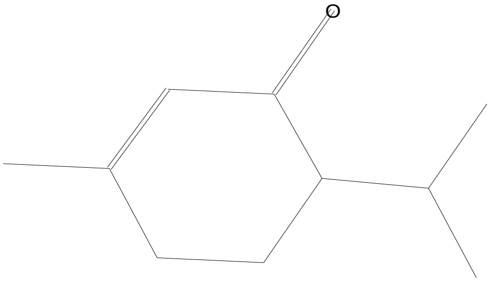

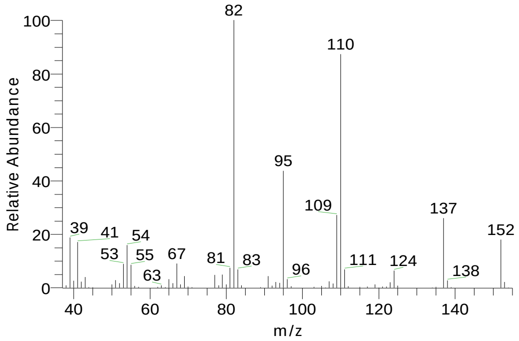

2-Cyclohexen-1-one, 3-methyl-6-(1-methylethyl)-
Formula C10H16O, MW 152, CAS# 89-81-6, Entry# 17471
p-Menth-1-en-3-one

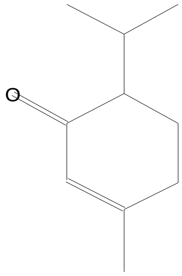

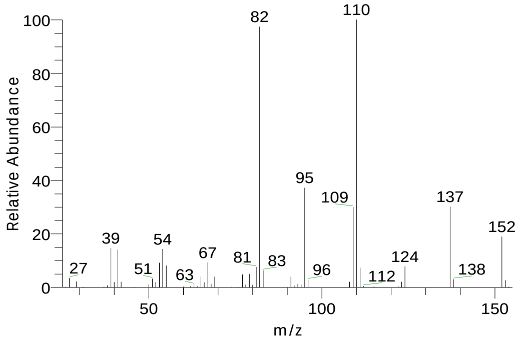

2-Cyclohexen-1-one, 3-methyl-6-(1-methylethyl)-
Formula C10H16O, MW 152, CAS# 89-81-6, Entry# 11813
p-Menth-1-en-3-one

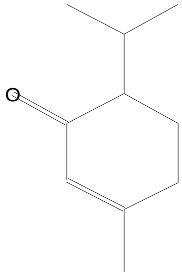

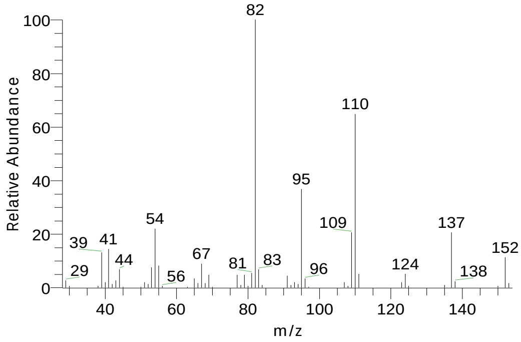

2-CYCLOHEXEN-1-ONE, 3-METHYL-6-(1-METHYLETHYL)-
Formula C10H16O, MW 152, CAS# 89-81-6, Entry# 35994
6-ISOPROPYL-3-METHYL-2-CYCLOHEXEN-1-ONE #

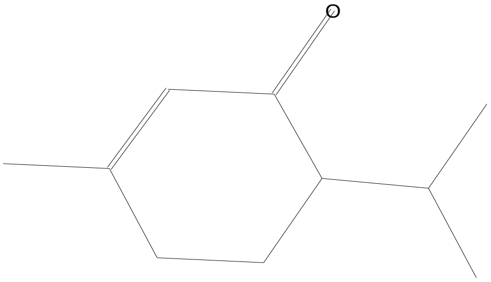

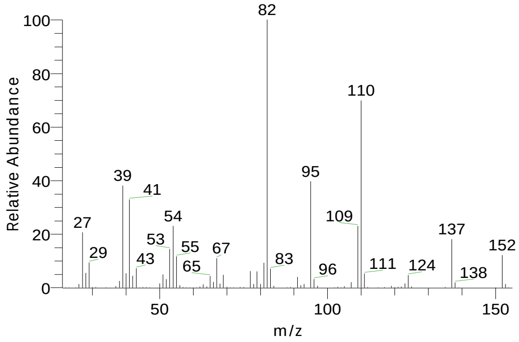

My GC-MS Report

Compound Structure | Hit Spectrum

2-Cyclohexen-1-one, 3-methyl-6-(1-methylethyl)-
Formula C10H16O, MW 152, CAS# 89-81-6, Entry# 51632
p-Menth-1-en-3-one

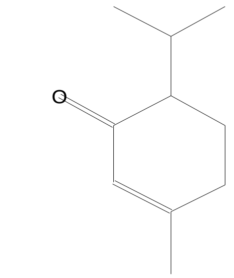

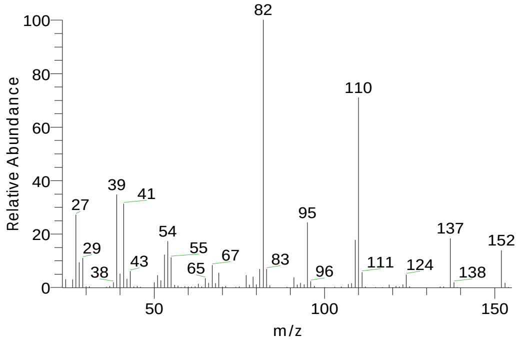

peppermint_oil #1188 RT: 7.98 AV: 1 NL: 3.00E8
T: + c EI Full ms [50.000-750.000]

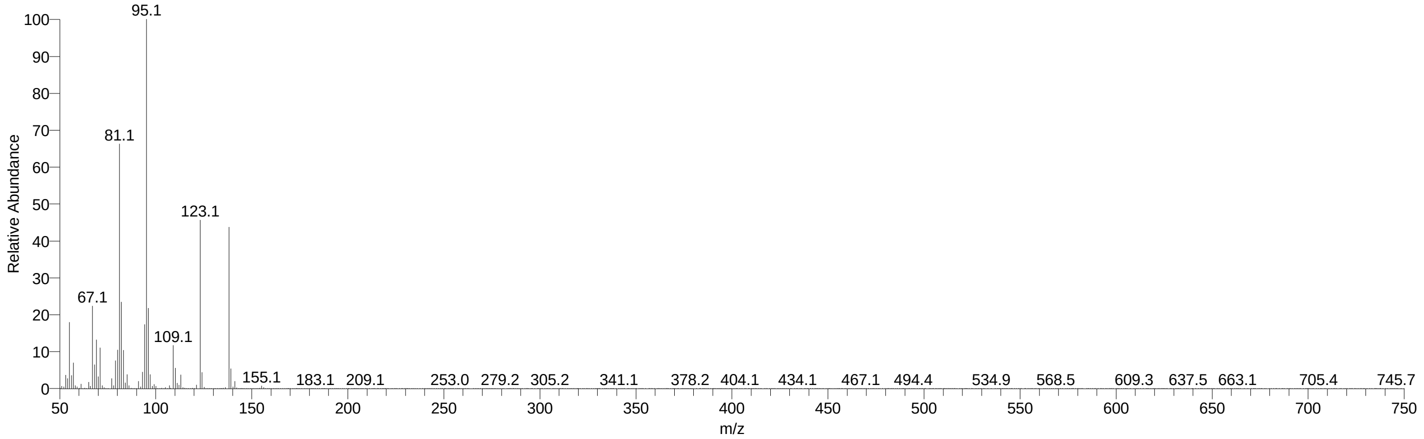

RT	Compound Name	Area %	MF	Molecular Formula	Molecular Weight	Cas #	Library
7.98	Cyclohexanol, 5-methyl-2-(1-methylethyl)-, acetate	32.76	967	C12H22O2	198	16409-45-3	replib
7.98	CYCLOHEXANOL, 5-METHYL-2-(1-METHYLETHYL)-, ACETATE	32.76	959	C12H22O2	198	2623-23-6	WileyRegistry8e
7.98	Menthyl acetate	32.76	951	C12H22O2	198	89-48-5	mainlib
7.98	Menthyl acetate	32.76	948	C12H22O2	198	89-48-5	replib
7.98	CYCLOHEXANOL, 5-METHYL-2-(1-METHYLETHYL)-, ACETATE	32.76	948	C12H22O2	198	16409-45-3	WileyRegistry8e

Compound Structure | Hit Spectrum

Cyclohexanol, 5-methyl-2-(1-methylethyl)-, acetate
Formula C12H22O2, MW 198, CAS# 16409-45-3, Entry# 14465
Menthol, acetate

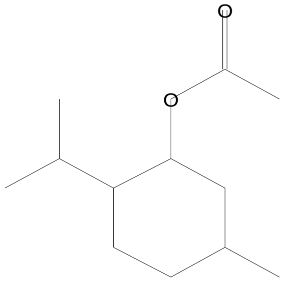

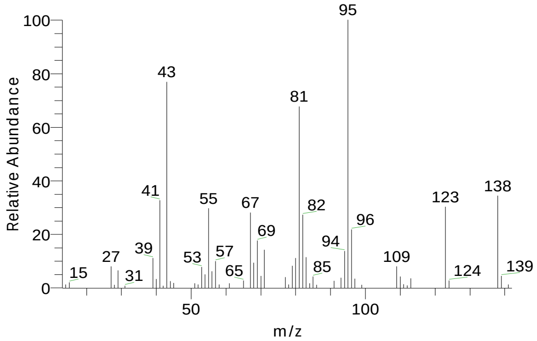

My GC-MS Report

Compound Structure | Hit Spectrum

CYCLOHEXANOL, 5-METHYL-2-(1-METHYLETHYL)-, ACETATE
Formula $C12H22O2$, MW 198, CAS# 2623-23-6, Entry# 82542
CYCLOHEXANOL, 5-METHYL-2-(1-METHYLETHYL)-, ACETATE, [1R-(1à,2á,5à)]-

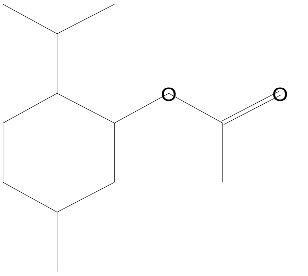

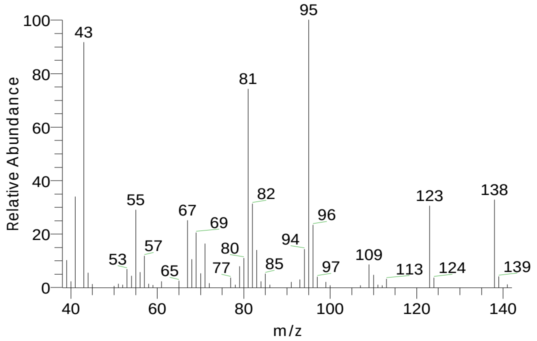

Menthyl acetate
Formula $C12H22O2$, MW 198, CAS# 89-48-5, Entry# 10354
Cyclohexanol, 5-methyl-2-(1-methylethyl)-, acetate, (1à,2á,5à)-

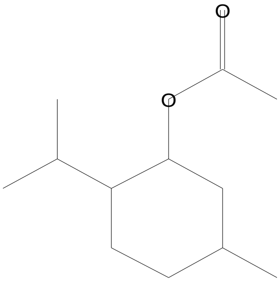

SI 931, RSI 951, mainlib, Entry# 10354, CAS# 89-48-5, Menthyl acetate

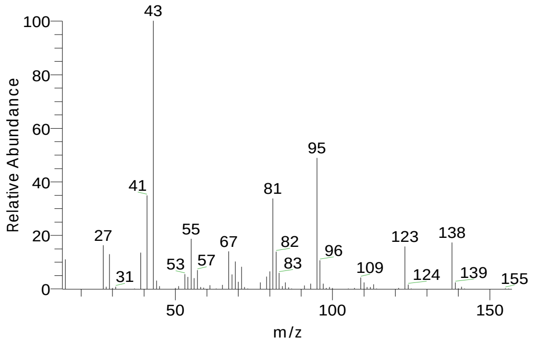

Menthyl acetate
Formula $C12H22O2$, MW 198, CAS# 89-48-5, Entry# 3027
Cyclohexanol, 5-methyl-2-(1-methylethyl)-, acetate, (1à,2á,5à)-

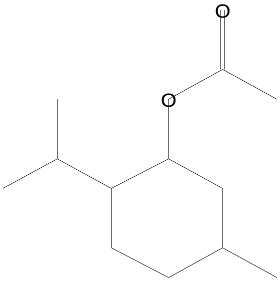

SI 930, RSI 948, replib, Entry# 3027, CAS# 89-48-5, Menthyl acetate

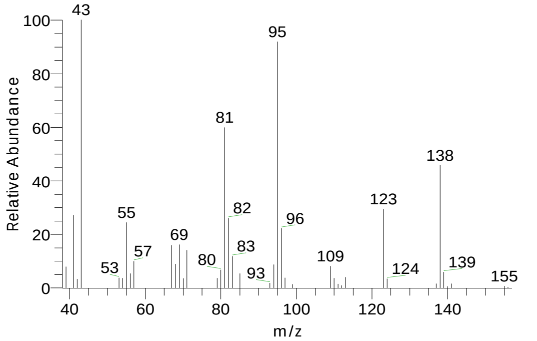

CYCLOHEXANOL, 5-METHYL-2-(1-METHYLETHYL)-, ACETATE
Formula $C12H22O2$, MW 198, CAS# 16409-45-3, Entry# 82538
2-ISOPROPYL-5-METHYLCYCLOHEXYL ACETATE #

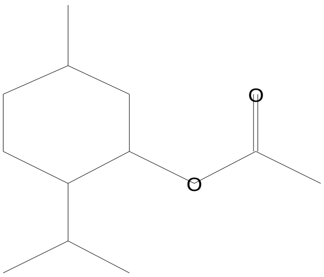

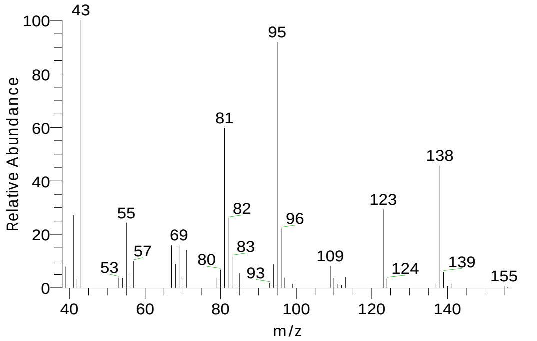

My GC-MS Report

peppermint_oil #1241 RT: 8.16 AV: 1 NL: 8.37E6
T: + c EI Full ms [50.000-750.000]

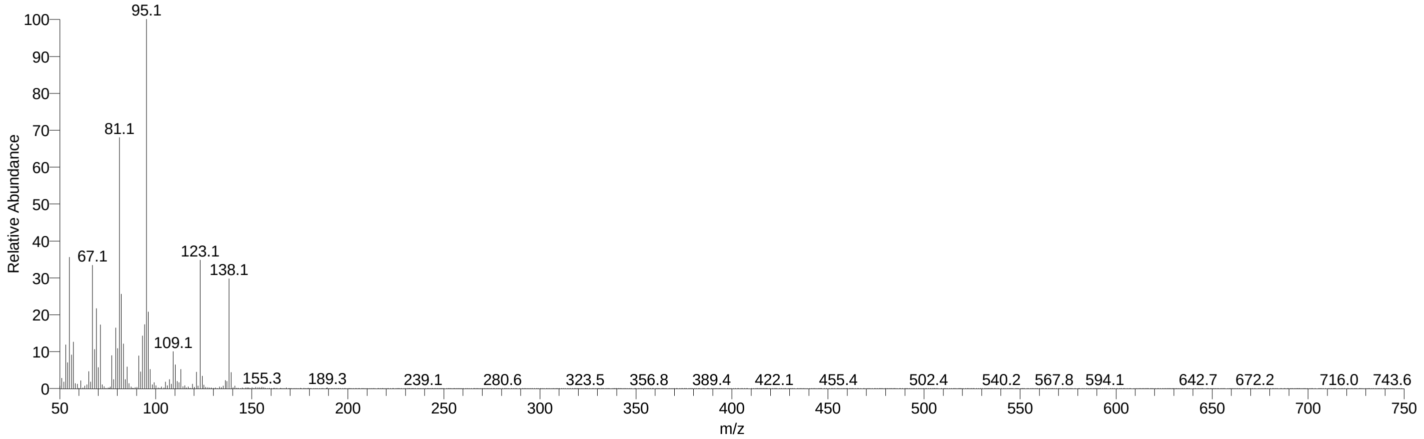

RT	Compound Name	Area %	MF	Molecular Formula	Molecular Weight	Cas #	Library
8.16	Cyclohexanol, 5-methyl-2-(1-methylethyl)-, acetate	0.21	955	C12H22O2	198	16409-45-3	replib
8.16	CYCLOHEXANOL, 5-METHYL-2-(1-METHYLETHYL)-, ACETATE	0.21	942	C12H22O2	198	2623-23-6	WileyRegistry8e
8.16	CYCLOHEXANOL, 5-METHYL-2-(1-METHYLETHYL)-, ACETATE	0.21	916	C12H22O2	198	16409-45-3	WileyRegistry8e
8.16	Menthyl acetate	0.21	939	C12H22O2	198	89-48-5	mainlib
8.16	CYCLOHEXANOL, 5-METHYL-2-(1-METHYLETHYL)-, ACETATE	0.21	916	C12H22O2	198	16409-45-3	WileyRegistry8e

Compound Structure | Hit Spectrum

Cyclohexanol, 5-methyl-2-(1-methylethyl)-, acetate
Formula C12H22O2, MW 198, CAS# 16409-45-3, Entry# 14465
Menthol, acetate

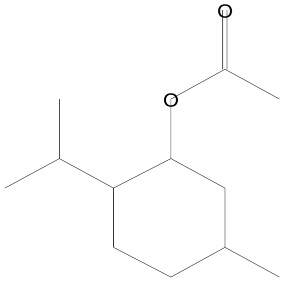

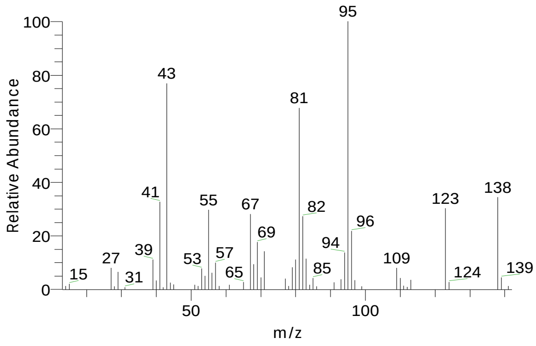

CYCLOHEXANOL, 5-METHYL-2-(1-METHYLETHYL)-, ACETATE
Formula C12H22O2, MW 198, CAS# 2623-23-6, Entry# 82542
CYCLOHEXANOL, 5-METHYL-2-(1-METHYLETHYL)-, ACETATE, [1R-(1à,2á,5à)]-

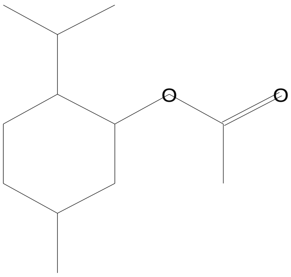

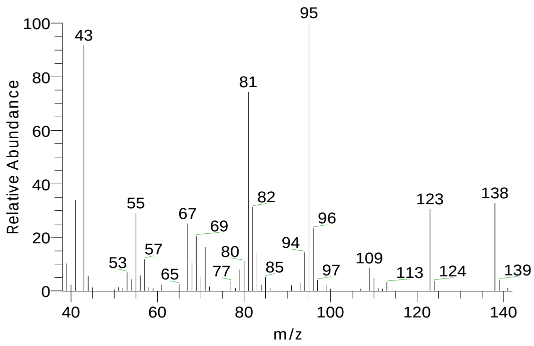

My GC-MS Report

Compound Structure | Hit Spectrum

CYCLOHEXANOL, 5-METHYL-2-(1-METHYLETHYL)-, ACETATE
Formula C12H22O2, MW 198, CAS# 16409-45-3, Entry# 82536
2-ISOPROPYL-5-METHYLCYCLOHEXYL ACETATE #

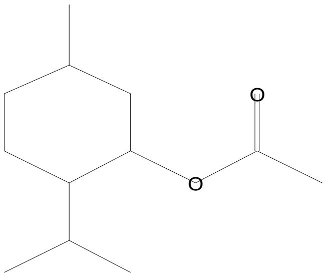

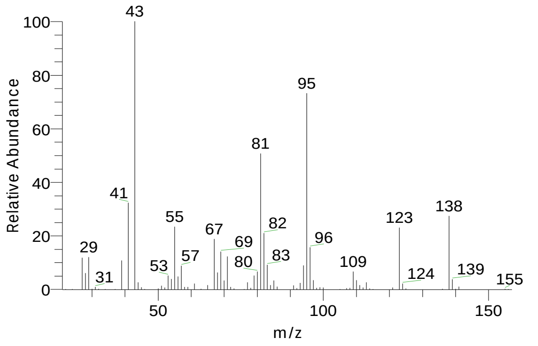

SI 894, RSI 939, mainlib, Entry# 10354, CAS# 89-48-5, Menthyl acetate

Menthyl acetate
Formula C12H22O2, MW 198, CAS# 89-48-5, Entry# 10354
Cyclohexanol, 5-methyl-2-(1-methylethyl)-, acetate, (1à,2á,5à)-

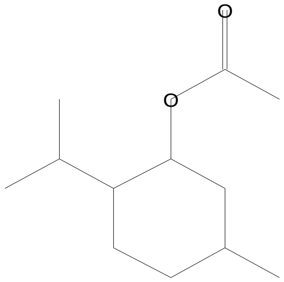

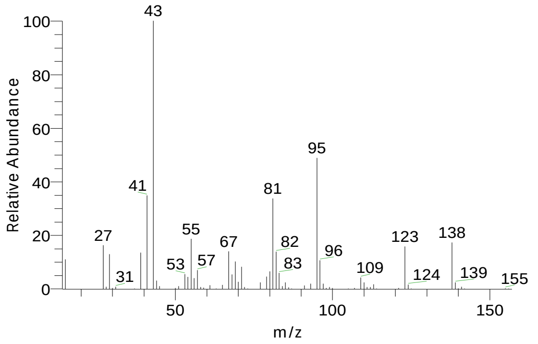

CYCLOHEXANOL, 5-METHYL-2-(1-METHYLETHYL)-, ACETATE
Formula C12H22O2, MW 198, CAS# 16409-45-3, Entry# 82534
2-ISOPROPYL-5-METHYLCYCLOHEXYL ACETATE #

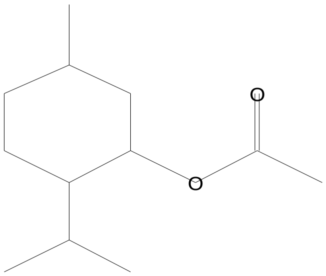

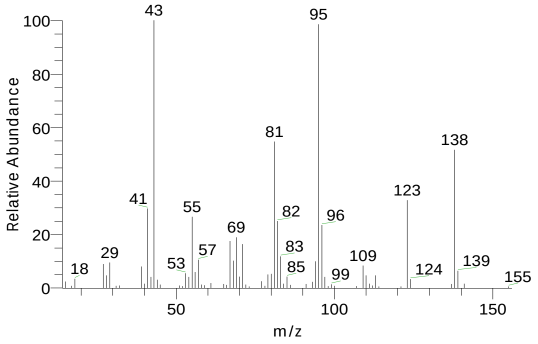

peppermint_oil #1392 RT: 8.67 AV: 1 NL: 7.27E6
T: + c EI Full ms [50.000-750.000]

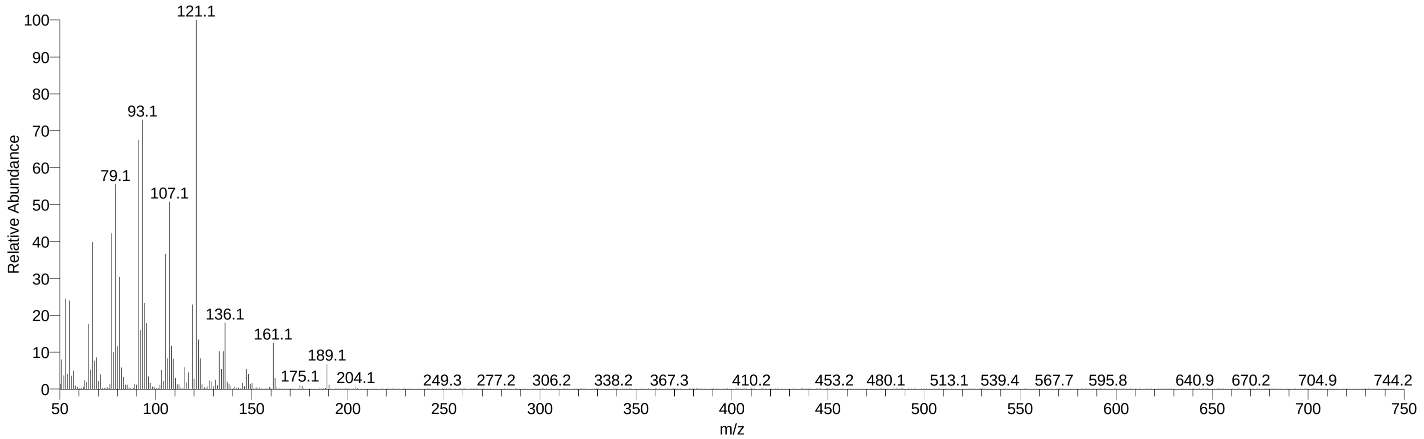

RT	Compound Name	Area %	MF	Molecular Formula	Molecular Weight	Cas #	Library
8.67	BICYCLO[8.1.0]UNDECA-2,6-DIENE, 3,7,11,11-TETRAMETHYL-, (1R*,2Z,6E,10R*)-(.+-.)-	0.46	902	C15H24	204	100762-46-7	WileyRegistry8e
8.67	Elemene isomer	0.46	927	C15H24	204	NA	mainlib

My GC-MS Report

RT	Compound Name	Area %	MF	Molecular Formula	Molecular Weight	Cas #	Library
8.67	ç-Elemene	0.46	885	C15H24	204	29873-99-2	replib
8.67	Cyclohexane, 1-ethenyl-1-methyl-2-(1-methylethenyl)-4-(1-methylethylidene)-	0.46	901	C15H24	204	3242-08-8	mainlib
8.67	ç-Elemene	0.46	867	C15H24	204	29873-99-2	mainlib

Compound Structure | Hit Spectrum

BICYCLO[8.1.0]UNDECA-2,6-DIENE, 3,7,11,11-TETRAMETHYL-, (1R*,2Z,6E,10R*)-(.+-.)-
Formula C15H24, MW 204, CAS# 100762-46-7, Entry# 89533
(.+-.)-LEPIDOZENE

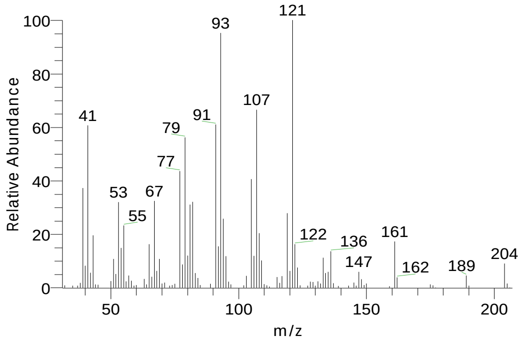

Elemene isomer
Formula C15H24, MW 204, CAS# NA, Entry# 102822
$:28BQSLMQNYHVFRDT-UHFFFAOYSA-N

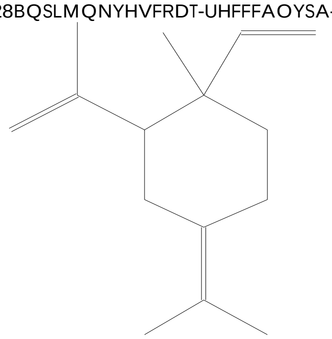

SI 890, RSI 927, mainlib, Entry# 102822, CAS# NA, Elemene isomer

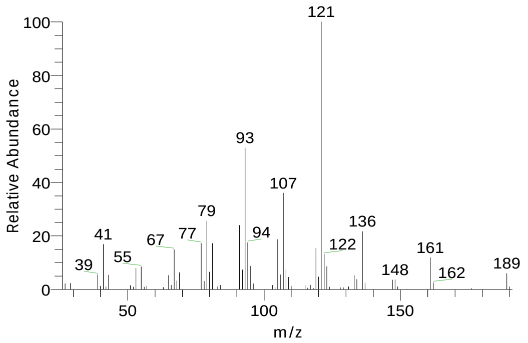

ç-Elemene
Formula C15H24, MW 204, CAS# 29873-99-2, Entry# 1378
1-Methyl-2-(1-methylethenyl)-4-(1-methylethylidene)-1-vinylcyclohexane), (1R-trans)-

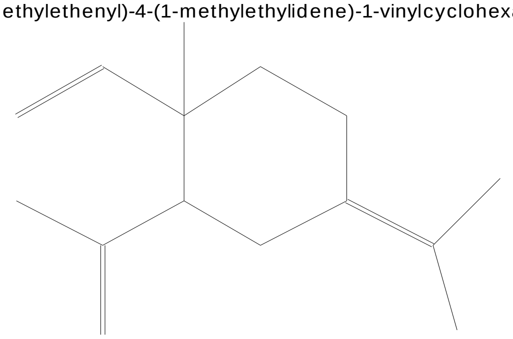

SI 873, RSI 885, replib, Entry# 1378, CAS# 29873-99-2, ç-Elemene

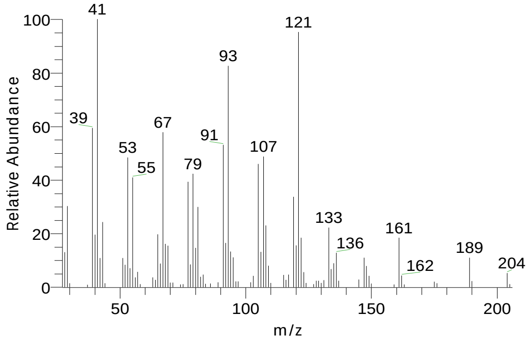

Cyclohexane, 1-ethenyl-1-methyl-2-(1-methylethenyl)-4-(1-methylethylidene)-
Formula C15H24, MW 204, CAS# 3242-08-8, Entry# 102823
o-Menth-8-ene, 4-isopropylidene-1-vinyl-

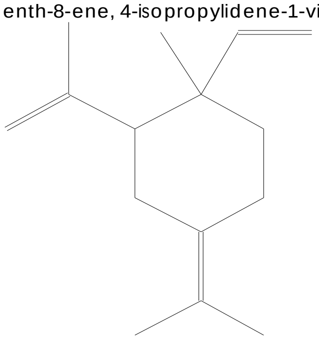

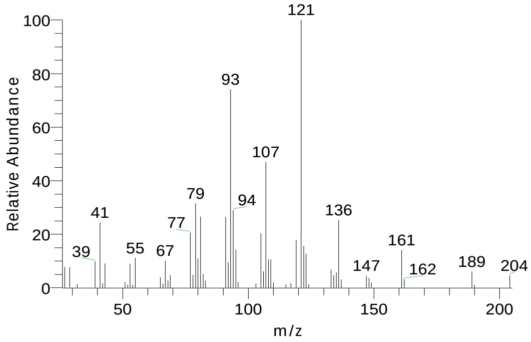

My GC-MS Report

Compound Structure

Hit Spectrum

ç-Elemene
Formula C15H24, MW 204, CAS# 29873-99-2, Entry# 102784
1-Methyl-2-(1-methylethenyl)-4-(1-methylethylidene)-1-vinylcyclohexane), (1R-trans)-

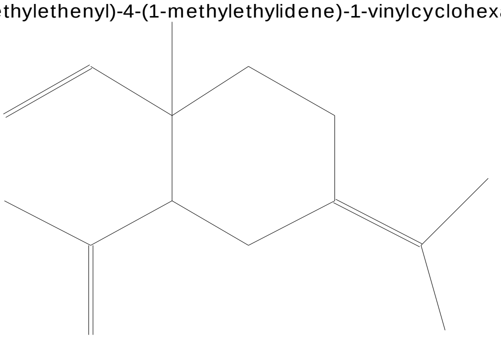

SI 859, RSI 867, mainlib, Entry# 102784, CAS# 29873-99-2, ç-Elemene

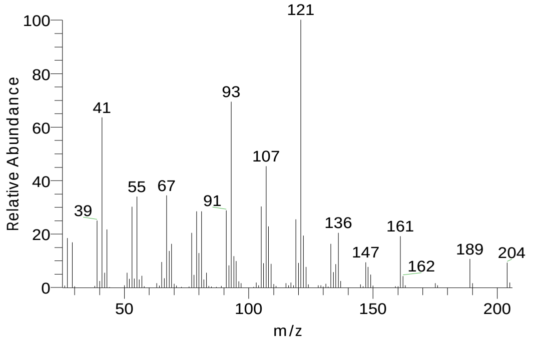

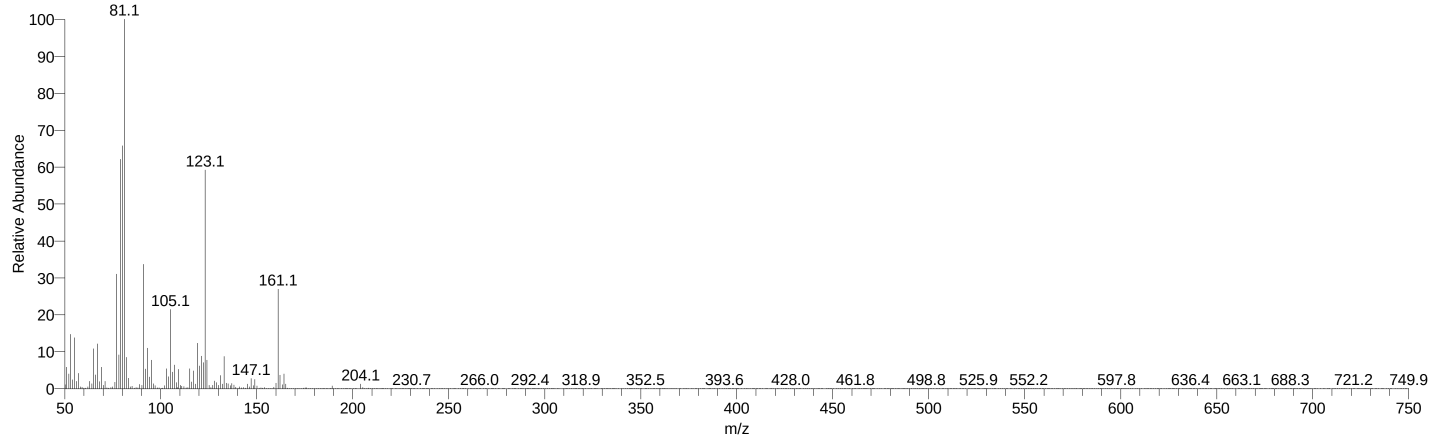

RT	Compound Name	Area %	MF	Molecular Formula	Molecular Weight	Cas #	Library
9.73	CYCLOBUTA[1,2:3,4]DICYCLOPENTENE, 1,2,3,3A,3Bá,4,5,6,6Aá,6Bà-DECAHYDRO-1à-ISOPROPYL-3Aà-METHYL-6-METHYLENE-	0.58	931	C15H24	204	5208-59-3	WileyRegistry8e
9.73	(-)-á-Bourbonene	0.58	896	C15H24	204	5208-59-3	replib
9.73	CYCLOBUTA[1,2:3,4]DICYCLOPENTENE, 1,2,3,3A,3Bá,4,5,6,6Aá,6Bà-DECAHYDRO-1à-ISOPROPYL-3Aà-METHYL-6-METHYLENE-	0.58	874	C15H24	204	5208-59-3	WileyRegistry8e
9.73	(-)-á-Bourbonene	0.58	873	C15H24	204	5208-59-3	mainlib
9.73	CYCLOBUTA[1,2:3,4]DICYCLOPENTENE, 1,2,3,3A,3Bá,4,5,6,6Aá,6Bà-DECAHYDRO-1à-ISOPROPYL-3Aà-METHYL-6-METHYLENE-	0.58	898	C15H24	204	5208-59-3	WileyRegistry8e

My GC-MS Report

Compound Structure | Hit Spectrum

Formula C15H24, MW 204, CAS# 5208-59-3, Entry# 89559

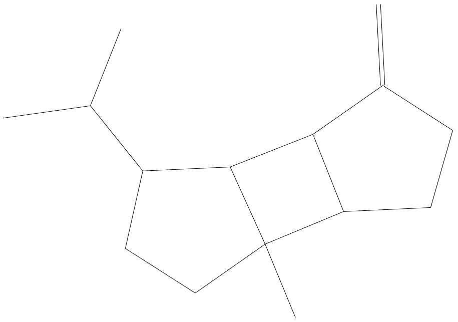

(-)-á-Bourbonene

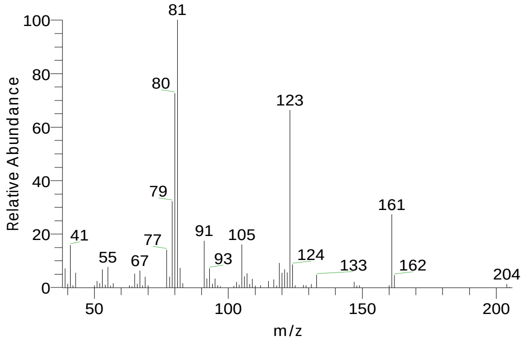

SI 885, RSI 896, replib, Entry# 11633, CAS# 5208-59-3, (-)-á-Bourbonene

Formula C15H24, MW 204, CAS# 5208-59-3, Entry# 11633

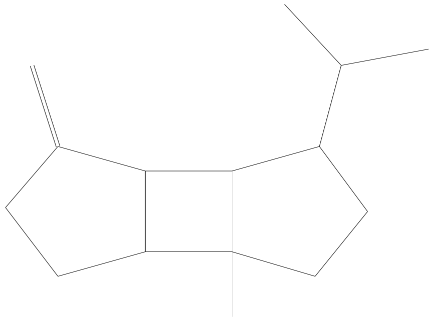

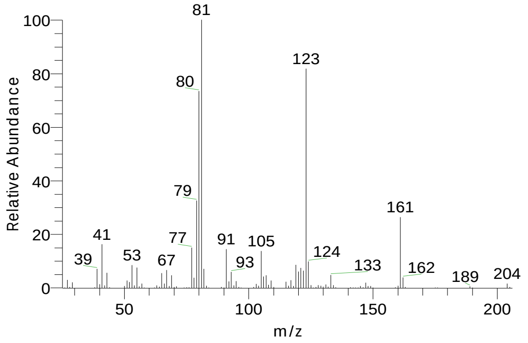

Formula C15H24, MW 204, CAS# 5208-59-3, Entry# 89478

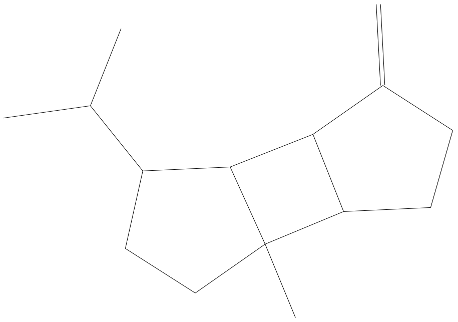

(-)-á-Bourbonene

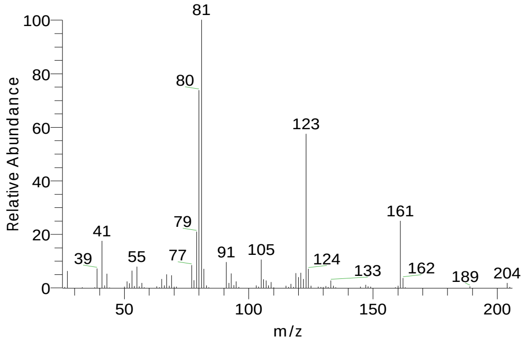

Formula C15H24, MW 204, CAS# 5208-59-3, Entry# 50341

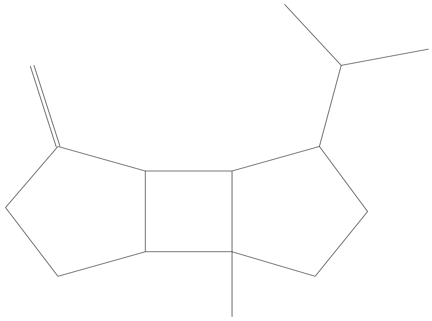

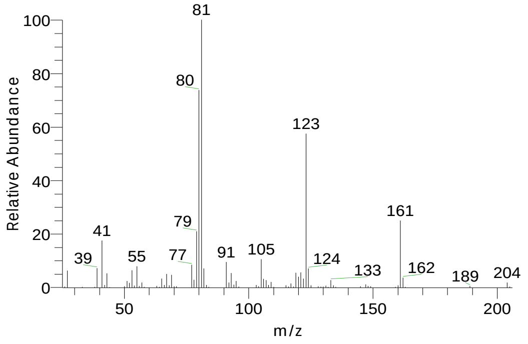

My GC-MS Report

Compound Structure | Hit Spectrum

Formula C15H24, MW 204, CAS# 5208-59-3, Entry# 89479

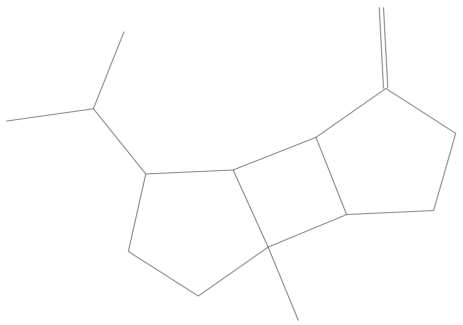

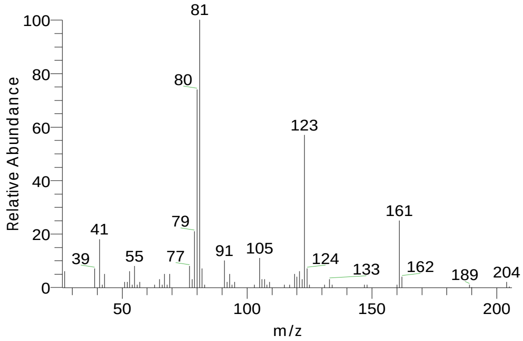

peppermint_oil #1974 RT: 10.62 AV: 1 NL: 1.11E8
T: + c EI Full ms [50.000-750.000]

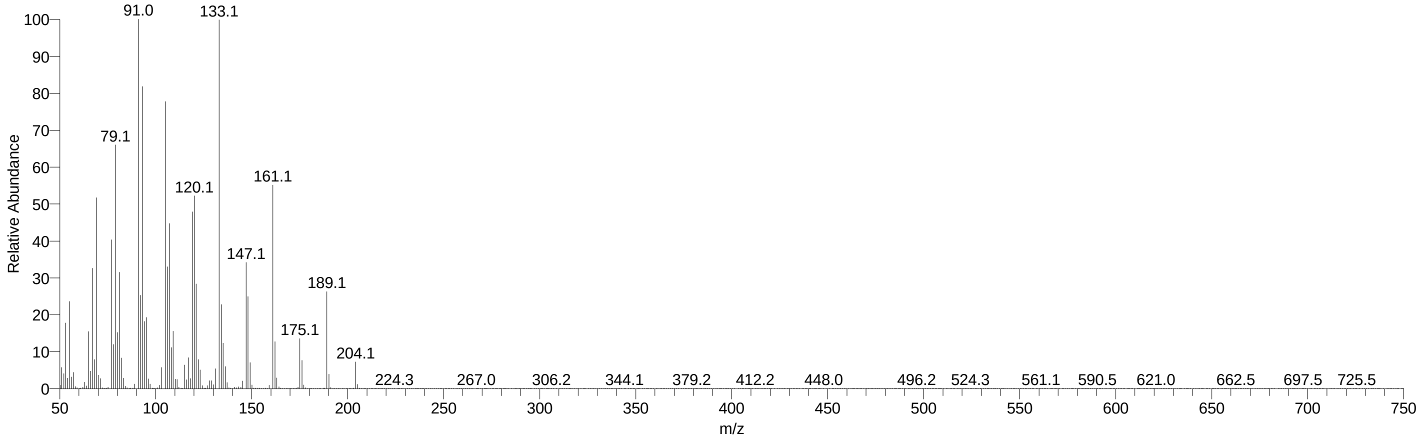

RT	Compound Name	Area %	MF	Molecular Formula	Molecular Weight	Cas #	Library
10.62	Caryophyllene	18.48	941	C15H24	204	87-44-5	mainlib
10.62	BICYCLO[7.2.0]UNDEC-4-ENE, 4,11,11-TRIMETHYL-8-METHYLENE-, [1R-(1R*,4E,9S*)]-	18.48	941	C15H24	204	87-44-5	WileyRegistry8e
10.62	BICYCLO[7.2.0]UNDEC-4-ENE, 4,11,11-TRIMETHYL-8-METHYLENE-, [1R-(1R*,4E,9S*)]-	18.48	932	C15H24	204	87-44-5	WileyRegistry8e
10.62	TRANS(á)-CARYOPHYLLENE	18.48	924	C15H24	204	NA	WileyRegistry8e
10.62	Isocaryophillene	18.48	918	C15H24	204	NA	mainlib

Compound Structure | Hit Spectrum

Caryophyllene
Formula C15H24, MW 204, CAS# 87-44-5, Entry# 66572
Bicyclo[7.2.0]undec-4-ene, 4,11,11-trimethyl-8-methylene-, [1R-(1R*,4E,9S*)]-

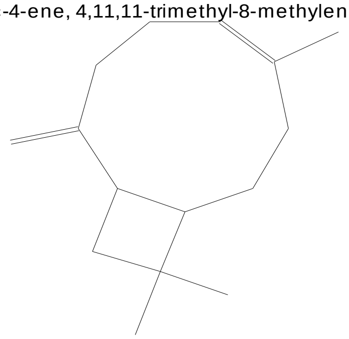

SI 941, RSI 941, mainlib, Entry# 66572, CAS# 87-44-5, Caryophyllene

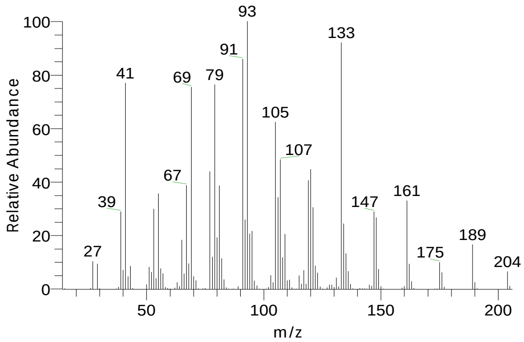

My GC-MS Report

Compound Structure | Hit Spectrum

BICYCLO[7.2.0]UNDEC-4-ENE, 4,11,11-TRIMETHYL-8-METHYLENE-, [1R-(1R*,4E,9S*)]-
Formula $C_{15}H_{24}$, MW 204, CAS# 87-44-5, Entry# 89162
2,6,10,10-TETRAMETHYLBICYCLO[7.2.0]UNDECA-1,6-DIENE #

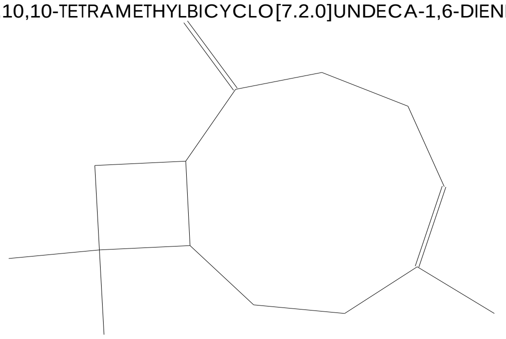

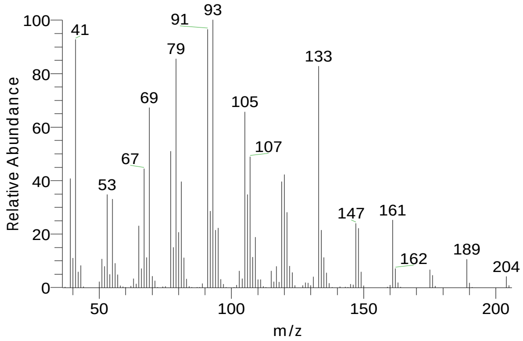

BICYCLO[7.2.0]UNDEC-4-ENE, 4,11,11-TRIMETHYL-8-METHYLENE-, [1R-(1R*,4E,9S*)]-
Formula $C_{15}H_{24}$, MW 204, CAS# 87-44-5, Entry# 89170
2,6,10,10-TETRAMETHYLBICYCLO[7.2.0]UNDECA-1,6-DIENE #

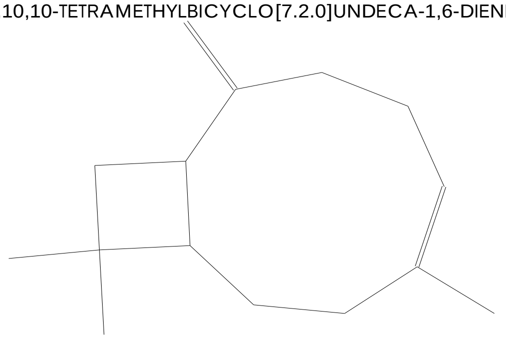

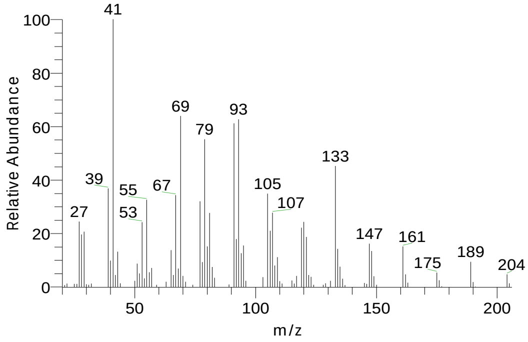

TRANS(á)-CARYOPHYLLENE
Formula $C_{15}H_{24}$, MW 204, CAS# NA, Entry# 89641

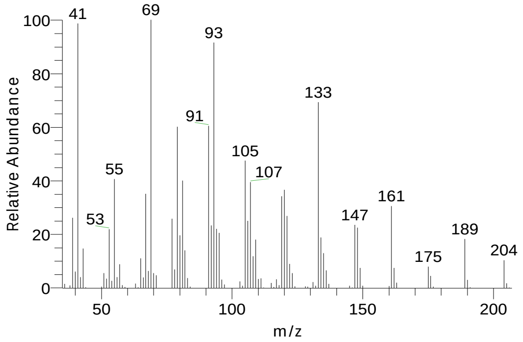

Isocaryophillene
Formula $C_{15}H_{24}$, MW 204, CAS# NA, Entry# 65988
4,11,11-Trimethyl-8-methylenebicyclo[7.2.0]undec-4-ene #

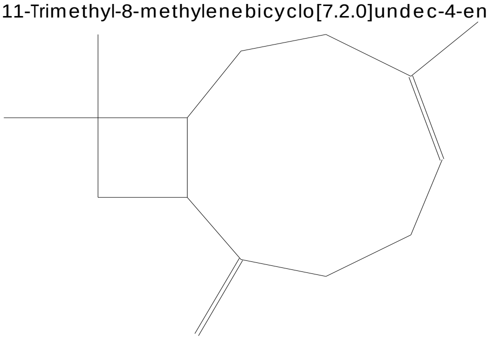

SI 913, RSI 918, mainlib, Entry# 65988, CAS# NA, Isocaryophillene

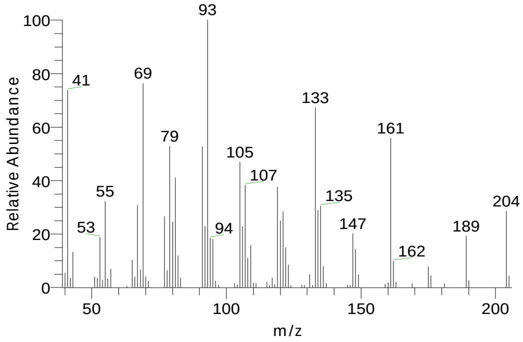

My GC-MS Report

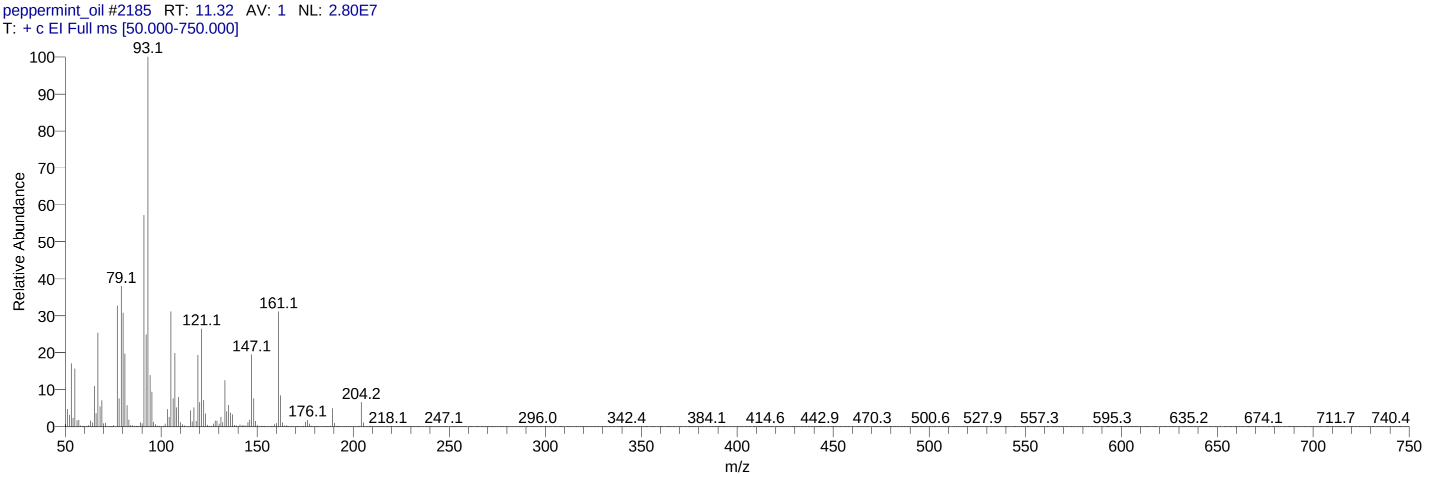

RT	Compound Name	Area %	MF	Molecular Formula	Molecular Weight	Cas #	Library
11.32	Humulene	1.41	887	C15H24	204	6753-98-6	replib
11.32	cis-à-Bisabolene	1.41	878	C15H24	204	29837-07-8	mainlib
11.32	1,4,8-CYCLOUNDECATRIENE, 2,6,6,9-TETRAMETHYL-, (E,E,E)-	1.41	864	C15H24	204	6753-98-6	WileyRegistry8e
11.32	NAPHTHALENE, 1,2,3,5,6,7,8,8A-OCTAHYDRO-1,8A-DIMETHYL-7-(1-METHYLETHENYL)-, [1R-(1à,7á,8Aà)]-	1.41	862	C15H24	204	4630-07-3	WileyRegistry8e
11.32	CYCLOHEXENE, 4-(1,5-DIMETHYL-1,4-HEXADIENYL)-1-METHYL-	1.41	878	C15H24	204	17627-44-0	WileyRegistry8e

Compound Structure	Hit Spectrum

Humulene
Formula C15H24, MW 204, CAS# 6753-98-6, Entry# 14003
à-Caryophyllene

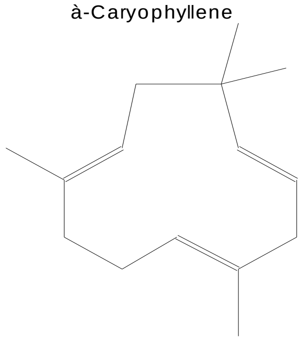

SI 868, RSI 887, replib, Entry# 14003, CAS# 6753-98-6, Humulene

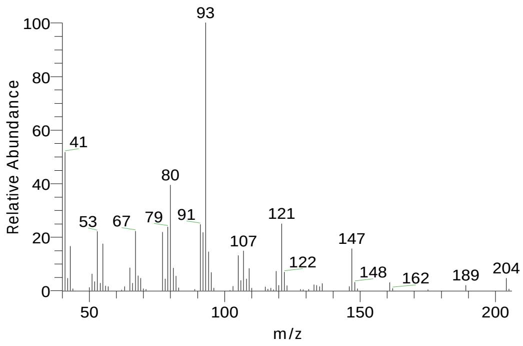

cis-à-Bisabolene
Formula C15H24, MW 204, CAS# 29837-07-8, Entry# 65792
4-[(1Z)-1,5-Dimethyl-1,4-hexadienyl]-1-methyl-1-cyclohexene

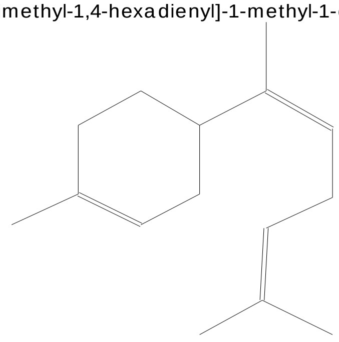

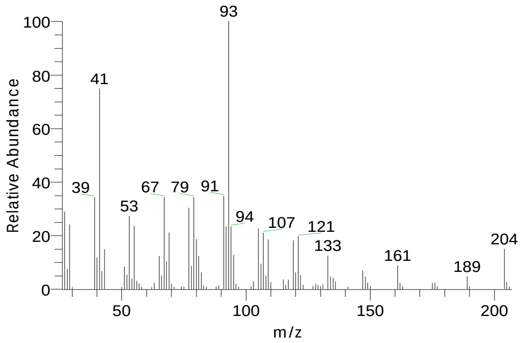

My GC-MS Report

Compound Structure | Hit Spectrum

1,4,8-CYCLOUNDECATRIENE, 2,6,6,9-TETRAMETHYL-, (E,E,E)-
Formula C15H24, MW 204, CAS# 6753-98-6, Entry# 89111
2,6,6,9-TETRAMETHYL-1,4,8-CYCLOUNDECATRIENE #

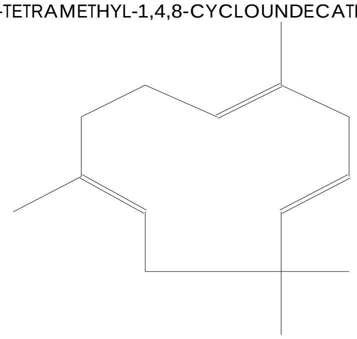

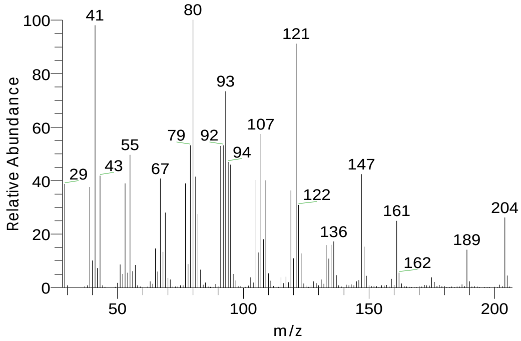

Formula C15H24, MW 204, CAS# 4630-07-3, Entry# 89318
3-ISOPROPENYL-4A,5-DIMETHYL-1,2,3,4,4A,5,6,7-OCTAHYDRONAPHTHALENE #

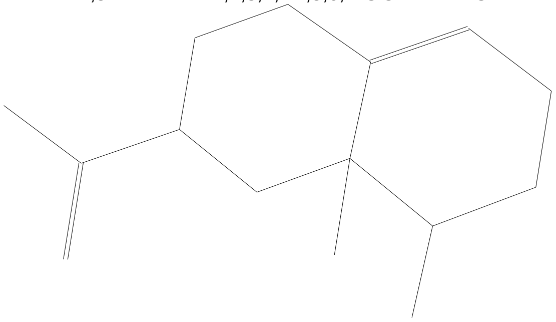

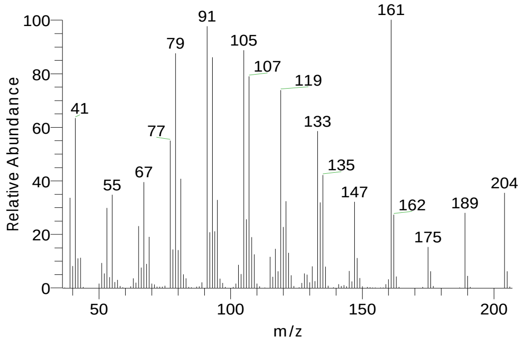

CYCLOHEXENE, 4-(1,5-DIMETHYL-1,4-HEXADIENYL)-1-METHYL-
Formula C15H24, MW 204, CAS# 17627-44-0, Entry# 89572
4-[(1E)-1,5-DIMETHYL-1,4-HEXADIENYL]-1-METHYL-1-CYCLOHEXENE #

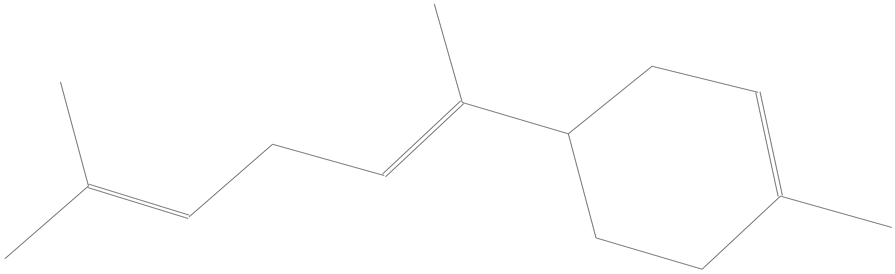

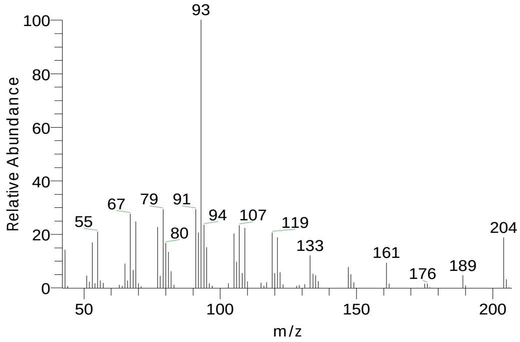

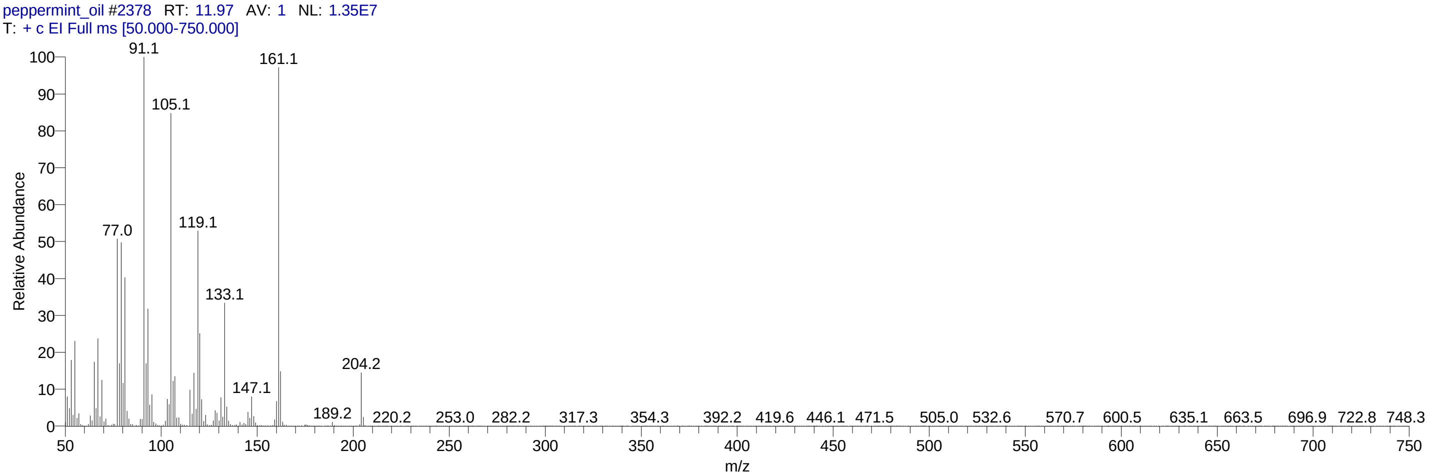

RT	Compound Name	Area %	MF	Molecular Formula	Molecular Weight	Cas #	Library
11.97	1,6-CYCLODECADIENE, 1-METHYL-5-METHYLENE-8-(1-METHYLETHYL)-, [S-(E,E)]-	0.88	951	C15H24	204	23986-74-5	WileyRegistry8e

My GC-MS Report

RT	Compound Name	Area %	MF	Molecular Formula	Molecular Weight	Cas #	Library
11.97	1,6-CYCLODECADIENE, 1-METHYL-5-METHYLENE-8-(1-METHYLETHYL)-, [S-(E,E)]-	0.88	934	C15H24	204	23986-74-5	WileyRegistry8e
11.97	Germacrene D	0.88	939	C15H24	204	23986-74-5	replib
11.97	Germacrene D	0.88	954	C15H24	204	23986-74-5	mainlib
11.97	1,6-CYCLODECADIENE, 1-METHYL-5-METHYLENE-8-(1-METHYLETHYL)-, [S-(E,E)]-	0.88	951	C15H24	204	23986-74-5	WileyRegistry8e

Compound Structure	Hit Spectrum

1,6-CYCLODECADIENE, 1-METHYL-5-METHYLENE-8-(1-METHYLETHYL)-, [S-(E,E)]-
Formula C15H24, MW 204, CAS# 23986-74-5, Entry# 89578
GERMACRA-1(10),4(15),5-TRIENE, (-)-

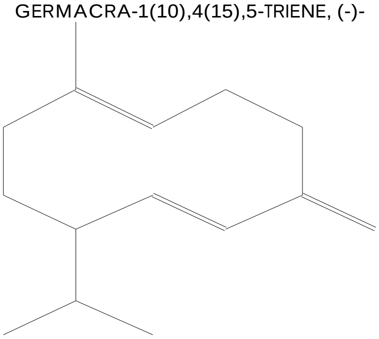

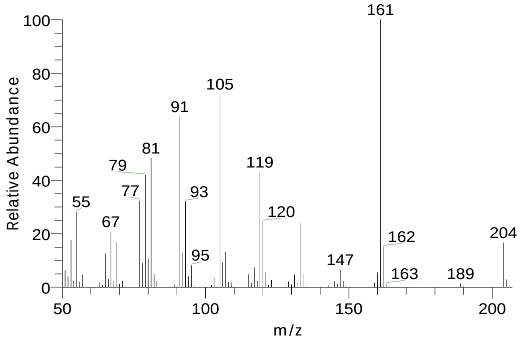

1,6-CYCLODECADIENE, 1-METHYL-5-METHYLENE-8-(1-METHYLETHYL)-, [S-(E,E)]-
Formula C15H24, MW 204, CAS# 23986-74-5, Entry# 89560
GERMACRA-1(10),4(15),5-TRIENE, (-)-

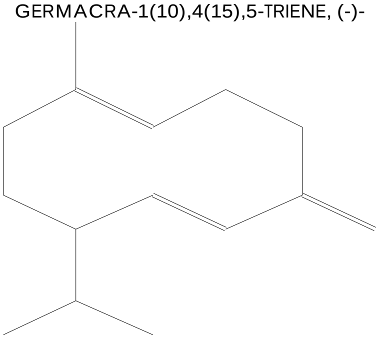

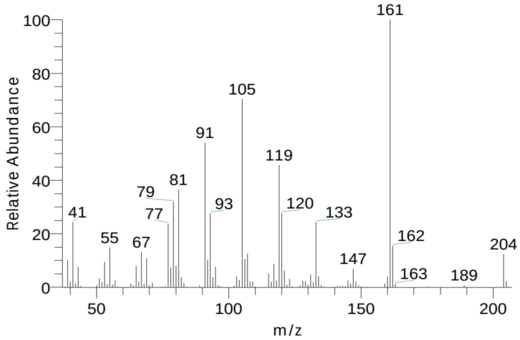

Germacrene D
Formula C15H24, MW 204, CAS# 23986-74-5, Entry# 24886
(S,1Z,6Z)-8-Isopropyl-1-methyl-5-methylenecyclodeca-1,6-diene

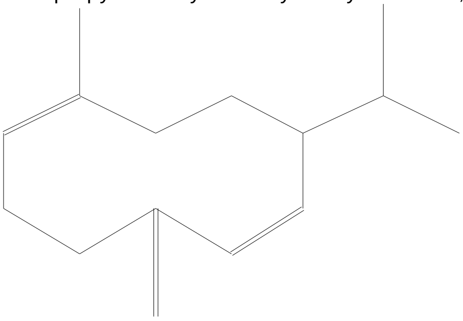

SI 921, RSI 939, replib, Entry# 24886, CAS# 23986-74-5, Germacrene D

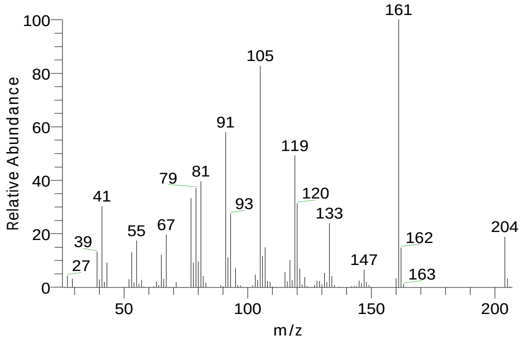

My GC-MS Report

Compound Structure | Hit Spectrum

Germacrene D
Formula C15H24, MW 204, CAS# 23986-74-5, Entry# 150487
(S,1Z,6Z)-8-Isopropyl-1-methyl-5-methylenecyclodeca-1,6-diene

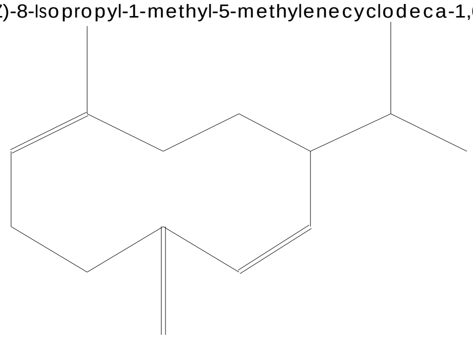

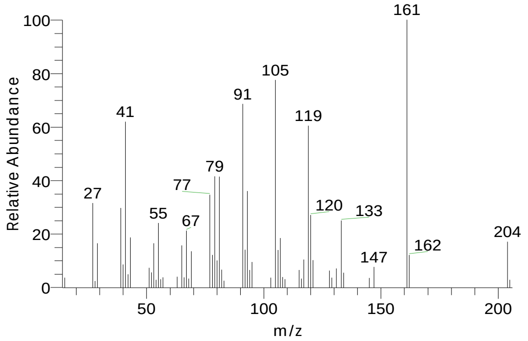

1,6-CYCLODECADIENE, 1-METHYL-5-METHYLENE-8-(1-METHYLETHYL)-, [S-(E,E)]-
Formula C15H24, MW 204, CAS# 23986-74-5, Entry# 362375
GERMACRA-1(10),4(15),5-TRIENE, (-)-

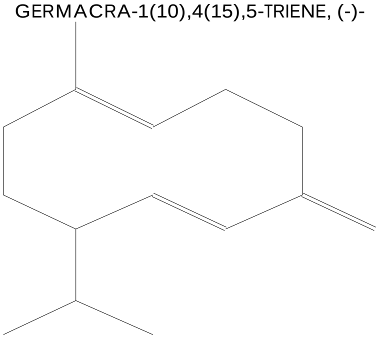

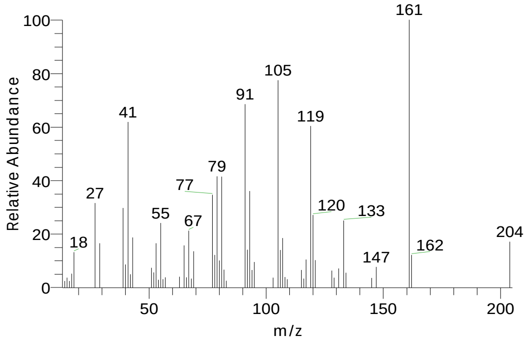

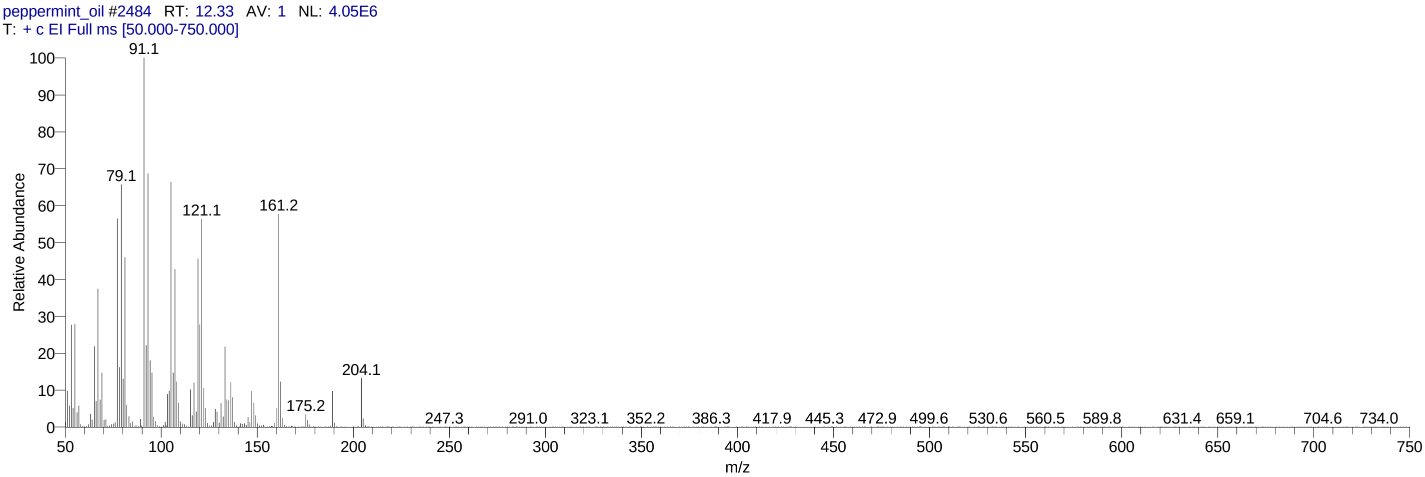

RT	Compound Name	Area %	MF	Molecular Formula	Molecular Weight	Cas #	Library
12.33	(S,1Z,6Z)-8-Isopropyl-1-methyl-5-methylenecyclodeca-1,6-diene	0.34	905	C15H24	204	317819-80-0	mainlib
12.33	Alloaromadendrene	0.34	914	C15H24	204	25246-27-9	replib
12.33	á-Longipinene	0.34	884	C15H24	204	41432-70-6	mainlib
12.33	NAPHTHALENE, 1,2,4A,5,6,8A-HEXAHYDRO-4,7-DIMETHYL-1-(1-METHYLETHYL)-, (1à,4Aà,8Aà)-	0.34	885	C15H24	204	31983-22-9	WileyRegistry8e
12.33	ç-Elemene	0.34	895	C15H24	204	29873-99-2	replib

My GC-MS Report

Compound Structure | Hit Spectrum

(S,1Z,6Z)-8-Isopropyl-1-methyl-5-methylenecyclodeca-1,6-diene
Formula C15H24, MW 204, CAS# 317819-80-0, Entry# 150394
1,6-Cyclodecadiene, 1-methyl-5-methylene-8-(1-methylethyl)-, (1Z,6Z,8S)-

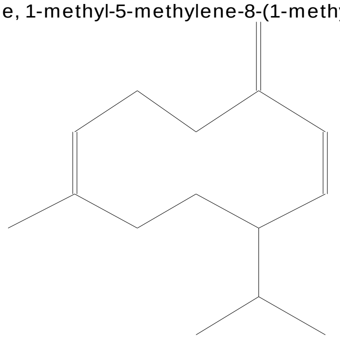

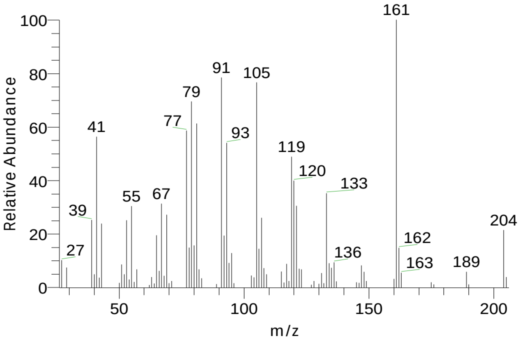

Alloaromadendrene
Formula C15H24, MW 204, CAS# 25246-27-9, Entry# 1320

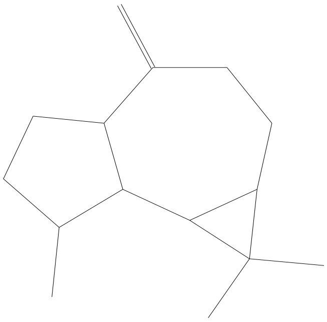

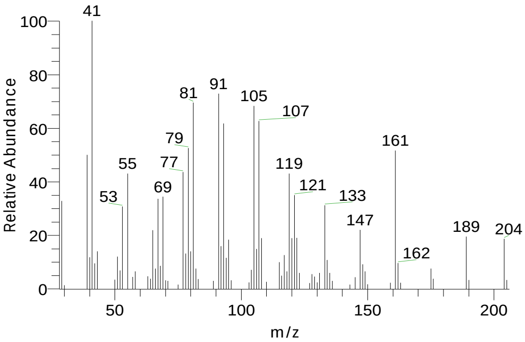

á-Longipinene
Formula C15H24, MW 204, CAS# 41432-70-6, Entry# 60945
(1S,2R,7S,8S)-2,6,6-Trimethyl-9-methylenetricyclo[5.4.0.02,8]undecane

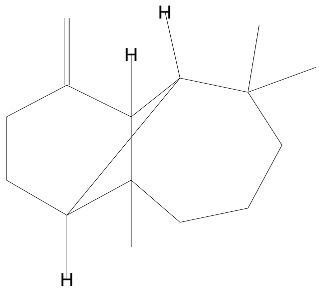

SI 875, RSI 884, mainlib, Entry# 60945, CAS# 41432-70-6, á-Longipinene

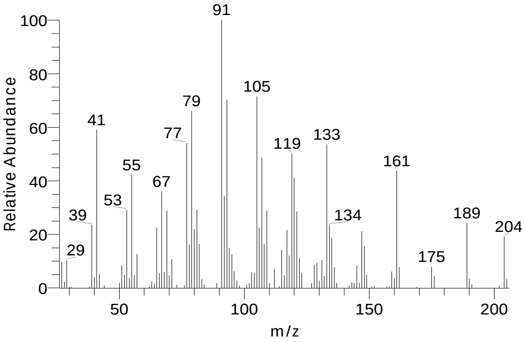

NAPHTHALENE, 1,2,4A,5,6,8A-HEXAHYDRO-4,7-DIMETHYL-1-(1-METHYLETHYL)-, (1à,4Aà,8Aà)-
Formula C15H24, MW 204, CAS# 31983-22-9, Entry# 89349
NAPHTHALENE, 1,2,4Aá,5,6,8Aá-HEXAHYDRO-1á-ISOPROPYL-4,7-DIMETHYL-

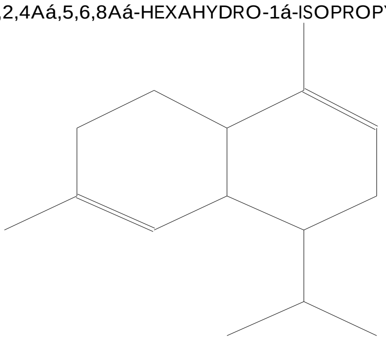

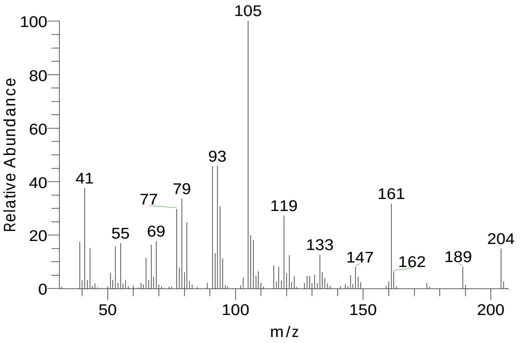

My GC-MS Report

Compound Structure

ç-Elemene
Formula C15H24, MW 204, CAS# 29873-99-2, Entry# 1378
1-Methyl-2-(1-methylethenyl)-4-(1-methylethylidene)-1-vinylcyclohexane), (1R-trans)-

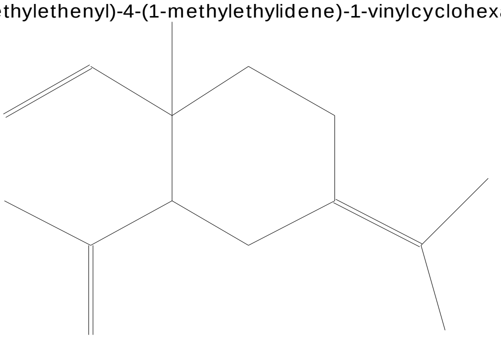

Hit Spectrum

SI 873, RSI 895, replib, Entry# 1378, CAS# 29873-99-2, ç-Elemene

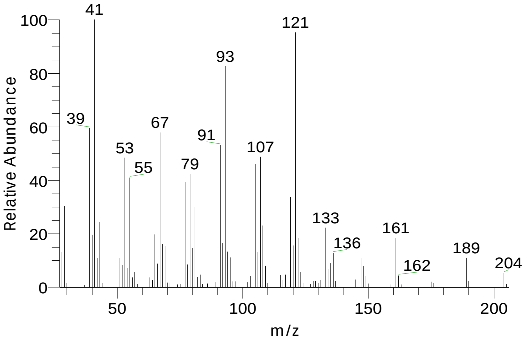

Supplement: Supplementary data 2 [file mmc2.pdf]
