# Supplementary material for: Larvicidal and adulticidal activity of essential oils from plants of the Lamiaceae family against the West Nile virus vector, Culex pipiens (Diptera: Culicidae)
Source: Saudi J Biol Sci. 2022 Jun 16;29(8):103350. doi: 10.1016/j.sjbs.2022.103350 (PMC9232543; doi:10.1016/j.sjbs.2022.103350)

My GC-MS Report

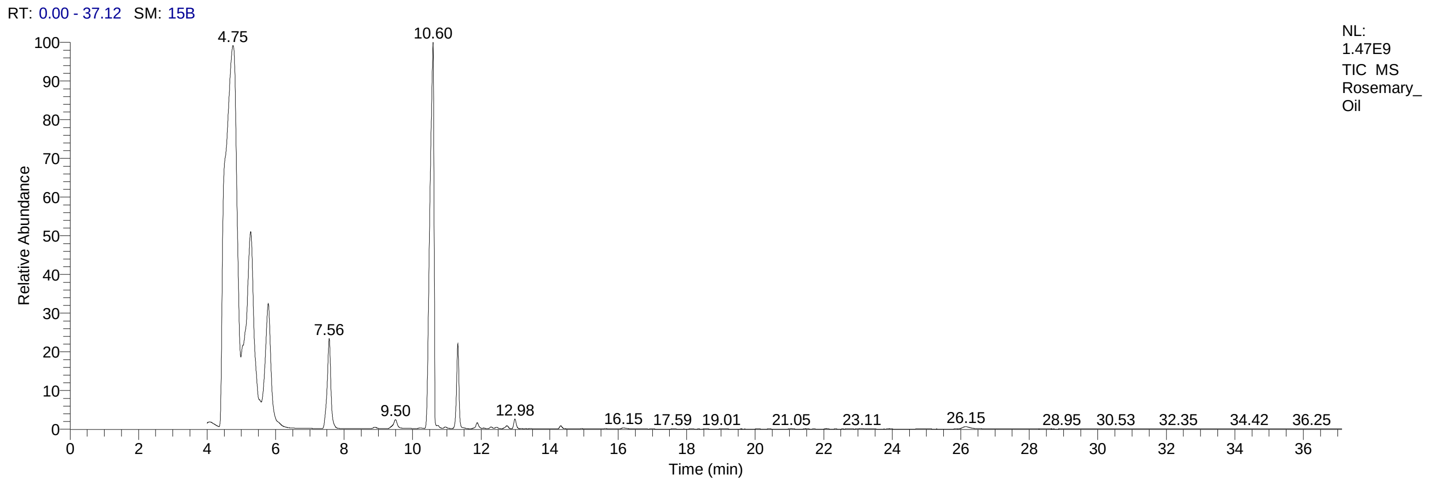

RT	Area %	Peak Area	Peak Height
4.74	56.43	25013820264.90	1129121917.45
5.02	0.12	55129962.68	21722588.01
5.27	7.16	3173657422.95	395266522.26
5.79	5.40	2395131674.20	321059292.84
7.57	3.69	1637296673.38	301132213.64
9.50	0.34	149689467.29	27714656.04
10.60	23.00	10192565723.37	1411154462.06
11.32	3.20	1418669492.20	344647332.02
11.88	0.26	115987575.21	23356318.14
12.98	0.39	171609677.11	39798533.75

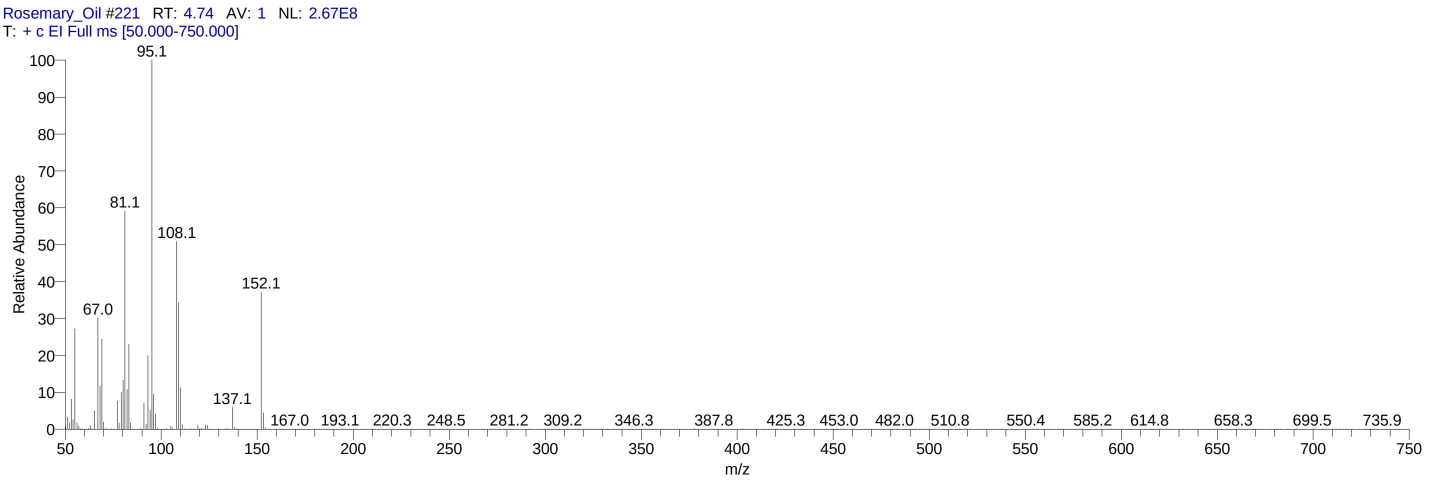

RT	Compound Name	Area %	MF	Molecular Formula	Molecular Weight	Cas #	Library
4.74	(+)-2-Bornanone	56.43	940	C10H16O	152	464-49-3	replib
4.74	BICYCLO[2.2.1]HEPTAN-2-ONE, 1,7,7-TRIMETHYL-	56.43	942	C10H16O	152	76-22-2	WileyRegistry8e
4.74	(+)-2-Bornanone	56.43	937	C10H16O	152	464-49-3	replib
4.74	Bicyclo[2.2.1]heptan-2-one, 1,7,7-trimethyl-, (1S)-	56.43	944	C10H16O	152	464-48-2	mainlib
4.74	BICYCLO[2.2.1]HEPTAN-2-ONE, 1,7,7-TRIMETHYL-	56.43	934	C10H16O	152	76-22-2	WileyRegistry8e

My GC-MS Report

Compound Structure | Hit Spectrum

(+)-2-Bornanone
Formula C10H16O, MW 152, CAS# 464-49-3, Entry# 14551
Bicyclo[2.2.1]heptan-2-one, 1,7,7-trimethyl-, (1R)-

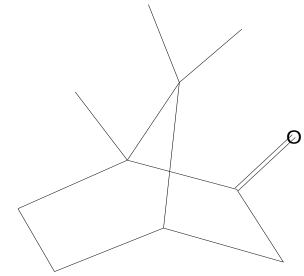

SI 939, RSI 940, replib, Entry# 14551, CAS# 464-49-3, (+)-2-Bornanone

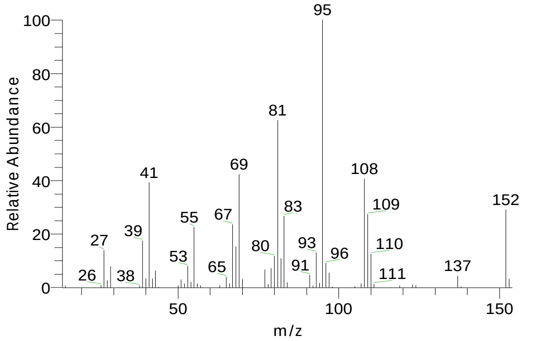

BICYCLO[2.2.1]HEPTAN-2-ONE, 1,7,7-TRIMETHYL-
Formula C10H16O, MW 152, CAS# 76-22-2, Entry# 36210
NORBORNAN-2-ONE

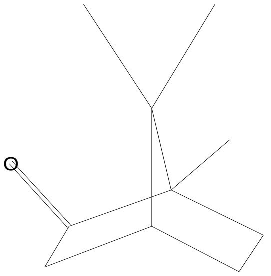

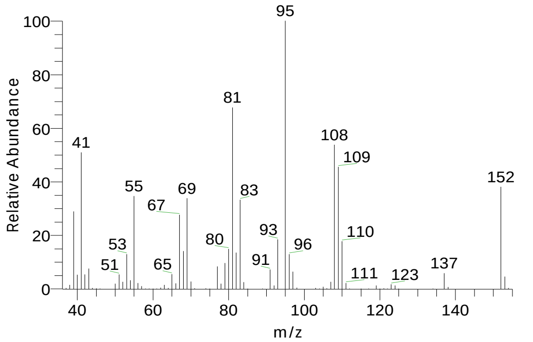

(+)-2-Bornanone
Formula C10H16O, MW 152, CAS# 464-49-3, Entry# 14554
Bicyclo[2.2.1]heptan-2-one, 1,7,7-trimethyl-, (1R)-

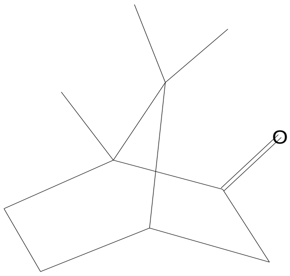

SI 937, RSI 937, replib, Entry# 14554, CAS# 464-49-3, (+)-2-Bornanone

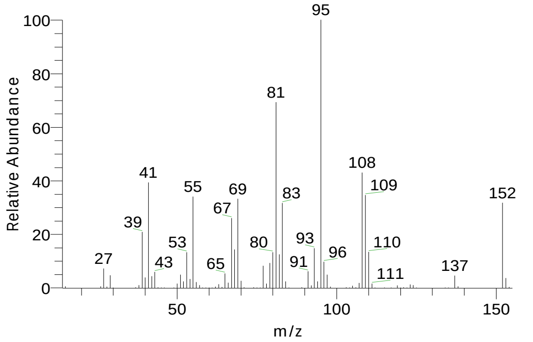

Bicyclo[2.2.1]heptan-2-one, 1,7,7-trimethyl-, (1S)-
Formula C10H16O, MW 152, CAS# 464-48-2, Entry# 68159
(-)-Alcanfor

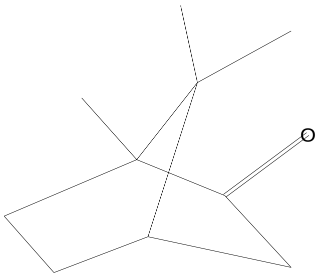

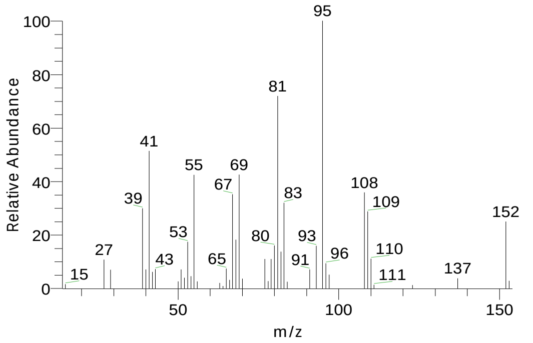

My GC-MS Report

Compound Structure | Hit Spectrum

BICYCLO[2.2.1]HEPTAN-2-ONE, 1,7,7-TRIMETHYL-
Formula C10H16O, MW 152, CAS# 76-22-2, Entry# 394883
NORBORNAN-2-ONE

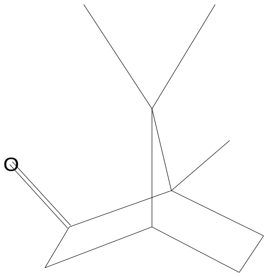

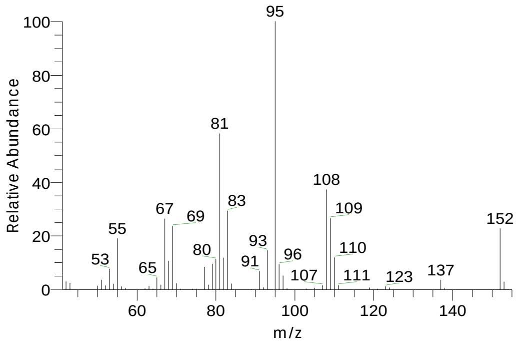

Rosemary_Oil #306 RT: 5.02 AV: 1 NL: 7.88E7
T: + c EI Full ms [50.000-750.000]

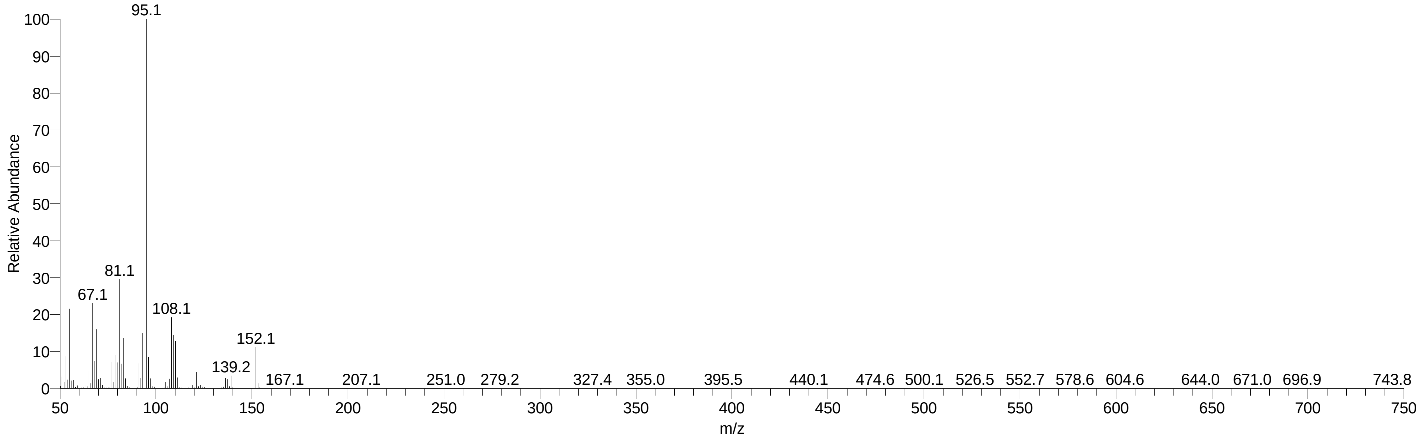

RT	Compound Name	Area %	MF	Molecular Formula	Molecular Weight	Cas #	Library
5.02	Bicyclo[2.2.1]heptan-2-one, 1,7,7-trimethyl-, (1S)-	0.12	919	C10H16O	152	464-48-2	mainlib
5.02	Camphor	0.12	862	C10H16O	152	76-22-2	replib
5.02	1,7,7-TRIMETHYL-BICYCLO[2.2.1]HEPTAN-2-ONE	0.12	862	C10H16O	152	NA	WileyRegistry8e
5.02	BICYCLO[2.2.1]HEPTAN-2-ONE, 1,7,7-TRIMETHYL-	0.12	868	C10H16O	152	76-22-2	WileyRegistry8e
5.02	Camphor	0.12	863	C10H16O	152	76-22-2	replib

Compound Structure | Hit Spectrum

Bicyclo[2.2.1]heptan-2-one, 1,7,7-trimethyl-, (1S)-
Formula C10H16O, MW 152, CAS# 464-48-2, Entry# 68159
(-)-Alcanfor

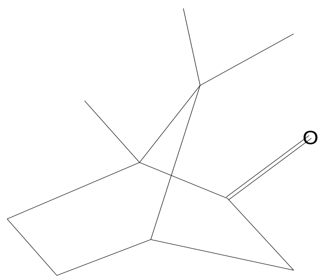

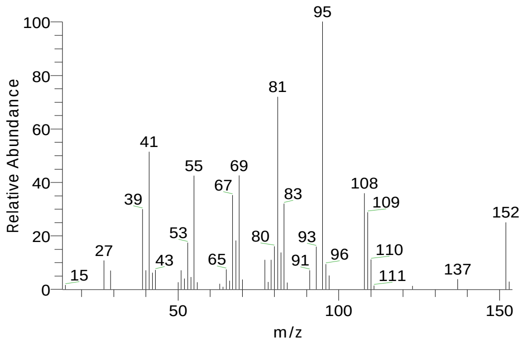

My GC-MS Report

Compound Structure | Hit Spectrum

Camphor
Formula C10H16O, MW 152, CAS# 76-22-2, Entry# 14454
Bicyclo[2.2.1]heptan-2-one, 1,7,7-trimethyl-

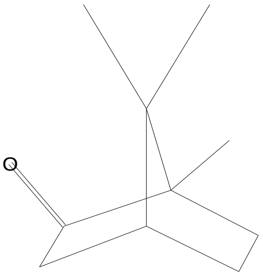

SI 850, RSI 862, replib, Entry# 14454, CAS# 76-22-2, Camphor

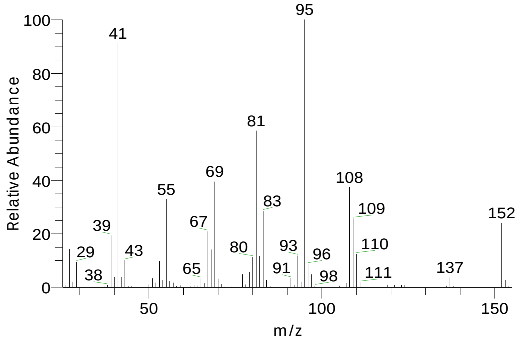

1,7,7-TRIMETHYL-BICYCLO[2.2.1]HEPTAN-2-ONE
Formula C10H16O, MW 152, CAS# NA, Entry# 371259
1,7,7-TRIMETHYLBICYCLO[2.2.1]HEPTAN-2-ONE

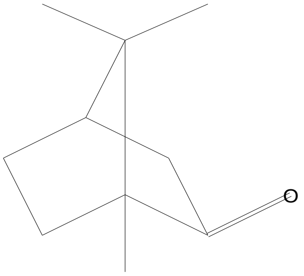

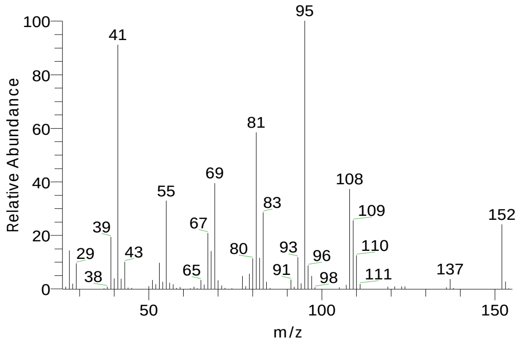

BICYCLO[2.2.1]HEPTAN-2-ONE, 1,7,7-TRIMETHYL-
Formula C10H16O, MW 152, CAS# 76-22-2, Entry# 394883
NORBORNAN-2-ONE

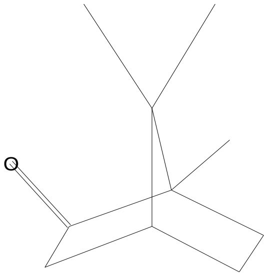

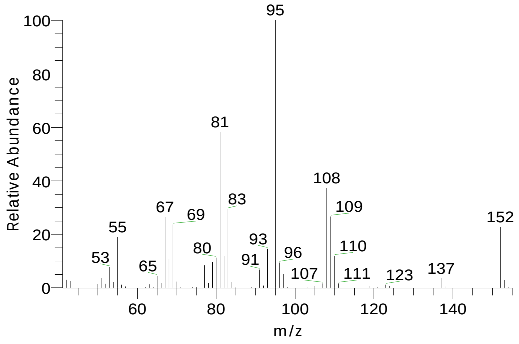

Camphor
Formula C10H16O, MW 152, CAS# 76-22-2, Entry# 14453
Bicyclo[2.2.1]heptan-2-one, 1,7,7-trimethyl-

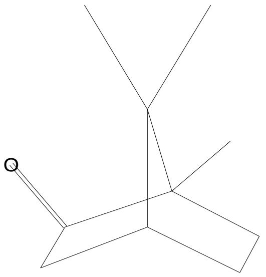

SI 849, RSI 863, replib, Entry# 14453, CAS# 76-22-2, Camphor

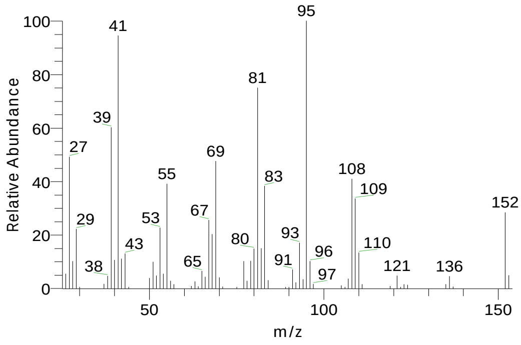

My GC-MS Report

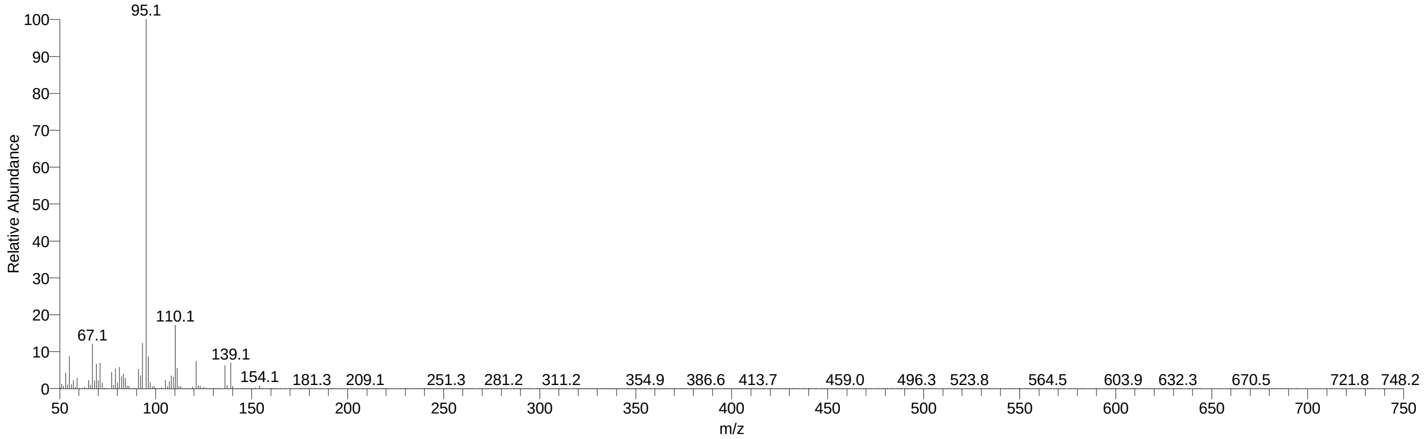

RT	Compound Name	Area %	MF	Molecular Formula	Molecular Weight	Cas #	Library
5.27	Isoborneol	7.16	935	C10H18O	154	124-76-5	replib
5.27	endo-Borneol	7.16	933	C10H18O	154	507-70-0	replib
5.27	endo-Borneol	7.16	931	C10H18O	154	507-70-0	mainlib
5.27	BICYCLO[2.2.1]HEPTAN-2-OL, 1,7,7-TRIMETHYL-, (1S-ENDO)-	7.16	926	C10H18O	154	464-45-9	WileyRegistry8e
5.27	Bicyclo[2.2.1]heptan-2-ol, 1,7,7-trimethyl-, (1S-endo)-	7.16	928	C10H18O	154	464-45-9	replib

Compound Structure

Hit Spectrum

Isoborneol
Formula C10H18O, MW 154, CAS# 124-76-5, Entry# 14437
Bicyclo[2.2.1]heptan-2-ol, 1,7,7-trimethyl-, exo-

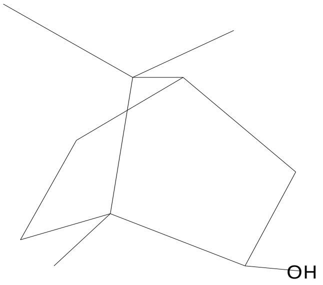

SI 935, RSI 935, replib, Entry# 14437, CAS# 124-76-5, Isoborneol

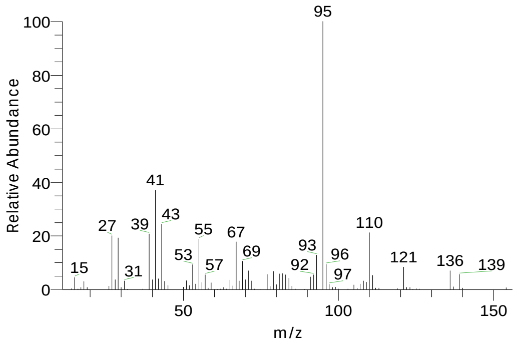

endo-Borneol
Formula C10H18O, MW 154, CAS# 507-70-0, Entry# 14458
endo-2-Hydroxy-1,7,7-trimethylnorbornane

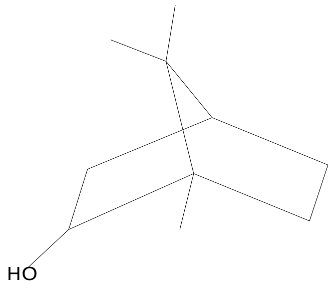

SI 931, RSI 933, replib, Entry# 14458, CAS# 507-70-0, endo-Borneol

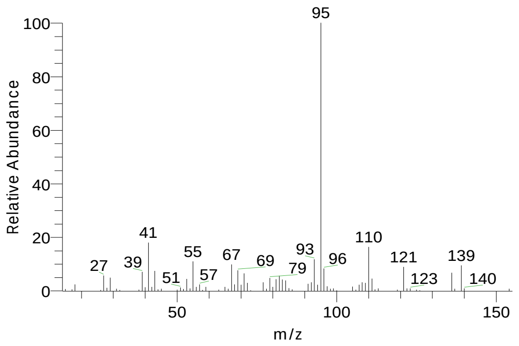

My GC-MS Report

Compound Structure | Hit Spectrum

endo-Borneol
Formula C10H18O, MW 154, CAS# 507-70-0, Entry# 68442
endo-2-Hydroxy-1,7,7-trimethylnorbornane

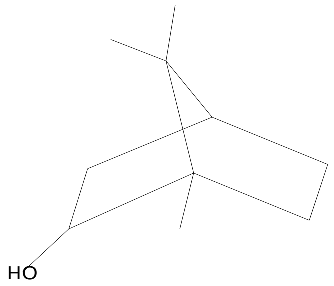

SI 931, RSI 931, mainlib, Entry# 68442, CAS# 507-70-0, endo-Borneol

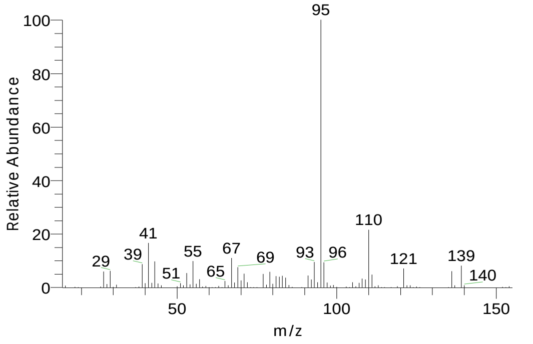

BICYCLO[2.2.1]HEPTAN-2-OL, 1,7,7-TRIMETHYL-, (1S-ENDO)-
Formula C10H18O, MW 154, CAS# 464-45-9, Entry# 38741
1,7,7-TRIMETHYLBICYCLO[2.2.1]HEPTAN-2-OL #

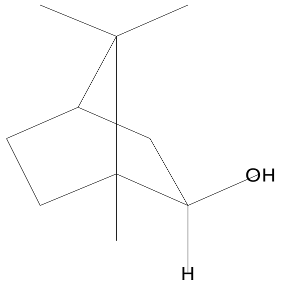

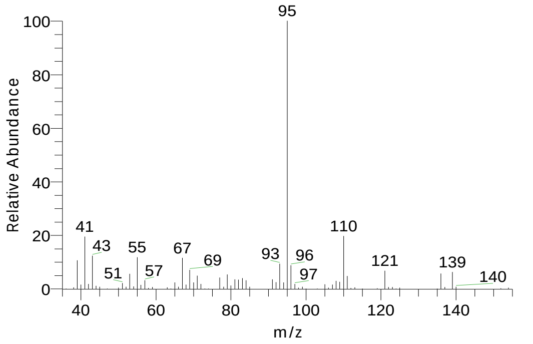

Bicyclo[2.2.1]heptan-2-ol, 1,7,7-trimethyl-, (1S-endo)-
Formula C10H18O, MW 154, CAS# 464-45-9, Entry# 14433
Borneol, (1S,2R,4S)-(-)-

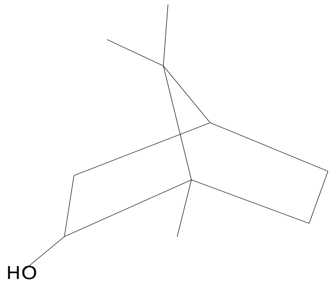

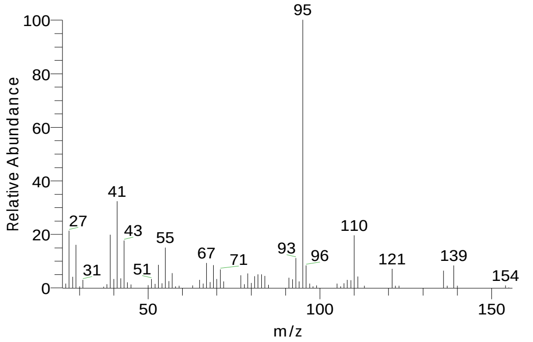

Rosemary_Oil #534 RT: 5.79 AV: 1 NL: 7.17E7
T: + c EI Full ms [50.000-750.000]

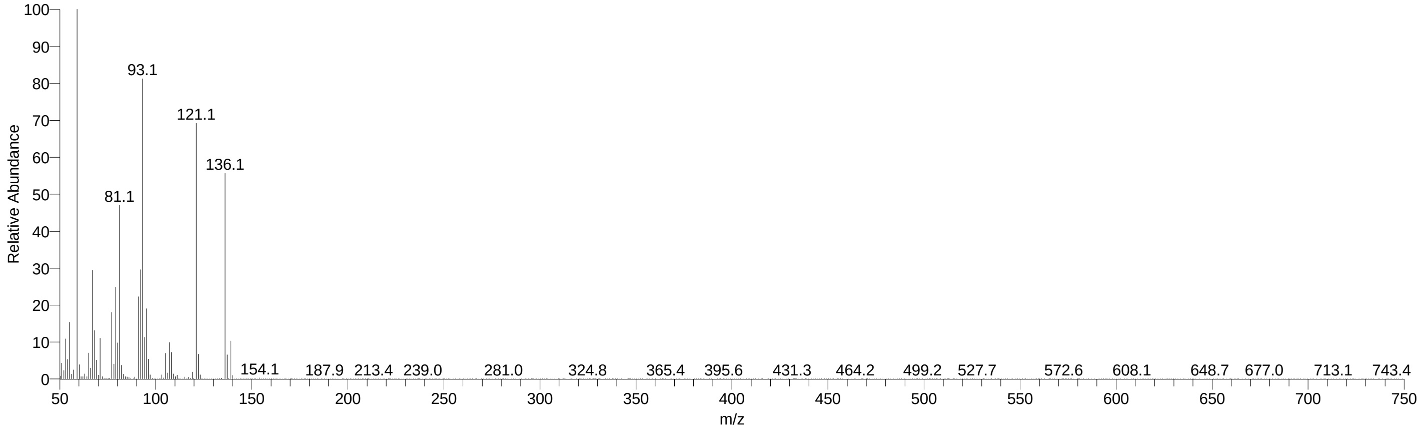

RT	Compound Name	Area %	MF	Molecular Formula	Molecular Weight	Cas #	Library
5.79	(-)-(1S,2R,4R)-BETA-FENCHOL	5.40	937	C10H18O	154	470-08-6	WileyRegistry8e
5.79	3-CYCLOHEXENE-1-METHANOL, à,à,4-TRIMETHYL-	5.40	927	C10H18O	154	98-55-5	WileyRegistry8e

My GC-MS Report

RT	Compound Name	Area %	MF	Molecular Formula	Molecular Weight	Cas #	Library
5.79	3-CYCLOHEXENE-1-METHANOL , à,à,4-TRIMETHYL-	5.40	926	C10H18O	154	98-55-5	WileyRegistry8e
5.79	3-CYCLOHEXENE-1-METHANOL , à,à,4-TRIMETHYL-, (S)-	5.40	928	C10H18O	154	10482-56-1	WileyRegistry8e
5.79	1,6-OCTADIEN-3-OL, 3,7-DIMETHYL-, PROPANOATE	5.40	931	C13H22O2	210	144-39-8	WileyRegistry8e

Compound Structure | Hit Spectrum

(-)-(1S,2R,4R)-BETA-FENCHOL
Formula C10H18O, MW 154, CAS# 470-08-6, Entry# 38737
(-)-BETA-FENCHOL

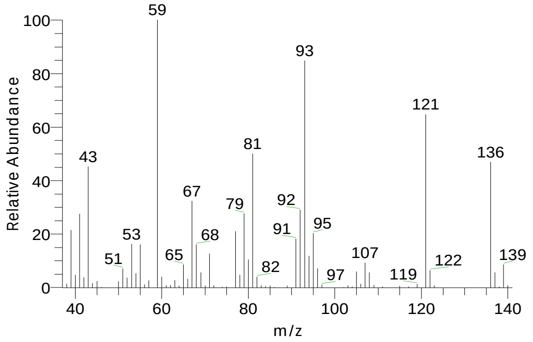

3-CYCLOHEXENE-1-METHANOL, à,à,4-TRIMETHYL-
Formula C10H18O, MW 154, CAS# 98-55-5, Entry# 38742
3-CYCLOHEXENE-1-METHANOL, à,à,4-TRIMETHYL-, (S)-

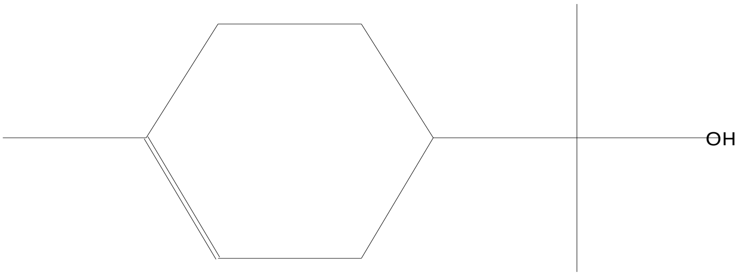

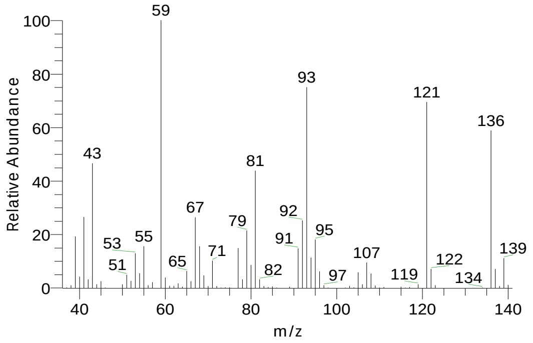

3-CYCLOHEXENE-1-METHANOL, à,à,4-TRIMETHYL-
Formula C10H18O, MW 154, CAS# 98-55-5, Entry# 38365
3-CYCLOHEXENE-1-METHANOL, à,à,4-TRIMETHYL-, (S)-

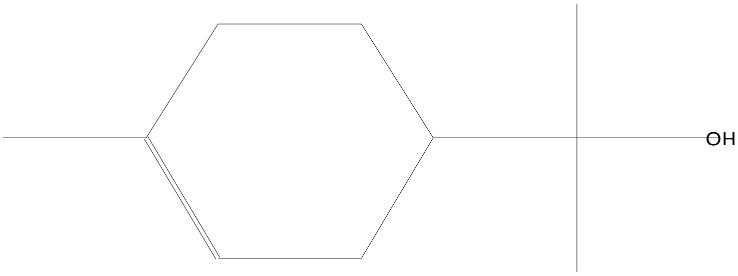

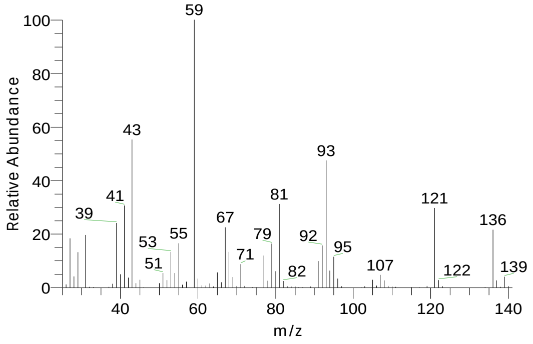

3-CYCLOHEXENE-1-METHANOL, à,à,4-TRIMETHYL-, (S)-
Formula C10H18O, MW 154, CAS# 10482-56-1, Entry# 38380
2-(4-METHYL-3-CYCLOHEXEN-1-YL)-2-PROPANOL #

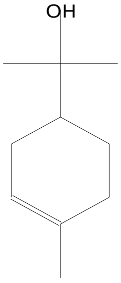

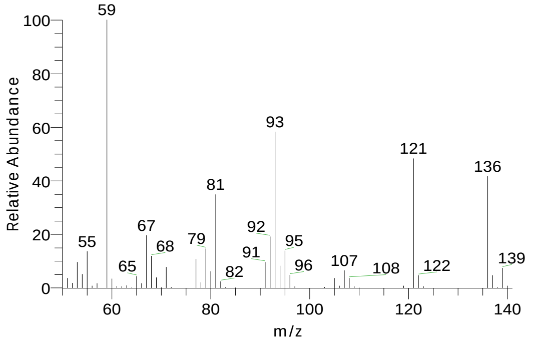

My GC-MS Report

Compound Structure | Hit Spectrum

1,6-OCTADIEN-3-OL, 3,7-DIMETHYL-, PROPANOATE
Formula C13H22O2, MW 210, CAS# 144-39-8, Entry# 96030
1,5-DIMETHYL-1-VINYL-4-HEXENYL PROPIONATE #

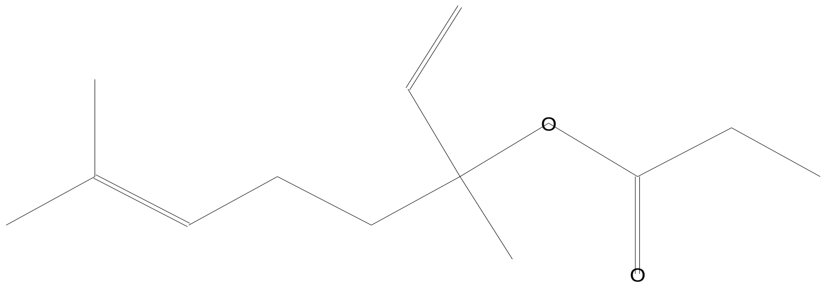

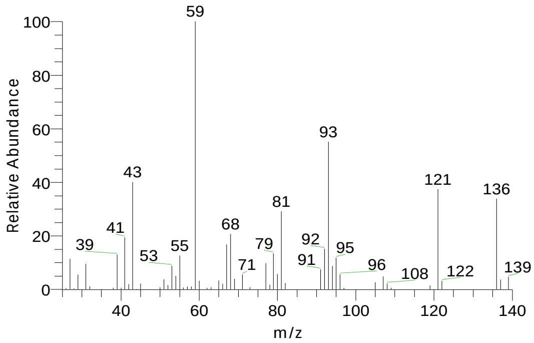

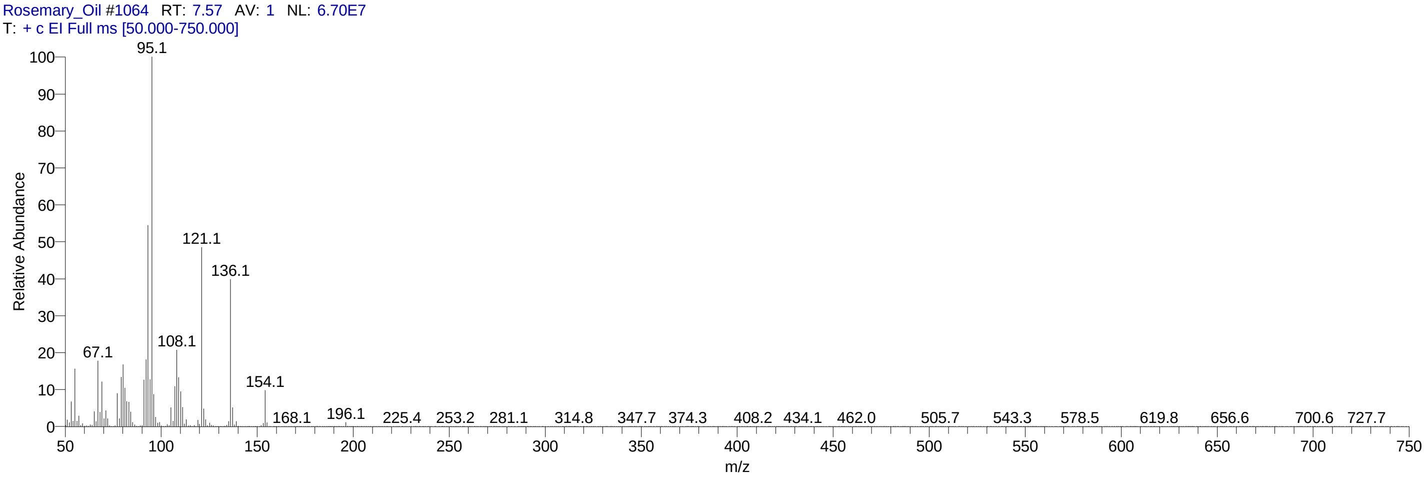

RT	Compound Name	Area %	MF	Molecular Formula	Molecular Weight	Cas #	Library
7.57	BICYCLO[2.2.1]HEPTAN-2-OL, 1,7,7-TRIMETHYL-, ACETATE, (1S-ENDO)-	3.69	953	C12H20O2	196	5655-61-8	WileyRegistry8e
7.57	BICYCLO[2.2.1]HEPTAN-2-OL, 1,7,7-TRIMETHYL-, ACETATE, ENDO-	3.69	945	C12H20O2	196	76-49-3	WileyRegistry8e
7.57	Bornyl acetate	3.69	943	C12H20O2	196	76-49-3	mainlib
7.57	Bicyclo[2.2.1]heptan-2-ol, 1,7,7-trimethyl-, acetate, (1S-endo)-	3.69	938	C12H20O2	196	5655-61-8	mainlib
7.57	BICYCLO[2.2.1]HEPTAN-2-OL, 1,7,7-TRIMETHYL-, ACETATE, EXO-	3.69	936	C12H20O2	196	125-12-2	WileyRegistry8e

Compound Structure | Hit Spectrum

BICYCLO[2.2.1]HEPTAN-2-OL, 1,7,7-TRIMETHYL-, ACETATE, (1S-ENDO)-
Formula C12H20O2, MW 196, CAS# 5655-61-8, Entry# 80072
1,7,7-TRIMETHYLBICYCLO[2.2.1]HEPT-2-YL ACETATE #

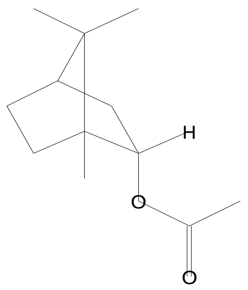

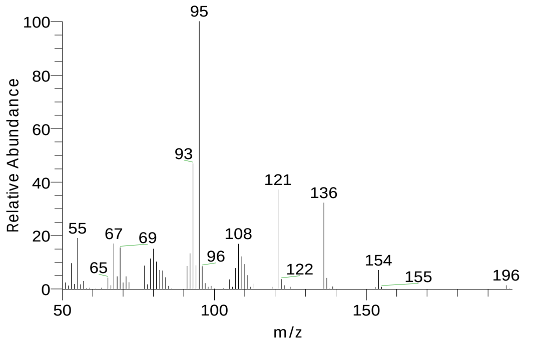

My GC-MS Report

Compound Structure | Hit Spectrum

BICYCLO[2.2.1]HEPTAN-2-OL, 1,7,7-TRIMETHYL-, ACETATE, ENDO-
Formula C12H20O2, MW 196, CAS# 76-49-3, Entry# 80076
1,7,7-TRIMETHYLBICYCLO[2.2.1]HEPT-2-YL ACETATE #

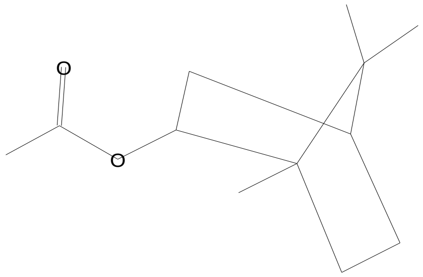

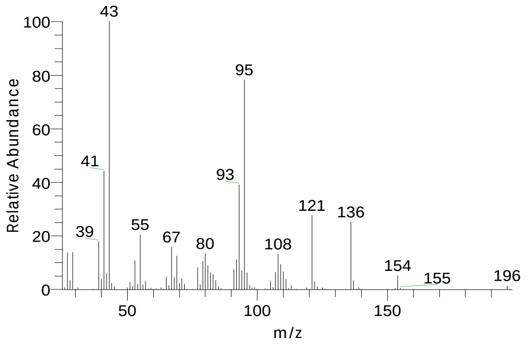

Bornyl acetate
Formula C12H20O2, MW 196, CAS# 76-49-3, Entry# 67808
Bicyclo[2.2.1]heptan-2-ol, 1,7,7-trimethyl-, acetate, endo-

SI 942, RSI 943, mainlib, Entry# 67808, CAS# 76-49-3, Bornyl acetate

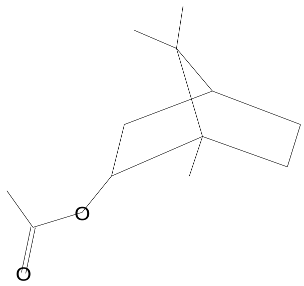

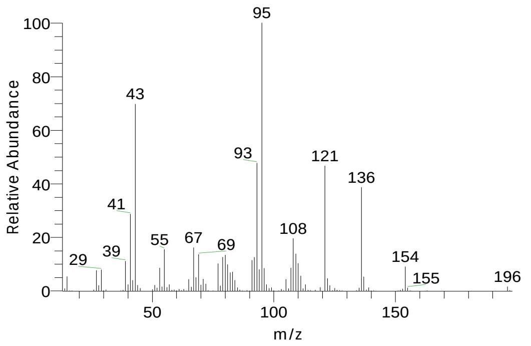

Bicyclo[2.2.1]heptan-2-ol, 1,7,7-trimethyl-, acetate, (1S-endo)-
Formula C12H20O2, MW 196, CAS# 5655-61-8, Entry# 68273
Borneol, acetate, (1S,2R,4S)-(-)-

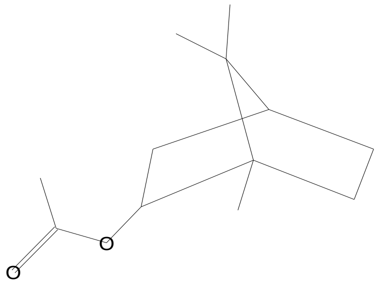

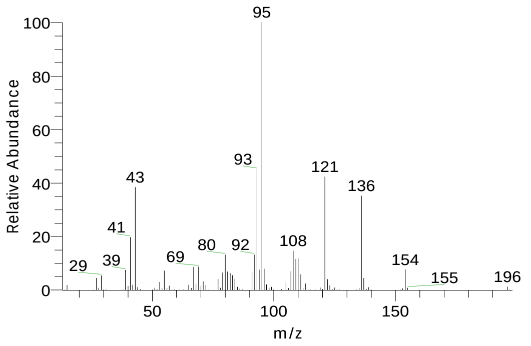

BICYCLO[2.2.1]HEPTAN-2-OL, 1,7,7-TRIMETHYL-, ACETATE, EXO-
Formula C12H20O2, MW 196, CAS# 125-12-2, Entry# 80091
1,7,7-TRIMETHYLBICYCLO[2.2.1]HEPT-2-YL ACETATE #

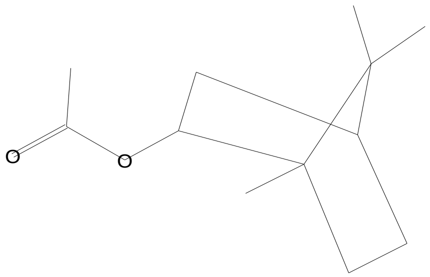

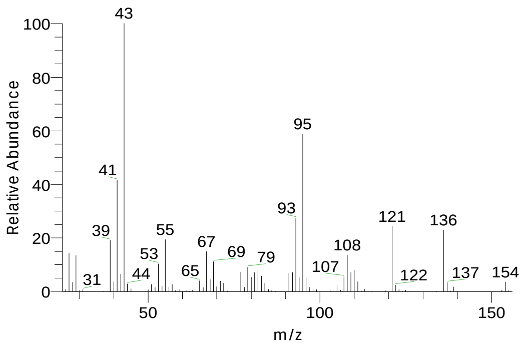

My GC-MS Report

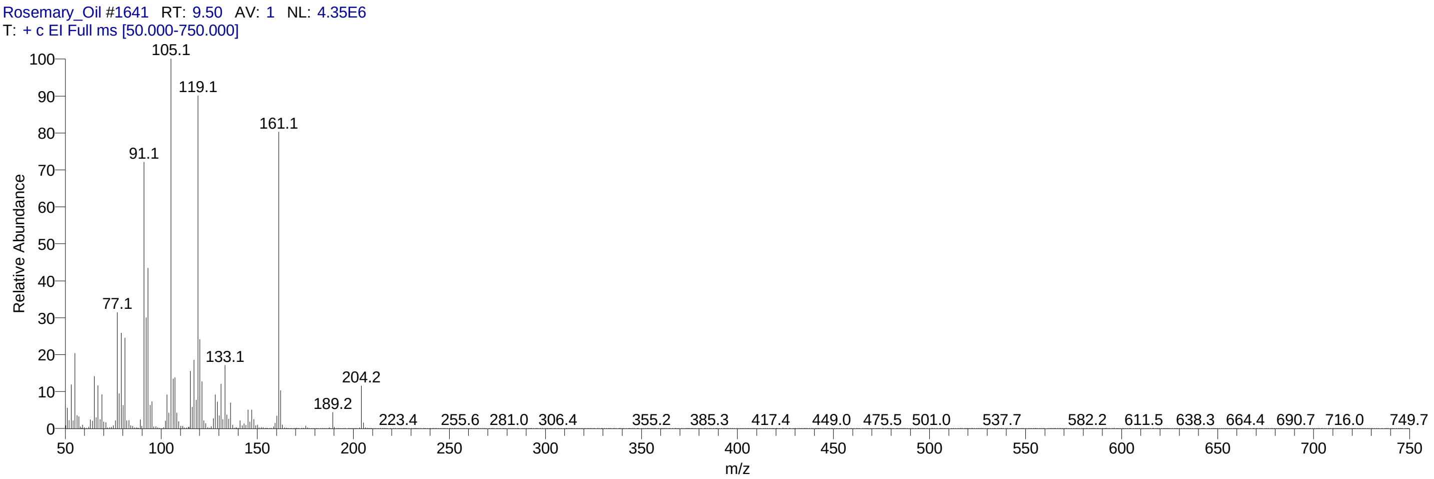

RT	Compound Name	Area %	MF	Molecular Formula	Molecular Weight	Cas #	Library
9.50	.alfa.-Copaene	0.34	928	C15H24	204	NA	mainlib
9.50	TRICYCLO[4.4.0.0(2,7)]DEC-3-ENE, 1,3-DIMETHYL-8-(1-METHYLETHYL)-, ST	0.34	941	C15H24	204	3856-25-5	WileyRegistry8e
9.50	TRICYCLO[4.4.0.0(2,7)]DEC-3-ENE, 1,3-DIMETHYL-8-(1-METHYLETHYL)-, ST	0.34	913	C15H24	204	3856-25-5	WileyRegistry8e
9.50	Copaene	0.34	911	C15H24	204	3856-25-5	replib
9.50	TRICYCLO[4.4.0.0(2,7)]DEC-3-ENE, 1,3-DIMETHYL-8-(1-METHYLETHYL)-, ST	0.34	909	C15H24	204	3856-25-5	WileyRegistry8e

Compound Structure

Hit Spectrum

.alfa.-Copaene
Formula C15H24, MW 204, CAS# NA, Entry# 150578
$:28VLXDPFLIRFYIME-UHFFFAOYSA-N

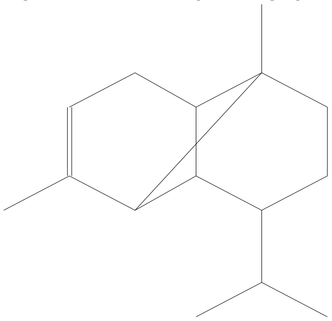

SI 921, RSI 928, mainlib, Entry# 150578, CAS# NA, .alfa.-Copaene

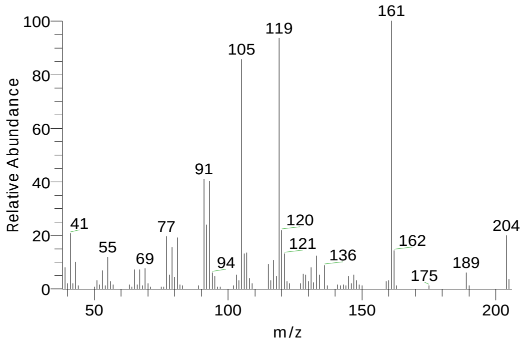

TRICYCLO[4.4.0.0(2,7)]DEC-3-ENE, 1,3-DIMETHYL-8-(1-METHYLETHYL)-, ST
Formula C15H24, MW 204, CAS# 3856-25-5, Entry# 89462
TRICYCLO[4.4.0.0(2,7)]DEC-3-ENE, 1,3-DIMETHYL-8-(1-METHYLETHYL)-, STEREOISOMER

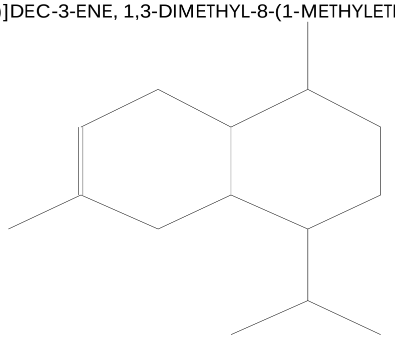

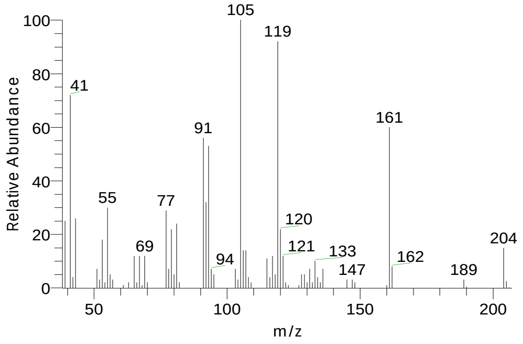

My GC-MS Report

Compound Structure	Hit Spectrum

TRICYCLO[4.4.0.0(2,7)]DEC-3-ENE, 1,3-DIMETHYL-8-(1-METHYLETHYL)-, ST
Formula C15H24, MW 204, CAS# 3856-25-5, Entry# 89460
TRICYCLO[4.4.0.0(2,7)]DEC-3-ENE, 1,3-DIMETHYL-8-(1-METHYLETHYL)-, STEREOISOMER

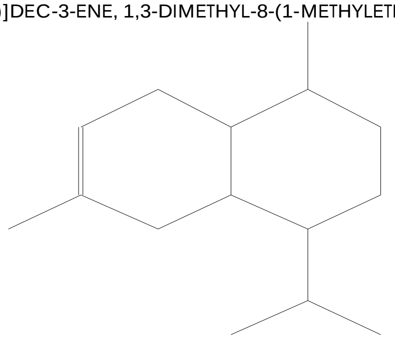

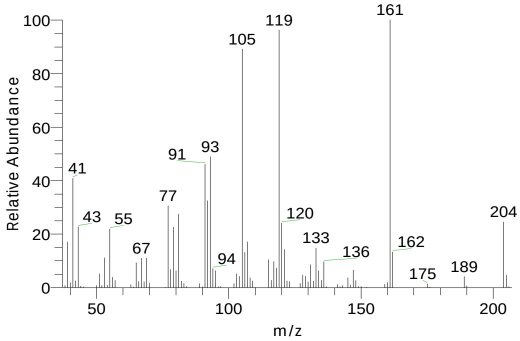

Copaene
Formula C15H24, MW 204, CAS# 3856-25-5, Entry# 18672
Tricyclo[4.4.0.02,7]dec-3-ene, 1,3-dimethyl-8-(1-methylethyl)-, stereoisomer

SI 905, RSI 911, replib, Entry# 18672, CAS# 3856-25-5, Copaene

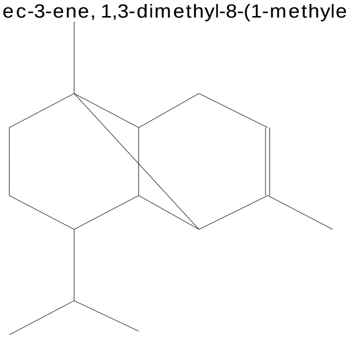

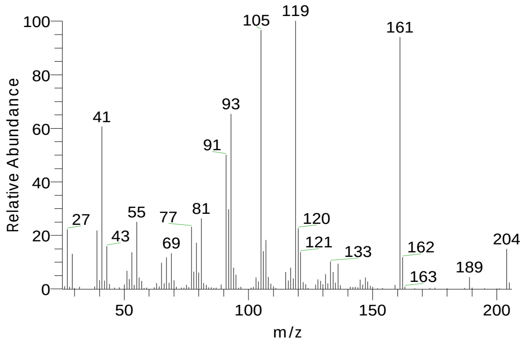

TRICYCLO[4.4.0.0(2,7)]DEC-3-ENE, 1,3-DIMETHYL-8-(1-METHYLETHYL)-, ST
Formula C15H24, MW 204, CAS# 3856-25-5, Entry# 89461
TRICYCLO[4.4.0.0(2,7)]DEC-3-ENE, 1,3-DIMETHYL-8-(1-METHYLETHYL)-, STEREOISOMER

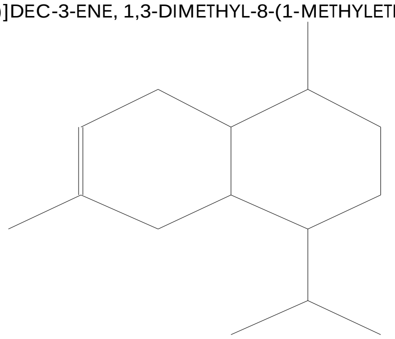

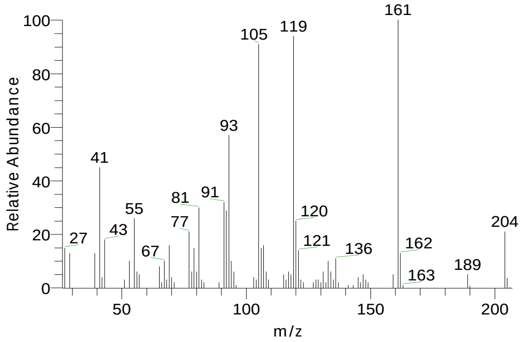

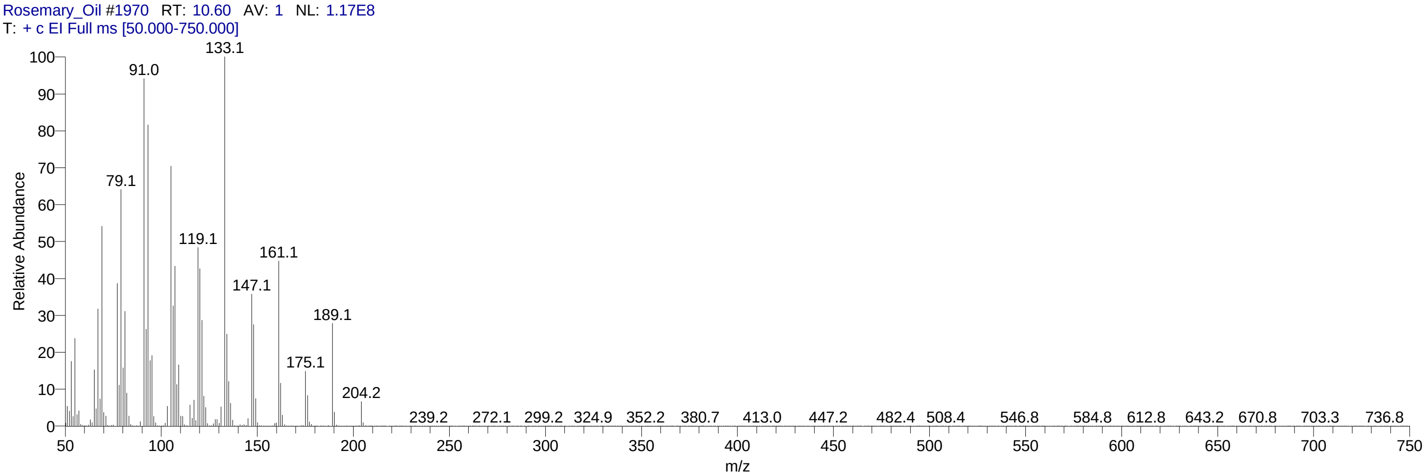

RT	Compound Name	Area %	MF	Molecular Formula	Molecular Weight	Cas #	Library
10.60	Caryophyllene	23.00	947	C15H24	204	87-44-5	mainlib
10.60	BICYCLO[7.2.0]UNDEC-4-ENE, 4,11,11-TRIMETHYL-8-METHYLENE-, [1R-(1R*,4E,9S*)]-	23.00	942	C15H24	204	87-44-5	WileyRegistry8e

My GC-MS Report

RT	Compound Name	Area %	MF	Molecular Formula	Molecular Weight	Cas #	Library
10.60	TRANS(á)-CARYOPHYLLENE	23.00	932	C15H24	204	NA	WileyRegistry8e
10.60	BICYCLO[7.2.0]UNDEC-4-ENE, 4,11,11-TRIMETHYL-8-METHYLENE-, [1R-(1R*,4E,9S*)]-	23.00	932	C15H24	204	87-44-5	WileyRegistry8e
10.60	BICYCLO[7.2.0]UNDEC-4-ENE, 4,11,11-TRIMETHYL-8-METHYLENE-, [1R-(1R*,4E,9S*)]-	23.00	923	C15H24	204	87-44-5	WileyRegistry8e

Compound Structure	Hit Spectrum

Caryophyllene
Formula C15H24, MW 204, CAS# 87-44-5, Entry# 66572
Bicyclo[7.2.0]undec-4-ene, 4,11,11-trimethyl-8-methylene-, [1R-(1R*,4E,9S*)]-

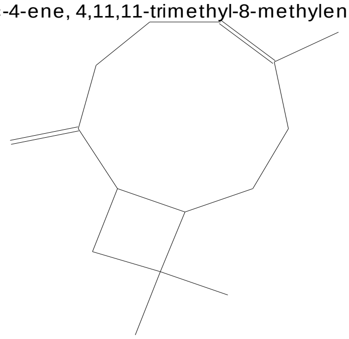

SI 947, RSI 947, mainlib, Entry# 66572, CAS# 87-44-5, Caryophyllene

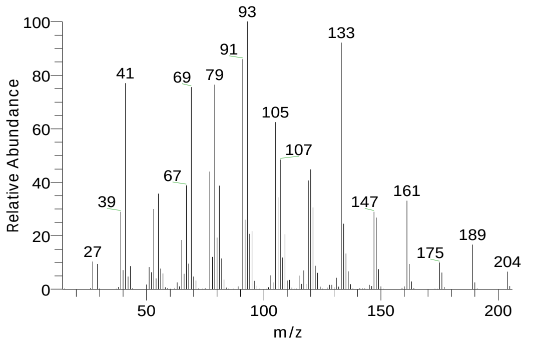

BICYCLO[7.2.0]UNDEC-4-ENE, 4,11,11-TRIMETHYL-8-METHYLENE-, [1R-(1R*,4E,9S*)]-
Formula C15H24, MW 204, CAS# 87-44-5, Entry# 89162
2,6,10,10-TETRAMETHYLBICYCLO[7.2.0]UNDECA-1,6-DIENE #

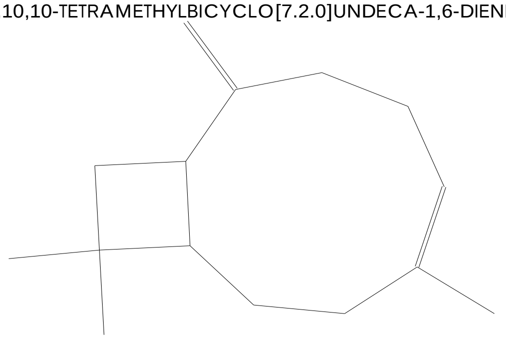

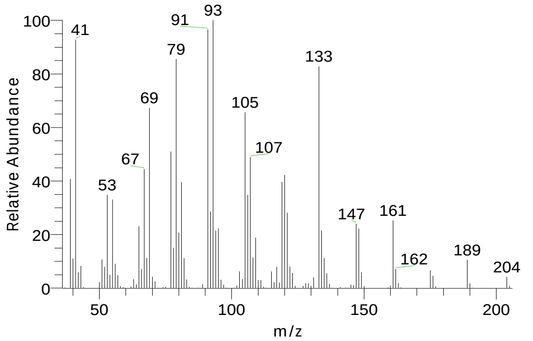

TRANS(á)-CARYOPHYLLENE
Formula C15H24, MW 204, CAS# NA, Entry# 89641

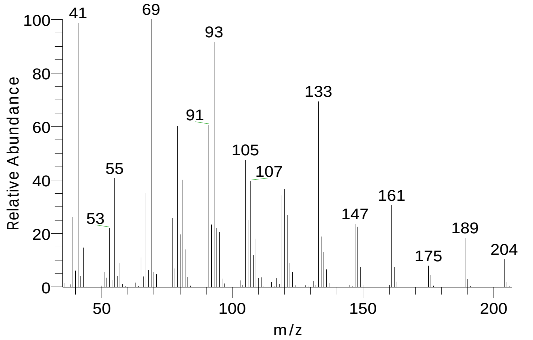

BICYCLO[7.2.0]UNDEC-4-ENE, 4,11,11-TRIMETHYL-8-METHYLENE-, [1R-(1R*,4E,9S*)]-
Formula C15H24, MW 204, CAS# 87-44-5, Entry# 89170
2,6,10,10-TETRAMETHYLBICYCLO[7.2.0]UNDECA-1,6-DIENE #

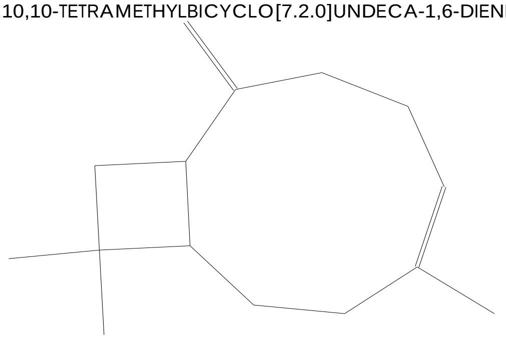

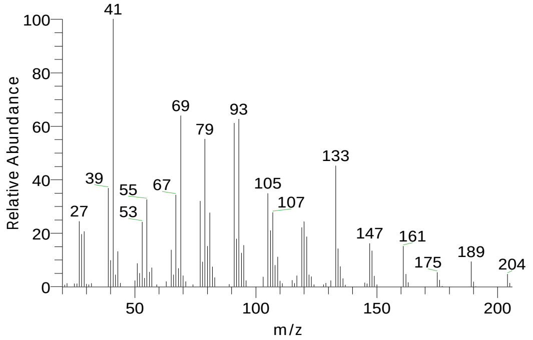

My GC-MS Report

Compound Structure | Hit Spectrum

BICYCLO[7.2.0]UNDEC-4-ENE, 4,11,11-TRIMETHYL-8-METHYLENE-, [1R-(1R*,4E,9S*)]-
Formula C15H24, MW 204, CAS# 87-44-5, Entry# 89160
2,6,10,10-TETRAMETHYLBICYCLO[7.2.0]UNDECA-1,6-DIENE #

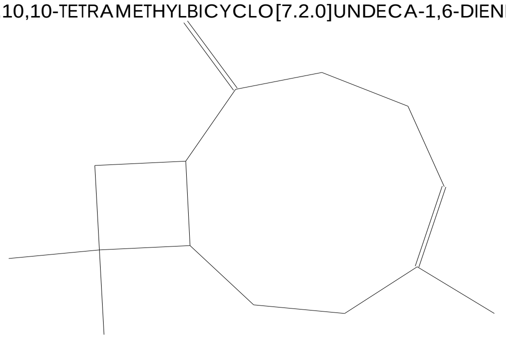

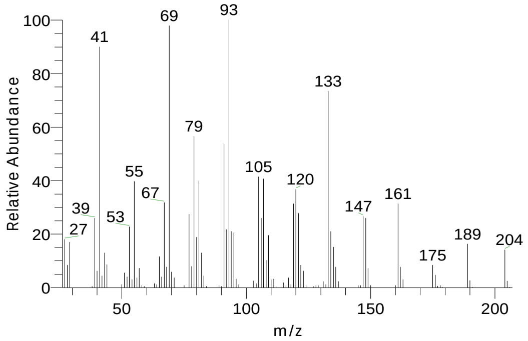

Rosemary_Oil #2184 RT: 11.32 AV: 1 NL: 7.18E7
T: + c EI Full ms [50.000-750.000]

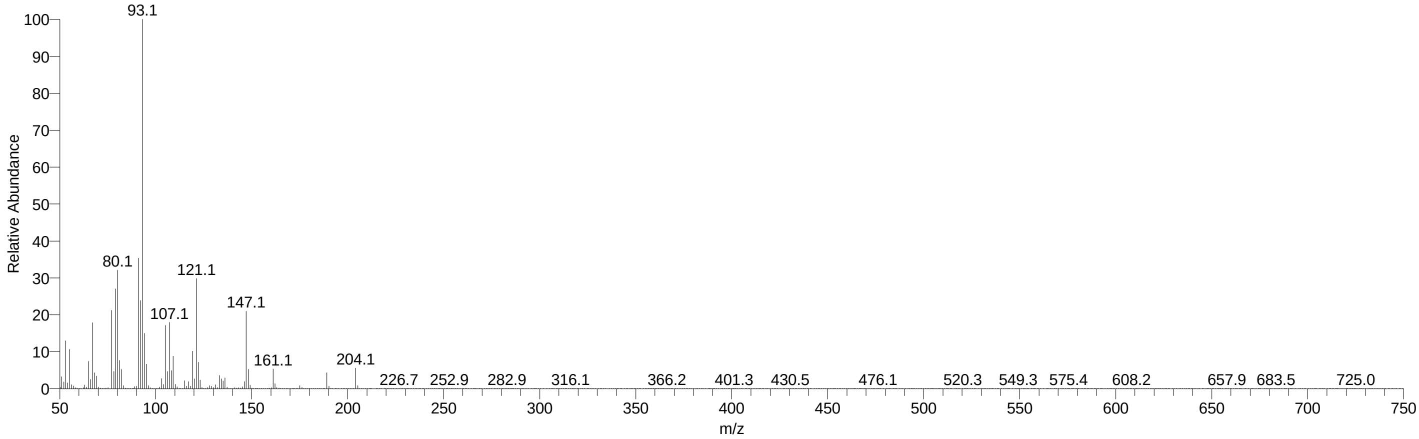

RT	Compound Name	Area %	MF	Molecular Formula	Molecular Weight	Cas #	Library
11.32	Humulene	3.20	947	C15H24	204	6753-98-6	replib
11.32	1,4,8-CYCLOUNDECATRIENE, 2,6,6,9-TETRAMETHYL-, (E,E,E)-	3.20	924	C15H24	204	6753-98-6	WileyRegistry8e
11.32	1,4,8-CYCLOUNDECATRIENE, 2,6,6,9-TETRAMETHYL-, (E,E,E)-	3.20	916	C15H24	204	6753-98-6	WileyRegistry8e
11.32	Humulene	3.20	919	C15H24	204	6753-98-6	replib
11.32	1,4,8-CYCLOUNDECATRIENE, 2,6,6,9-TETRAMETHYL-, (E,E,E)-	3.20	908	C15H24	204	6753-98-6	WileyRegistry8e

Compound Structure | Hit Spectrum

Humulene
Formula C15H24, MW 204, CAS# 6753-98-6, Entry# 14003
à-Caryophyllene

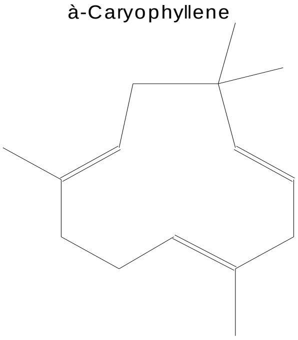

SI 940, RSI 947, replib, Entry# 14003, CAS# 6753-98-6, Humulene

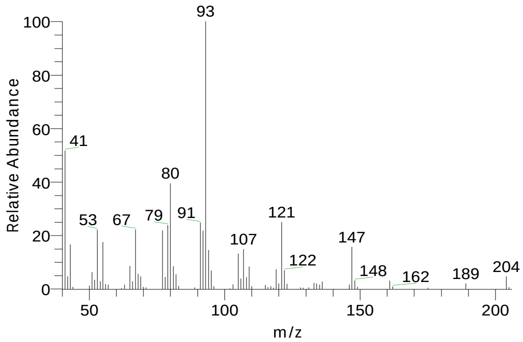

My GC-MS Report

Compound Structure | Hit Spectrum

1,4,8-CYCLOUNDECATRIENE, 2,6,6,9-TETRAMETHYL-, (E,E,E)-
Formula C15H24, MW 204, CAS# 6753-98-6, Entry# 89108
2,6,6,9-TETRAMETHYL-1,4,8-CYCLOUNDECATRIENE #

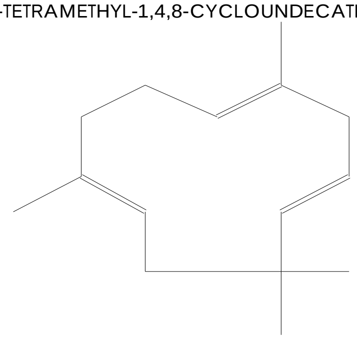

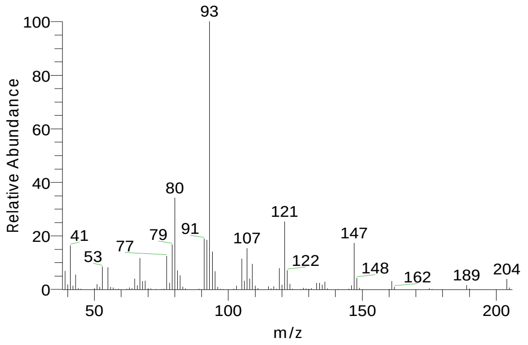

1,4,8-CYCLOUNDECATRIENE, 2,6,6,9-TETRAMETHYL-, (E,E,E)-
Formula C15H24, MW 204, CAS# 6753-98-6, Entry# 89110
2,6,6,9-TETRAMETHYL-1,4,8-CYCLOUNDECATRIENE #

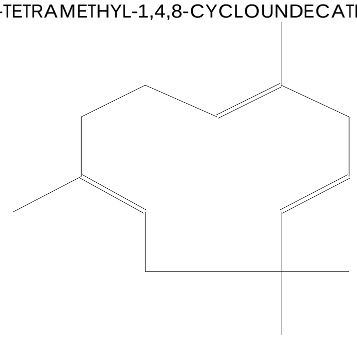

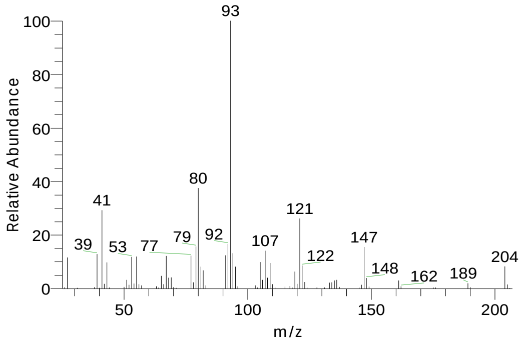

Humulene
Formula C15H24, MW 204, CAS# 6753-98-6, Entry# 14104
à-Caryophyllene

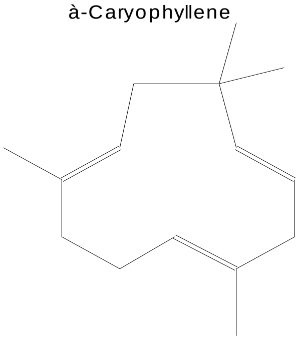

SI 909, RSI 919, replib, Entry# 14104, CAS# 6753-98-6, Humulene

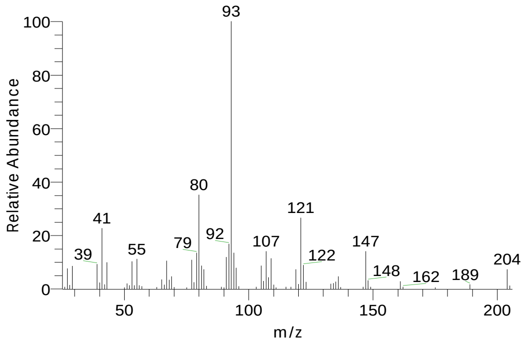

1,4,8-CYCLOUNDECATRIENE, 2,6,6,9-TETRAMETHYL-, (E,E,E)-
Formula C15H24, MW 204, CAS# 6753-98-6, Entry# 89113
2,6,6,9-TETRAMETHYL-1,4,8-CYCLOUNDECATRIENE #

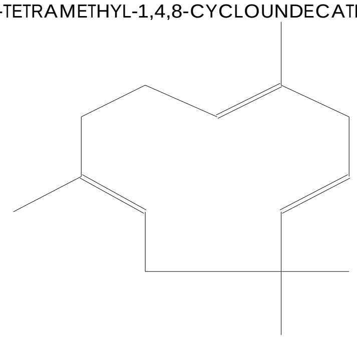

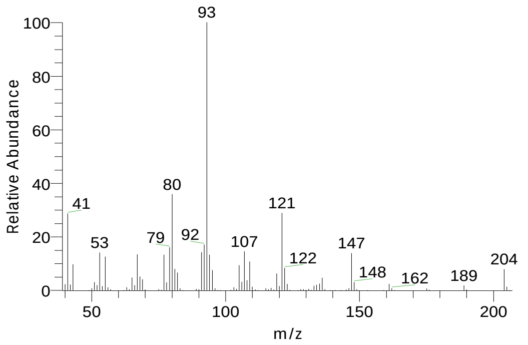

My GC-MS Report

Rosemary_Oil #2351 RT: 11.88 AV: 1 NL: 2.80E6
T: + c EI Full ms [50.000-750.000]

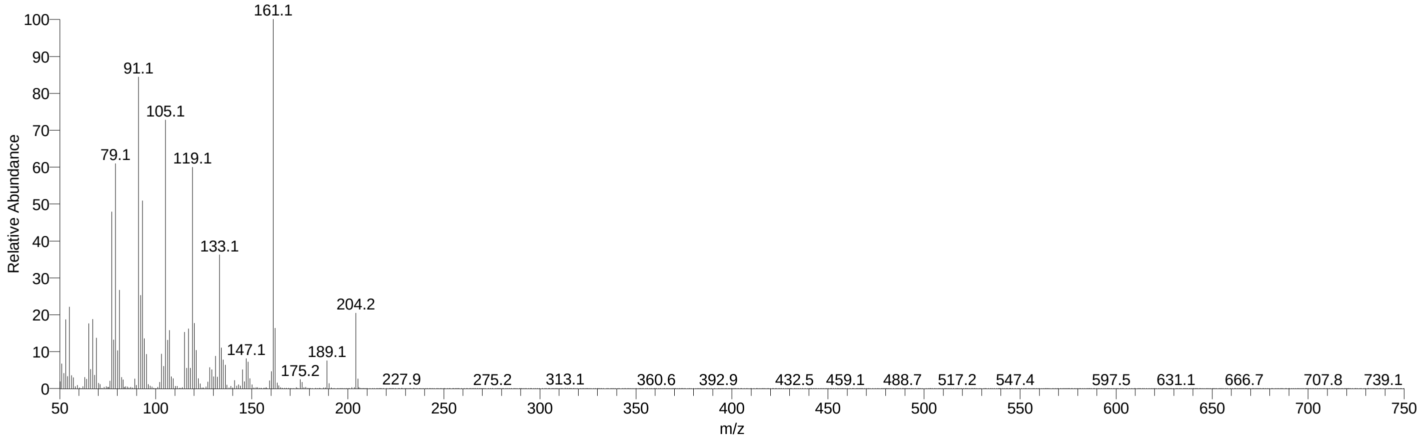

RT	Compound Name	Area %	MF	Molecular Formula	Molecular Weight	Cas #	Library
11.88	ç-Muurolene	0.26	945	C15H24	204	30021-74-0	replib
11.88	Naphthalene, 1,2,4a,5,6,8a-hexahydro-4,7-dimethyl-1-(1-methylethyl)-	0.26	949	C15H24	204	483-75-0	replib
11.88	ç-Muurolene	0.26	926	C15H24	204	30021-74-0	replib
11.88	Naphthalene, 1,2,3,4,4a,5,6,8a-octahydro-7-methyl-4-methylene-1-(1-methylethyl)-, (1à,4aá,8aà)-	0.26	913	C15H24	204	39029-41-9	replib
11.88	(1S,4aR,8aS)-1-Isopropyl-7-methyl-4-methylene-1,2,3,4,4a,5,6,8a-octahydronaphthalene	0.26	912	C15H24	204	6980-46-7	mainlib

Compound Structure	Hit Spectrum

ç-Muurolene
Formula C15H24, MW 204, CAS# 30021-74-0, Entry# 24843

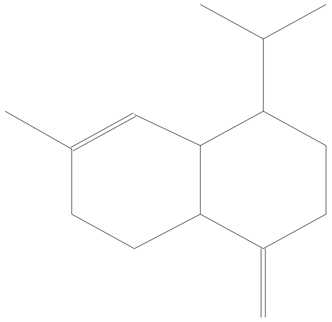

SI 932, RSI 945, replib, Entry# 24843, CAS# 30021-74-0, ç-Muurolene

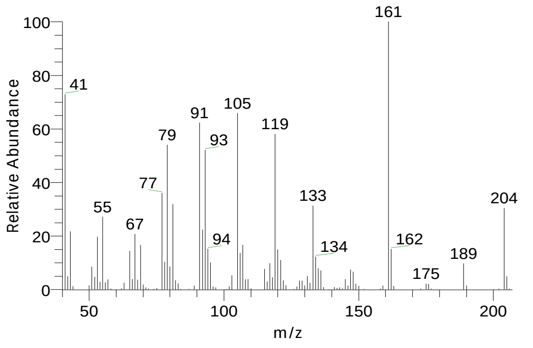

Naphthalene, 1,2,4a,5,6,8a-hexahydro-4,7-dimethyl-1-(1-methylethyl)-
Formula C15H24, MW 204, CAS# 483-75-0, Entry# 24889
1-Isopropyl-4,7-dimethyl-1,2,4a,5,6,8a-hexahydronaphthalene #

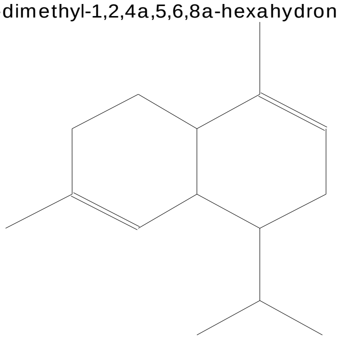

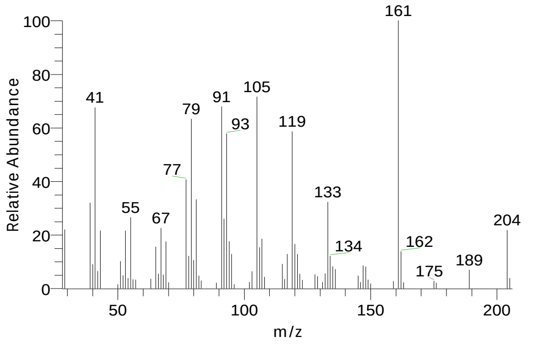

My GC-MS Report

Compound Structure	Hit Spectrum

ç-Muurolene
Formula C15H24, MW 204, CAS# 30021-74-0, Entry# 24906

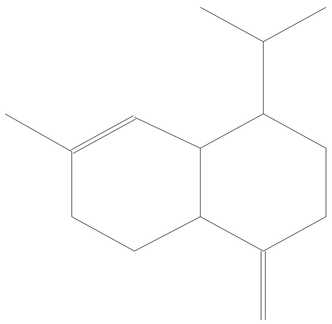

SI 917, RSI 926, replib, Entry# 24906, CAS# 30021-74-0, ç-Muurolene

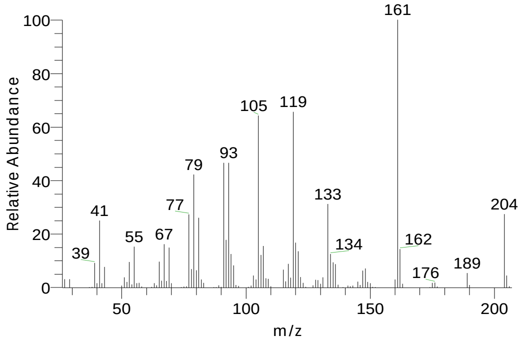

Formula C15H24, MW 204, CAS# 39029-41-9, Entry# 24895
ç-Cadinene

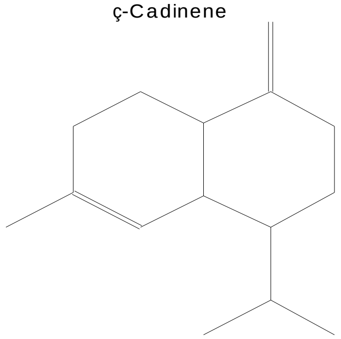

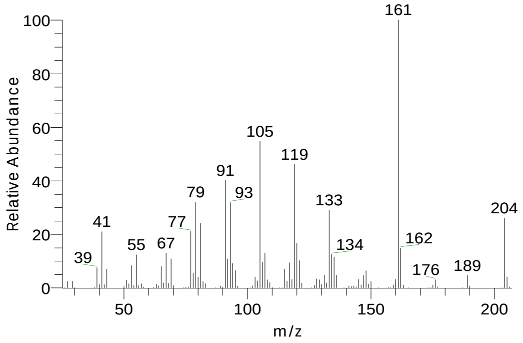

(1S,4aR,8aS)-1-Isopropyl-7-methyl-4-methylene-1,2,3,4,4a,5,6,8a-octahydronaphthalene
Formula C15H24, MW 204, CAS# 6980-46-7, Entry# 150501
ç-Amorphene

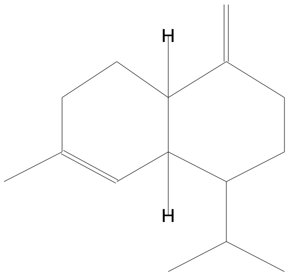

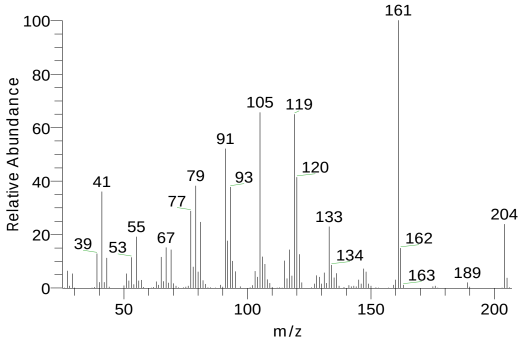

Rosemary_Oil #2680 RT: 12.98 AV: 1 NL: 3.94E6
T: + c EI Full ms [50.000-750.000]

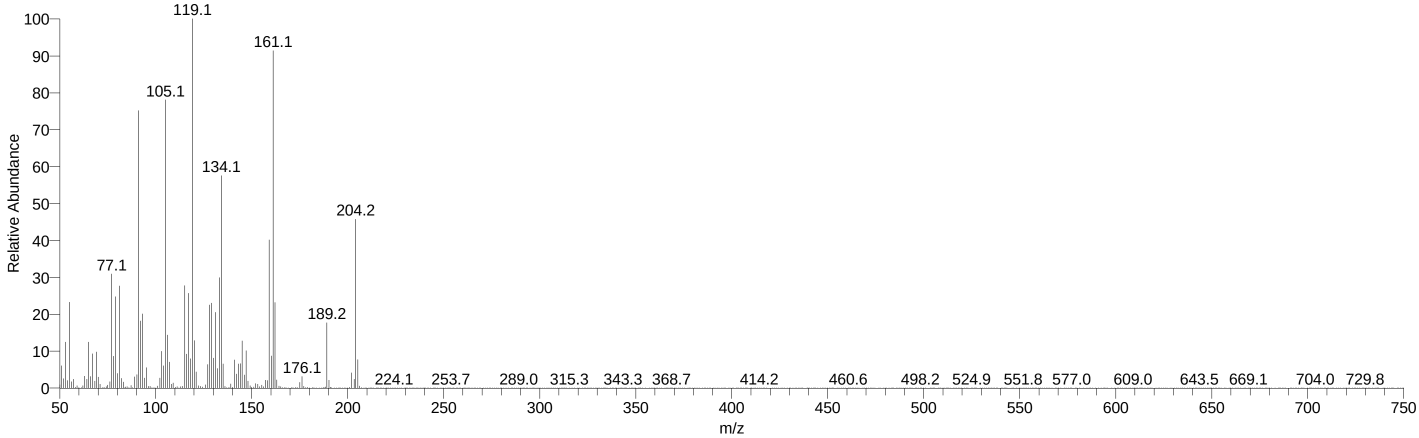

RT	Compound Name	Area %	MF	Molecular Formula	Molecular Weight	Cas #	Library
12.98	NAPHTHALENE, 1,2,3,5,6,8A-HEXAHYDRO-4,7-DI METHYL-1-(1-METHYLETHYL)-, (1S-CIS)-	0.39	928	C15H24	204	483-76-1	WileyRegistry8e

My GC-MS Report

RT	Compound Name	Area %	MF	Molecular Formula	Molecular Weight	Cas #	Library
12.98	Naphthalene, 1,2,3,5,6,8a-hexahydro-4,7-dimethyl-1-(1-methylethyl)-, (1S-cis)-	0.39	911	C15H24	204	483-76-1	replib
12.98	1-Isopropyl-4,7-dimethyl-1,2,3,5,6,8a-hexahydronaphthalene	0.39	913	C15H24	204	16729-01-4	mainlib
12.98	NAPHTHALENE, 1,2,3,5,6,8A-HEXAHYDRO-4,7-DIMETHYL-1-(1-METHYLETHYL)-, (1S-CIS)-	0.39	873	C15H24	204	483-76-1	WileyRegistry8e
12.98	NAPHTHALENE, 1,2,3,5,6,8A-HEXAHYDRO-4,7-DIMETHYL-1-(1-METHYLETHYL)-, (1S-CIS)-	0.39	863	C15H24	204	483-76-1	WileyRegistry8e

Compound Structure	Hit Spectrum

NAPHTHALENE, 1,2,3,5,6,8A-HEXAHYDRO-4,7-DIMETHYL-1-(1-METHYLETHYL)-, (1S-CIS)-
Formula C15H24, MW 204, CAS# 483-76-1, Entry# 89277
1-ISOPROPYL-4,7-DIMETHYL-1,2,3,5,6,8A-HEXAHYDRONAPHTHALENE #

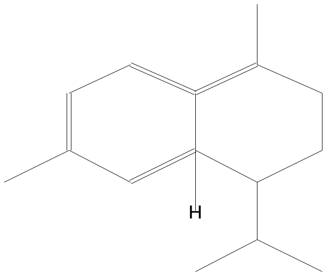

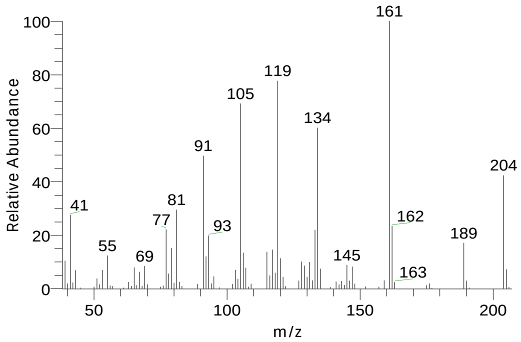

Naphthalene, 1,2,3,5,6,8a-hexahydro-4,7-dimethyl-1-(1-methylethyl)-, (1S-cis)-
Formula C15H24, MW 204, CAS# 483-76-1, Entry# 24907
Cadina-1(10),4-diene

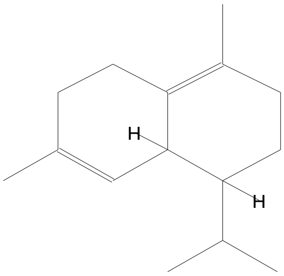

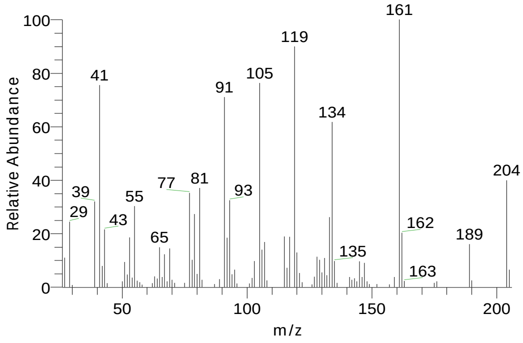

1-Isopropyl-4,7-dimethyl-1,2,3,5,6,8a-hexahydronaphthalene
Formula C15H24, MW 204, CAS# 16729-01-4, Entry# 150583
Cadina-1(10),4-diene

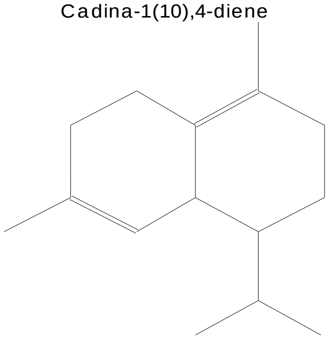

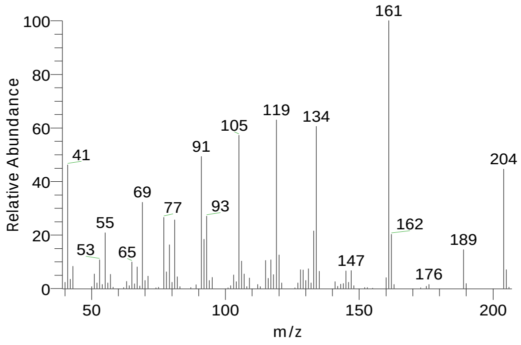

My GC-MS Report

Compound Structure | Hit Spectrum

NAPHTHALENE, 1,2,3,5,6,8A-HEXAHYDRO-4,7-DIMETHYL-1-(1-METHYLETHYL)-, (1S-CIS)-
Formula $C_{15}H_{24}$, MW 204, CAS# 483-76-1, Entry# 89273
1-ISOPROPYL-4,7-DIMETHYL-1,2,3,5,6,8A-HEXAHYDRONAPHTHALENE #

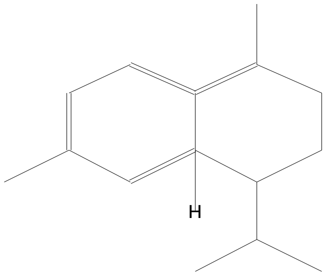

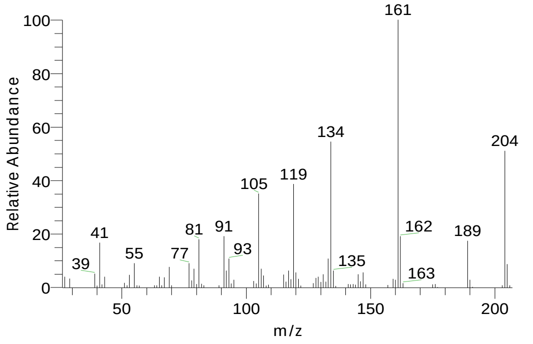

NAPHTHALENE, 1,2,3,5,6,8A-HEXAHYDRO-4,7-DIMETHYL-1-(1-METHYLETHYL)-, (1S-CIS)-
Formula $C_{15}H_{24}$, MW 204, CAS# 483-76-1, Entry# 89275
1-ISOPROPYL-4,7-DIMETHYL-1,2,3,5,6,8A-HEXAHYDRONAPHTHALENE #

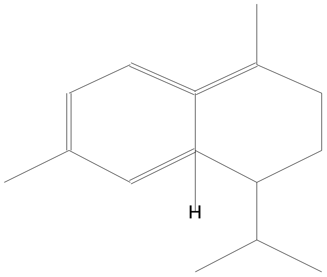

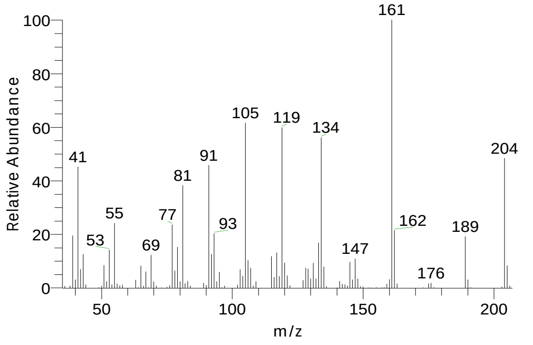

Supplement: Supplementary data 3 [file mmc3.pdf]
