# Supplementary material for: Larvicidal and adulticidal activity of essential oils from plants of the Lamiaceae family against the West Nile virus vector, Culex pipiens (Diptera: Culicidae)
Source: Saudi J Biol Sci. 2022 Jun 16;29(8):103350. doi: 10.1016/j.sjbs.2022.103350 (PMC9232543; doi:10.1016/j.sjbs.2022.103350)
Supplement: Supplementary data 4 [file mmc4.pdf]

Larvicidal and Adulticidal Activity of Essential Oils from Plants of the Lamiaceae Family against the West Nile Virus Vector, Culex pipiens (Diptera: Culicidae)

By Hanan Bosly

WORD COUNT 5139

TIME SUBMITTED 30-APR-2022 03:35PM

PAPER ID 85419116

Larvicidal and Adulticidal Activity of Essential Oils from Plants of the Lamiaceae Family against the West Nile Virus Vector, Culex pipiens (Diptera: Culicidae)

Abstract

Culex pipiens mosquitoes are the most widely distributed primary vector of the West Nile virus worldwide. Many attempts for investigation of botanical pesticides to avoid the development of pesticide resistance to conventional synthetic pesticides that are recognized as a threat to the diversity of ecosystems. The study aimed to determine the components of three essential oils of Lamiaceae family, lavender (*Lavandula angustifolia*), peppermint (*Mentha piperita* L.), and rosemary (*Rosmarinus officinalis* L.) by gas chromatography-mass spectrometry (GC-MS) analysis. Furthermore, aimed to validate the insecticidal activities of these oils as larvicidal agents against the third instar larvae of *Culex pipiens* using five different concentrations (62.5, 125, 250, 500, and 1000 ppm) for each oil in five replicates and as an adulticidal agent against approximately three day-old female adults of *Cx. Pipiens* using 0.5, 1, 2, 4, and 5% concentrations in three replicates. The results generally showed a dose-related response. At 1000 ppm, rosemary oil showed the highest larvicidal (100%) (LC_{50}, 214.97 ppm), followed by peppermint oil (92.00% mortality and LC_{50} (269.35 ppm). Lavender oil showed the lowest efficacy with 87.20% mortality and LC_{50} (301.11 ppm). At 5% oil concentration, the highest knockdown rate at 1 h was recorded for lavender oil (95.55%), followed by peppermint oil (88.89%) and lastly rosemary oil (84.44%). After 24 h, rosemary oil showed the lowest adult mortality rate (88.89%; LC_{50}, 1.44%), while lavender and peppermint oils both showed a 100% mortality rate, with (LC_{50}, 0.81% and 0.91%, respectively). The chemical constituents of the oils consisted of monoterpenes and sesquiterpenes that determined their insecticidal activities against the target insect stage. The study proposed that rosemary essential oil may be useful for the control of *Cx. pipiens* larvae as part of an integrated water treatment strategy, and lavender and peppermint oils may be used in an integrated plan for adult's control.

Keywords: Lavender, Peppermint, Rosemary, Essential Oils, *Culex pipiens,* Insecticidal.

1. Introduction

The Lamiaceae family called the mint family is characterized by economically important species of aromatic plants including 250 genera and 7825 species (Prakash et al., 2016). Their essential oils are obtained from different aerial parts (seeds, leaves, flowers, shoots, and fruits) or roots. These oils contain characteristic functional groups such as aldehydes, esters, phenols, ketones, alcohols, organic acids, hydrocarbons and terpenoids Essential oils may be preferred in the industrial economic sector due to the development of fewer toxic side products and economic viability (Nazar et al., 2022; Ramos da Silva et al., 2021).

The present study investigated three essential oils from three genera of the family Lamiaceae. The first was lavender oil from the flowering parts of *Lavandula angustifolia* L. This species showed anti-inflammatory, antibacterial, antiviral, and antifungal properties and anticancer activity as well as to provide mood disturbance relief (Cavanagh and Wilkinson, 2002; Denner, 2009; Rai et al., 2020). The second was peppermint oil from the aerial parts of *Mentha* x *piperita* L. which is a hybrid mint (*M. aquatica* × *M. spicata*). Peppermint oil is used in the health industry because of its wide spectrum of therapeutic properties. These include analgesic, antispasmodic, and antiemetic action, abdominal pain relief, antioxidant activity, and cytotoxicity against bacteria and fungi as well as insecticidal effects against many pests (da Silva Ramos et al., 2017; Kalemba and Synowiec, 2019; Mahendran and Rahman, 2020). The third was rosemary oil from the flowering tops of the plant *Rosmarinus officinalis* L. which has been shown to have anti-inflammatory properties with potential applications in inflammatory-related diseases. Furthermore, it exerts anti-depressive, antimicrobial, antioxidant, antiallergic, and smooth muscle relaxant effects, as well as shows antifungal, antimutagenic, and insecticidal activities (Borges et al., 2019; De Oliveira et al., 2019; Isman et al., 2008; Krzyżowski et al., 2020).

Culex pipiens is commonly known house mosquito and one of the most widely distributed mosquitoes worldwide and several studies reported the involvement of *Cx. pipiens* in transmission of the West Nile virus (Al-Mekhlafi et al., 2018; El-Akhal et al., 2015). A previous study suggested that control efforts focused on *Cx. pipiens* alone may greatly reduce both human exposure, infection, and epidemic distribution (Hamer et al., 2008). The research interest for botanical pesticides containing natural compounds as active ingredients, from which essential oils are considered a part. Where the use of these compounds attempts to avoid the development of pesticide resistance to conventional synthetic pesticides that are also now recognized as a threat to the diversity of ecosystems (Ahmed et al., 2021).

The present study aimed to evaluate the adulticidal and larvicidal potential of lavender, peppermint, and rosemary oils against the common house mosquito, *Cx. pipiens*. Furthermore, we aimed to determine the oils compounds by gas chromatography-mass spectrometry (GC-MS) analysis.

2. Material and Methods

2.1. Mosquito colony rearing and experimental conditions

Mosquitoes (*Cx. pipiens*) were reared for several generations at Center for Environmental Research and Studies at Jazan University. Rearing was performed under controlled conditions (27 ± 2 °C, relative humidity at 70% ± 10%, and 12:12 h light:dark regime). Adult mosquitoes were reared in wooden cages and supplied daily by 10% sucrose solution soaked in sponge pieces for 3–4 days post emergence. Then, females were supplied pigeon blood meal. Plastic oviposition cups contained tap water (without chlorine) were placed in the cages. The resulting egg mass were transferred into plastic pans containing 3 L of tap water for 24 h. The hatching larvae were provided with a diet of fish food daily. The third instar *Cx. pipiens* larvae were used for the larvicidal examination, and the adults used for the adulticidal examination were glucose-fed female mosquitoes reared under the aforementioned controlled conditions.

2.2. Essential oils

Lavender essential oil, steam-distilled from flowering tops of *L. angustifolia*, (Lamiales: Lamiaceae; Case No. 8000-28-0), Peppermint essential oil, steam-distilled from the aerial parts of *M. piperita* L. (Lamiales: Lamiaceae; Case No. 8006-90-4), and Rosemary essential oil, steam-distilled from the tops of *R. officinalis* L. (Lamiales: Lamiaceae; Case No. 8000-25-7) were purchased from NOW Foods company (Natural Organic and Wholesome Foods), a distributor in Jeddah, Kingdom of Saudi Arabia.

2.3. Identification of the chemical composition using gas chromatography-mass spectrometer

The investigation of the chemical composition of the essential oils was performed by GC-MS using the Trace GC-TSQ mass spectrometer (Thermo Fisher Scientific, Waltham, MA, USA) with a direct capillary column, TG–5MS (30 m × 0.25 mm × 0.25 m thickness of the film). The operating conditions of the GC were column oven temperature initially maintained at 50 °C, then elevated at a rate of 5 °C/min up to 250 °C, maintained for 2 min, then elevated at a rate of 30 °C/min to 300 °C. The MS transfer line and the injector were adjusted at 270 °C and 260 °C, respectively,

and helium was the carrier gas at rate, 1 mL/min. The solvent retention time was 4 min, and 1 μL of the diluted samples was automatically injected using an AS1300 autosampler and GC split mode. In full scanning mode, electrospray ionization (EI) mass spectra were in range 50–650 m/s at an ionization voltage of 70 V. The temperature of the ion source was fixed at 200 °C. The mass spectra of the components then compared with those in the mass spectral libraries from the NIST 14 and WILEY 09 databases, the selected constituents were identified from the Total Ion Chromatogram (TIC).

2.3. Larvicidal assay

The larvicidal activity was determined for the lavender, peppermint, and rosemary oils against *Cx. pipiens* third instar larvae according to guidelines of the World Health Organization (WHO, 2005). The solution of each oil was set as stock by mixing 1 mL oil with distilled water containing 0.2 mL Tween-20. Subsequently, five different concentrations were prepared, (62.5, 125, 250, 500, and 1000 ppm) based on v/v percent of 1% of stock solution. Twenty-five *Cx. pipiens* larvae were offered to each oil in 250 mL glass beakers containing 150 mL water at the aforementioned controlled conditions. Five replicates per concentration per oil and control were conducted. The larval mortalities were detected after 24 h for estimation of larval lethal concentration (LC_{50}) by probit analysis.

2.4. Adulticidal assay

The adulticidal activity for the oils was analyzed by the adapted CDC bottle protocol (Ilahi et al., 2021; WHO, 2016). Five different concentrations from each oil dissolved in ethanol were prepared (0.5, 1, 2, 4, and 5%). Each prepared concentration for every oil was used to coat the CDC bottles (250 mL Wheaton bottles with screw lids) similarly to the control bottle, which was coated with only ethanol. The solvent was evaporated from the bottles for 1 h at 27 ± 2 ºC. Three replicates per concentration per oil and the control were conducted. Adult glucose-fed female mosquitoes (n = 15) aged 3–4 d were selected using an aspirator and gently introduced to each bottle, and the bottles were closed with their lids. If a mosquito was knocked down or unable to move or stand within 60 min of exposure, it was considered as dead. The number was recorded for each bottle after 5, 10, 20, 30 and 60 min for determination of the median knockdown time (KT_{50}) value and KT_{90} and KT_{95} values of each concentration through probit analysis. Live mosquitoes were then removed from bottles after 1 h and placed in separate paper cups containing 10% sucrose solution.

Subsequently, the adult mortality rate was measured after 24 h within the replicates for determination of the sLC_{50}, LC_{90} and LC_{95} by probit analysis.

2.5. Data analysis

The percentage mortalities were calculated according to Abbott's formula (Abbott, 1925). The larval control results did not need correction, as the mortality was less than 5%, according to the WHO guidelines (WHO, 2005) (no larval control mortality recorded throughout the study). The adult data were also not corrected, as the control adults had a mortality of less than 20% (WHO, 2016). Data from all the replicates were statistically analyzed to determine the larval and adult's LC_{50}, LC_{50}, and LC_{95} values and the adult KT_{50}, KT_{90}, and KT_{95} values as well as chi-square values within confidence limits at 95% (lower confidence limit [LCL] and upper confidence limit [UCL]) by probit analysis using regression between log oil concentration and probit values. Mortality data were subjected to one-way analysis of variance (ANOVA) (with a least significant difference (LSD) test). Data analysis was performed using SPSS software (IBM SPSS Statistics v22 – 64 bit), and $p < 0.05$ was considered significant.

3. Results

3.1. Chemical analysis

The GC-MS analysis represented that the main compounds of the three essential oils were the monoterpenoids and sesquiterpenes (Table 4; Figure 4). In lavender (*L. angustifolia*) essential oil, the main represented area% were for monoterpenoids, linalool (23.75%), linalyl anthranilate (21.92%), lavandulyl acetate (11.85%), 4-terpineol (8.90%), and linalyl acetate (6.66%), followed by geranyl acetate (2.99%), L-α-terpineol (0.70%), and bornyl acetate (0.10%). The sesquiterpenoids were β-caryophyllene (16.35%), (E)-β-famesene (14.09%), germacrene D (1.29%), caryophyllene oxide (0.77%), γ-muurolene (0.35%), and transe-α-bergamotene (0.26%). In peppermint (*M. piperita* L.) oil, the main represented monoterpenoids were menthyl acetate (32.76%), L-menthol (26.41%), and L-menthone (13.57%), followed by pulegone (1.94%), L-alpha-terpineol (1.63%), and piperitone (1.32%). The main sesquiterpenoid was β-caryophyllene (18.48%), followed by humulene (1.41%), germacrene D (0.88%), β-bourbonene (0.58%), elemene isomer (0.46%), and alloaromadendrene (0.34%). In Rosemary (*R. officinalis* L.) essential oil, the chemical compounds were represented by camphor (56.55%) as the major monoterpenoid, followed by isoborneol (7.16%), α-terpineol (5.40%), and bornyl acetate (3.69%), and the main

sesquiterpenoid was β-caryophyllene (23.0%), followed by humulene (3.20%), cadina-1(10),4-diene (0.39%), α-copaene (0.34%), and γ-muurolene (0.26%).

3.2. Larvicidal activity

The data on the larvicidal activity of the tested essential oils against the third instar larvae of *Cx. pipiens* are represented in Table 1. The probit regression responses of the essential oils tested revealed that the larvicidal activities after 24 h ranged from 87.20 to 100% mortality at 1000 ppm. Rosemary essential oil showed the highest efficacy by inducing 100% mortality (LC_{50}, 214.97, LC_{90}, 671.77, and LC_{95}, 927.90 ppm), followed by peppermint oil by 92.00% mortality (LC_{50}, 269.35, LC_{90}, 1137.74, and LC_{95}, 1711.70 ppm). Lavender oil was the least effective, inducing 87.20% mortality (LC_{50}, 301.11, LC_{90}, 1437.63, and LC_{95}, 2239.31 ppm) (Figure 1).

3.3 Adulticidal activity

The effect of the test concentrations of the three essentials oils on *Cx. pipiens* females were evaluated after 60 min of exposure in terms of LC_{50}, which is shown in Figure 2, KT_{50}, KT_{90}, and KT_{95}, which are listed in Table 2. According to the analysis, the highest knockdown rate was recorded for lavender oil (95.55%) followed by that for peppermint oil (88.89%) and lastly rosemary oil (84.44%) at the highest tested concentration of the oils (5%).

When considering knockdown times, data revealed that at a concentration of 5%, the knockdown times were the lowest for lavender oil compared with those recorded for the other two oils. Lavender oil had a KT_{50} of 17.43 min, KT_{90} of 57.51 min, and KT_{95} of 80.68 min. Peppermint oil showed a KT_{50} of 22.81 min, KT_{90} of 95.54 min, and KT_{95} of 143.40 min. Furthermore, rosemary oil revealed a KT_{50} of 26.64 min, KT_{90} of 102.56 min, and KT_{95} of 150.29 min.

Adult toxicity of the three tested oils after 24 h was also assessed (Table 3), and probit regression results are shown in Figure 3. Data showed contrary results to those observed for the larvicidal activity of the tested oils. Here, rosemary essential oil showed the least adulticidal efficiency (88.89% mortality rate) and an LC_{50} of 1.44% after 24 h at the highest tested concentration (5%). Lavender and peppermint oils at 5% concentration both showed a mortality rate of 100%, with an LC_{50} of 0.81% and 0.91%, respectively, after 24 h. It was noted that the lowest median adulticidal concentration was that of lavender oil which was in line with knockdown assay results.

4. Discussion

The present study tested the larvicidal and adulticidal efficacy of lavender, peppermint, and rosemary essential oils against *Cx. pipiens* mosquitos. These effects were expected owing to the active constituents of these oils. The major components present in lavender essential oil as determined by GC-MS in this study corresponded with the findings of previous studies (El-Akhal et al., 2021; Karamaouna et al., 2013), as did the components present in the peppermint essential oil (Ebadollahi et al., 2017; Mackled et al., 2019; Samarasekera et al., 2008) and the rosemary essential oil (Jafari-sales and Pashazadeh, 2020; Zeghib et al., 2020). Generally, the tested three essential oils recorded chemical constituents and their insecticidal activities in accordance with the methods of several previous studies reviewed by Ebadollahi et al (2020).

The study was coverage the larvicidal activity of the tested oils against the third instar larvae of *Cx. Pipiens*. The study evaluated the concentration-related mortalities for each of the oils, and graded the mortalities at the highest used concentration (1000 ppm), where rosemary oil acquired the most potent larvicidal activity (100% mortality) followed by peppermint oil (92%) and lastly lavender oil (87%).

In line with the present results about larvicidal LC_{50} value, Radwan et al. (2008), recorded an LC_{50} of 216.10 ppm for rosemary essential oil and a lower larvicidal efficacy of peppermint oil (>500 ppm) against *Cx. pipiens*. The study presented that the saturated monoterpenoid alcohol menthol, which is the major component in peppermint oil, exhibited lower larvicidal activity against *Cx. pipiens*. Zeghib et al. (2020) evaluated larvicidal activity against the fourth instar larvae of *Cx. pipiens* of rosemary essential oil extracted from the aerial parts of *R. officinalis*. They recorded 100% mortality at 99.34 ppm of the oil concentration and evaluated camphor as one of the major components. A further component that had a role in toxicity was β-caryophyllene, which was the second most abundant component detected in rosemary oil (23%) and the third most abundant in lavender (16.35%) and peppermint oils (18.48%) and displayed effective larvicidal activity as an individual compound against *Anopheles subpictus, Aedes albopictus*, and *Cx. tritaeniorhynchus* (Govindarajan et al., 2016). A sesquiterpene compound, α-humulene, was present in a higher percentage in rosemary oil (3.20%) than in peppermint oil (1.41%) and not detected in lavender oil. This compound exhibited larvicidal activity at low dosages against *A. subpictus, Ae. albopictus,* and *Cx. tritaeniorhynchus* third instar larvae (Govindarajan and Benelli, 2016). Linalool, the main constituent in lavender oil, had larvicidal activity against third instar larvae of *Ae. aegypti*, and morphological alterations detected in larvae exposed to sublethal doses of linalool

included abdomen elongation and curving and gut content partial extrusion involving the perithrophic matrix (Fujiwara et al., 2017). Essential oil extracted from *M. piperita* leaves exhibited larvicidal and repellent efficacy against the early fourth instar larvae of *Ae. aegypti* (Kumar et al., 2011). Another study showed toxicity effects of peppermint and lavender essential oils against *M. domestica* larvae in conjunction with larval morphological abnormalities (Bosly, 2013). Moreover, studies reported that minor compounds found such as bornyl acetate (present in rosemary oil) exhibited strong larvicidal activity against *Cx. pipiens* larvae (Zeghib et al., 2020). Other compounds present in high concentrations, similarly to our study, such as linalool and terpineol (in lavender oil) and menthone (in peppermint oil) individually exhibited low larvicidal activities with recorded LD_{50} of 193,194, and 156 mg/L, respectively, against *Cx. pipiens* larvae (Radwan et al., 2008; Traboulsi et al., 2002).

Contrary to the larvicidal results obtained in this study, lavender, peppermint, and rosemary essential oils at 1000 ppm concentration resulted in larval mortality of 68, 100 and 80%, respectively, after 24 h against *Cx. quinquefasciatus*, revealing the potent larvicidal effect of peppermint oil (Manimaran et al., 2012). El-Akhal et al (2021) showed that lavender oil at 800 ppm resulted in 100% larval mortality with an LC_{50} of 140 ppm against *Cx. pipiens* larvae. Zhu and Tian (2013) tested the larvicidal activities of camphor, eucalyptol, terpine-4-ol, germacrene D, caryophyllene oxide, and caryophyllene against *A. anthropophagus* and found that the most potent were caryophyllene oxide and germacrene D, followed by terpine-4-ol and camphor, and the least potent was caryophyllene. In addition, Dias and Moraes (2014) reported that majority of essential oils showed potent larvicidal activity derived from species of particular families, such as Laminaceae that are rich in sesquiterpenes and monoterpene hydrocarbons, and their individual pure compounds showed against mosquito larvae high activity.

Herein, the tested plant oils are not toxic to vertebrates and identified to be eco-friendly as well as used as medicinal plants. However their varied related toxicity potential with insect stage. Rosemary oil displayed the strongest larvicidal activity, while lavender and peppermint oils exhibited the strongest adulticidal activity. The adulticidal and knockdown activities of the tested oils recorded 100% mortalities for lavender and peppermint oils with KT_{50} of 17.43 and 22.81 min, respectively, while rosemary oil showed only 88.89% mortality with a KT_{50} of 26.64 min.

Structural variations of peppermint constituents, menthol, menthyl acetate, menthone, α-terpineol, pulegone, and β-caryophyllene, which were previously shown to be adulticidal and have

knockdown efficacy against 3-day-old females of *Cx. Quinquefasciatus*, could contribute towards retaining or enhancing the oil mosquitocidal activity (Samarasekera et al., 2008). Pulegone was the most effective adulticidal among different tested terpenes with LC_{50} values lower than 0.1 mg/L against *Ae. aegypti* adults (Flores Guillermo et al., 2020). Linalool had an LC_{50} value of 14.87 µg/mL against adult *Cx. pipiens* mosquitoes (Tabari et al., 2017). Additionally, the repellent properties of peppermint essential oil were established against adults *Ae. aegypti* (Kumar et al., 2011; Manh and Tuyet, 2020). Effective repellent activity was also evaluated for lavender and rosemary essential oils against adult mosquitoes of *Cx. pipiens pallens* (Choi et al., 2002).

The toxicity for rosemary essential oil towards adults of *Cx. pipiens* was previously evaluated and proposed be attributed to its major monoterpenes constituents and recorded strong fumigant toxicity against adults, meanwhile, showed weak toxicity against larvae (Zahran et al., 2017). The study confirmed the oil constituent's role in toxicity, where interaction between the oil constituents is very important for the development of its insecticidal formulation and adjustment of the content active substances for formulation to ensure the biological efficacy as previously confirmed (Pavela, 2015; Sharma et al., 2022).

5. Conclusion

The present study reviled that rosemary oil acquired the most potent larvicidal activity (100% mortality) followed by peppermint oil (92%) and lastly lavender oil (87%). The adulticidal and knockdown activities of the tested oils recorded 100% mortalities for lavender and peppermint oils with KT_{50} of 17.43 and 22.81 min, respectively, while rosemary oil showed only 88.89% mortality with a KT_{50} of 26.64 min. The present study suggests that the synergistic effect of compounds present in each essential oil may elevate its biological activity against the target insect stage and proposed rosemary essential oil may be useful for control of *Cx. pipiens* larvae as a water treatment product and lavender and peppermint for application in control of adult mosquitoes.

Acknowledgements: The author wish to appreciate the Center for Environmental Research and Studies at Jazan University for the technical support.

Conflict of interest: The author declare that she has no competing interests

Table ligands and Figures captions

Table 1. The larvicidal effects of essential oils against the third instar larvae of *Culex pipiens* at 24 h post-treatment.

Table 2. Probit analysis of knockdown time and mortality rates of *Culex pipiens* females after oil exposure for 60 min.

Table 3. The adulticidal effects of essential oils against female adults of *Culex pipiens* at 24 h post-treatment.

Table 4. Chemical constituents of lavender, peppermint and rosemary essential oils.

Figure 1. Probit regression responses of lavender, peppermint and rosemary essential oils against *Culex pipiens* larval mortality.

Figure 2. Knockdown rate (mortality %) of lavender, peppermint and rosemary essential oils against *Culex pipiens* female adults. Significant differences at 0.05 level between different superscript letters to means of the same oil.

Figure 3. Probit regression responses of lavender, peppermint and rosemary essential oils against *Culex pipiens* adult mortality.

Figure 4. The TIC chromatograms of lavender, peppermint and rosemary essential oils chemical constituents detected by GC–MS.

References

Abbott, W.S., 1925. A method of computing the effectiveness of an insecticide. J. econ. Entomol 18(2), 265–267. https://doi.org/10.1093/jee/18.2.265a

Ahmed, N., Alam, M., Saeed, M., Ullah, H., Iqbal, T., Al-Mutairi, K.A., Shahjeer, K., Ullah, R., Ahmed, S., Ahmed, N.A.A.H., Khater, H.F., Salman, M., 2021. Botanical Insecticides Are a Non-Toxic Alternative to Conventional Pesticides in the Control of Insects and Pests, in: Global Decline of Insects. IntechOpen. https://doi.org/10.5772/intechopen.100416

Al-Mekhlafi, F.A., Abutaha, N., Farooq, M., Al-Wadaan, M., 2018. Insecticidal effect of Solenostemma argel extracts against Culex pipiens. J. Am. Mosq. Control Assoc. 34(3), 217–223. https://doi.org/10.2987/17-6725.1

Borges, R.S., Ortiz, B.L.S., Pereira, A.C.M., Keita, H., Carvalho, J.C.T., 2019. Rosmarinus officinalis essential oil: A review of its phytochemistry, anti-inflammatory activity, and mechanisms of action involved. J. Ethnopharmacol. 229, 29–45. https://doi.org/10.1016/j.jep.2018.09.038

Bosly, A.H., 2013. Evaluation of insecticidal activities of Mentha piperita and Lavandula angustifolia essential oils against house fly, Musca domestica L.(Diptera: Muscidae). J. Entomol. Nematol. 5(4), 50–54. https://doi.org/10.5897/jen2013.0073

Cavanagh, H.M.A., Wilkinson, J.M., 2002. Biological activities of lavender essential oil. Phyther. Res. 16(4), 301–308. https://doi.org/10.1002/ptr.1103

Choi, W.-S., Park, B.-S., Ku, S.-K., Lee, S.-E., 2002. Repellent activities of essential oils and monoterpenes against Culex pipiens pallens. J. Am. Mosq. Control Assoc. 18(4), 348–351. . https://doi.org/10.1016/j.fitote.2006.05.028

da Silva Ramos, R., Rodrigues, A.B.L., Farias, A.L.F., Simões, R.C., Pinheiro, M.T., Ferreira, R.M. dos A., Costa Barbosa, L.M., Picanço Souto, R.N., Fernandes, J.B., Santos, L. da S., 2017. Chemical composition and in vitro antioxidant, cytotoxic, antimicrobial, and larvicidal activities of the essential oil of Mentha piperita L.(Lamiaceae). Sci. World J. 2017. https://doi.org/10.1155/2017/4927214

De Oliveira, J.R., Camargo, S.E.A., De Oliveira, L.D., 2019. Rosmarinus officinalis L.(rosemary) as therapeutic and prophylactic agent. J. Biomed. Sci. 26, 1–22. https://doi.org/10.1186/s12929-019-0499-8

Denner, S.S., 2009. Lavandula angustifolia miller: english lavender. Holist. Nurs. Pract. 23(1), 57–64. . https://doi.org/10.1097/01.HNP.0000343210.56710.fc

Dias, C.N., Moraes, D.F.C., 2014. Essential oils and their compounds as Aedes aegypti L.(Diptera: Culicidae) larvicides. Parasitol. Res. 113, 565–592. https://doi.org/10.1007/s00436-013-3687-6

Ebadollahi, A., Davari, M., Razmjou, J., Naseri, B., 2017. Separate and combined effects of Mentha piperata and Mentha pulegium essential oils and a pathogenic fungus Lecanicillium muscarium against Aphis gossypii (Hemiptera: Aphididae). J. Econ. Entomol. 110(3), 1025–1030. https://doi.org/10.1093/jee/tox065

Ebadollahi, A., Ziaee, M., Palla, F., 2020. Essential oils extracted from different species of the Lamiaceae plant family as prospective bioagents against several detrimental pests. Molecules 25(7), 1556. https://doi.org/10.3390/molecules25071556

El-Akhal, F., Guemmouh, R., Ez Zoubi, Y., El Ouali Lalami, A., 2015. Larvicidal activity of Nerium oleander against larvae West Nile vector mosquito Culex pipiens (Diptera: Culicidae). J. Parasitol. Res. 2015. https://doi.org/10.1155/2015/943060

El-Akhal, F., Ramzi, A., Farah, A., Ez Zoubi, Y., Benboubker, M., Taghzouti, K., El Ouali Lalami, A., 2021. Chemical Composition and Larvicidal Activity of Lavandula angustifolia Subsp. angustifolia and Lavandula dentata Spp. dentata Essential Oils against Culex pipiens Larvae, Vector of West Nile Virus. Psyche A J. Entomol. 2021, 8872139. https://doi.org/10.1155/2021/8872139

Fujiwara, G.M., Annies, V., de Oliveira, C.F., Lara, R.A., Gabriel, M.M., Betim, F.C.M., Nadal, J.M., Farago, P. V, Dias, J.F.G., Miguel, O.G., Miguel, M.D., Marques, F.A., Zanin, S.M.W., 2017. Evaluation of larvicidal activity and ecotoxicity of linalool, methyl cinnamate and methyl cinnamate/linalool in combination against Aedes aegypti. Ecotoxicol. Environ. Saf. 139, 238–244. https://doi.org/https://doi.org/10.1016/j.ecoenv.2017.01.046

Govindarajan, M., Benelli, G., 2016. α-Humulene and β-elemene from Syzygium zeylanicum (Myrtaceae) essential oil: highly effective and eco-friendly larvicides against Anopheles subpictus, Aedes albopictus, and Culex tritaeniorhynchus (Diptera: Culicidae). Parasitol. Res. 115, 2771–2778. https://doi.org/10.1007/s00436-016-5025-2

Govindarajan, M., Rajeswary, M., Hoti, S.L., Bhattacharyya, A., Benelli, G., 2016. Eugenol, α-pinene and β-caryophyllene from Plectranthus barbatus essential oil as eco-friendly larvicides against malaria, dengue and Japanese encephalitis mosquito vectors. Parasitol. Res. 115(2), 807–815. https://doi.org/10.1007/s00436-015-4809-0

Guillermo, A.M.F., Nahuel, F., María, T.D., Andrés, M.V., Sara, M.P., 2020. Adulticidal effect of seven terpenes and a binary combination against Aedes aegypti. J. Vector Borne Dis. 57, 356. https://doi.org/10.4103/0972-9062.313975

Hamer, G.L., Kitron, U.D., Brawn, J.D., Loss, S.R., Ruiz, M.O., Goldberg, T.L., Walker, E.D., 2008. Culex pipiens (Diptera: Culicidae): a bridge vector of West Nile virus to humans. J. Med. Entomol. 45(1), 125–128. https://doi.org/10.1603/0022-2585(2008)45[125:cpdcab]2.0.co;2

Ilahi, I., Yousafzai, A.M., Attaullah, M., Haq, T.U., Rahim, A., Khan, W., Khan, A.A., Ullah, S., Jan, T., Khan, M.M., 2021. Mosquitocidal activities of Chenopodium botrys whole plant n-hexane extract against Culex quinquefasciatus. Brazilian J. Biol. 83. https://doi.org/10.1590/1519-6984.240842

Isman, M.B., Wilson, J.A., Bradbury, R., 2008. Insecticidal Activities of Commercial Rosemary Oils (*Rosmarinus officinalis*) against larvae of *Pseudaletia unipuncta* and *Trichoplusia ni* in Relation to Their Chemical Compositions. Pharm. Biol. 46, 82–87. https://doi.org/10.1080/13880200701734661

Jafari-sales, A., pashazadeh, M., 2020. Study of chemical composition and antimicrobial properties of Rosemary (*Rosmarinus officinalis*) essential oil on Staphylococcus aureus and Escherichia coli in vitro. Int. J. Life Sci. Biotechnol. 3, 62–69. https://doi.org/10.38001/ijlsb.693371

Kalemba, D., Synowiec, A. 2019. Agrobiological Interactions of Essential Oils of Two Menthol Mints: *Mentha piperita* and *Mentha arvensis*. Molecules, 25(1), 59. https://doi.org/10.3390/molecules25010059

Karamaouna, F., Kimbaris, A., Michaelakis, A., Papachristos, D., Polissiou, M., Papatsakona, P., Tsora, E., Miller, T., 2013. Insecticidal activity of plant essential oils against the vine mealybug, Planococcus ficus. J. Insect Sci. 13, 142. https://doi.org/10.1673/031.013.14201

Krzyżowski, M., Baran, B., Łozowski, B., Francikowski, J., 2020. The effect of Rosmarinus officinalis essential oil fumigation on biochemical, behavioral, and physiological parameters of Callosobruchus maculatus. Insects 11(6), 344. https://doi.org/10.3390/insects11060344

Kumar, S., Wahab, N., Warikoo, R., 2011. Bioefficacy of Mentha piperita essential oil against dengue fever mosquito Aedes aegypti L. Asian Pac. J. Trop. Biomed. 1, 85–88. https://doi.org/https://doi.org/10.1016/S2221-1691(11)60001-4

Mackled, M.I., EL-Hefny, M., Bin-Jumah, M., Wahba, T.F., Allam, A.A., 2019. Assessment of the toxicity of natural oils from Mentha piperita, Pinus roxburghii, and Rosa spp. against three stored product insects. Processes. 7(11), 861. https://doi.org/10.3390/pr7110861

Mahendran, G., Rahman, L., 2020. Ethnomedicinal, phytochemical and pharmacological updates on Peppermint (Mentha× piperita L.)—A review. Phyther. Res. 34, 2088–2139. https://doi.org/10.1002/ptr.6664

Manh, H. D., Tuyet, O. T. 2020. Larvicidal and Repellent Activity of Mentha arvensis L. Essential Oil against Aedes aegypti. Insects 11(3), 198. https://doi.org/10.3390/insects11030198

Manimaran, A., Cruz, M. M. J. J., Muthu, C., Vincent, S., Ignacimuthu, S., 2012. Larvicidal and knockdown effects of some essential oils against Culex quinquefasciatus Say, Aedes aegypti (L.) and Anopheles stephensi (Liston). Adv. Biosci. Biotechnol. 03(07), 855–862. https://doi.org/10.4236/abb.2012.37106

Nazar, N., Howard, C., Slater, A., Sgamma, T., 2022. Challenges in Medicinal and Aromatic Plants DNA Barcoding—Lessons from the Lamiaceae. Plants 11(1), 137. https://doi.org/10.3390/plants11010137

Pavela, R., 2015. Acute toxicity and synergistic and antagonistic effects of the aromatic compounds of some essential oils against Culex quinquefasciatus Say larvae. Parasitol. Res. 114(10), 3835–3853. https://doi.org/10.1007/s00436-015-4614-9

Prakash, O.M., Chandra, M., Pant, A.K., Rawat, D.S., 2016. Mint (Mentha spicata L.) oils, in: Essential Oils in Food Preservation, Flavor and Safety. Elsevier, pp. 561–572. ISBN 9780124166448.

Radwan, M.A., El-Zemity, S.R., Mohamed, S.A., Sherby, S.M., 2008. Larvicidal activity of some essential oils, monoterpenoids and their corresponding N-methyl carbamate derivatives against Culex pipiens (Diptera: Culicidae). Int. J. Trop. Insect Sci. 28(2), 61–68. https://doi.org/10.1017/s1742758408962366

Rai, V. K., Sinha, P., Yadav, K. S., Shukla, A., Saxena, A., Bawankule, D. U., Tandon, S., Khan, F., Chanotiya, C. S., Yadav, N. P., 2020. Anti-psoriatic effect of Lavandula angustifolia essential oil and its major components linalool and linalyl acetate. J. Ethnopharmacol. 261, 113127. https://doi.org/10.1016/j.jep.2020.113127

Ramos da Silva, L. R., Ferreira, O. O., Cruz, J. N., de Jesus Pereira Franco, C., Oliveira Dos Anjos, T., Cascaes, M. M., Almeida da Costa, W., Helena de Aguiar Andrade, E., & Santana de Oliveira, M., 2021. Lamiaceae Essential Oils, Phytochemical Profile, Antioxidant, and Biological Activities. Evid Based Complement Alternat Med.: eCAM, 2021, 6748052. https://doi.org/10.1155/2021/6748052

Samarasekera, R., Weerasinghe, I.S., Hemalal, K.P., 2008. Insecticidal activity of menthol derivatives against mosquitoes. Pest. Manag. Sci. 64, 290-295. https://doi.org/10.1002/ps.1516

Sharma, S., Loach, N., Gupta, S., Mohan, L., 2022. Evaluation of larval toxicity, mode of action and chemical composition of citrus essential oils against Anopheles stephensi and Culex

quinquefasciatus. Biocatal. Agric. Biotechnol. 39, 102284. https://doi.org/10.1016/j.bcab.2022.102284

Tabari, M.A., Youssefi, M.R., Esfandiari, A., Benelli, G., 2017. Toxicity of β-citronellol, geraniol and linalool from Pelargonium roseum essential oil against the West Nile and filariasis vector Culex pipiens (Diptera: Culicidae). Res. Vet. Sci. 114, 36–40. https://doi.org/https://doi.org/10.1016/j.rvsc.2017.03.001

Traboulsi, A.F., Taoubi, K., El-Haj, S., Bessiere, J., Rammal, S., 2002. Insecticidal properties of essential plant oils against the mosquito Culex pipiens molestus (Diptera: Culicidae). Pest. Manag. Sci., 58, 491-495. https://doi.org/10.1002/ps.486

WHO. 2005. Guidelines for laboratory and field testing of mosquito larvicides. World Health Organization, 1–41. Retrieved from http://whqlibdoc.who.int/hq/2005/WHO_CDS_WHOPES_GCDPP_2005.13.pdf?ua=1

WHO. 2016. Test procedures for insecticide resistance monitoring in malaria vector mosquitoes, (Second edition) (Updated June 2018). pp. 1–48. https://apps.who.int/iris/bitstream/handle/10665/250677/9789241511575-eng.pdf

Zahran, H.E.-D.M., Abou-Taleb, H.K., Abdelgaleil, S.A.M., 2017. Adulticidal, larvicidal and biochemical properties of essential oils against Culex pipiens L. J. Asia. Pac. Entomol. 20(1), 133–139. https://doi.org/10.1016/j.aspen.2016.12.006

Zeghib, F., Tine-Djebbar, F., Zeghib, A., Bachari, K., Sifi, K., Soltani, N., 2020. Chemical Composition and Larvicidal Activity of Rosmarinus officinalis Essential Oil Against West Nile Vector Mosquito Culex pipiens (L.). J. Essent. Oil Bear. Plants 23(6), 1463–1474. https://doi.org/10.1080/0972060X.2020.1860138

Zhu, L., Tian, Y., 2013. Chemical composition and larvicidal activity of essential oil of Artemisia gilvescens against Anopheles anthropophagus. Parasitol. Res. 112(3), 1137–1142. https://doi.org/10.1007/s00436-012-3243-9

Larvicidal and Adulticidal Activity of Essential Oils from Plants of the Lamiaceae Family against the West Nile Virus Vector, Culex pipiens (Diptera: Culicidae)

ORIGINALITY REPORT

16%

SIMILARITY INDEX

PRIMARY SOURCES

1	Hossam El-Din M. Zahran, Hamdy K. Abou-Taleb, Samir A.M. Abdelgaleil. "Adulticidal, larvicidal and biochemical properties of essential oils against Culex pipiens L", Journal of Asia-Pacific Entomology, 2017<br>Crossref	43 words — 1%
2	www.mdpi.com<br>Internet	37 words — 1%
3	www.science.gov<br>Internet	36 words — 1%
4	www.ncbi.nlm.nih.gov<br>Internet	27 words — 1%
5	iwaponline.com<br>Internet	26 words — 1%
6	WWW.mdpi.com<br>Internet	23 words — 1%
7	Toshio KAWAI, Tsuneyuki YAMAUCHI, Yuriko MIYAMA, Haruhiko SAKURAI, Hirohiko UKAI, Shiro TAKADA, Fumiko OHASHI, Masayuki IKEDA. "Benzyl Alcohol as a	22 words — 1%

Marker of Occupational Exposure to Toluene", Industrial Health, 2007
Crossref

8 Leandro P. França, Ana Claudia F. Amaral, Aline de S. Ramos, José Luiz P. Ferreira et al. "Piper capitarianum essential oil: a promising insecticidal agent for the management of Aedes aegypti and Aedes albopictus", Environmental Science and Pollution Research, 2020 — 20 words — < 1%
Crossref

9 www.sciencepub.net — 20 words — < 1%
Internet

10 link.springer.com — 19 words — < 1%
Internet

11 Shalika Rathore, Srijana Mukhia, Smita Kapoor, Vinod Bhatt, Rakshak Kumar, Rakesh Kumar. "Seasonal variability in essential oil composition and biological activity of Rosmarinus officinalis L. accessions in the western Himalaya", Scientific Reports, 2022 — 18 words — < 1%
Crossref

12 www.entomoljournal.com — 18 words — < 1%
Internet

13 www.hindawi.com — 16 words — < 1%
Internet

14 www.frontiersin.org — 15 words — < 1%
Internet

15 Ahmed H. Tantawy, Shaimaa M. Farag, Lamees Hegazy, Hong Jiang, Man-Qun Wang. "The larvicidal activity of natural inspired piperine-based — 14 words — < 1%

dienehydrazides against Culex pipiens", Bioorganic Chemistry, 2020
Crossref

16	Mohamed Habib Oueslati, Nael Abutaha, Faraj Al-Ghamdi, Imededdine Arbi Nehdi et al. "Analysis of the chemical composition and in vitro cytotoxic activities of the essential oil of the aerial parts of Lavandula atriplicifolia Benth", Journal of King Saud University - Science, 2020<br>Crossref	13 words — < 1%
17	Abdulrhman A. Almadiy, Gomah E. Nenaah. "Bioactivity and safety evaluations of Cupressus sempervirens essential oil, its nanoemulsion and main terpenes against Culex quinquefasciatus Say", Environmental Science and Pollution Research, 2021<br>Crossref	12 words — < 1%
18	Adam Kowalczyk, Elżbieta Piątkowska, Piotr Kuś, Zvonimir Marijanović, Igor Jerković, Carlo I.G. Tuberoso, Izabela Fecka. "Volatile compounds and antibacterial effect of commercial mint cultivars - chemotypes and safety", Industrial Crops and Products, 2021<br>Crossref	12 words — < 1%
19	Hojjat Jahanian, Niloofar Kahkeshani, Alireza Sanei-Dehkordi, Murray B. Isman, Mina Saeedi, Mahnaz Khanavi. " as a natural insecticide: a review ", International Journal of Pest Management, 2022<br>Crossref	12 words — < 1%
20	bioone.org<br>Internet	12 words — < 1%
21	Nehad E.M. Taktak, Mohamed E.I. Badawy. "Potential of hydrocarbon and oxygenated monoterpenes against Culex pipiens larvae: Toxicity,	11 words — < 1%

biochemical, pharmacophore modeling and molecular docking studies", Pesticide Biochemistry and Physiology, 2019
Crossref

22 Welton Aaron de Almeida, Isabella Coimbra Vila Nova, Jéssica da Silva Nascimento, Maiara Celine de Moura et al. "Effects of Plectranthus barbatus leaf extract on survival, digestive proteases, midgut morphophysiology and gut microbiota homeostasis of Aedes aegypti larvae", South African Journal of Botany, 2021 — 11 words — < 1%
Crossref

23 onlinelibrary.wiley.com — 11 words — < 1%
Internet

24 "Role of plants and plant based products towards the control of insect pests and vectors: A novel review", Journal of Coastal Life Medicine, 2016 — 10 words — < 1%
Crossref

25 Liang Zhu, Yingjuan Tian. "Chemical composition and larvicidal activity of essential oil of Artemisia gilvescens against Anopheles anthropophagus", Parasitology Research, 2012 — 10 words — < 1%
Crossref

26 M. Shimoda. "Volatile Flavor Compounds of Sweetened Condensed Milk", Journal of Food Science, 8/2001 — 10 words — < 1%
Crossref

27 Marimuthu Govindarajan, Rajamohan Sivakumar, Mohan Rajeswary, Kaliyan Veerakumar. "Mosquito larvicidal activity of thymol from essential oil of Coleus aromaticus Benth. against Culex tritaeniorhynchus, Aedes albopictus, and Anopheles subpictus (Diptera: Culicidae)", Parasitology Research, 2013 — 10 words — < 1%
Crossref

28 Petya K. Christova, Ivaiyla N. Dincheva, Slavtcho B. Slavov, Ana M. Dobreva et al. "Evaluation of growth response of phytopathogens Alternaria alternata, Diaporthe nobilis and Phytophthora plurivora to inhibitory potential of three essential oils of Monarda didyma genotypes", Journal of Plant Diseases and Protection, 2021 — 10 words — < 1%
Crossref

29 Samia M.M. Mohafrash, Sahar Ahmed Fallatah, Shaimaa Mahmoud Farag, Abdel-Tawab H. Mossa. "Mentha spicata essential oil nanoformulation and its larvicidal application against Culex pipiens and Musca domestica", Industrial Crops and Products, 2020 — 10 words — < 1%
Crossref

30 bio-protocol.org — 10 words — < 1%
Internet

31 preview-behavioralandbrainfunctions.biomedcentral.com — 10 words — < 1%
Internet

32 repository.up.ac.za — 10 words — < 1%
Internet

33 www.scielo.br — 10 words — < 1%
Internet

34 Paula Sampaio Araújo, Maxwell Batista Caixeta, Aryelle Canedo, Eloiza da Silva Nunes, Caio Monteiro, Thiago Lopes Rocha. "Toxicity of plant-based silver nanoparticles to vectors and intermediate hosts: Historical review and trends", Science of The Total Environment, 2022 — 9 words — < 1%
Crossref

35 www.isnff-jfb.com
Internet

9 words — < 1%

36 www.jvbd.org
Internet
9 words — < 1%

37 Guido Flamini, Pier Luigi Cioni, Ivano Morelli, Mario Macchia, Lucia Ceccarini. " Main Agronomic−Productive Characteristics of Two Ecotypes of L. and Chemical Composition of Their Essential Oils ", Journal of Agricultural and Food Chemistry, 2002
Crossref
8 words — < 1%

38 Radhika Samarasekera. "Insecticidal activity of menthol derivatives against mosquitoes", Pest Management Science, 03/2008
Crossref
8 words — < 1%

39 Sen-Sung Cheng, Ju-Yun Liu, Kun-Hsien Tsai, Wei-June Chen, Shang-Tzen Chang. " Chemical Composition and Mosquito Larvicidal Activity of Essential Oils from Leaves of Different Provenances ", Journal of Agricultural and Food Chemistry, 2004
Crossref
8 words — < 1%

40 Tharsius Raja William Raja, Pathalam Ganesan, Munusamy Rajiv Gandhi, Veeramuthu Duraipandiyan et al. "Effect of compound Musizin isolated from Rhamnus wightii Wight and Arn on the immature stages of filarial vector mosquito Culex quinquefasciatus Say (Diptera: Culicidae) and its non-target studies", Biocatalysis and Agricultural Biotechnology, 2018
Crossref
8 words — < 1%

41 Zhaojun Sheng, Rongchao Jian, Fangying Xie, Bin Chen et al. "Screening of larvicidal activity of 53 essential oils and their synergistic effect for the improvement
8 words — < 1%

of deltamethrin efficacy against Aedes albopictus", Industrial Crops and Products, 2020
Crossref

42 meridian.allenpress.com
Internet
8 words — < 1%

43 stars.library.ucf.edu
Internet
8 words — < 1%

44 www.fspublishers.org
Internet
8 words — < 1%

45 www.mapress.com
Internet
8 words — < 1%

46 www.tandfonline.com
Internet
8 words — < 1%

47 E. Evergetis. "Chemical composition and larvicidal activity of essential oils from six Apiaceae family taxa against the West Nile virus vector Culex pipiens", Parasitology Research, 02/26/2009
Crossref
7 words — < 1%

48 Gillij, Y.G.. "Mosquito repellent activity of essential oils of aromatic plants growing in Argentina", Bioresource Technology, 200805
Crossref
7 words — < 1%

49 Sara El Kharraf, Soukaina El-Guendouz, Abdellah Farah, Bahia Bennani, Maria C. Mateus, El Mestafa El Hadrami, M. Graça Miguel. "Hydrodistillation and simultaneous hydrodistillation-steam distillation of Rosmarinus officinalis and Origanum compactum: Antioxidant, anti-inflammatory, and antibacterial effect of the essential oils", Industrial Crops and Products, 2021
Crossref
7 words — < 1%

50	"Recent Advances in Environmental Science from the Euro-Mediterranean and Surrounding Regions", Springer Nature, 2018<br>Crossref	6 words — < 1%
51	Advances in Plant Biopesticides, 2014.<br>Crossref	6 words — < 1%
52	Giovanni Benelli, Roman Pavela, Rianasoambolanoro Rakotosaona, Emmanuel Randrianarivo, Marcello Nicoletti, Filippo Maggi. " Chemical composition and insecticidal activity of the essential oil from endemic to Madagascar ", Natural Product Research, 2017<br>Crossref	4 words — < 1%

EXCLUDE QUOTES	OFF	EXCLUDE SOURCES	OFF
EXCLUDE BIBLIOGRAPHY	ON	EXCLUDE MATCHES	OFF
